# c-MYC-Induced AP4 Attenuates DREAM-Mediated Repression by p53

**DOI:** 10.3390/cancers15041162

**Published:** 2023-02-11

**Authors:** Markus Kaller, Wenjing Shi, Heiko Hermeking

**Affiliations:** 1 Experimental and Molecular Pathology, Institute of Pathology, Ludwig-Maximilians-University München, D-80337 Munich, Germany; 2 German Cancer Consortium (DKTK), Partner Site Munich, D-80336 Munich, Germany; 3 German Cancer Research Center (DKFZ), D-69120 Heidelberg, Germany

**Keywords:** c-MYC, AP4, TFAP4, p53, p21, DREAM complex, ARF/INK4A, senescence, cell cycle progression, breast cancer, E2F target genes

## Abstract

**Simple Summary:**

Deregulated expression of the c-MYC oncogene activates the tumor suppressor p53, which has been suggested to represent a failsafe mechanism against the uncontrolled expansion of tumor cells. Here, we analyzed the role of the c-MYC-induced *TFAP4/AP4* gene in this context using a genetic approach in MCF-7 breast cancer cells. Inactivation of *AP4* resulted in elevated levels of both spontaneous and c-MYC-induced DNA damage, senescence, and diminished cell proliferation. Inactivation of *p53* in *AP4*-deficient cells reverted senescence and proliferative defects. Furthermore, loss of AP4 resulted in p53-dependenct, enhanced repression of DREAM and E2F target genes after the induction of c-MYC, which could be abrogated by the concomitant depletion of p21 or the DREAM complex component LIN37. These p53-dependent effects were reflected on the levels of gene expressions and clinical associations in primary breast cancer tumors from patient cohorts. Our results established AP4 as a pivotal factor at the crossroads of c-MYC, E2F, and p53-mediated target gene regulation.

**Abstract:**

Background: The deregulated expression of the c-MYC oncogene activates p53, which is presumably mediated by ARF/INK4, as well as replication-stress-induced DNA damage. Here, we aimed to determine whether the c-MYC-inducible AP4 transcription factor plays a role in this context using a genetic approach. Methods: We used a CRISPR/Cas9 approach to generate *AP4*- and/or *p53*-deficient derivatives of MCF-7 breast cancer cells harboring an ectopic, inducible c-MYC allele. Cell proliferation, senescence, DNA damage, and comprehensive RNA expression profiles were determined after activation of c-MYC. In addition, we analyzed the expression data from primary breast cancer samples. Results: Loss of *AP4* resulted in elevated levels of both spontaneous and c-MYC-induced DNA damage, senescence, and diminished cell proliferation. Deletion of *p53* in *AP4*-deficient cells reverted senescence and proliferation defects without affecting DNA damage levels. RNA-Seq analyses showed that loss of *AP4* enhanced repression of DREAM and E2F target genes after p53 activation by c-MYC. Depletion of p21 or the DREAM complex component LIN37 abrogated this effect. These p53-dependent effects were conserved on the level of clinical and gene expression associations found in primary breast cancer tumors. Conclusions: Our results establish AP4 as a pivotal factor at the crossroads of c-MYC, E2F, and p53 target gene regulation.

## 1. Introduction

The c-MYC transcription factor is encoded by a proto-oncogene, which shows elevated and/or deregulated expression in more than 70% of all cancers [1]. In breast cancer, c-MYC is expressed at elevated levels in 30–50% and amplified in nearly 15% of cases [2].

Oncogenic, deregulated c-MYC expression induces unscheduled DNA replication [3]. Furthermore, activation of c-MYC leads to the accumulation of the p53 tumor suppressor protein, which mediates c-MYC-induced apoptosis [4,5]. Evidence from mouse models suggests that post-transcriptional activation of p53 is mediated by Arf/Ink4 activation, which blocks the p53-specific E3-ligase Mdm2 [6]. The relevance of INK4A/ARF for p53 activation by c-MYC in human cells has not been unambiguously clarified [7]. The *INK4A/B* locus, which encodes p14/ARF and the CDK-inhibitor p16, is frequently inactivated in cancer cell lines due to DNA methylation, mutations, or chromosomal deletions [8]. In human cells, p53 activation induced by c-MYC has been shown to occur due to DNA damage, which is caused by oncogene-induced replication stress [3,9,10]. Interestingly, c-MYC abrogates a p53-mediated cell cycle arrest as it induces DNA replication in the presence of high p53 activity [11]. However, the mechanism underlying the ability of c-MYC to override the anti-proliferative activity of p53 has remained largely unknown.

We identified the *AP4* gene as a direct target of c-MYC in the breast cancer cell line MCF-7 [12]. *AP4* encodes a BR-HLH-LZ transcription factor that binds to so-called E-boxes (CAGCTG) in the vicinity of target genes [13] and mediates their repression or induction [14]. The CDK inhibitor *CDKN1A*/*p21* and *MDM2* represent targets for direct repression by AP4 [12,15,16] and are directly induced by p53 [17,18]. So far, the role and outcome of their antagonistic regulation by AP4 and p53 have not been studied.

The DREAM complex has been implicated in the repression of gene expression by p53 [19]. DREAM is composed of dimerization partner (DP), retinoblastoma-(RB)-like, E2F4-5, and MuvB proteins. It is involved in the down-regulation of numerous genes, which encode factors mediating cell cycle progression and checkpoint control. Mechanistically, the induction of p21 by p53 leads to hypo-phosphorylation of the pRB-related proteins p107/RBL1 and p130/RBL2, which then associate with E2F4-5/DP and additional proteins, such as LIN54, LIN37, LIN9, and RBBP4 [19]. The resulting DREAM complex then represses transcription by binding to E2F or cell cycle genes homology region (CHR) promoter sites. Conversely, the repression of *p21* by AP4 is predicted to attenuate DREAM complex activity and alleviate the repression of its target genes. This suggests that the activation of AP4 may contribute to the abrogation of p53 function by c-MYC.

The *p53*-proficient MCF-7 breast cancer cell line, which is deficient of *p14/ARF* and *p16*, represents a luminal, estrogen-receptor-positive breast cancer subtype in which endogenous c-MYC expression is amenable to regulation by both estrogens and anti-estrogens [12,20,21]. Ectopic expression of c-MYC has been shown to override the cell cycle arrest caused by estrogen antagonists in MCF-7 cells [12,22]. Here we determined the role of AP4 and p53 downstream of c-MYC activation in MCF-7 cells by using a genetic approach. When c-MYC was ectopically activated in MCF-7 cells with the deletion of *AP4*, it resulted in decreased proliferation and increased induction of p21, which was associated with increased DNA damage and senescence when compared with c-MYC activation in *AP4*-proficient cells. Additional deletion of *p53* fully reverted the proliferative defects and the senescent phenotype of *AP4*-deficient cells. Comprehensive gene expression profiling revealed that c-MYC-induced AP4 mitigates DREAM-mediated gene repression. Thereby, AP4 presumably limits the inhibitory effects of p53 and contributes to productive cell cycle progression after activation of c-MYC.

## 2. Materials and Methods

### 2.1. Cell Culture and Treatments

The human breast cancer cell line MCF-7 and its derivatives generated here were grown in Dulbecco’s modified Eagle’s medium (DMEM, Invitrogen, Carlsbad, CA, USA) supplemented with 10% fetal bovine serum (FBS) (Invitrogen), 100 units/mL penicillin, and 0.1 mg/mL streptomycin in an incubator with 5% CO_2_ at 37 °C. siRNAs and negative controls were transfected with Lipofectamine RNAi MAX Transfection Reagent (Invitrogen, Carlsbad, CA, USA) at a final concentration of 12.5 nM. Hygromycin with a final concentration of 0.25 mg/mL was used to maintain MCF-7 cells harboring pRTR-c-MYC vectors. The anti-estrogen ICI 182,780/Fulvestrant [23] was applied at a final concentration of 1 μM for 72 h before doxycycline (DOX, Sigma-Aldrich, St. Louis, MO, USA) dissolved in water was applied at a final concentration of 1 µg/mL. FlexiTube siRNAs (consisting of a pool of 4 different siRNAs) targeting *p21* or *LIN37*) and control siRNAs were purchased from Qiagen (Hilden, Germany). The sequence information of guide RNAs used for *AP4* and *p53* deletion are listed in Tables S1 and S2.

### 2.2. Generation of AP4-Deficient MCF-7 Cells

MCF-7 cells harboring a pRTR-c-*MYC* expression vector (MCF-7/pRTR-c-*MYC*), which ectopically expresses *c-MYC* and *mRFP* from a bidirectional, DOX-inducible promoter, were previously described in [12]. Deletion of exon 2 of the *AP4* gene was achieved as described in [24]. In short, 48 h after transfection with pSpCas9(BB)-2A-GFP vectors [25], each expressing 1 of 3 guide RNAs (Table S1), single GFP-positive cells were sorted into individual 96-wells using a FACSARIA cell sorter (BD Biosystems, Franklin Lakes, NJ, USA). Single-cell clones were expanded for two weeks and subjected to Western blot analysis to verify the loss of AP4 protein expression.

### 2.3. Generation of p53-Deficient Cell Pools

In order to delete *p53*, a CRISPR/Cas9 approach was used in both *AP4* wild-type and *AP4*-deficient cells. A guide RNA targeting exon 3 of the *TP53* gene (Table S2, [26]) was cloned into pSpCas9(BB)-2A-GFP. Cells were seeded at 2 × 10^5^ cells/6-well and transfected with 2 µg pSpCas9(BB)-2A-GFP-p53 (kind gift from Peter Jung) using Lipofectamine LTX (Invitrogen, Carlsbad, CA, USA). Cells were transferred into 25T flasks 48 h after transfection. Nutlin-3a (10 µM) was applied for two weeks to select *p53*-deficient cells. The resulting *p53*-deficient cell pools were subjected to Western Blot analysis to verify the loss of p53 protein expression.

### 2.4. Western Blot Analysis

Samples were lysed in RIPA buffer supplemented with Mini Protease Inhibitor (Roche, Basel, Switzerland), sonicated, and centrifuged at 16,000× *g* at 4 °C for 15 min. A Micro BCA Protein Assay Kit (Pierce, Appleton, WI, USA) was used to determine the protein concentrations. 60–70 μg protein per sample was used for SDS-PAGE. The antibodies used are listed in Table S3. Uncropped Western blot membranes are displayed in Figure S1.

### 2.5. Beta-Galactosidase (β-Gal) Staining

β-gal staining was performed using a Senescence β-Galactosidase Staining Kit (#9860, Cell Signaling Technology, Danvers, MA, USA) according to the manufacturer’s instructions. Cells were seeded into 6-well plates at a density of 2 × 10^5^ cells/well. Cells were washed twice with HBSS before fixation for 30 min at room temperature. The final pH of the staining solution containing X-gal was adjusted to around pH 5.9–6.1. After overnight incubation at 37 °C, cells were stained and imaged by using a microscope (Axiovert 25, Zeiss, Jena, Germany) with Axiovision software (Version 4.8, Zeiss).

### 2.6. Immunofluorescence Analysis

Cells were seeded on sterile round 12 mm coverslips in a 6-well plate at a density of 2 × 10^5^ cells/well. Cells were grown to 40–60% confluency, washed twice with PBS, fixed with 4% paraformaldehyde for 10 min, permeabilized with 0.2% Triton X-100 for 5 min, then blocked in 1% BSA/PBS for 1 h at room temperature. DNA damage foci were detected by a γH2AX-specific antibody incubated at 4 °C overnight. Cellular chromatin was stained by DAPI (Roche, Basel, Switzerland). For F-Actin staining, samples were incubated with Alexa Fluor 488-coupled phalloidin (Thermo Fisher, Waltham, MA, USA) in 1% BSA in HBSS for 45 min at room temperature and washed 3× with 1 mL 0.05% Tween-20 in PBS for 5 min. Stained cells were covered with ProLong Gold antifade (Invitrogen) and recorded with a confocal microscope (LSM 700, Zeiss) equipped with 405 nm, 488 nm, and 555 nm lasers using a Plan-Apochromat 63×/1.40 Oil DIC M27 objective and ZEN 2009 software. After image acquisition (2048 × 2048 pixel 16 bit), original LSM files were converted into TIFF files. Foci quantification was performed with the Image J software (Version 1.53t). Nuclei with more than 10 foci were considered γH2AX-positive. The fluorescence intensity was normalized to DAPI. For each condition, at least three microscope fields with a total of 150 cells were quantified. The antibodies used are listed in Table S3.

### 2.7. Comet Assay

Comet assays were performed using the Comet Assay Kit (3-well slides, ab238544, Abcam, Boston, MA, USA), as described previously [24], and imaged by using a microscope (Axiovert 25, Zeiss, Jena, Germany) with Axiovision software (Version 4.8, Zeiss).

### 2.8. RNA Isolation and Quantitative Real-Time Polymerase Chain Reaction (qPCR) Analysis

Cells were seeded at 0.5 × 10^5^ cells/mL in 6-well plates and treated with ICI for 72 h. Subsequently, ectopic expression of c-MYC was induced with DOX for the indicated time points in the presence of ICI. A High Pure RNA Isolation Kit (Roche) was used to isolate RNA from cells. For cDNA synthesis, the Verso cDNA kit (ThermoScientific, Waltham, MA, USA) was used according to the manufacturer’s instructions. A total of 1 µg of RNA and anchored oligo-dT primers were used for reverse transcription. For qPCR analysis, a LightCycler 480 Instrument II (Roche) and Fast SYBR Green Master Mix (Applied Biosystems, Foster City, CA, USA) were used. Relative gene expression was determined using the 2^−ΔΔCt^ method [27]. The individual mRNA levels were normalized to β-actin. All qPCR primers are listed in Table S4.

### 2.9. Assessment of Proliferation by Real-Time Impedance Measurement

Cell proliferation was measured with impedance measurements (X-celligence RTCA DP, Roche). Cells were seeded at a density of 6 × 10^3^ cells in a 96-well microtiter plate (E-Plate Cardio 96, Agilent, Santa Clara, CA, USA) in triplicate and subjected to the indicated treatments. Cellular impedance was measured every 60 min for a period of up to 72 h with the X-celligence system (Roche). In parallel, cells were also seeded into 48-well plates in triplicate and counted at the end time point using a Neubauer chamber to validate impedance measurements.

### 2.10. Colony Formation Assay

A total of 1 × 10^3^ cells per well were seeded into 6-well plates and cultured for 14 days. Subsequently, cells were stained with crystal violet after fixation.

### 2.11. Transcriptomic Analysis

Total RNA from MCF-7/pRTR-c-MYC cells was isolated using a High Pure RNA Isolation Kit (Roche). Random primed cDNA libraries were constructed and sequenced using the NovaSeq 6000 (Illumina, San Diego, CA, USA) platform by GATC (Konstanz, Germany). Each sample was covered by at least 30 million paired-end read pairs of 150 bp length. RNA-Seq FASTQ files were processed using the RNA-Seq module implemented in the CLC Genomics Workbench v20.0.2 software (Qiagen Bioinformatics, Dusseldorf, Germany) and mapped to the GRCh38/hg38 human reference genome and its associated gene and transcript annotation (ENSEMBL) using the settings mismatch cost = 2, insertion cost = 2, deletion cost = 3, length fraction = 0.8, and similarity fraction = 0.8. RNA-Seq data were filtered to exclude weakly expressed transcripts with less than 20 mapped exon reads in all samples from the analysis and subjected to upper quartile normalization using the R/Bioconductor RUVSeq (remove unwanted variation from RNA-Seq data) package as described in Risso et al. [28]. Differential gene expression analysis was performed with DESeq2 [29] after normalization using the RUVg approach to remove variation between RNA samples resulting from differences in library preparation. Principal component analysis (PCA) was performed using the PCA functionality of the EDASeq R package as implemented in RUVSeq. Gene set enrichment analysis (GSEA) was performed with the fgsea R package [30]. Prior to GSEA, expression changes from low-count genes were adjusted using the ashr (adaptive shrinkage) estimator [31]. The significance of enrichments is presented by normalized enrichment scores (NES) and Benjamini-Hochberg-adjusted *p* values. Heat-maps were generated with Morpheus (Broad Institute, Cambridge, MA, USA). Gene sets were obtained from the Molecular Signatures database (MSigDB) [32].

### 2.12. Analysis of ChIP-Seq, RNA Expression, and Clinical Data from Public Databases

Direct regulation of DREAM targets was assessed by analysis of publicly available ChIP-Seq data for E2F4 (SRX194566, MCF-7), LIN9 (SRX4213896, MCF-10A), and RBL2 (SRX016031, IMR90) obtained from ChIP-Atlas (https://chip-atlas.org (accessed on 19 December 2022)) [33]. The Integrative Genomics Viewer (IGV) [34] was used for the visualization of ChIP-Seq profiles. For the analysis of human breast cancer samples, we retrieved expression and clinical data from the TCGA-BRCA cohort [35]. The statistics for forest plots were calculated with a log-rank test. For binary classification of cases (high/low expression), the Survminer R package (https://CRAN.R-project.org/package=survminer (accessed on 18 February 2020)) was used to determine optimal cutoff values.

### 2.13. Statistical Analysis

Results are presented as mean +/− standard deviation (SD). Each set of experiments was repeated at least three times. A Student’s *t*-test was used to test the null hypothesis stating no significant differences between each individual parameter were measured. Differences were considered to be significant if *p* < 0.05. Statistics were performed with Prism 8 (GraphPad Software, San Diego, CA, USA).

## 3. Results

### 3.1. Generation and Characterization of AP4-and/or p53-Deficient MCF-7/pRTR-c-MYC Cell Lines

**Figure 1:**
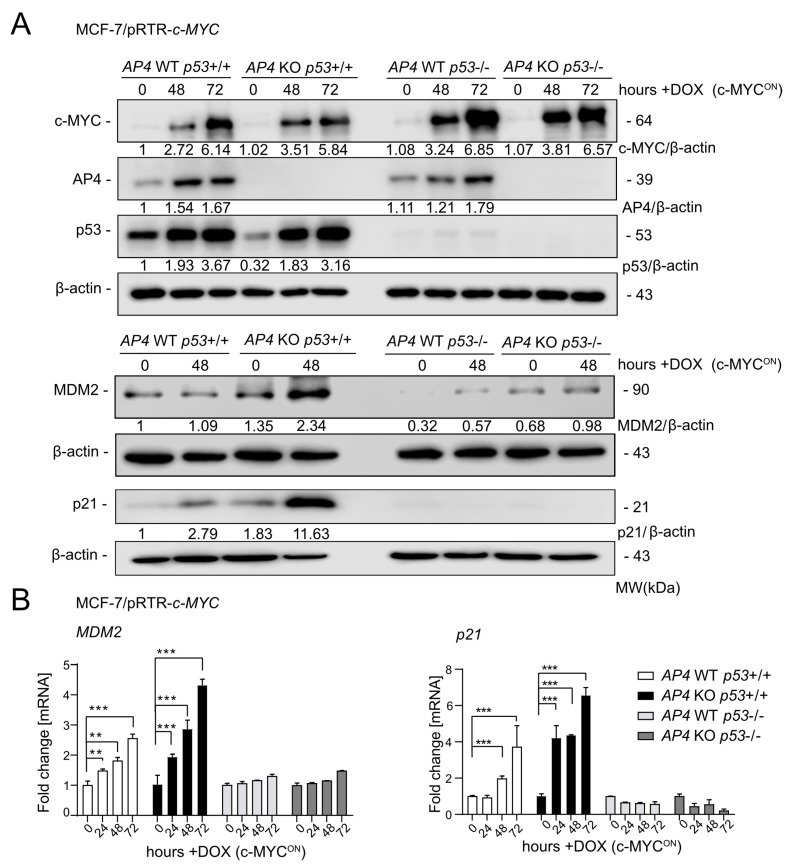
Effects of *AP4* and/or *p53* deletion on p21/CDKN1A and MDM2 expression after activation of c-MYC. (**A**) Western blot analysis of the indicated cells after treatment with DOX for the indicated periods. β-actin served as a loading control. (**B**) qPCR analysis of the indicated cells after treatment with DOX for the indicated period. The mean +/− SD (*n* = 3) is provided with **: *p* < 0.01, ***: *p* < 0.001. The uncropped blots are shown in Figure S1.

In order to study the role of AP4 downstream of c-MYC in breast cancer cells, *AP4* was inactivated in MCF-7/pRTR-c-MYC cells using a CRISPR/Cas9 approach, as described earlier [24]. In addition, we inactivated *p53* in these cells by introducing InDel mutations into exon 3 of *p53* by CRISPR/Cas9, as described previously [26]. The induction of the ectopic c-*MYC* allele, activation of AP4, and loss of p53 expression was verified by Western blot analysis (Figure 1A). The c-MYC-induced elevation of p53 protein levels was similar in *AP4* wild-type and *AP4*-deficient cells, indicating that loss of AP4 had no significant effect on the c-MYC-mediated activation of p53. As expected, basal expression levels, as well as c-MYC-induced up-regulation of the direct p53 targets *p21* and *MDM2*, were highly dependent on the presence of p53 in these cells (Figure 1A(lower panel),B). Moreover, in *AP4*-deficient cells harboring wild-type p53, basal expression, as well as c-MYC-induced up-regulation of p21 and MDM2, were elevated when compared to *AP4* wild-type cells (Figure 1A). Similar results were obtained by qPCR analysis of *p21* and *MDM2* mRNA expression (Figure 1B). Hence, both p21 and MDM2 expression are antagonistically regulated by AP4 and p53 after induction of c-MYC in MCF-7 cells.

### 3.2. Loss of AP4 Suppresses Induction of Cell Proliferation by Ectopic c-MYC

**Figure 2:**
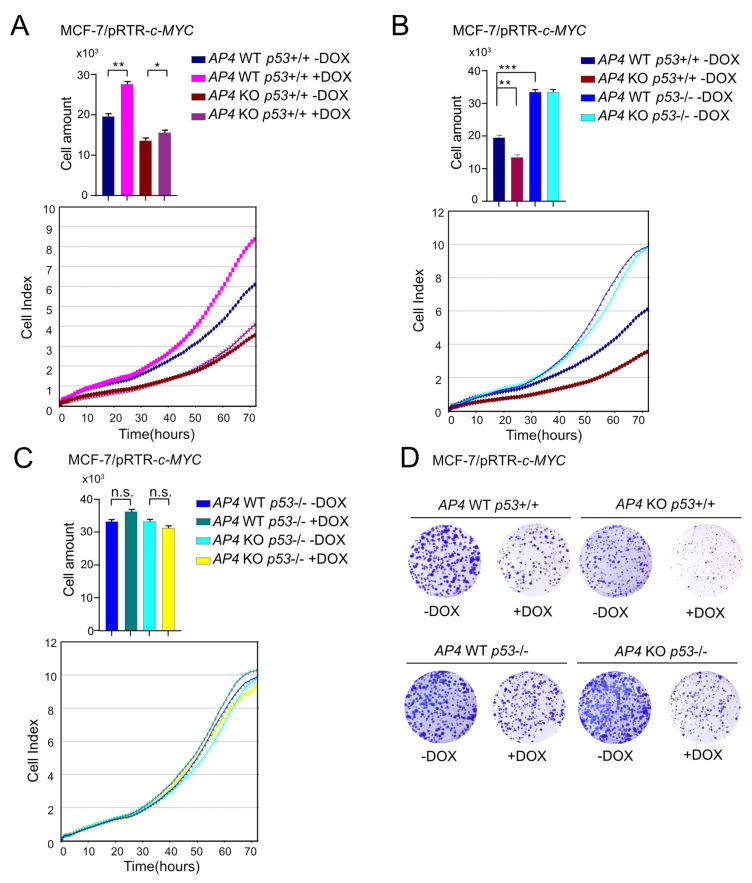
Effects of *AP4*- and *p53*-deficiency on basal and c-MYC-induced cell proliferation. (**A**–**C**) Cell proliferation of the indicated cell lines was determined by impedance measurement. Cell numbers were determined at 72 h. Results are presented as the mean +/− SD with *: *p* < 0.05, **: *p* < 0.01, ***: *p* < 0.001, n.s.: no significance. (**D**) Colony formation assays with the indicated cell lines. Cells are shown 14 days after seeding.

Activation of ectopic c-MYC by DOX treatment resulted in a significant increase in proliferation in MCF-7 cells with wild-type *AP4* and *p53* alleles, as shown by cellular impedance measurements. The induction of cell proliferation was severely diminished in *AP4*-deficient *p53* wild-type cells (Figure 2A). In the absence of ectopic c-MYC expression, the inactivation of *AP4* resulted in a pronounced decrease in cell proliferation when compared with *AP4*-proficient cells (Figure 2B). Interestingly, the deletion of *p53* not only reverted the proliferative defect of *AP4*-deficient cells but led to a strong increase in proliferative capacity independent of the *AP4* status (Figure 2B). Furthermore, an enhancement of cell proliferation by activation of ectopic c-MYC could not be observed after deletion of *p53* irrespective of the *AP4* status, presumably since *p53*-negative MCF-7 cells are already proliferating at the maximum rate (Figure 2C,D).

The p53-dependent proliferative defects of *AP4*-deficient cells were confirmed by assaying colony formation (Figure 2D). Here, the loss of *AP4* in *p53* wild-type cells resulted in decreased colony-forming capacity when compared to *AP4* wild-type cells. Conversely, the loss of *p53* dramatically increased colony formation. Furthermore, the deletion of *p53* in *AP4*-deficient cells reverted their decreased colony-forming capacity and resulted in colony numbers comparable to *p53*-deficient/*AP4* wild-type cells. Interestingly, prolonged activation of c-MYC for two weeks resulted in an overall decrease in the number and size of colonies, irrespective of *AP4* and *p53* status. However, while this suppression of colony formation was less pronounced in *AP4* wild-type/*p53*-deficient cells compared with *AP4/p53* wild-type cells, it was dramatically enhanced in *AP4/p53*-deficient cells, suggesting that *AP4/p53*-deficient cells were particularly sensitive to prolonged activation of c-MYC. Taken together, these results showed that the decreased proliferation of *AP4*-deficient MCF-7 cells was dependent on the presence of wild-type *p53*, suggesting that p53 acts as a major suppressor of proliferation in these cells. These findings were different from our previously published results obtained in *p53*-mutant CRC cell lines SW480 and DLD1 [24], where deletion of *AP4* resulted in a significantly diminished proliferative capacity.

### 3.3. Loss of AP4 Causes Senescence in Breast Cancer Cells, Which Is Dependent on Wild-Type p53

**Figure 3:**
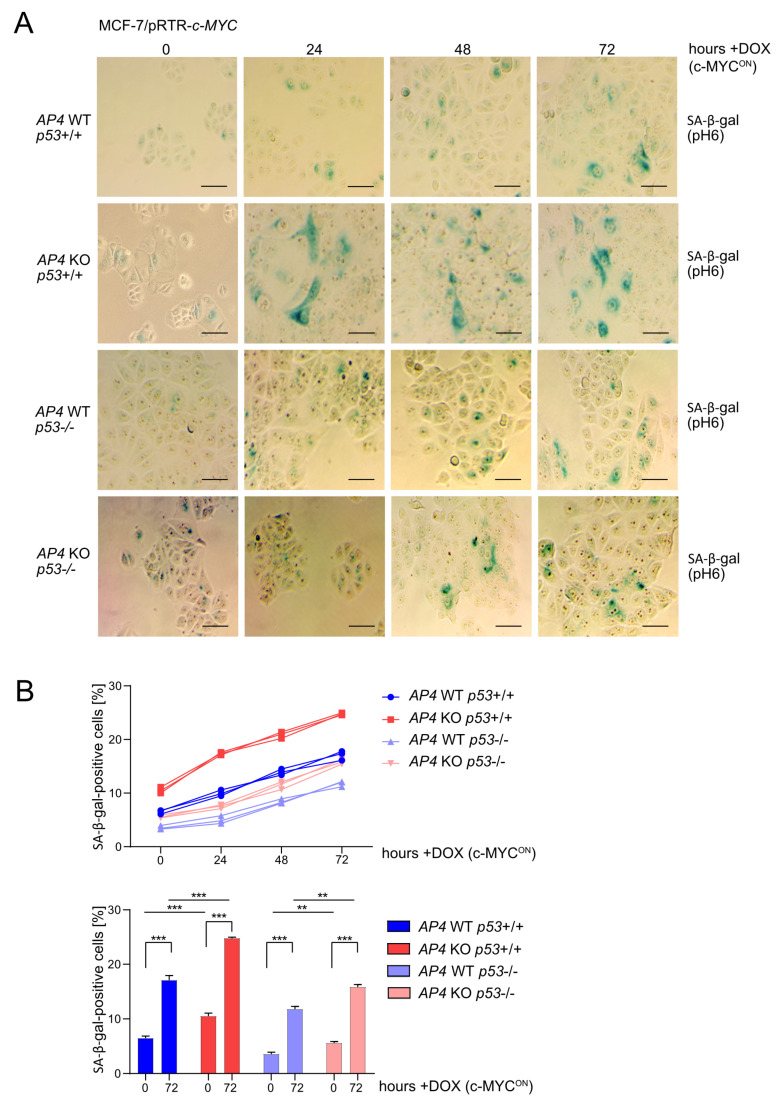
Effects of *AP4* and/or *p53* inactivation on basal and c-MYC-induced senescence. (**A**) Detection of basal (no DOX treatment) and c-MYC-induced (24, 48, and 72 h DOX treatment) senescence by β-gal detection at pH 6. Scale bars: 100 µm. (**B**) Quantification of β-gal detection as shown in (**A**). Three microscopic fields with 120 cells in total were evaluated. Results are presented as the mean +/− SD with **: *p* < 0.01, ***: *p* < 0.001.

Next, we analyzed whether the decreased proliferation of *AP4*-deficient cells was associated with increased senescence, as shown previously in CRC cell lines and MEFs [24,36]. The fraction of senescent, β-galactosidase positive cells was elevated in untreated *AP4*-deficient cells when compared with *AP4* wild-type cells (Figure 3A). Deletion of *p53* resulted in a decrease in basal senescence. Furthermore, the deletion of *p53* reverted the increased basal senescence levels observed in *AP4*-deficient cells. Induction of ectopic c-MYC for up to 72 h led to a significant increase in the fraction of senescent, β-galactosidase positive cells independently of the *AP4* or *p53* status (Figure 3A,B). However, in *AP4*-deficient/*p53* wild-type cells, the frequency of β-galactosidase positive cells was higher when compared with *AP4/p53* wild-type cells after activation of c-MYC for up to 72 h. Moreover, the deletion of *p53* reverted the increased senescence levels observed in *AP4*-deficient cells after activation of c-MYC. Taken together, these results showed that the loss of *AP4* results in increased senescence in MCF-7 cells and strongly suggested that functional p53 mediates, at least in part, the induction of a senescent phenotype in *AP4*-deficient cells. However, the relative increase in senescence after activation of c-MYC appeared to be largely independent of p53.

### 3.4. Deletion of AP4 or p53 Increases Spontaneous and c-MYC-Induced DNA Damage in Breast Cancer Cells

**Figure 4:**
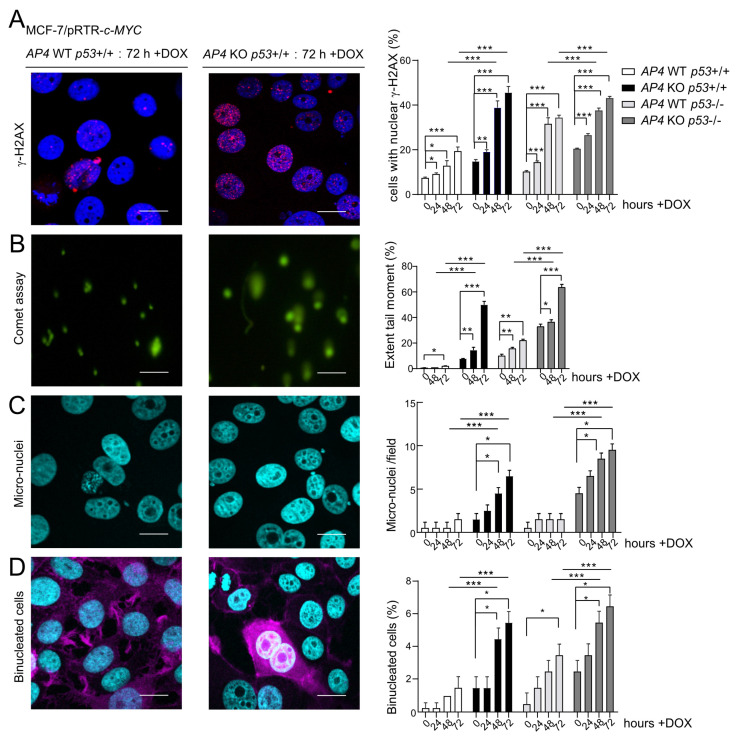
Effects of *AP4*- and/or *p53*-deficiency on basal and c-MYC-induced DNA damage. (**A**–**D**) Activation of c-MYC in the indicated cells by treatment with DOX for the indicated periods. (**A**) Evaluation of DNA damage by detection of γH2AX foci in five microscopic fields with 150 cells in total. Scale bars: 20 µm. (**B**) Evaluation of unrepaired DNA damage by Comet assays in ten microscopic fields with 150 cells in total. Scale bars: 10 µm. (**C**) Quantification of micronuclei by DAPI staining in five microscopic fields with 150 cells in total. Scale bars: 20 μm. (**D**) Detection of bi-nucleated cells by DAPI and F-actin staining in ten microscopic fields with 300 cells in total. Scale bars: 20 μm. (**A**–**D**) Representative images of all time points and genotypes analyzed are provided in Figures S2–S5. (**A**–**D**) Results are presented as the mean +/− SD with *: *p* < 0.05, **: *p* < 0.01, ***: *p* < 0.001.

We have previously shown that AP4 suppresses DNA damage, which occurs spontaneously or at an increased rate after c-MYC activation. AP4 suppresses DNA damage by directly and indirectly (via repressing miR-22) inducing MDC1/Mediator of DNA damage checkpoint 1 [24]. Therefore, we determined whether the loss of AP4 in breast cancer cells also increases DNA damage. Indeed, after the deletion of *AP4*, increased levels of γH2AX-positive nuclear foci were detected in MCF-7 cells (Figure 4A and Figure S2). Induction of ectopic c-MYC for up to 72 h led to a significant and time-dependent increase in the fraction of γH2AX-positive cells irrespective of their *AP4* and *p53* status (Figure 4A). Furthermore, both basal and c-MYC-induced levels of DNA damage were elevated in *AP4*- and/or *p53*-deficient cells when compared with *AP4/p53* wild-type cells. These results were corroborated by comet assays (Figure 4B and Figure S3), which also showed that both spontaneous and c-MYC-induced DNA damage was strongly elevated in *AP4/p53*-deficient compared to *AP4/p53* wild-type cells. In addition, a dramatic increase in micronuclei was detected in *AP4-* and *AP4/p53* double-deficient cells after induction of c-MYC, which could not be observed in *AP4/p53* wild-type or *p53*-deficient cells (Figure 4C and Figure S4). Micronuclei result from the missegregation of chromosomes during mitosis. Since *AP4-* and *AP4/p53* double-deficient cells accumulate high levels of DNA damage after c-MYC activation, they may enter mitosis with unrepaired DNA damage, causing the missegregation of chromosomes.

Furthermore, the number of bi-nucleated cells was highly elevated in *AP4-* and *AP4/p53*-deficient cells when compared with *AP4/p53* wild-type cells (Figure 4D and Figure S5). *p53*-deficient cells also displayed an increase in the number of bi-nucleated cells, albeit to a lesser extent. Similar to micronuclei, bi-nucleated cells presumably result from unrepaired DNA damage in *AP4*-deficient cells, which leads to incomplete chromosome segregation and incomplete cytokinesis. These results show that while *AP4-* and *AP4/p53*-deficient cells accumulate high levels of either spontaneous or c-MYC-induced DNA damage, the decreased proliferation of *AP4*-deficient cells is completely reverted by additional inactivation of *p53*.

### 3.5. Characterization of AP4- and p53-Dependent Effects in the c-MYC-Regulated Transcriptome

**Figure 5:**
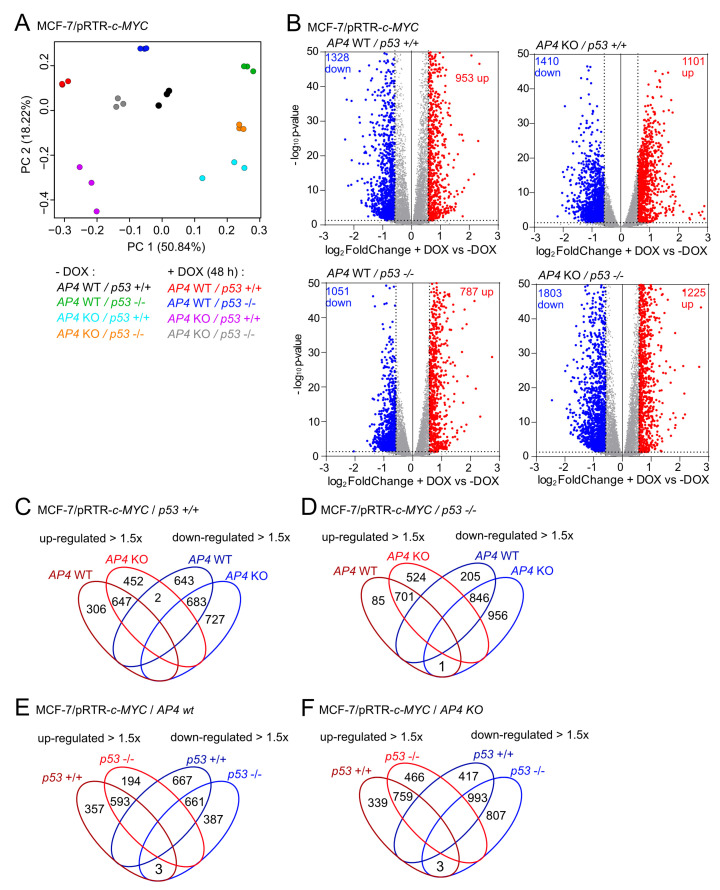
Transcriptional profiling of genotype-dependent and c-MYC-induced RNA expression changes in breast cancer cells. (**A**) Principal component analysis (PCA) of RNA expression in MCF-7/pRTR-*c-MYC* cells with the indicated genotypes and treatments. (**B**) Volcano plots showing differential RNA expression (fold changes > 1.5, FDR *q*-value < 0.05) between DOX-treated and untreated cells with the indicated genotypes. Significantly up- and down-regulated RNAs are highlighted as indicated. Non-significantly regulated genes are shown in gray. The numbers of differentially regulated RNAs are indicated. (**C**–**F**) Venn diagrams showing overlap between up- and down-regulated genes in MCF-7/pRTR-*c-MYC* with the indicated *AP4* and *p53* status after induction of c-*MYC* with DOX.

To determine the potential impact of AP4 and/or p53 on c-MYC-induced differential gene expression, we performed a comprehensive Next Generation Sequencing (NGS) analysis after the activation of ectopic c-MYC in the *AP4*- and/or *p53*-deficient MCF-7 cells characterized above. To do so, cells were pre-treated with ICI for 72 h in order to down-regulate endogenous c-MYC. Ectopic expression of c-MYC was induced by addition of DOX for 48 h in the presence of ICI. For each of the four genotypes, NGS libraries representing RNAs isolated from both DOX-treated (*n* = 3) and ICI-only (i.e., un-) treated cells (*n* = 3) were generated and subjected to RNA-Seq analysis with more than 30 million paired-end reads per library. Principal component analysis (PCA) showed that both untreated and DOX-treated *AP4/p53* wild-type, *AP4*- and/or *p53*-deficient MCF-7/pRTR-c-MYC cells were characterized by distinct transcriptomes (Figure 5A).

Of note, the majority of variation between DOX-treated and untreated cells was captured by principal component (PC) one in all genotypes, strongly indicating that c-MYC-induced gene expression changes were similar between the different genotypes for a large number of genes.

Differential gene expression analyses using DESeq2 showed that, in MCF-7/pRTR-*c-MYC* cells (*AP4/p53* wild-type), 953 genes were significantly up-regulated, and 1328 genes were down-regulated after treatment with DOX for 48 h (Figure 5B, Table S5). In *AP4*-deficient*/p53* wild-type MCF-7/pRTR-*c-MYC* cells, 1101 genes were significantly up-regulated, and 1410 genes were down-regulated after activation of *c-MYC* by DOX treatment (Figure 5B, Table S6). In *AP4*-proficient*/p53*-deficient MCF-7/pRTR-*c-MYC* cells, 787 genes were significantly up-regulated, and 1051 genes were down-regulated after DOX treatment (Figure 5B, Table S7). In *AP4/p53*-deficient MCF-7/pRTR-*c-MYC* cells, 1225 genes were significantly up-regulated, and 1803 genes were down-regulated after DOX treatment (Figure 5B, Table S8). Interestingly, the overlap between mRNAs differentially either up- or down-regulated (≥1.5× fold change) genes in *AP4*-deficient or *AP4* wild-type MCF-7/pRTR-*c-MYC* cells after treatment with DOX was substantial but not complete, irrespective of the *p53* status (Figure 5C,D). Likewise, the overlap between mRNAs differentially either up- or down-regulated (≥1.5× fold change) in *p53*-deficient or *p53* wild-type MCF-7/pRTR-*c-MYC* cells after treatment with DOX was substantial but not complete, irrespective of the *AP4* status (Figure 5E,F). This suggested that the regulation of molecular and cellular pathways by c-MYC while sharing commonalities, showed differences that were dependent on the *AP4* and/or *p53* status of the respective cells. However, using this approach, we observed very little or no overlap between genes showing strong opposing regulation (≥1.5× fold change up- or down-regulation) in *AP4* wild-type and *AP4* KO cells, neither in *p53* wild-type or KO background. Notably, one of the three genes displaying opposing regulation in *p53* wild-type versus *p53* KO cells irrespective of their *AP4* status (Figure 5E,F) was *CDKN1A*/p21, as shown by qPCR (Figure 1B), suggesting that its up-regulation after activation of *c-MYC* may be a critical factor for limiting the c-MYC-induced increase in proliferation in *p53* wild-type cells.

**Figure 6:**
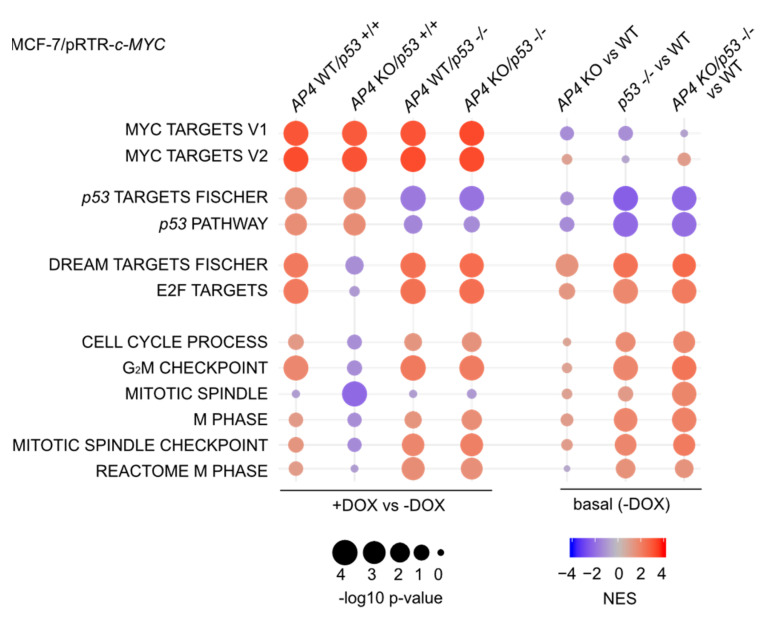
Loss of AP4 results in p53-dependent differences in c-MYC-induced RNA expression changes of genes involved in cell proliferation. Dot plot representation of gene set enrichment analyses (GSEA) of the indicated functional categories obtained from comparisons of DOX-treated (48 h DOX) vs. untreated samples of the indicated genotypes (left), as well as the comparison of basal (no DOX treatment) expression levels in the indicated genotypes vs. *AP4/p53* wild-type cells (WT). The significance of enrichments is presented by normalized enrichment scores (NES) and Benjamini-Hochberg-adjusted *p* values.

Next, we employed gene set enrichment analyses (GSEA) in order to identify molecular and cellular pathways which display differences in the regulation of their components in MCF-7/pRTR-*c-MYC* cells with divergent *AP4* and/or *p53* status after treatment with DOX (Figure 6). Direct c-MYC targets were activated after treatment with DOX, irrespective of the *AP4* or *p53* status. As expected, the activation of p53 targets after treatment with DOX was highly dependent on the presence of functional *p53*.

The repression of *p21* by AP4 is predicted to attenuate DREAM complex activity and alleviate the repression of its target genes. This suggested that the activation of AP4 by c-MYC may contribute to the abrogation of p53-mediated gene repression. Interestingly, an up-regulation of E2F- and DREAM-target gene signatures was observed in *AP4*/*p53* wild-type cells after treatment with DOX and was reversed in *AP4*-deficient/*p53* wild-type cells (Figure 6). Moreover, the differential regulation of E2F/DREAM target genes between *AP4* wild-type and KO cells was not observed when *p53* was inactivated, suggesting a direct involvement of p53. In addition, the activation of mRNAs belonging to functional categories largely comprising E2F/DREAM targets, such as gene sets representing processes involved in cell cycle progression (e.g., “G_2_/M checkpoint”, “Mitotic spindle”), were also abrogated in *AP4*-deficient cells in a p53-dependent manner. We thus hypothesized that loss of *AP4* may lead to enhanced repression of E2F target genes via hyper-activation of the DREAM complex.

Of note, a direct comparison of basal expression levels (i.e., MCF-7 cells not treated with DOX) indicated that the expression of E2F/DREAM targets, as well as the functional categories represented by these, was increased in *p53*-deficient cells when compared with *AP4/p53* wild-type cells, suggesting an inhibitory effect of wild-type *p53* on their expression (Figure 6). The elevated basal expression of genes involved in cell cycle progression in *p53*-deficient cells, irrespective of their *AP4* status, may explain the increased proliferation of these cells, as well as their lack of responsiveness to ectopic c-MYC. The basal expression of E2F/DREAM targets in *AP4*-deficient cells was increased compared to *AP4/p53* wild-type cells (Figure 6).

**Figure 7:**
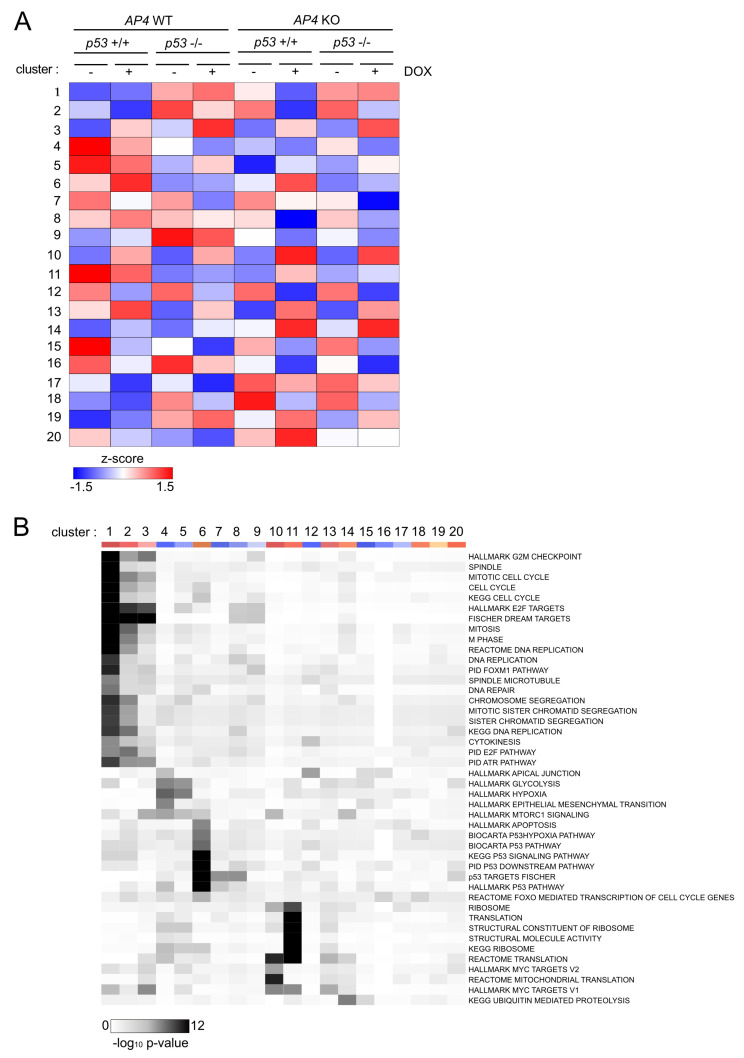
Clustering of gene expressions with genotype-dependent differences in c-MYC-mediated regulation. (**A**) Heat-map of RNA expression clusters comprising genes with statistically significant, genotype-dependent differences in regulation after induction of c-MYC. Clusters were determined using KMeans clustering. Cluster numbers are indicated on the left. (**B**) Heat-map of enrichment of functional categories in the transcriptional clusters as determined in (**A**). Cluster numbers are indicated on top. Statistical significance was determined by Fisher’s exact test.

**Figure 8:**
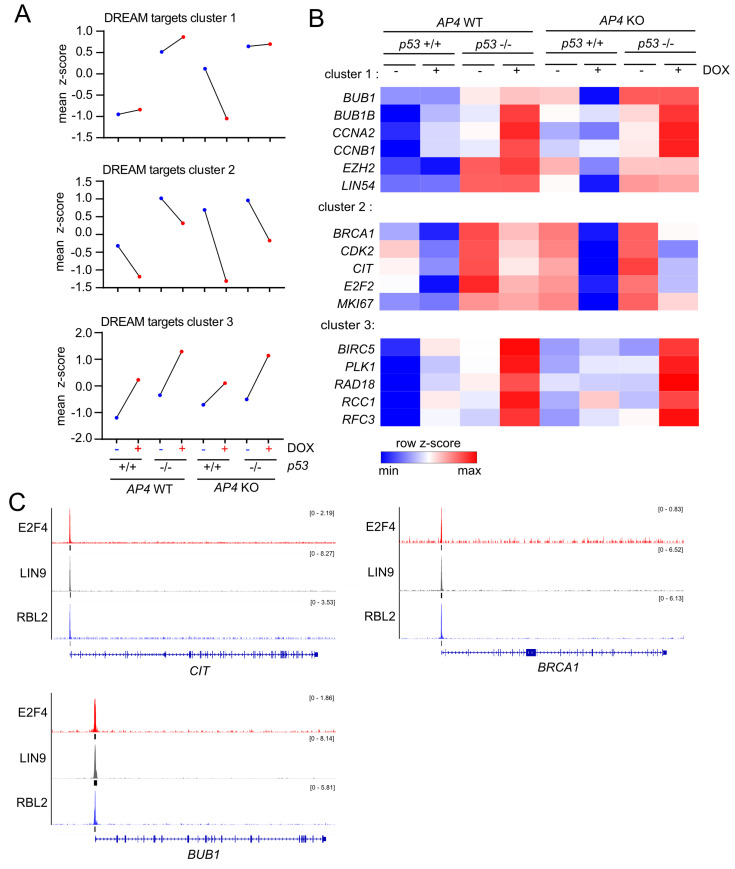
Expression clusters of DREAM targets with distinct genotype-dependent differences in c-MYC-mediated regulation. (**A**) Dot plot representation of normalized RNA expression of all DREAM targets with statistically significant, genotype-dependent differences in regulation after induction of c-MYC grouped in the indicated transcriptional clusters. (**B**) Heat-map of RNA expression of selected DREAM target genes with statistically significant, genotype-dependent differences in regulation after induction of c-MYC grouped in the indicated transcriptional clusters. (**C**) ChIP-Seq enrichment profiles for E2F4, LIN9, and RBL2 were obtained from ChIP-Atlas and generated with the Integrative Genomics Viewer (IGV). Black vertical bars below ChIP-Seq histograms indicate peaks called with MACS2 (*q*-value < 1 × 10^−5^). Gene structure ideograms are shown below the ChIP-seq tracks.

In order to identify genes with similar genotype-dependent differences in regulations after activation of c-MYC, we employed a two-factor (genotype and treatment) interaction analysis design using DESeq2, followed by KMeans clustering (*n* = 20) (Figure 7A). In total, we identified 2309 genes that displayed genotype-dependent differences in c-MYC-induced regulation (Figure S6, Table S9). Next, we determined which functional categories were significantly over-represented in at least one of the identified transcriptional clusters. Thereby, we identified a strong enrichment of E2F/DREAM targets, as well as pathways involved in cell cycle progression in the transcriptional clusters 1 and 2 (Figure 7B). Interestingly, the DREAM target genes in these clusters were characterized by elevated basal expression in *AP4*- and/or *p53*-deficient cells and p53-dependent down-regulation in *AP4*-deficient cells (Figure 8A), thus corroborating the findings of GSEA (Figure 6). A total of 202 E2F/DREAM targets were associated with clusters 1 (129/156) and 2 (73/155) (listed in Table S10). Of note, we also identified a subset of DREAM targets over-represented in cluster 3 (34/99), which does not display *p53*-dependent down-regulation in *AP4*-deficient cells but is induced upon activation of c-MYC. However, induction of the majority of these genes was attenuated in *AP4*-deficient cells, suggesting potential opposing regulation via the p53-21 axis also for these genes (Figure 8A). Representative examples of DREAM targets of clusters 1, 2, and 3 are shown in Figure 8B.

We verified the regulation of several exemplary, previously validated DREAM targets (*BUB1*, *CIT1*, and *BRCA1* [37,38]) by DREAM using publicly available ChIP-Seq data, which confirmed binding of the DREAM complex components E2F4, LIN9, and RBL2 in the promoter regions of the respective genes (Figure 8C). Notably, we also identified a subset of direct c-MYC targets displaying AP4-dependent differences in c-MYC-induced activation (cluster 10). These genes were characterized by stronger induction by c-MYC in *AP4*-deficient cells compared with *AP4* wild-type cells irrespective of *p53* status. This indicated that AP4 is not required for their activation by c-MYC but rather attenuates their induction by c-MYC. Whether their increased expression contributes to the decreased c-MYC-induced proliferation in *AP4*-deficient cells remains to be determined. Collectively, these results showed that the inactivation of *AP4* results in the down-regulation of numerous E2F/DREAM targets after activation of c-MYC in a p53-dependent manner.

### 3.6. Repression of DREAM Targets after c-MYC Activation Is Mediated by p21 and LIN37

**Figure 9:**
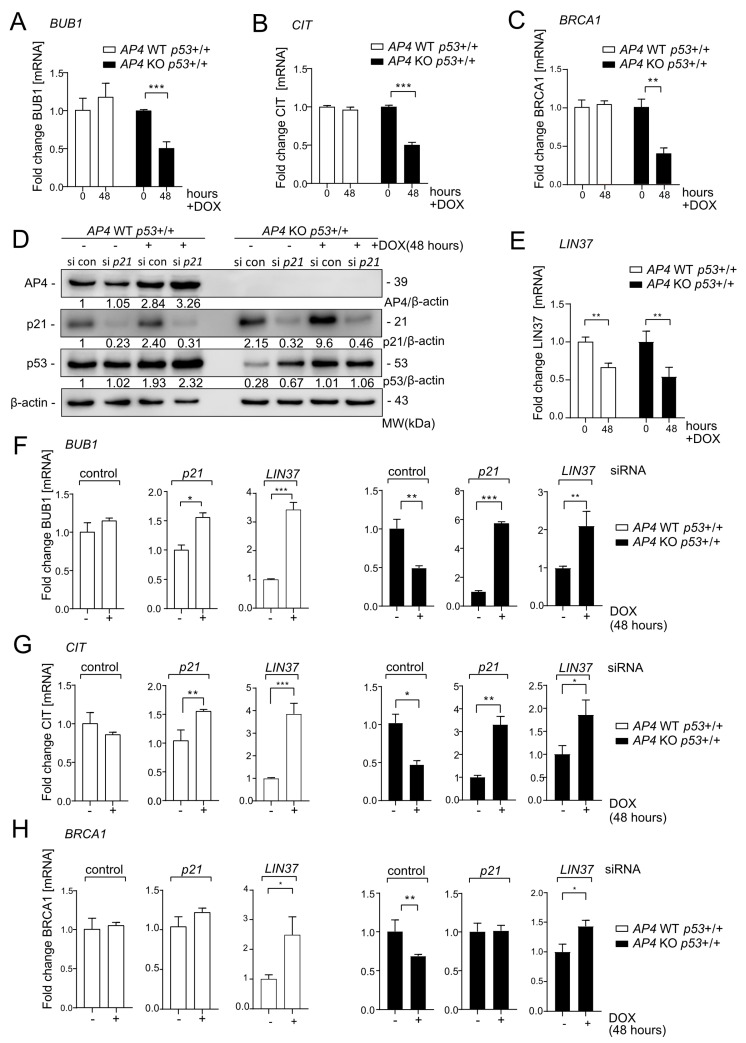
c-MYC-induced down-regulation of DREAM target genes in *AP4*-deficient breast cancer cells is dependent on p21 and LIN37. (**A**–**H**) Cells were pre-treated with ICI for 72 h before addition of DOX for 48 h. (**A**–**C**) qPCR analysis of *BUB1*, *CIT*, and *BRCA1* expression after induction of c-MYC in MCF7-pRTR-*c-MYC* cells. (**D**) Western blot analysis of AP4, p21, and p53 expression after *p21* siRNA (si *p21*) or control siRNA (si con) transfection and activation of c-MYC with DOX for 48 h. β-actin served as a loading control. € qPCR analysis of *LIN37* expression after induction of c-MYC in *p53* wild-type cells with the indicated *AP4* genotype. (**F**–**H**) qPCR analysis of *BUB1*, *CIT*, and *BRCA1* expression after induction of c-MYC in *p53* wild-type cells with the indicated *AP4* status after transfection with *p21*- or *LIN37*-specific siRNAs, or control siRNA. Cells were transfected with siRNAs immediately before addition of DOX. (**A**–**C**,**E**,**F**) Results are presented as mean +/− SD (*n* = 3) with *: *p* < 0.05, **: *p* < 0.01, ***: *p* < 0.001. The uncropped blots are shown in Figure S1.

Next, we verified the c-MYC-induced downregulation of the DREAM targets *BUB1*, *CIT1*, and *BRCA1* [37,38] in *AP4*-deficient/*p53* wild-type MCF-7 cells by qPCR (Figure 9A–C). Indeed, while expression of these genes remained rather unchanged in *AP4* wild-type cells, they were significantly repressed in *AP4*-deficient cells, thus confirming our NGS data. Therefore, we analyzed whether siRNA-mediated down-regulation of either p21 or the DREAM component LIN37 [39] (Figure S7) may revert the effect of loss of *AP4* on DREAM target gene repression. Of note, while p21 was induced after activation of c-MYC, *LIN37* was repressed after activation of c-MYC in MCF-7 cells (Figure 9D,E), suggesting that their siRNA-mediated down-regulation may have divergent effects. Interestingly, the three analyzed genes (*BUB1*, *CIT*, and *BRCA1*) where induced by c-MYC activation after RNAi-mediated inactivation of either *p21* or *LIN37* in *AP4* wild-type cells (Figure 9F–H). However, after activation of c-MYC in *AP4*-deficient cells repression of these genes was observed which was either converted into an induction (*BUB1, CIT*) or abrogated (*BRCA1*) upon RNAi-mediated inactivation of *p21* or *LIN37* (Figure 9F–H). While the effect of RNAi-mediated inactivation of *LIN37* was stronger than that of *p21* in *AP4* wild-type cells, it was weaker in *AP4*-deficient cells, which may in part be explained by the different regulation of p21 and *LIN37* after activation of c-MYC (Figure 9D,E): the effect of p21 knockdown may be more pronounced in *AP4*-deficient cells due to elevated p21 levels in these cells compared to *AP4* wild-type cells, whereas the levels of LIN37 are presumably already rather low after activation of c-MYC. In summary, these results validated that c-MYC–induced regulation of DREAM target genes is modulated by the opposing effects of AP4 and p53 on the p21-DREAM axis. These context-dependent, differential gene regulations down-stream of c-MYC are likely to have important consequences for the cellular outcome of c-MYC activation.

### 3.7. Association of c-MYC and AP4 Expression with p21, DREAM Targets and Patient Survival Is Dependent on p53 Status

**Figure 10:**
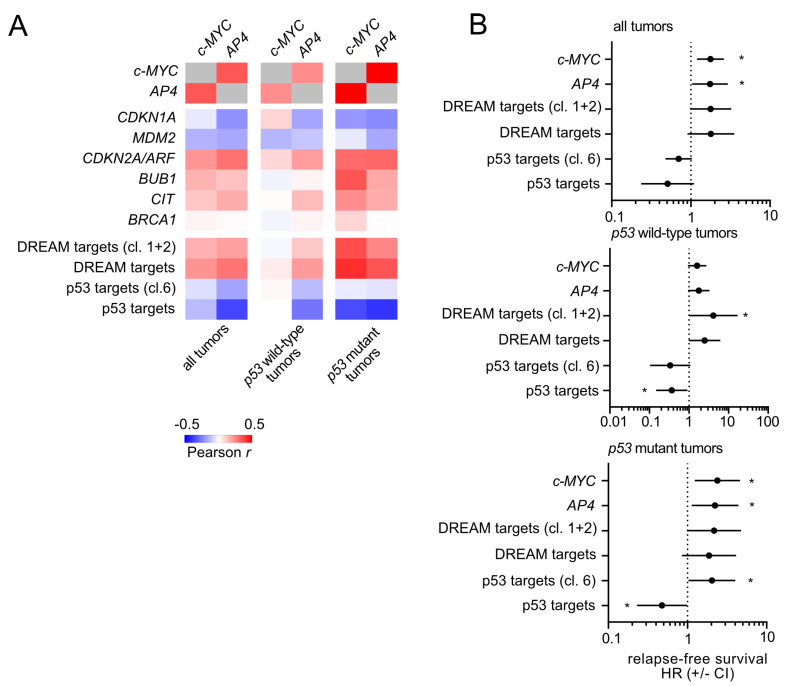
Conservation of c-MYC/AP4/p53/p21/DREAM targets correlation and clinico-pathological associations in primary breast cancer. (**A**) Expression correlations (Pearson *r*) of *c-MYC* and *AP4* with *p21/CDKN1A*, DREAM target and p53 target gene signatures were analyzed using public expression data (TCGA-BRCA). (**B**) Association analysis of the indicated factors and gene signatures with patient survival with regard to *p53* status using public expression data (TCGA-BRCA). Asterisks indicate statistically significant associations (*p* < 0.05).

Next, we analyzed whether the regulations identified above are conserved in primary breast carcinomas. For this, we analyzed RNA expression data from primary breast carcinomas and their associated clinico-pathological characteristics deposited in the TCGA database [35]. Expression of *c-MYC* and *AP4* showed a positive correlation (Figure 10A), as shown previously by us for CRC [24,40]. Interestingly, expression of *c-MYC* displayed a positively correlation with *p21/CDKN1A* expression. In line with our observations, this was only evident in tumors with wild-type *p53* but not those with mutant *p53*.

Moreover, the expression of DREAM targets was associated with c-MYC to a lesser extent in *p53* wild-type tumors when compared with *p53* mutant tumors. Conversely, a negative association of *c-MYC* with p53 target expression could not be observed in *p53* wild-type but only in *p53* mutant tumors. Taken together, these strongly imply that in *p53* wild-type tumors, high c-MYC levels may induce p53 and p21, which counteracts the c-MYC-induced activation of E2F/DREAM target expression.

Moreover, a high expression of *c-MYC* or *AP4* was associated with shortened relapse-free survival and an increased hazard ratio (Figure 10B). Interestingly, this association was only significant in *p53* mutant tumors but not in *p53* wild-type tumors. Hence, the presence of wild-type p53 appears to suppress the detrimental effects of high c-MYC/AP4 levels in breast carcinomas, possibly via activation of p21 and consequently, DREAM-mediated repression of cell cycle regulatory genes.

## 4. Discussion

Deregulated c-MYC expression has been shown to activate the p53 tumor suppressor, either by activation of p14/ARF or the induction of DNA damage due to unscheduled DNA replication. We have previously shown that the c-MYC-induced transcription factor AP4 regulates several pro-tumorigenic processes, including cell proliferation, EMT, and stemness, and suppresses DNA damage and senescence [14,36,40,41]. In addition, AP4 represses several p53 direct target genes, such as *MDM2* or *p21/CDKN1A* [12,15,16]. However, whether the c-MYC-mediated induction of *AP4* might play a role in inhibiting the tumor-suppressive effects of p53 activation has not been comprehensively analyzed to date.

Here, we abrogated AP4 expression in the breast cancer cell line MCF-7 harboring an ectopic, inducible *c-MYC* allele previously generated by us [12] using a CRISPR/Cas9 approach. Ectopic expression of c-MYC activates p53 in these cells, which allowed us to employ this system to analyze the effects of *AP4* loss on c-MYC-mediated activation of p53 and on processes downstream of p53.

The E3-ligase MDM2 is a negative regulator of p53 protein levels [42,43]. Since c-MYC-induced levels of p53 protein were highly similar in *AP4* wild-type and *AP4*-deficient cells, we concluded that repression of *MDM2* by AP4 is unlikely to account for the activation of p53 after induction of c-MYC. In mouse embryo fibroblasts (MEFs) and murine models of lymphomagenesis, the up-regulation of p53 after activation of c-MYC has been shown to be caused by induction of p19^ARF^, as it inhibits Mdm2 and thus leads to stabilization of p53 [6,44]. The MCF-7 cell line used here harbors homozygous deletions of the *INK4A/ARF* locus [45,46]. Therefore, ARF expression cannot be detected in these cells and does not account for the up-regulation of p53 by c-MYC observed here. Hence, a more likely scenario in this context is that the induction of DNA damage due to DNA replication stress induced by c-MYC ultimately leads to activation of p53 [9,47].

Here, deletion of *AP4* resulted in increased spontaneous DNA damage, senescence and reduced proliferation. We have previously shown that *AP4* loss has similar effects in colorectal cell lines and mouse embryo fibroblasts [24,36,41]. Interestingly, additional deletion of *p53* in *AP4*-deficient MCF-7 cells fully reverted their proliferative defects, and furthermore rendered these *AP4/p53*-deficient cells insensitive towards ectopic c-MYC expression, at least with regard to proliferation. Furthermore, even though c-MYC activation in *AP4/p53*-deficient breast cancer cells resulted in a dramatic increase in DNA damage, it did not result in senescence. These results are different from our previous findings obtained with *p53*-mutant CRC cell lines, where deletion of *AP4* caused a significant decrease in cell proliferation due to the induction of senescence. Potentially, the difference may be due to the complete loss of p53 activity in MCF-7 cells versus the presence of a mutant p53 in the CRC cell lines studied before. Alternatively, cell-type specific differences in the role of AP4 may exist. How *AP4/p53*-deficient cells regain high proliferative capacity and suppress senescence, even in the presence of high levels of spontaneous and/or c-MYC-induced DNA damage, is currently not understood and remains to be elucidated. It is likely that deletion of *p53* allows cell cycle progression and proliferation in the presence of DNA damage, whereas in wild-type *p53* cells uncoordinated DNA replication in the absence of AP4 activates p53 and attenuates cell cycle progression. Interestingly, we found recently that AP4 enhances DNA repair by inducing MDC1 expression [24], which may contribute to the positive effect of AP4 on c-MYC-induced proliferation.

The antagonistic regulation of *p21* by AP4 and p53 predicted that inactivation of *AP4* in *p53*-proficient cells would result in enhanced repression of DREAM and E2F target genes via the p21-DREAM axis after p53 activation by c-MYC. We generated comprehensive profiles of c-MYC-induced changes in RNA expression to determine which molecular and cellular pathways were affected by loss of *AP4* and/or *p53*. Thereby, we determined that an important role of AP4 after activation of c-MYC in *p53*-proficient cells lies in the maintenance of E2F/DREAM target gene expression. Gene set enrichment analysis (GSEA) showed that while the DREAM and E2F target gene signatures were up-regulated in *AP4* wild-type cells upon activation of c-MYC, they were repressed in *AP4*-deficient cells. Furthermore, we identified three subsets of DREAM targets that displayed distinct regulatory patterns after activation of c-MYC that were significantly affected by loss of either *AP4* and/or *p53*. For DREAM targets showing a slight induction or repression after activation of c-MYC, deletion of *AP4* in *p53* wild-type cells caused significantly stronger repression (clusters 1, 2). For DREAM targets showing an induction after activation of c-MYC, deletion of *AP4* in *p53* wild-type cells caused significantly weaker induction (cluster 3). This effect of loss of *AP4* could be abrogated or reverted by additional deletion of *p53.* Collectively, these differences in regulation all contributed to the regulatory patterns observed in GSEA. For the selected DREAM targets *BUB1*, *CIT*, and *BRCA1*, the effect of *AP4* inactivation could be reverted by concomitant siRNA-mediated depletion of p21 or the DREAM complex component LIN37, providing strong evidence that the enhanced activity of the p21-DREAM axis in *AP4*-deficient cells is causally involved in the repression of these genes by p53 after c-MYC activation. We had previously observed that siRNA-mediated depletion of p21 reduces the number of senescent cells in *AP4*-deficient MEFs [36], strongly suggesting that the AP4-mediated inhibition of the p53-p21-DREAM axis may also be critical for the suppression of senescence.

The regulation of E2F activity by c-MYC is well established [48]. For example, c-MYC regulates E2F activity via direct transcriptional activation of G1 cyclins, such as Cyclin D1 and cyclin-dependent kinases (e.g., CDK4 [49]), as well as by directly inducing expression of E2F1 [50,51]. This interplay between c-MYC and E2F transcriptional activities is crucial for the control of cell-cycle progression. The results presented here strongly argue for a role of AP4 downstream of c-MYC in the regulation of E2F and DREAM activities via its repression of *p21*, which presumably contributes to the cell-cycle progression-enhancing effects of AP4 [12,41], as well as to the abrogation of p53 activity by c-MYC [11]. Moreover, the p53-dependent nature of the regulatory relationship between the c-MYC/AP4 axis and p21/DREAM-mediated gene repression was at least in part reflected by RNA expression correlations, as well as clinical associations in primary breast carcinomas. Since AP4 expression has been shown to be elevated in various types of cancer besides breast cancer and is associated with poor prognosis [52], this function of AP4 may also be relevant in other tumor entities.

## 5. Conclusions

Here we show that activation of p53 by c-MYC is largely driven by replication stress-induced DNA damage and not mediated by p14/ARF in MCF-7 breast cancer cells. After c-MYC activation, *AP4* was necessary to suppress DNA damage and senescence and thereby facilitates cell proliferation. In *AP4*-deficient cells, p53 mediates senescence and inhibits cell proliferation. Our results show that AP4 represents a pivotal factor required for the balancing of c-MYC, E2F, and p53 activities via repressing *p21* and thereby attenuating the activity of the repressive DREAM complex. This function of AP4 appears to be important for a coordinated induction of cell cycle progression by c-MYC and presumably contributes to c-MYC-driven tumorigenesis.

## Data Availability

RNA expression profiling data obtained in this study were deposited in the Gene Expression Omnibus website (accession no. GSE221265).

## Supplementary Materials

The following supporting information can be downloaded at: https://www.mdpi.com/article/10.3390/cancers15041162/s1. Figure S1: Original, uncropped Western Blot images. Figure S2: Characterization of AP4- and p53-deficiency on basal and c-MYC-induced DNA damage by γH2AX staining. Figure S3: Characterization of *AP4*- and *p53*-deficiency on basal and c-MYC-induced DNA damage by Comet assays. Figure S4: Basal and c-MYC-induced formation of micronuclei in AP4- and p53-deficient cells. Figure S5: Characterization of AP4- and p53-deficiency on basal and c-MYC-induced formation of bi-nucleated cells. Figure S6: Clustering of gene expressions with genotype-dependent differences in c-MYC-mediated regulation. Figure S7: Validation of siRNA-mediated depletion of *LIN37* and *p21* by qPCR. Table S1: Sequence information for guide RNAs used for *AP4* deletion. Table S2: Sequence information for guide *p53* used for *p53* deletion. Table S3: List of Antibodies used. Table S4: Oligonucleotides used for qPCR. Table S5: mRNAs significantly up- or downregulated (≥1.5× fold change) in MCF-7/pRTR-c-MYC cells (*AP4* wild-type/*p53* wild-type). Table S6: mRNAs significantly up- or downregulated (≥1.5× fold change) in MCF-7/pRTR-c-MYC cells (*AP4* KO/*p53* wild-type). Table S7: mRNAs significantly up- or downregulated (≥1.5× fold change) in MCF-7/pRTR-c-MYC cells (*AP4* wild-type/*p53*−/−). Table S8: mRNAs significantly up- or downregulated (≥1.5× fold change) in MCF-7/pRTR-c-MYC cells (*AP4* KO/*p53*−/−). Table S9: 2309 mRNAs with genotype-dependent differences in c-MYC-induced regulation. Table S10: E2F/DREAM targets associated with transcriptional clusters 1, 2 and 3.
