# Supplementary material for: c-MYC-Induced AP4 Attenuates DREAM-Mediated Repression by p53
Source: Cancers (Basel). 2023 Feb 11;15(4):1162. doi: 10.3390/cancers15041162 (PMC9954515; doi:10.3390/cancers15041162)
Supplement: Supplementary file 1 [file cancers-15-01162-s001.zip › cancers-2172178-supplementary.pdf]

Supplementary Materials

Supplementary Figures

Figure S1: Original, uncropped Western Blot images.

Figure S2: Characterization of the effects of *AP4*- and *p53*-deficiency on basal and c-MYC-induced DNA damage by γH2AX staining.

Figure S3: Characterization of the effects of *AP4*- and *p53*-deficiency on basal and c-MYC-induced DNA damage by Comet assays.

Figure S4: Basal and c-MYC-induced formation of micronuclei in *AP4*- and *p53*-deficient cells.

Figure S5: Characterization of the effects of *AP4*- and *p53*-deficiency on basal and c-MYC-induced formation of bi-nucleated cells.

Figure S6: Clustering of gene expressions with genotype-dependent differences in c-MYC-mediated regulation.

Figure S7: Validation of siRNA-mediated depletion of *LIN37* and *p21* by qPCR.

Supplementary Tables

Table S1: Sequence information for guide RNAs used for *AP4* deletion.

Table S2: Sequence information for guide *P53* used for *p53* deletion.

Table S3: List of Antibodies used.

Table S4: Oligonucleotides used for qPCR.

Table S5: mRNAs significantly up- or downregulated ($\geq$ 1.5x fold change) in MCF-7/pRTR-c-MYC cells (*AP4* wild-type/*p53* wild-type).

Table S6: mRNAs significantly up- or downregulated ($\geq$ 1.5x fold change) in MCF-7/pRTR-c-MYC cells (*AP4* KO/*p53* wild-type).

Table S7: mRNAs significantly up- or downregulated ($\geq$ 1.5x fold change) in MCF-7/pRTR-c-MYC cells (*AP4* wild-type/*p53* -/-).

Table S8: mRNAs significantly up- or downregulated ($\geq$ 1.5x fold change) in MCF-7/pRTR-c-MYC cells (*AP4* KO/*p53* -/-).

Table S9: 2309 mRNAs with genotype-dependent differences in c-MYC-induced regulation.

Table S10: E2F/DREAM targets associated with transcriptional clusters 1, 2 and 3.

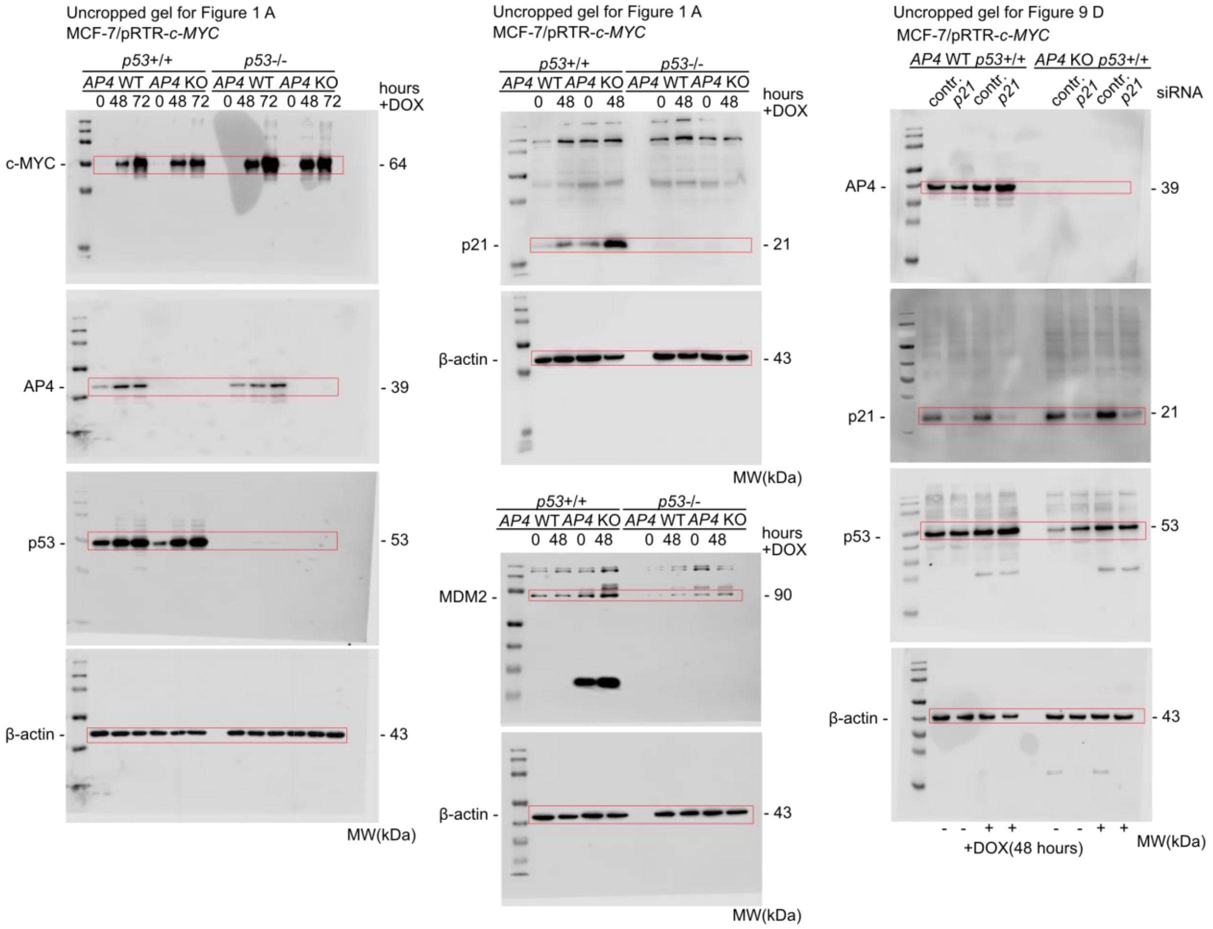

Figure S1: Original, uncropped Western Blot images.

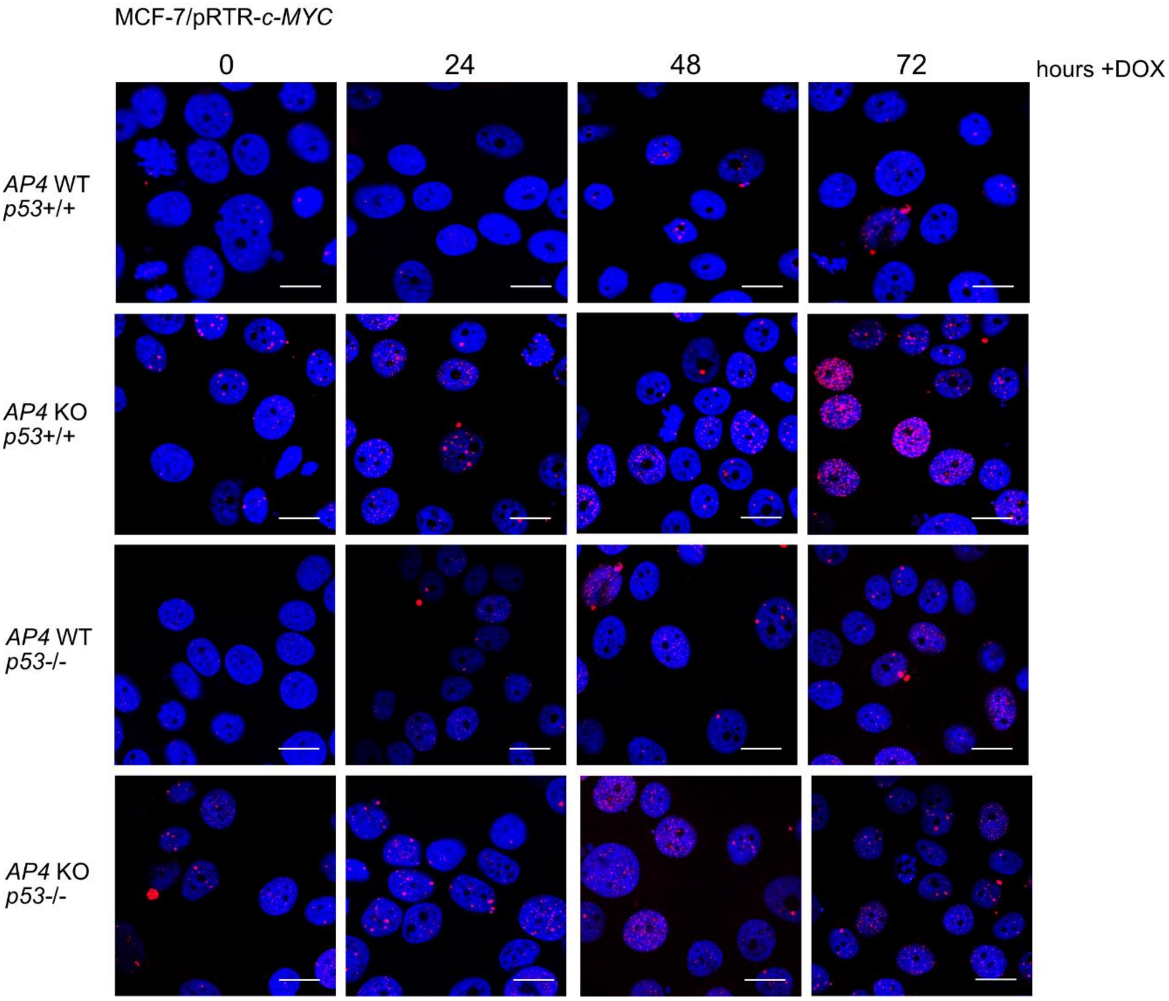

Figure S2: Characterization of the effects of *AP4*- and *p53*-deficiency on basal and c-MYC-induced DNA damage by γH2AX staining. Detection of spontaneous (no DOX treatment) and c-MYC-induced (24, 48 and 72 hours DOX treatment) DNA damage by staining of γH2AX foci in the indicated cell lines. Scale bars: 20 µm.

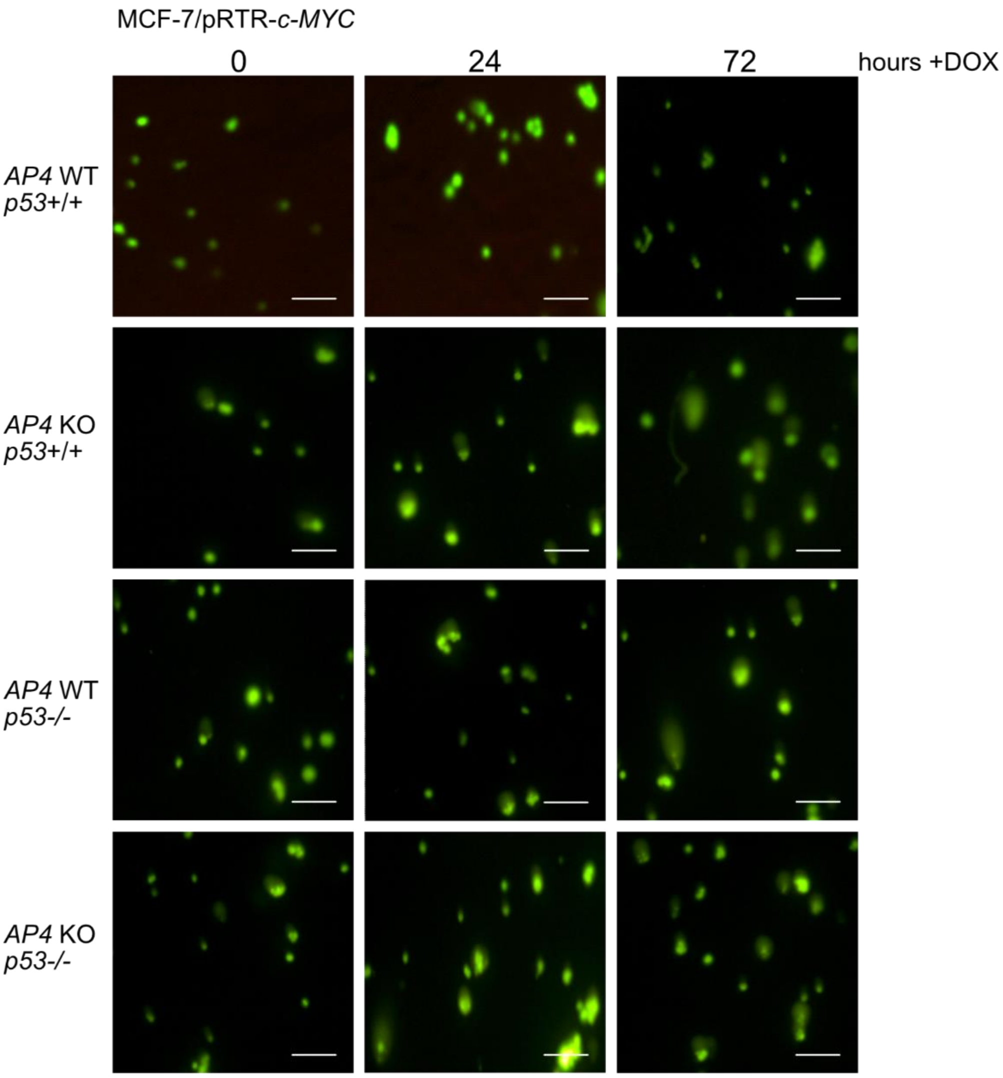

Figure S3: Characterization of the effects of *AP4*- and *p53*-deficiency on basal and c-MYC-induced DNA damage by Comet assays. Detection of spontaneous (no DOX treatment) and c-MYC-induced (24, 48 and 72 hours DOX treatment) DNA damage by Comet assays in the indicated cell lines. Scale bars: 10 µm

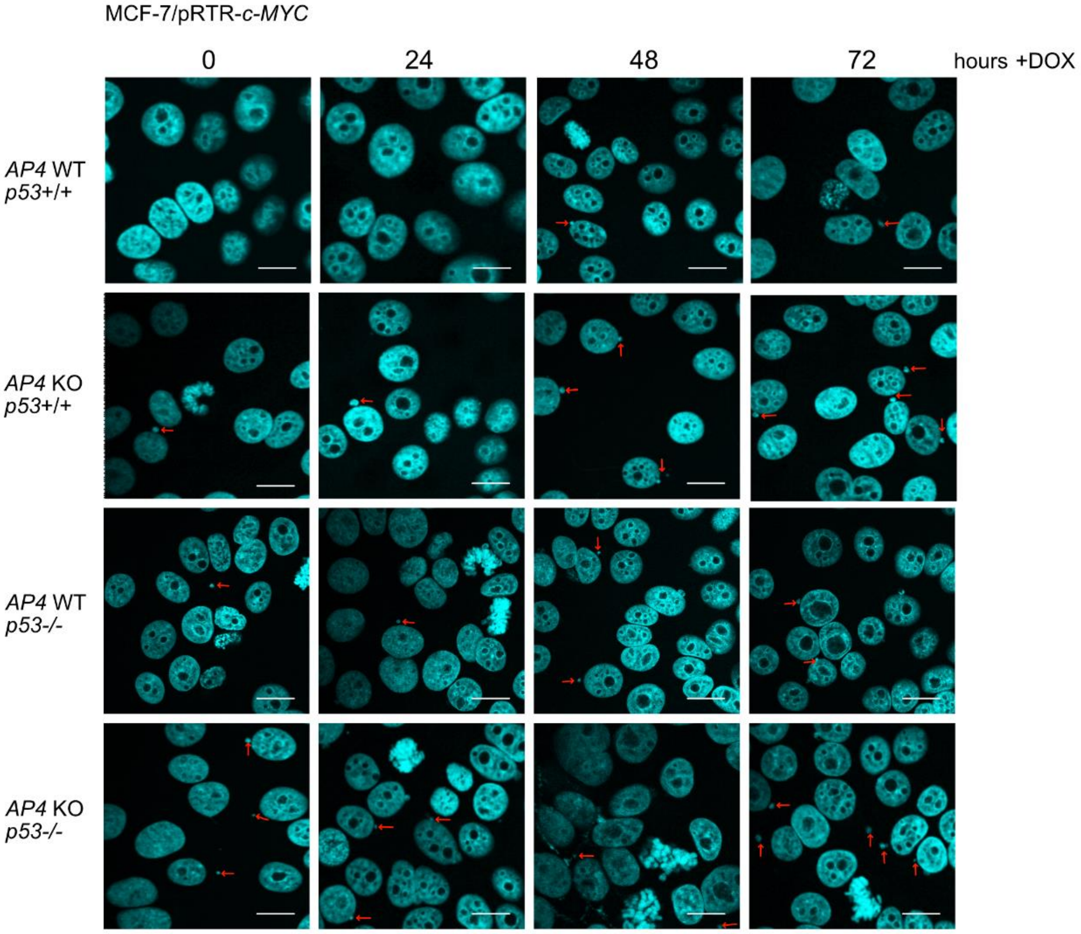

Figure S4: Basal and c-MYC-induced formation of micronuclei in *AP4*- and *p53*-deficient cells. Detection of micronuclei by DAPI staining after DOX induced c-MYC activation for the indicated time periods and cell lines. Arrows indicate micronuclei. Scale bars: 20 µm.

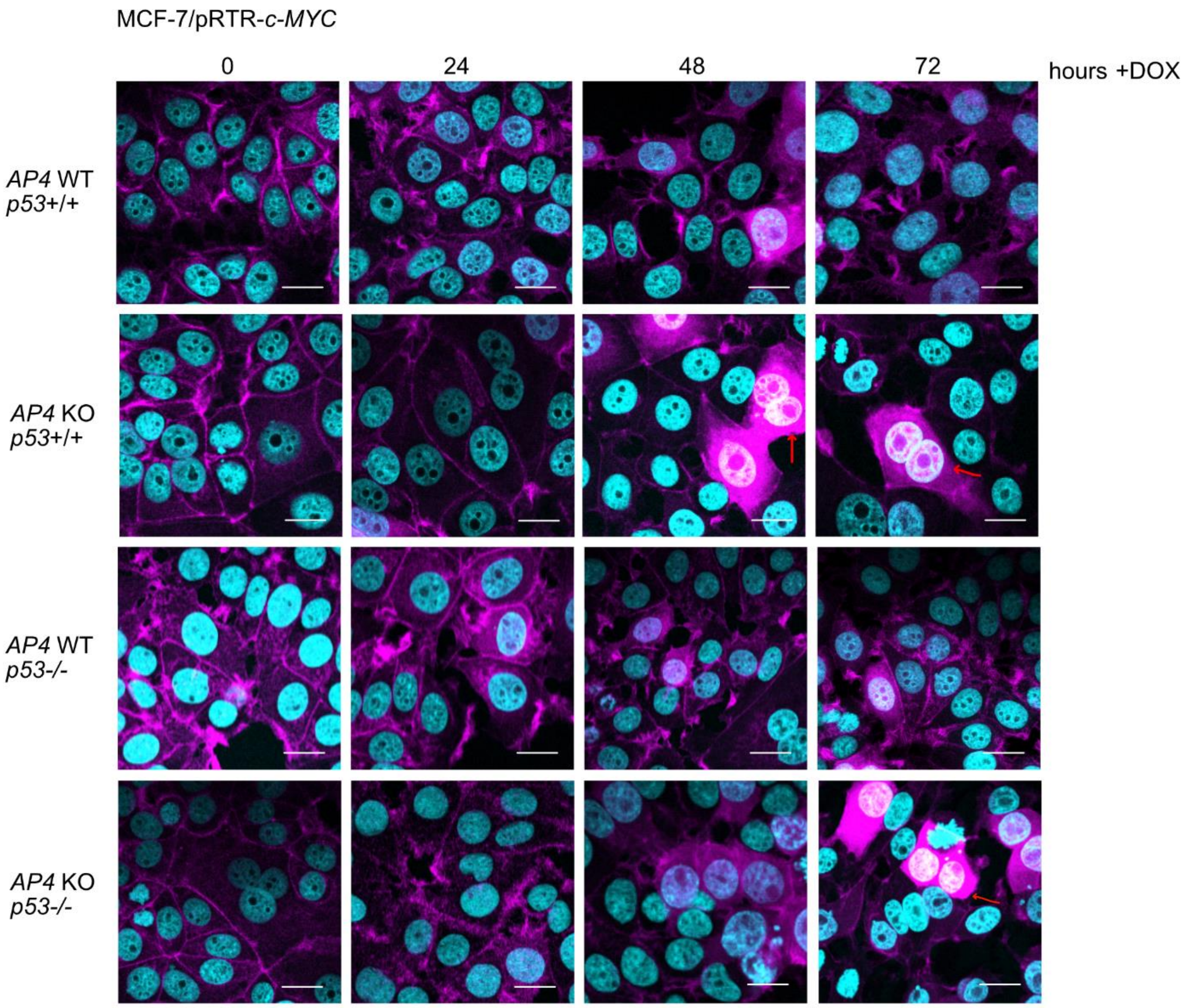

Figure S5: Characterization of the effects of *AP4*- and *p53*-deficiency on basal and c-MYC-induced formation of bi-nucleated cells. Detection of bi-nucleated cells after DOX induced c-MYC activation for the indicated time periods and cell lines. Scale bars: 20 µm.

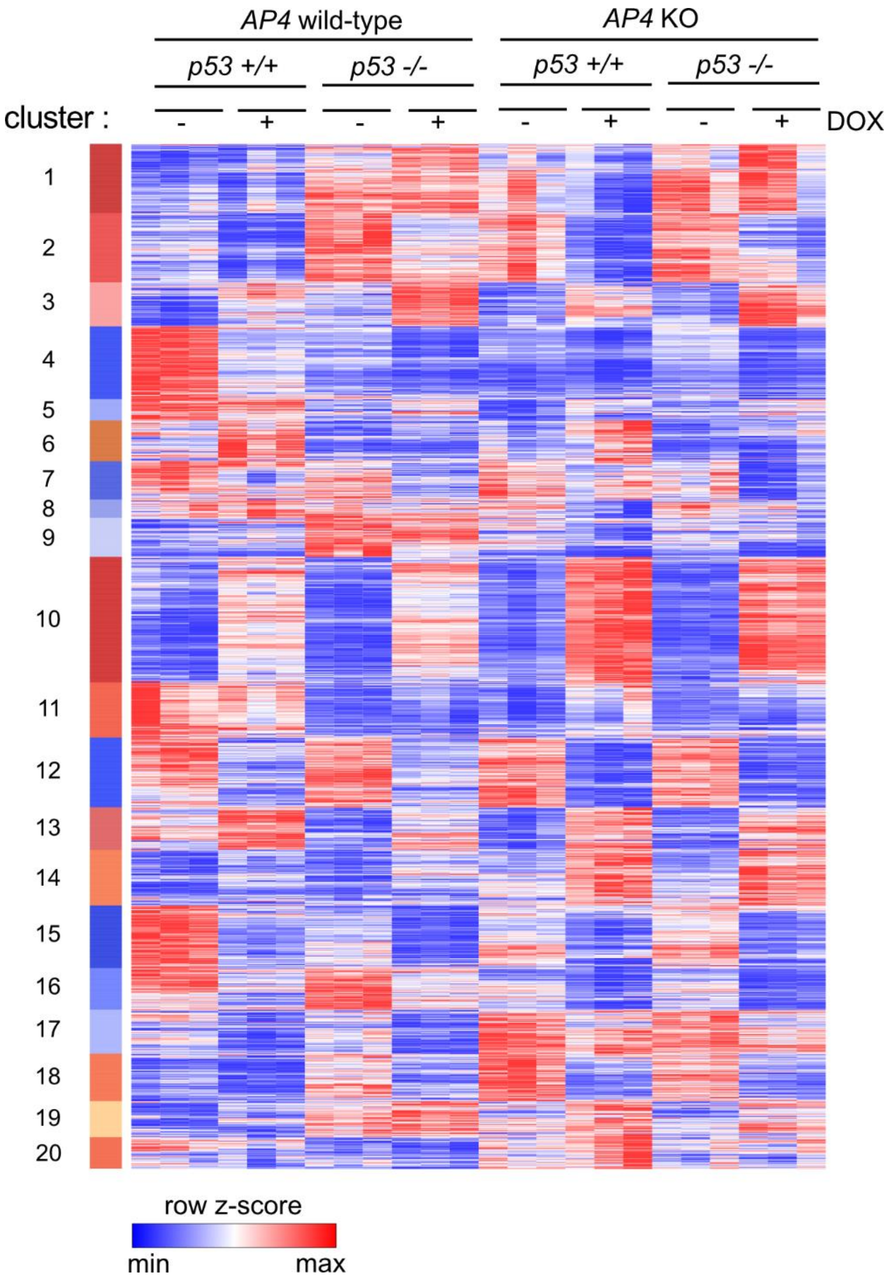

Figure S6: Clustering of gene expressions with genotype-dependent differences in c-MYC-mediated regulation. Heat-map of RNA expression of 2309 genes with statistically significant, genotype-dependent differences in regulation after induction of c-MYC. Transcriptional clusters were determined using KMeans clustering. Clusters are indicated on the left.

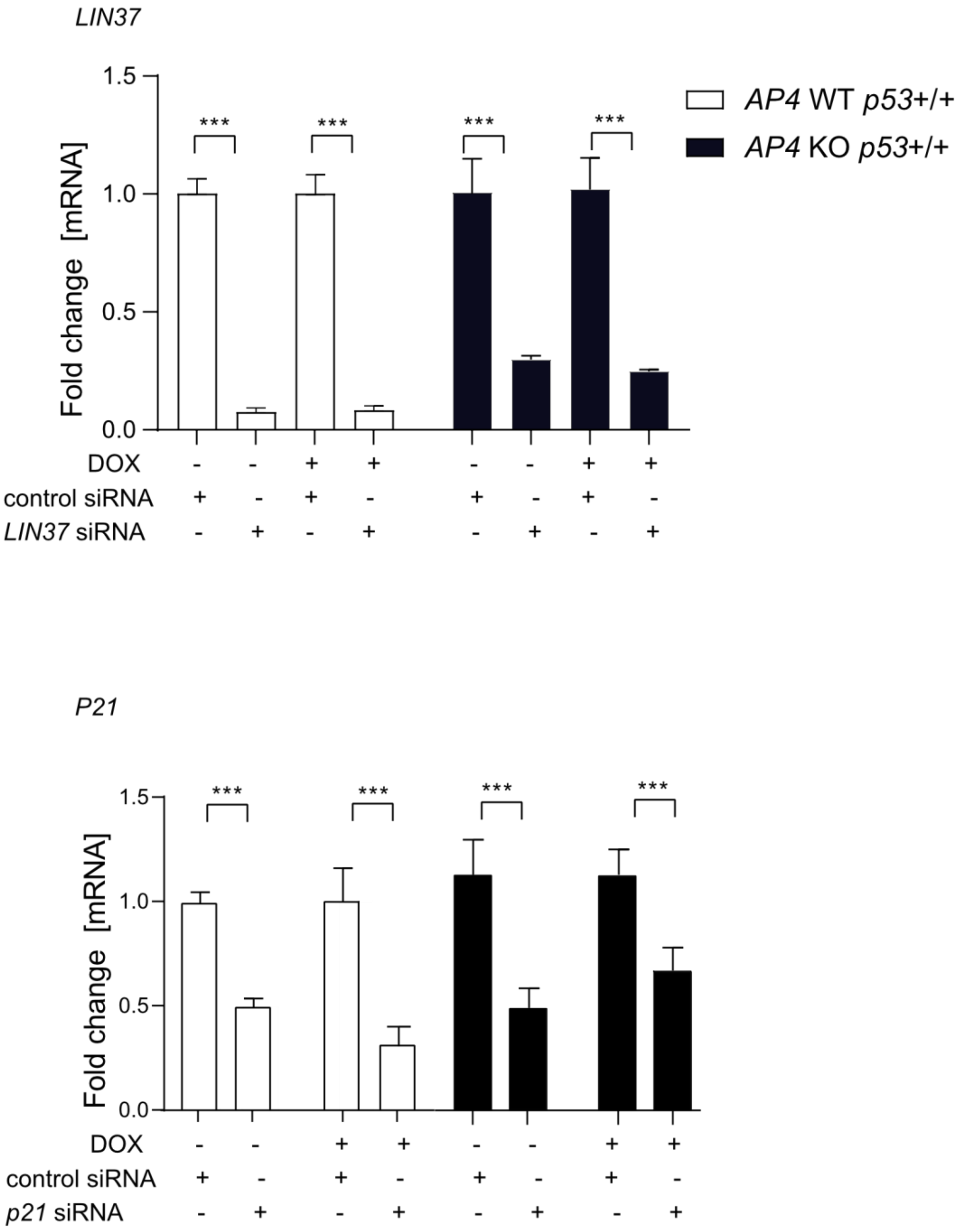

Figure S7: Validation of siRNA-mediated depletion of *LIN37* and *p21* by qPCR. qPCR analysis of *LIN37* and *p21* expression after induction of c-MYC in *p53* +/+ cells with the indicated *AP4* status after transfection with *LIN37*- or *p21*-specific siRNAs. Cells were pre-treated with ICI for 72 hours before siRNA transfection. DOX was added after transfection for 48 hours. Results are presented as mean +/- SD (*n* = 3) with ***: $p < 0.001$.

Table S1: Sequence information for guide RNAs used for *AP4* deletion.

	Sequence information (5'-3')
Guide RNA 1 Forward	CACCGACCAGGAGCGGCGGATTCGG
Guide RNA 1 Reverse	AAACCCGAATCCGCCGCTCCTGGTC
Guide RNA 2 Forward	CACCGGCGTCTCCGCTCGTTGCTGT
Guide RNA 2 Reverse	AAACACAGCAACGAGCGGAGACGCC
Guide RNA 3 Forward	CACCGCGCATGCAGAGCATCAACGC
Guide RNA 3 Reverse	AAACGCGTTGATGCTCTGCATGCGC

Table S2: Sequence information for guide *P53* used for *P53* deletion.

	Sequence information
sgRNA	3'-AUUUUGCCCTGTCGTAGTTTAGTAGG-5'
target	5'- ATATCGTCCGGGGACAGCATCAAATCATCCATTGCTTGG-3'

Table S3: List of Antibodies.

Epitope	Species	Catalog No.	Company	Use	Dilution	Source
Primary antibodies						
β-actin	Human	# A2066	Sigma-Aldrich	WB	1:1000	rabbit
TFAP4	Human	# MCA4993Z	AbD Serotec	WB	1:1000	mouse
P53	Human	#sc-126	Santa Cruz	WB	1:1000	mouse
c-MYC	Human	#06-340	Millipore	WB	1:1000	rabbit
γ-H2AX	Human	# JBW301	Sigma-Aldrich	IF	1:1000	mouse
MDM2	Human	# 86934	Cell Signaling Technology	WB	1:1000	rabbit
F-actin	N.A.	# A12379	Thermo Fisher	IF	1:50	Alexa Fluor® 488 conjugated
P21	Human	#2947	Cell Signaling Technology	WB	1:1000	rabbit
Secondary antibodies						
Anti-mouse IgG HRP	N.A.	# W4021	Promega	WB	1:10,000	goat
Anti-rabbit HRP	N.A.	# A0545	Sigma-Aldrich	WB	1:10,000	goat
anti-mouse IgG Alexa Fluor Plus 555	N.A.	# A32727	Thermo Fisher	IF	1:1000	goat
anti-mouse IgG Alexa Fluor Plus 488	N.A.	# A32731	Thermo Fisher	IF	1:1000	goat

Table S4: Oligonucleotides used for qPCR.

mRNA	Forward (5'-3')	Reverse (5'-3')
β-actin	TGACATTAAGGAGAAGCTGTGCTAC	GAGTTGAAGGTAGTTTCGTGGATG
p21	GGCGGCAGACCAGCATGACAGATT	GCAGGGGGCGGCCAGGGTAT
MDM2	TGCCAAGCTTCTCTGTGAAAG	TCCTTTTGATCACTCCCACC
BRCA1	CTGAAGACTGCTCAGGGCTATC	AGGGTAGCTGTTAGAAGGCTGG
CIT	TGGAAGGTGATGACCGTCTA	ACGTCCACAAGACACAGTGC
BUB1	GAGTGATATCTTCAGCTTGTG	AACAACCTGCTCAACATCAAC
LIN37	CACTGGCAAAAGGCCATC	GGTCGAACAGCTTGATCACAT

Table S5: mRNAs significantly up- or downregulated (≥ 1.5x fold change) MCF-7/pRTR-c-MYC cells (*AP4*/*p53* wild-type)

Significantly up-regulated mRNAs			Significantly down-regulated mRNAs		
Gene symbol	Log_2 fold change	padj	Gene symbol	Log_2 fold change	padj
MYC	3.872047865	0	MAN2B1	-0.584983277	1.65E-51
AC026786.1	3.515811968	1.82E-179	SFXN5	-0.585050857	1.53E-05
USP2-AS1	3.057708622	2.39E-47	TRPT1	-0.585320296	7.95E-14
PDE4A	2.709562237	5.52E-60	GTF2IRD2B	-0.585631561	4.56E-14
CPNE7	2.46735213	1.59E-59	SLCO4C1	-0.5857502	0.008287374
DUSP2	2.349669966	2.76E-47	GGT6	-0.585755058	1.40E-05
GAL	2.327390887	0	AC087591.1	-0.585993134	0.027013662
PLD6	2.31596678	8.06E-79	PPP1R3E	-0.586096835	0.000158393
KCNQ4	2.308870143	5.63E-25	FAAH2	-0.586412779	0.028685388
RAB3IL1	2.176964342	7.90E-53	LRTOMT	-0.586587815	0.00053131
AC007342.4	2.167684215	2.25E-15	ZNF230	-0.586732577	0.007735687
FABP5	2.158516911	0	RIBC2	-0.587264141	0.034820211
RNF125	2.072788382	1.03E-49	PPM1E	-0.587484214	0.00766181
RGS16	2.041455687	5.23E-89	EML2	-0.58810585	2.00E-24
POLR3G	1.994562072	1.04E-189	GOLGA7B	-0.588201433	1.86E-05
TMEM52	1.972954045	9.30E-14	LRRC27	-0.588261016	1.42E-05
AC040162.1	1.926291443	1.78E-54	ZNF503-AS2	-0.588334611	0.014399461
SORD	1.858242279	8.85E-238	PLEC	-0.588492011	4.70E-18
HOXC8	1.820335106	2.01E-11	SEPTIN9	-0.58894392	1.37E-106
TEX15	1.784018858	4.16E-25	PCED1A	-0.589027559	5.51E-13
RN7SL3	1.769358454	0.003107445	PXMP4	-0.589163563	2.13E-12
EFCAB10	1.752343011	2.95E-10	SDCBP	-0.589285439	3.42E-29
CAMKK1	1.745092885	8.04E-27	DOCK8-AS1	-0.589288274	0.04233111
ANKRD13B	1.739720715	3.35E-85	PLA2G6	-0.589797226	0.000410097
AMER1	1.73005578	2.32E-106	ATXN1	-0.589829626	6.80E-15
PPARGC1B	1.72703481	8.15E-69	AC006128.1	-0.589833853	0.000104049
LRFN1	1.656451375	7.06E-27	DTX3	-0.590095854	1.71E-08
BTG2	1.635713281	7.00E-138	CALR	-0.590153988	1.19E-114
HPDL	1.612742166	1.04E-143	CPNE2	-0.590321677	1.48E-07
SLC16A1	1.605820701	0	NBPF1	-0.590499715	2.18E-31
ADGRE2	1.604737118	3.51E-21	CTAGE4	-0.590548356	0.000367472

SORD2P	1.601021279	1.37E-09	IL4R	-0.59063869	8.57E-13
EN2	1.59338902	2.29E-65	CORO1A	-0.590931476	0.003469932
DNAH17-AS1	1.564745919	1.57E-10	PRLR	-0.590964736	7.43E-13
RPP25	1.539617804	6.58E-58	SH3BP5	-0.591016238	1.17E-86
SMIM11A	1.534161253	7.90E-07	DKK1	-0.591137753	4.75E-34
MICOS10-NBL1	1.523778304	5.70E-07	FZD7	-0.591266812	6.17E-05
TAF4B	1.522090831	9.99E-103	TMEM79	-0.591344131	6.43E-10
DBF4P1	1.518149231	5.61E-06	PIGP	-0.592054901	3.45E-06
ADAT2	1.51229078	4.94E-76	PALM	-0.592840713	1.59E-15
NFE2L3	1.511081631	5.01E-58	MPZL3	-0.592951275	4.10E-24
COQ8A	1.509246618	8.85E-110	SYT12	-0.592980465	9.14E-22
PODXL2	1.507121088	1.80E-85	INAFM1	-0.593139477	5.99E-05
TBC1D4	1.499130131	7.34E-114	ARHGEF10L	-0.593290068	1.99E-09
AC005224.3	1.485803325	6.25E-05	JDP2	-0.593494943	3.22E-12
UNC5B	1.480978125	6.87E-09	RNF32	-0.594098843	0.016228739
SLC29A1	1.477325903	1.40E-239	KATNAL1	-0.59484033	8.26E-05
MICAL2	1.471433661	1.71E-19	TPM1	-0.594846252	9.75E-66
EMSLR	1.466403633	3.94E-122	CTDSP2	-0.594953378	9.20E-36
ACTL8	1.46402373	2.39E-05	PGAP6	-0.595348982	2.64E-42
GRIN2C	1.449243863	2.55E-17	PLIN3	-0.597136446	6.59E-31
HRK	1.397695068	6.83E-06	PPP2R2A	-0.597156856	5.11E-72
MATK	1.383410773	2.87E-112	IPO5P1	-0.598449903	0.005696481
TERT	1.372827749	5.97E-05	CFAP44	-0.600049862	3.04E-06
METTL8	1.364043067	1.22E-108	DUSP1	-0.600319697	1.19E-12
KCTD12	1.363547867	1.29E-29	ATP1A1-AS1	-0.600360202	0.006494926
NPAS1	1.358410036	1.13E-05	AC016405.3	-0.601009285	0.024214329
PPM1J	1.355082212	1.10E-09	ACTR1B	-0.601020233	3.73E-38
NLN	1.346476765	5.84E-255	AC004233.2	-0.601052199	2.64E-05
MXI1	1.334248968	3.71E-127	AC020978.5	-0.601541623	6.72E-13
MIR17HG	1.333935956	4.70E-06	ATF6B	-0.601906025	9.82E-41
NRARP	1.333791617	2.99E-32	COL9A2	-0.601908125	0.000760398
FAM117B	1.331065618	8.79E-53	ELFN1	-0.602355202	9.90E-06
RRP9	1.321874764	3.07E-118	ARHGAP27P1-BPTFP1-KPNA2P3	-0.602578337	2.14E-09
SMKR1	1.3157789	7.70E-36	ACSS3	-0.602674655	7.99E-08
PITX1	1.311646704	2.46E-57	TTLL3	-0.603160234	5.78E-12
JAG2	1.305610791	6.72E-116	APLP1	-0.603657643	0.000106219
GPR63	1.303894453	3.15E-10	MSRB1	-0.604308787	1.56E-27
BCL11B	1.298261513	2.44E-21	LRRC24	-0.604768201	0.002474234
GEMIN5	1.29557054	7.08E-245	FGFR2	-0.604858723	7.12E-13
SLC16A10	1.294422832	5.13E-09	TMEM254-AS1	-0.604953524	0.038017552
SHISA9	1.289384605	1.69E-34	TLE2	-0.605195527	2.41E-07
LYAR	1.287686556	3.01E-170	SLX1B	-0.605499751	0.000143829
ODC1	1.287572951	5.41E-249	PPM1K	-0.606272425	0.000265365
CHN1	1.28458884	3.93E-67	MID1	-0.606463333	4.76E-05
AP006333.1	1.284449156	1.23E-08	ZNF555	-0.60870412	0.005528965

LINGO1	1.284319071	2.18E-12	CTNNAL1	-0.608998984	3.64E-31
VWCE	1.28069695	0.00028696	CYTH2	-0.609379789	9.22E-31
ARC	1.273548138	7.47E-05	SEMA7A	-0.609388139	9.15E-05
JPH1	1.272945754	1.63E-32	NUTM2D	-0.609754688	0.004941474
CD3EAP	1.272380885	5.21E-136	CROT	-0.609911073	1.88E-16
ZNF296	1.270265566	3.85E-18	SIPA1	-0.610098066	0.000383969
ACSM3	1.268702907	1.47E-05	ZNF688	-0.612081855	8.30E-06
NAT8L	1.267738244	1.60E-59	PCDH1	-0.612088972	3.13E-11
GNPDA1	1.267571126	4.15E-100	CSRP1	-0.612457927	2.28E-86
CRYM-AS1	1.257564981	0.000277728	AL441992.2	-0.613153518	0.024151459
RPP40	1.255747304	6.96E-59	CARD19	-0.613603693	3.51E-13
BMP7	1.247887327	3.35E-33	SPTSSB	-0.61391718	3.58E-121
PMAIP1	1.242039203	6.26E-15	MFAP2	-0.613953043	0.000528259
CCDC86	1.237803645	5.14E-164	KLF12	-0.613996268	2.27E-05
UBR5-AS1	1.233899488	2.67E-06	BPIFB2	-0.614011456	0.012959708
FAM81A	1.223504788	2.96E-15	MGLL	-0.614415022	0.015810501
IL27RA	1.221040933	2.38E-86	SPATA20	-0.61451353	9.81E-49
PFKM	1.208335466	4.17E-169	ZNF251	-0.614635976	2.15E-13
GASK1B	1.207688194	0.000129694	ERICD	-0.615518513	0.001408135
AC009831.1	1.204420216	1.34E-05	RAB5B	-0.615537493	4.27E-59
BIRC3	1.203833022	0.000138799	AL158212.3	-0.615727128	0.003687668
AP001931.2	1.203505577	0.000288897	SRRM2-AS1	-0.615855733	0.024732882
PPIF	1.20054244	1.16E-203	NFATC2	-0.615954632	4.15E-07
FJX1	1.198471288	1.41E-45	AL354707.1	-0.616036408	0.002407596
NPM3	1.198325111	3.51E-145	P4HA2	-0.6161501	9.20E-12
EEF1AKMT4	1.197320718	8.39E-94	SUMF1	-0.61657442	3.08E-35
TWNK	1.191724488	5.42E-94	ZNF362	-0.616649736	3.17E-10
TMC5	1.185772947	1.34E-10	FAM161B	-0.617098192	0.000797966
SLC7A11	1.183110275	1.52E-25	HSD11B1L	-0.617109408	0.043903615
ANK1	1.178653459	0.000555284	CBLN3	-0.617436566	0.032916446
GFOD1	1.177094787	4.50E-36	HHLA3	-0.617497124	0.007333039
ABCC4	1.172997051	6.55E-41	ZDHHC12	-0.617659357	3.24E-24
RPL23AP7	1.172800735	1.49E-11	PAQR4	-0.618128612	8.84E-20
PER1	1.167534811	3.78E-23	SLC28A1	-0.618256195	0.001443849
SLC25A19	1.165063989	1.17E-71	HEYL	-0.618578169	0.043700052
TYRO3	1.157948952	6.47E-44	ZNF837	-0.619603394	0.00271202
PDCD2L	1.157114994	3.78E-39	SHOX2	-0.619963218	0.028383832
H2BC11	1.156305869	2.75E-05	ZSCAN16-AS1	-0.620011486	0.045694888
CARMIL2	1.154769389	4.06E-14	B3GNT3	-0.620199434	1.38E-28
SACS	1.154554753	4.06E-82	HGSNAT	-0.620410464	1.46E-13
CFAP157	1.149226803	3.82E-07	SRSF5	-0.620748924	3.93E-29
DUSP7	1.148603932	7.08E-24	ARHGEF28	-0.621015021	0.00223981
MYBBP1A	1.148221621	3.23E-234	OSBPL5	-0.621136898	1.48E-06
CALML4	1.146066636	5.46E-05	STARD4	-0.621177848	1.64E-07
KCNK5	1.139064243	2.29E-08	STARD13	-0.621555603	0.000475472
SLC27A5	1.138422043	1.78E-25	KIF13B	-0.621949726	4.92E-10
IFIT2	1.13650842	2.55E-05	CDYL2	-0.62218084	1.11E-41

CENPV	1.135647828	7.30E-25	TAPBP	-0.622502651	8.78E-55
DUSP9	1.131244113	0.000173679	CALCOCO1	-0.622872658	1.01E-17
USP31	1.130956527	2.16E-54	FBXL2	-0.623046259	0.012242362
CMTM8	1.129734638	1.89E-11	TSPAN4	-0.6231285	1.70E-13
GRB14	1.128627711	3.72E-20	TMEM256	-0.623181926	2.90E-05
ADAM11	1.124707415	2.20E-10	CUL9	-0.623612797	1.22E-19
RPIA	1.121614956	7.01E-133	YIPF1	-0.62403277	2.01E-09
MPP6	1.118016826	8.72E-67	TINCR	-0.624065403	1.53E-20
RAB3A	1.113781769	1.01E-20	LDLR	-0.62416711	5.97E-59
RRP1B	1.104427254	2.00E-153	PROB1	-0.624309602	0.002226156
NANOS1	1.103767884	1.40E-11	AL031777.2	-0.624754617	0.026840736
SLC25A15	1.10178884	1.88E-100	ACBD4	-0.624902014	7.50E-07
DPH2	1.099782602	2.30E-101	PCDHA11	-0.625008733	1.18E-11
CHCHD4	1.098254418	2.07E-78	TBC1D3L	-0.625230115	8.76E-08
PSAT1	1.093959251	5.56E-107	AC139887.2	-0.625243189	0.023576873
BEX2	1.093595022	0.002080531	FUT8	-0.625674186	1.32E-48
TFB2M	1.091542221	3.30E-49	RHOC	-0.626262929	3.43E-83
SFXN4	1.090233151	1.73E-67	RNF215	-0.626892518	7.24E-10
FTL	1.088773124	7.03E-167	SMIM5	-0.627303326	0.00020343
RNF19B	1.087682921	5.94E-27	KIAA0825	-0.62751679	0.047897352
WDR3	1.086514827	2.98E-222	FCHSD1	-0.627551387	1.22E-10
FAM72C	1.085405923	0.005394203	KCNK15	-0.627638468	6.99E-98
HSPA4L	1.084040441	2.61E-124	OSCP1	-0.628119018	0.007923979
NDUFAF2	1.083044083	1.16E-76	LRRC20	-0.628728238	9.02E-05
FAM216A	1.082613395	8.56E-24	COL4A4	-0.629686055	0.011826325
MAP3K21	1.082387368	1.44E-31	MIDN	-0.630078941	1.58E-55
FXN	1.080967699	1.17E-36	MISP3	-0.630151981	2.94E-09
DIXDC1	1.080469935	5.77E-06	ARID1B	-0.631167268	2.10E-42
FAM78A	1.079643515	7.07E-14	UNC13D	-0.631431387	5.54E-58
TDRD1	1.077371996	3.52E-46	TGFBR1	-0.631770955	9.36E-54
GPRC5B	1.075055679	0.000385313	CELSR1	-0.632228592	4.38E-54
SYT7	1.071515442	4.27E-24	ECH1	-0.632263905	3.25E-45
POLR1B	1.065418625	3.85E-165	ZNF462	-0.633008807	2.71E-11
BEND3	1.061928212	1.37E-26	PACSIN1	-0.633719442	1.78E-09
CC2D2A	1.059969133	8.61E-21	PTPRG-AS1	-0.633740215	2.77E-06
DEPTOR	1.059824381	1.25E-13	APOBEC3B	-0.633777267	0.019432173
EPOP	1.05746042	2.24E-78	MFGE8	-0.634736289	1.18E-06
ABLIM1	1.053296758	3.03E-100	CD9	-0.634816215	6.71E-108
SLC27A4	1.053172207	9.80E-91	SBK1	-0.63499315	0.001543242
GALNT18	1.053067562	1.76E-58	FAM102A	-0.635457957	5.60E-47
MLKL	1.049508331	8.40E-63	SMAD3	-0.635765693	4.56E-70
TCOF1	1.048917801	3.56E-224	CYP4F11	-0.635934412	0.015343912
MNX1-AS1	1.038029207	8.76E-12	LMF2	-0.63699506	1.80E-31
RNASEH1-AS1	1.029719164	2.34E-17	LTBP3	-0.637165383	3.23E-72
MON1A	1.028526557	7.32E-30	MBOAT7	-0.638678304	1.96E-37
GPR135	1.02785834	4.55E-05	TNFAIP8	-0.639073088	1.47E-20
PRELID3A	1.025295254	4.86E-12	ANKRD37	-0.63940841	0.024717262

SNRPA1	1.022821255	9.36E-156	PRR15L	-0.63984558	9.70E-18
YRDC	1.021045793	6.18E-36	ZSWIM4	-0.640151093	1.94E-06
UTP20	1.019820635	3.64E-87	SERHL2	-0.640299839	0.016234616
TRAP1	1.018554184	0	FBP1	-0.640431652	5.23E-33
KBTBD6	1.017736679	3.48E-33	AC046134.2	-0.640557776	0.007537357
LYSMD2	1.016565895	8.37E-44	IQCJ-SCHIP1	-0.641008269	3.34E-07
NOTCH1	1.015241906	4.99E-92	SERPINB1	-0.641117151	1.65E-24
WNT10B	1.011715183	1.84E-17	RHBDF1	-0.641156886	1.32E-37
STOM	1.009605371	2.05E-155	PNPLA7	-0.641289696	0.001727551
ZNRF2P1	1.009093927	0.0002192	ALKBH7	-0.641456077	4.80E-14
LRP4	1.008596805	0.000309452	GPR39	-0.64152363	0.003627432
SLCO4A1	1.005775356	2.61E-34	PLEKHO2	-0.641722758	0.007244973
CCDC85B	1.004813964	6.04E-98	DYRK1B	-0.641896776	8.63E-16
SNHG30	1.003703146	1.84E-15	CES3	-0.641913237	0.041096749
ATP6V1C2	1.001846894	5.37E-05	S100A13	-0.642131126	5.04E-45
KCNG1	1.001106428	6.69E-18	ST3GAL5	-0.642314399	0.001818982
PRR5	1.001046376	1.33E-45	SLC38A2	-0.642423004	2.89E-107
RABEPK	1.000888694	2.03E-53	C6orf226	-0.643404023	0.002807995
CTU1	0.999969576	3.53E-17	SEPTIN10	-0.643474833	1.19E-08
DNAJC2	0.997571222	1.44E-222	RAB6B	-0.643827641	1.38E-07
NFIX	0.99436367	4.62E-19	C21orf58	-0.644143908	3.25E-06
INTS13	0.992674815	5.50E-79	NR4A1	-0.644190149	3.63E-15
NKD2	0.991105452	6.19E-09	CRYL1	-0.644632966	5.33E-14
AC118553.2	0.990479083	1.33E-06	FAM71E1	-0.644722698	0.027503754
RPL23AP82	0.989884671	2.02E-27	ARHGEF38	-0.644834894	0.002109888
GNB4	0.987679266	2.28E-07	LINC00886	-0.645055925	9.48E-05
TASOR2	0.98581924	1.53E-121	MICA	-0.645111658	1.93E-09
PCOLCE2	0.985676293	3.04E-29	DLC1	-0.645439339	0.016733829
LOXL3	0.985001467	2.28E-08	DNASE1L1	-0.646040667	1.14E-17
TRPM6	0.983860471	3.22E-07	AC068888.1	-0.646206326	8.22E-55
DGUOK-AS1	0.983581754	0.000919641	C1orf115	-0.646448222	2.21E-05
SUPV3L1	0.982980779	4.00E-69	FLNB	-0.646647451	2.87E-104
RUNDC3B	0.980360957	0.000363128	RWDD2A	-0.64730103	7.44E-08
PNP	0.980022184	7.27E-87	ADCK5	-0.647716641	9.13E-11
NTHL1	0.97954216	8.73E-31	AC106795.2	-0.647798074	0.012647658
ATP6V0E2-AS1	0.979281563	0.000380085	RNF39	-0.64841362	0.006547076
DDX10	0.976085861	1.68E-50	BACE1	-0.648483431	3.86E-51
SPRY1	0.975555113	2.28E-12	FMN1	-0.650017355	4.53E-07
TMC8	0.973822581	9.89E-05	FLNB-AS1	-0.650019133	0.036993363
REXO4	0.973452798	4.75E-126	ARFGAP3	-0.650906551	2.01E-15
TXLNG	0.972718249	2.26E-93	TTC9	-0.65095372	2.74E-21
SCML2	0.972177153	4.53E-10	PRSS8	-0.651261169	8.70E-17
FDXR	0.970198338	1.15E-73	ZNF112	-0.651556798	0.018042421
LDLRAD3	0.969999018	1.37E-18	ROCK1P1	-0.651587601	0.04579248
MARS2	0.968310258	4.38E-46	DNAJB2	-0.651656507	1.73E-22
SLC25A32	0.966889843	1.68E-75	SALL2	-0.651680449	0.029930005
ZNF778	0.966507929	6.62E-45	CSRP2	-0.651798129	0.019599733

CYP2J2	0.96605708	0.002475613	SSH3	-0.651904968	8.97E-62
GALNT14	0.964882958	1.03E-34	TUFT1	-0.652150427	2.37E-28
SLC25A33	0.961291263	2.36E-38	SLC24A1	-0.65262394	4.69E-12
EIF2B3	0.960504404	8.30E-35	TES	-0.652757931	2.52E-35
AC025423.4	0.960477128	0.000133253	RHOU	-0.653054424	2.90E-08
KLHL18	0.954691532	3.43E-47	GLDN	-0.653230774	0.012802693
KAZN	0.951865931	3.21E-30	FRG1HP	-0.654003543	0.011663971
ALDH1B1	0.949874919	1.26E-62	PXYLP1	-0.654454548	1.80E-07
AP002387.2	0.948069827	9.07E-09	CRELD1	-0.655306284	4.10E-11
SLC19A2	0.946502107	7.16E-28	LAMB1	-0.655394221	1.92E-80
AC018645.3	0.945699197	0.006936571	TMPRSS2	-0.656553792	1.97E-21
PSMG1	0.945332698	2.69E-85	C2orf72	-0.656861971	0.042715723
FMNL2	0.943974112	1.43E-27	SEPTIN1	-0.657226099	0.044390187
DDX21	0.941543332	0	CCNE2	-0.657497376	4.19E-11
CHAC1	0.940308562	0.00019894	XYLT1	-0.658111847	0.014724579
TBCE_2	0.940293208	3.49E-10	EXT1	-0.658304425	1.24E-07
DFFB	0.939398745	7.88E-11	AC015813.6	-0.658432865	4.55E-06
SMG1P2	0.938365154	1.95E-14	MAP3K14-AS1	-0.658466054	8.02E-06
PNO1	0.937390479	1.00E-74	VLDLR	-0.658780662	2.63E-08
PINX1_2	0.936968783	7.69E-22	STX5	-0.658978034	1.34E-24
TRMT61A	0.936584002	3.79E-52	NEAT1	-0.659359196	1.04E-35
NOLC1	0.935425416	6.64E-263	ABLIM3	-0.659404395	1.19E-13
DTX4	0.93398538	5.78E-18	CREB3L4	-0.659910965	3.10E-16
DIAPH2	0.932593689	1.31E-35	SRR	-0.660372073	1.93E-05
NDUFAF4	0.93088652	1.07E-37	GHR	-0.660503997	0.01015973
QSOX2	0.929965095	5.46E-73	LINC01547	-0.660570087	9.85E-07
TUBE1	0.929837831	2.26E-11	LMNTD2	-0.661144976	0.009465283
ICAM5	0.928832452	2.52E-05	ASIC3	-0.66135767	0.000132599
HSD11B2	0.928381756	0.005777468	ACOT4	-0.661359131	0.004738721
PRDM6	0.928353763	2.49E-05	RGS12	-0.661424642	6.52E-07
NOP16	0.928263049	3.53E-146	HCG4	-0.66151476	0.020019519
TOP1MT	0.927682123	8.12E-98	TJP3	-0.661894646	3.82E-19
AP000944.5	0.927435505	5.88E-05	LINC01138	-0.661940901	0.003667043
RAB29	0.925997458	8.74E-46	TRPS1	-0.662768356	2.97E-21
SIX1	0.924951202	3.16E-21	MAFF	-0.662828955	2.43E-06
SAPCD2	0.924076938	6.88E-191	CRIP2	-0.662874464	4.56E-57
ELL3	0.923841256	3.66E-13	GATA3-AS1	-0.664313415	0.000631656
ASPHD1	0.923618318	2.10E-16	FUCA1	-0.664897679	2.60E-17
UCK2	0.923178105	7.53E-88	MAN2B2	-0.66492612	9.38E-22
MRM3	0.921096731	3.89E-60	SPDEF	-0.665219967	2.93E-55
THUMPD2	0.919623318	8.64E-19	PLEKHB1	-0.665483088	2.20E-05
CDC42EP1	0.918173697	3.67E-71	IRF7	-0.665761519	0.000360488
TFR2	0.917858581	9.76E-07	LINC00294	-0.665779242	1.41E-23
TYW3	0.917849216	9.71E-44	IFT27	-0.666234121	7.57E-12
HMGA1	0.917785071	8.06E-251	SYCP2	-0.666706604	1.18E-17
RBM28	0.916764259	5.50E-90	CCDC15	-0.667346008	0.01611372

COQ3	0.916755854	3.67E-20	NAGK	-0.667394103	9.72E-27
IMP4	0.916193678	1.31E-169	TLCD5	-0.667526141	0.000308997
TFAP4	0.915867664	1.35E-38	ARHGAP29	-0.668628487	3.98E-21
ASNS	0.91317362	2.11E-108	APOL6	-0.668635638	6.43E-06
ASTN2	0.912033194	5.34E-08	SLC37A1	-0.668682933	6.01E-31
MAST1	0.91159449	0.000103275	KCTD11	-0.668683253	2.43E-13
PDSS1	0.910151896	2.74E-27	ANO6	-0.668742578	2.02E-60
AC025423.2	0.910091967	0.000106331	H6PD	-0.669130799	2.32E-18
GBX2	0.909832536	0.000481739	FMNL1	-0.669206204	3.03E-06
MAK16	0.908205338	1.28E-43	HMGCL	-0.669381745	1.72E-11
STK32C	0.906912857	1.28E-23	ARSJ	-0.669441635	4.00E-06
TANGO6	0.906705588	1.37E-28	DSC2	-0.669483176	5.82E-34
PDCD11	0.905587802	7.40E-124	ZNF596	-0.669617764	0.005773728
CTU2	0.905510799	4.14E-35	FA2H	-0.669669674	0.03700374
ZNF695	0.905112173	7.55E-06	BHLHE40	-0.669944271	1.60E-25
NUFIP1	0.904876429	5.61E-25	H1-0	-0.67000171	1.05E-83
GADD45A	0.901999334	6.84E-15	ACP2	-0.670447734	3.24E-12
FOSL1	0.900596505	9.00E-10	HLA-C	-0.670669144	2.38E-57
OAS1	0.899746305	0.010175716	KAT2B	-0.671730781	1.74E-19
PM20D2	0.898996793	1.59E-67	MIR22HG	-0.671968034	4.61E-14
GLS	0.898760572	7.94E-114	FADS2	-0.672088856	2.13E-08
PRKX	0.898132947	5.48E-68	IFI27L2	-0.672117555	9.79E-08
ARID5A	0.897808273	4.71E-11	GPR3	-0.672895012	0.016324255
RPUSD4	0.897756457	1.44E-37	TSTD1	-0.672933896	2.35E-18
BAG2	0.896977769	6.87E-18	SHB	-0.673018317	2.31E-47
GFM1	0.896683716	3.41E-120	RELL2	-0.673224292	2.92E-06
ASB13	0.896210774	4.06E-32	RAD51-AS1	-0.673253189	0.007977309
RIOX2	0.894926486	1.20E-83	PHLDA2	-0.673346636	2.41E-73
RITA1	0.894664476	1.95E-73	GRIN2D	-0.674079957	7.89E-05
SLC19A1	0.894045129	1.50E-64	PLD2	-0.674302599	7.05E-15
C1orf109	0.893836479	1.55E-27	NICN1	-0.674968682	0.000177903
TRMT11	0.893788776	3.83E-45	APAF1	-0.675374802	2.92E-09
ID2	0.891277069	6.85E-12	AP000769.1	-0.67591522	0.020166908
ME2	0.891069967	1.46E-36	AL450998.2	-0.676461614	0.003970559
PPID	0.889581181	1.92E-70	ALDH6A1	-0.676690578	1.42E-20
NCS1	0.888576922	5.02E-116	KRCC1	-0.677284402	1.55E-23
KREMEN2	0.887759005	1.11E-13	GPX8	-0.678013407	1.01E-15
HHEX	0.887666265	2.53E-05	ABCA5	-0.678115158	3.50E-14
LBHD1	0.8843661	6.79E-17	BFSP1	-0.678579337	2.07E-08
MRPS30	0.884278326	1.63E-70	ATP2B4	-0.678981859	0.023509188
SCO2	0.884277653	3.87E-21	GLTPD2	-0.679253177	0.010029415
HOMER2	0.884018711	2.30E-53	SYNPO	-0.679701045	0.01766189
CRACR2A	0.882506709	3.96E-13	RALY-AS1	-0.679796992	0.006067515
CCNG1	0.880867206	8.73E-67	FAM122C	-0.68004238	0.000504417
PDP2	0.880375075	1.60E-26	DUSP10	-0.680884935	0.023220743
ZNF485	0.879920486	4.58E-09	LRRC37A3	-0.6808978	3.58E-06
AC020763.4	0.878093708	1.33E-05	KCND3	-0.680937848	0.038867976

AL024508.1	0.877732333	5.39E-12	GATA3	-0.681654902	1.66E-145
RRP1	0.877692547	2.14E-89	PLEK2	-0.681768299	3.82E-09
SESN1	0.877571179	4.61E-28	FAM153CP	-0.682447016	1.04E-05
SKP2	0.876231531	6.60E-29	NAIPP2	-0.682708265	0.001334502
XPOT	0.875514166	0	THBS3	-0.682961295	8.48E-20
IMPDH1	0.874979461	2.06E-117	PAQR8	-0.683092297	0.009213688
FAM155B	0.874898541	5.89E-17	H2AC19	-0.683853746	1.23E-22
NEK5	0.874030534	0.006585292	HSPB11	-0.683898279	9.96E-06
ENTPD1	0.87359642	4.37E-18	NECTIN2	-0.6841307	6.79E-55
ACTR5	0.872897615	3.58E-17	AC012321.1	-0.684648955	0.002143127
DHODH	0.87191574	3.45E-35	ZBTB22	-0.685440629	4.21E-16
NR1D1	0.870919964	1.39E-10	ADM	-0.685748811	9.64E-17
MRPL50	0.869230579	5.88E-51	PDLIM7	-0.685811612	2.65E-42
WDR4	0.868304274	1.85E-28	CRACR2B	-0.685863584	4.03E-12
PUS7	0.867844878	3.48E-106	COPZ2	-0.685978071	8.99E-11
SINHCAF	0.867842592	1.00E-71	LRRC23	-0.686292559	0.001222698
ZBTB24	0.867476597	6.20E-31	PNPLA8	-0.686337031	7.18E-35
LRRC58	0.867456347	1.90E-143	PPFIA4	-0.686571621	0.004100339
GPM6B	0.867203932	0.001567436	SOX13	-0.68674507	4.96E-36
POP1	0.864437864	3.88E-55	PAK1	-0.686833619	0.042221183
CCDC112	0.863820721	1.16E-15	CNFN	-0.687271467	0.036778886
IL17D	0.862626405	1.25E-15	DCDC2	-0.687411455	1.41E-18
POLR1E	0.862109176	7.94E-34	ZNF862	-0.687633698	4.79E-10
CMSS1	0.862092221	6.74E-67	AC055811.4	-0.687711219	0.001245282
WDR43	0.861239624	2.30E-130	NFATC4	-0.687737308	0.002059371
RRP12	0.860313369	6.08E-119	ARPC4-TTLL3	-0.688064023	0.000521226
CEBPD	0.860152558	1.64E-12	EFNB3	-0.688769617	4.49E-05
DLEU1	0.859851763	2.72E-17	CYP2D7	-0.688921694	0.018522658
GTPBP10	0.858666452	7.37E-62	DPYSL2	-0.688992039	2.33E-16
PUS1	0.858376583	3.30E-62	AC093512.2	-0.689167915	0.00012287
ECSIT	0.857183567	1.03E-57	NPHP3	-0.689240327	3.84E-11
AL161772.1	0.856251932	1.92E-09	EPHX2	-0.689582283	0.016313709
ZC3H8	0.855858353	1.57E-28	OPTN	-0.689786603	9.58E-23
ADGRA3	0.855707152	6.90E-72	C2CD4C	-0.690736634	0.007600448
ZNF331	0.85471776	5.16E-09	DSCAM-AS1	-0.690806731	1.02E-14
TEAD4	0.854543864	3.07E-31	WBP1	-0.691291674	5.39E-32
IFRD1	0.854390428	3.00E-74	TRIM34	-0.691326583	0.000248113
JADE1	0.852994836	6.76E-35	YPEL5	-0.691627918	4.86E-29
HELB	0.852321974	3.20E-12	COL7A1	-0.691878026	0.001218925
FLVCR1	0.850686144	1.08E-17	NUDT18	-0.692049648	2.77E-05
E2F5	0.849979295	3.32E-39	USP51	-0.69374384	0.013653506
RUVBL1	0.847538743	7.81E-162	SYT10	-0.694871348	3.88E-19
HSPBAP1	0.847277829	4.43E-07	WDR78	-0.695357591	0.03980849
ZBTB2	0.846341749	1.13E-55	INPP5J	-0.695964356	0.040704297
PTDSS1	0.845891125	1.23E-126	TMEM229B	-0.696306342	4.21E-12
ARHGEF4	0.844694852	0.000364444	PLAAT3	-0.696492149	1.05E-08
XPO5	0.844498004	5.05E-161	AC008915.3	-0.696518279	0.034947742

SOX12	0.844194047	2.26E-67	NLGN2	-0.696689511	1.33E-28
SLC25A37	0.843848996	6.18E-47	FANK1	-0.696960523	2.30E-06
NOC3L	0.843559861	3.62E-51	KIAA0895L	-0.697032081	1.04E-18
POLR1C	0.84350198	1.83E-82	RNFT1	-0.697067768	1.19E-13
PLIN2	0.843294413	2.72E-05	CATSPERG	-0.697441004	0.039909759
SMAGP	0.842815704	1.02E-45	SLC4A3	-0.698035293	0.00016434
TNFAIP3	0.840411931	0.000245766	EDARADD	-0.69820891	0.002269728
GRPEL1	0.838811228	6.84E-80	RHOBTB1	-0.69844928	1.05E-10
LAMC3	0.837321359	0.012471305	VEGFC	-0.698828466	1.12E-12
WASF3	0.837181731	1.89E-21	UPK2	-0.699488779	7.36E-24
KLHL21	0.836907317	3.75E-47	ARSG	-0.699878134	6.58E-08
WNK2	0.836712944	3.36E-32	SDC4	-0.699955819	6.76E-106
TAP1	0.836555576	3.54E-31	AC007383.2	-0.700942306	0.025913248
ARMC6	0.836316805	6.38E-75	PXDN	-0.701143079	1.99E-94
FBXO45	0.835396367	6.69E-118	EFHC1	-0.70122847	9.86E-12
SLC39A14	0.834224616	3.18E-64	TEP1	-0.701373001	8.06E-19
MTHFD2	0.834084962	9.78E-158	THBS1	-0.701636497	4.97E-33
LYRM7	0.83405329	5.71E-21	SGMS1-AS1	-0.701998401	0.007917117
C12orf29	0.833943059	1.37E-32	GULP1	-0.702179937	3.95E-10
UBIAD1	0.833432692	1.59E-35	C4orf19	-0.702259697	3.74E-10
DHX33	0.831622659	1.26E-55	ACADS	-0.702536403	4.73E-06
EXOSC5	0.831616778	6.22E-50	KCNK15-AS1	-0.70258988	0.040918208
TIMM21	0.831529723	2.35E-36	TRIM16	-0.702731151	2.26E-44
LCP1	0.830747374	0.000128008	EPHA1	-0.703417132	1.22E-27
PACC1	0.8307071	3.54E-10	KLC3	-0.703787443	0.030660433
NOCT	0.82985841	1.19E-15	PCLO	-0.703964348	0.002478653
FP565260.3	0.829695354	1.03E-06	AC147651.1	-0.704028497	0.000436518
FIRRE	0.82950756	6.65E-13	ALS2CL	-0.704400695	2.41E-08
TIMM44	0.828818459	3.50E-48	TRIOBP	-0.705434188	7.59E-27
TRMT1	0.828736531	2.65E-73	MCC	-0.705515639	1.08E-08
GAN	0.828206695	7.24E-24	MARCHF2	-0.705539972	5.39E-07
FASTKD1	0.827063447	5.41E-31	APOLD1	-0.705808346	7.80E-05
GRPEL2	0.825950648	4.71E-29	AL138885.3	-0.705850838	0.032435804
TMEM33	0.824847829	6.45E-70	NRBP2	-0.706130304	1.69E-17
SLC16A1-AS1	0.82415519	0.000373531	PINK1	-0.706731779	8.56E-42
ZIC5	0.823386878	1.66E-09	KIF3C	-0.707341462	2.98E-12
FKBP5	0.823098275	3.69E-63	FEZ1	-0.707532982	0.002167805
THAP4	0.822861816	1.72E-72	CIRBP	-0.70874447	2.41E-98
AC002310.1	0.822737593	0.007250473	STAT2	-0.709040066	2.16E-17
FAM189B	0.821860654	8.51E-39	ERP27	-0.709050466	3.98E-05
HSPA12B	0.819876141	0.002487659	IL17RC	-0.709551165	5.21E-18
PRMT5	0.819851049	1.56E-109	KRT4	-0.710143901	0.009183874
SCLY	0.819418142	2.73E-36	SLC4A11	-0.710853465	0.00012704
NAGPA	0.817981144	7.22E-24	ENPP1	-0.711158451	1.09E-27
MRPS2	0.817839291	2.21E-104	MXRA8	-0.712316126	0.006376359
ENTPD1-AS1	0.817793404	3.63E-26	CHMP2A	-0.712688745	3.87E-43
ZNF239	0.817232593	3.56E-10	SAP30L-AS1	-0.712796559	0.048491739

UTP15	0.81617554	2.05E-49	ANXA2	-0.713208418	2.53E-176
DIMT1	0.816032412	1.79E-72	IL1R1	-0.713739894	1.27E-06
KBTBD8	0.815982882	1.14E-09	S100A4	-0.715367936	5.57E-16
WDR12	0.815643766	3.66E-53	ST3GAL4	-0.715783978	1.91E-88
CAMKMT	0.814742886	9.37E-08	AMTN	-0.715985671	0.004535811
FAM131C	0.814242863	0.000596071	CSGALNACT1	-0.716279203	9.22E-15
HEATR1	0.812806847	1.77E-60	JMJD7	-0.716326536	0.000899763
URB1	0.812415141	3.92E-106	ZBTB46	-0.716394053	1.52E-06
CCDC58	0.811739472	6.13E-32	NBEA	-0.716681741	1.00E-15
RBM19	0.811277215	1.02E-51	AC021092.2	-0.717073462	0.018756491
NIP7	0.8096937	1.05E-104	MAP3K12	-0.717271217	2.95E-13
BLMH	0.809427972	2.41E-49	VIPR1	-0.717395584	0.000190077
PUM3	0.808981864	1.15E-60	THNSL2	-0.71768029	3.77E-15
CFAP97	0.808962529	7.03E-65	KRT7-AS	-0.717926875	0.001506778
C15orf39	0.806792636	6.70E-38	NUCB2	-0.718016395	7.52E-33
GCFC2	0.806280769	2.42E-19	VPS37D	-0.718045552	0.00144156
SHANK3	0.802763369	8.32E-11	CAVIN1	-0.718134132	2.73E-14
COA7	0.802530963	9.56E-45	PLXNA3	-0.718676434	3.14E-31
TTLL12	0.801983367	1.90E-113	VASH1	-0.719197593	0.000132695
L3HYPDH	0.801380499	2.93E-20	LARP6	-0.719348502	0.000477864
AC125807.2	0.800132242	2.65E-13	BAD	-0.719372137	1.96E-27
FOXO3B	0.800008545	1.19E-06	SAMD9	-0.720025698	1.86E-11
DCUN1D5	0.798306494	8.55E-59	AC012513.3	-0.720057195	0.014610195
AC107871.1	0.797529212	2.86E-07	ERCC1	-0.720144947	2.31E-23
ZNF529	0.797460261	1.86E-05	SLC22A4	-0.720397721	0.008145777
ZNF283	0.795166135	1.14E-07	ETV5	-0.720693338	5.85E-31
ZMAT3	0.794944791	1.91E-75	MYLK-AS1	-0.720980047	0.005503011
PLCH1	0.794691474	6.92E-23	SYNM	-0.721833265	1.06E-05
TRIAP1	0.794618765	9.56E-55	HIPK2	-0.722635812	2.15E-18
NXPH4	0.794590054	4.79E-19	GDPD3	-0.722661974	1.74E-11
HOMER1	0.793875724	1.13E-45	CHRD	-0.723830627	5.43E-08
NOP56	0.793708084	1.75E-138	MALAT1	-0.724637977	1.51E-23
PFAS	0.793427081	8.04E-55	OSMR	-0.725241204	9.70E-35
RPUSD1	0.792876132	4.25E-66	AC069281.2	-0.725250702	1.15E-17
LRRC3	0.792782626	3.84E-05	CISH	-0.725668051	4.44E-06
YBX3	0.791231239	2.92E-162	SLC46A3	-0.727001042	4.57E-05
RP9P	0.789318132	1.18E-07	ASPH	-0.727314891	4.69E-35
NCL	0.788567407	1.40E-200	PSMG3-AS1	-0.72754312	2.28E-23
KIF9	0.788447195	2.97E-09	ZNF311	-0.727652977	0.024910334
PNPT1	0.786684655	2.79E-74	ERV3-1	-0.728168981	2.46E-12
PPAN	0.785747632	4.61E-58	LIN7A	-0.728181121	0.014170912
ESF1	0.785269649	6.09E-64	CLSTN3	-0.728448687	3.04E-15
TRIM47	0.785047103	1.72E-05	FAM174B	-0.729498903	1.58E-102
RIOK1	0.78460536	6.32E-49	ESPN	-0.729673147	2.81E-09
MDM2	0.783281392	6.65E-111	IGFBP5	-0.730038833	1.79E-32
FAM136A	0.783227532	1.19E-138	KIFAP3	-0.73011418	3.30E-07
CYCS	0.783105686	1.27E-145	CASP4	-0.730683344	2.66E-06

FASTKD3	0.78146085	9.89E-16
F12	0.781176902	1.45E-28
KANK1	0.781108675	4.22E-31
UBE3D	0.781007968	7.02E-06
SLC9B2	0.77919109	3.99E-15
PGAM5	0.778664928	1.01E-97
TRNP1	0.778113538	1.00E-07
AC012676.1	0.77738992	0.013715675
PFDN2	0.776818071	5.29E-67
FAM89A	0.776780981	3.78E-05
NAT10	0.776748205	1.45E-122
FAM53B	0.776471611	4.55E-52
CDC123	0.775806	2.11E-94
PWP1	0.775275031	2.07E-78
MCAT	0.775179496	1.19E-33
WDR75	0.774977293	2.69E-67
MDN1	0.773316661	4.51E-13
BOD1	0.773010229	7.58E-89
SIM2	0.771771553	6.67E-09
NLE1	0.769919359	1.09E-29
TPST2	0.769718868	1.55E-16
PALD1	0.769690924	4.81E-06
CEP83	0.769509695	4.03E-25
PHB	0.769374607	6.70E-190
EDN2	0.768660916	0.001831329
TAMM41	0.766631529	4.72E-23
ACER2	0.766370112	0.000899636
ZNF551	0.765872905	0.000346746
NFKBIB	0.76546458	1.65E-30
BOP1	0.76506499	2.98E-163
NOL10	0.764966631	2.98E-60
LRFN4	0.764826694	5.24E-61
GTF2H2	0.764145754	5.88E-53
ACAT1	0.764086375	4.94E-37
EXOSC4	0.763542062	8.18E-54
DNAH14	0.762905673	2.75E-06
HSPD1	0.762159725	0
ASCC3	0.761940744	5.34E-103
HDHD5	0.761163058	1.88E-62
CDKN1A	0.760820323	5.75E-120
ASS1	0.760691649	7.36E-62
REXO2	0.760208348	5.50E-27
SMAD9	0.760198108	0.0398121
WDR74	0.760021903	1.99E-67
MRPL36	0.759771013	9.28E-45
SETDB2	0.759722866	2.64E-09
TMCO6	0.759524181	1.31E-11

CAMK2N2	-0.731110271	7.88E-06
GLUL	-0.73119491	1.55E-24
HHAT	-0.731873138	6.09E-06
H2AC6	-0.732111565	1.64E-19
CD24	-0.732376184	1.64E-179
GLUD2	-0.733370815	0.024627306
PGAP3	-0.733475412	6.49E-10
SORBS3	-0.733845804	7.91E-20
AL390728.6	-0.733911875	0.003619856
SH3BGRL	-0.733974521	1.09E-41
ITGB1	-0.734603767	3.95E-172
KLF4	-0.73463493	1.07E-26
PPARA	-0.734836109	0.035762256
PRRT3	-0.734931448	8.63E-32
PRKACB	-0.734938591	2.61E-07
GATD3A	-0.734998792	0.014399461
HNMT	-0.736812259	0.010551418
TMEM107	-0.737703257	7.19E-07
NHLRC3	-0.737957781	1.37E-13
AC015712.6	-0.738486959	2.63E-10
NEU1	-0.739033637	8.51E-37
MAGED1	-0.739290987	4.02E-147
SGCB	-0.739501395	4.99E-16
AL132800.1	-0.739719489	0.005613353
ZNF117	-0.739925961	9.84E-14
RIN2	-0.740239338	6.94E-09
QSOX1	-0.740340485	2.90E-157
MYOF	-0.740658177	5.04E-198
PAQR6	-0.740814047	2.64E-07
ZC3H6	-0.741228281	2.06E-07
SQOR	-0.742003004	3.81E-17
TAPBPL	-0.742573122	1.40E-06
BCL3	-0.742578433	1.84E-16
ARL4C	-0.742589164	2.27E-06
AC112220.2	-0.743040592	1.05E-05
LRRC46	-0.743073771	0.036019376
CCDC159	-0.743290786	5.22E-07
CRABP2	-0.7437888	2.36E-165
AL390719.2	-0.743808903	0.045362886
SNN	-0.744277289	4.47E-12
MCOLN3	-0.744683596	0.001399548
DISP1	-0.744766657	0.00153228
TM7SF2	-0.745405602	5.82E-45
PRSS22	-0.745461591	3.44E-21
MC1R	-0.746546272	4.00E-22
MBOAT2	-0.746632229	4.87E-22
NMB	-0.746725744	4.25E-62

RCL1	0.757281467	3.74E-27	UPK3BL2	-0.747089365	0.004870413
MRTO4	0.756849884	1.29E-72	SLC2A10	-0.747558529	3.89E-24
IFI30	0.756550843	1.60E-71	UACA	-0.74821098	1.19E-39
BCL2L11	0.756184303	7.72E-20	MMP23B	-0.748222626	0.025048185
DHX37	0.755855558	2.71E-73	ZNF222	-0.748359698	0.025485687
PARVB	0.754740452	4.39E-07	RABAC1	-0.748488105	2.31E-32
NDC1	0.754259314	9.04E-59	FAT4	-0.748879187	2.21E-07
SRRD	0.754062963	6.15E-23	AC090114.2	-0.749138402	1.39E-11
RBM38	0.75401414	6.32E-56	F3	-0.749263445	2.38E-07
FOXN3	0.753641632	5.56E-20	FXYD3	-0.749828381	1.19E-68
DOHH	0.75344132	1.88E-15	LRG1	-0.749864088	0.008950721
ZNRF3	0.753143369	5.64E-15	ZNF821	-0.750557447	8.98E-08
FAM162A	0.752819197	1.10E-35	FRK	-0.75135437	9.82E-06
NOTCH2	0.752554831	2.70E-139	PLOD2	-0.751654968	2.14E-98
DYNC2H1	0.752230004	3.47E-25	KRT18	-0.75251403	2.30E-251
TMEM177	0.752172216	1.40E-11	TM4SF1	-0.752839837	0.025249387
USP18	0.751418616	1.41E-12	ATP2B1-AS1	-0.753153334	0.001130468
SNHG4	0.751203432	3.00E-25	AC253536.1	-0.753389369	4.88E-05
GEMIN4	0.750940183	7.11E-65	ADSS1	-0.753418059	4.52E-10
FAM210A	0.750804961	2.87E-33	OPLAH	-0.753467761	6.34E-06
ARHGEF19	0.7502828	0.000126747	AC010503.5	-0.754054072	0.000201336
PES1	0.750188016	4.43E-138	ESR1	-0.754190777	2.38E-30
NAF1	0.749679233	4.40E-23	SDAD1P1	-0.754246277	0.012478868
NT5C3A	0.748577818	1.15E-36	MIEF2	-0.75431607	5.70E-15
ADCY3	0.748550613	5.10E-70	RERG	-0.754483058	8.30E-07
CACHD1	0.748423942	0.003348738	XBP1	-0.754584906	4.59E-19
IKZF5	0.747821354	2.19E-29	TRIM46	-0.755083094	0.003689312
CCDC88C	0.746703359	8.67E-29	TUBG2	-0.755110542	6.99E-19
RSAD1	0.746284193	3.49E-54	TNRC6C-AS1	-0.755486326	2.87E-06
AC083899.1	0.746203225	0.001809081	AL136169.1	-0.755718187	0.031808909
TAP2	0.745670745	1.30E-36	KANK2	-0.755819343	9.44E-51
BBC3	0.745398721	1.54E-20	BNIP3L	-0.756154349	2.73E-65
AL118516.1	0.745357019	1.07E-05	C18orf32	-0.758757797	7.46E-11
KAT2A	0.745198044	4.95E-58	LINC01503	-0.759020181	0.001155463
UAP1	0.74383082	4.94E-47	AC133644.3	-0.759131019	8.85E-05
MTHFD1L	0.743435995	1.52E-101	DAGLA	-0.759191473	1.57E-05
CIPC	0.742781585	2.19E-39	SMPDL3A	-0.759204906	2.92E-07
CDV3	0.742211695	2.01E-112	UPP1	-0.759926821	3.40E-10
KDM2B	0.742068678	2.41E-47	DIO2	-0.760082973	5.07E-39
TTL	0.741220347	9.68E-45	SEZ6L2	-0.76087567	2.04E-64
TOMM5	0.740732885	4.43E-53	AC016727.1	-0.761146746	0.027324357
TRMT5	0.740185532	5.31E-25	AR	-0.761357559	9.64E-17
MRPS17	0.739968827	2.35E-18	BCAM	-0.761548491	7.97E-10
CCDC85C	0.739855395	1.14E-96	FAM66C	-0.762211476	0.000190561
DNAAF2	0.739274823	1.08E-32	BAG1	-0.762526244	2.54E-106
IPO5	0.738670432	9.58E-125	SYDE1	-0.762760444	5.27E-23
ABCF2_1	0.738649441	1.41E-78	SMIM29	-0.762886524	1.00E-10

DTD2	0.738409978	9.38E-28	GPR37	-0.763174837	0.000487208
SRXN1	0.737675086	7.87E-53	INHBB	-0.763660763	6.98E-110
BCS1L	0.73710609	1.87E-32	A4GALT	-0.763993218	1.98E-07
CEBPZ	0.736208162	8.28E-63	PKIG	-0.764133754	4.07E-13
AIF1L	0.734859143	1.36E-85	TMEM219	-0.764423029	3.93E-20
AC103706.1	0.734723656	0.028321602	ORAI3	-0.766362192	2.16E-21
MBLAC2	0.734457115	9.93E-12	NDRG1	-0.76639955	3.43E-157
WDR35	0.734053754	1.51E-24	GGT7	-0.766534321	1.86E-13
CAD	0.732884804	8.94E-94	PAG1	-0.766580655	0.000689689
MRPL30	0.732564395	2.33E-59	MARCKS	-0.767146645	2.28E-13
SNAPC4	0.732046495	5.72E-52	VMAC	-0.767305652	0.008003478
AKAP1	0.731990275	7.34E-79	AL390066.2	-0.767362968	0.035726081
TRIM14	0.731597988	5.88E-66	CTSV	-0.76784892	3.68E-06
AGPAT5	0.731448597	1.44E-51	MYL6	-0.768451641	7.62E-199
RSL24D1	0.731377201	2.30E-102	CAPN5	-0.769007705	1.68E-05
C1QBP	0.730853567	1.56E-137	BASP1	-0.769013794	2.09E-119
FOSB	0.730685495	0.039766227	RALGPS1	-0.769040006	7.09E-10
NPM1	0.729436802	0	AL109918.1	-0.769058783	0.008497235
SEH1L	0.729431741	1.96E-63	LPP-AS2	-0.769309003	0.000661232
RSL1D1	0.72854435	1.27E-170	CD2BP2-DT	-0.769755308	0.035688784
BCL2	0.72803515	0.009129641	EPHA4	-0.769878935	8.98E-15
AC044860.1	0.726762627	0.030975382	S100A16	-0.770175632	1.91E-176
NOL8	0.725122354	5.10E-69	ERMAP	-0.770190949	2.01E-09
FBL	0.724763272	1.79E-132	SLC16A13	-0.770473441	3.18E-05
CARNMT1	0.723538649	1.31E-23	PARD6A	-0.770492405	1.47E-06
COX10	0.723181238	1.30E-16	TTLL1	-0.771492908	9.66E-06
XPO4	0.722799322	4.10E-40	LINC00857	-0.771548423	0.009760638
CACYBP	0.721961786	2.44E-62	HLA-DRB5	-0.772829679	0.000538286
SOD2_1	0.72150498	9.34E-49	KRT80	-0.772908195	7.39E-242
NARS2	0.717953651	7.54E-30	AC099548.2	-0.773154759	0.002653682
RAPGEF5	0.717272242	3.82E-07	CUEDC1	-0.773305445	5.22E-41
SLC25A22	0.715476623	4.76E-42	UBL3	-0.773797102	1.21E-187
MAP6D1	0.715224289	8.87E-15	EPS8L1	-0.773962089	2.07E-26
CISD1	0.714793055	2.95E-21	MMP24OS	-0.774653473	6.42E-32
GNB1L	0.714632977	5.57E-14	LYPD6	-0.775489119	7.87E-11
AC068946.2	0.714132834	0.045837108	H1-2	-0.775503349	4.51E-08
CDIP1	0.713945274	0.040829031	BMP1	-0.777363851	2.98E-24
PYGO1	0.713920921	0.02024007	MAGED2	-0.77828301	1.53E-62
TUBGCP4	0.713349577	1.33E-34	PPP3CB-AS1	-0.778540937	0.002958351
MRPL3	0.713180249	1.72E-106	IDI2-AS1	-0.780600829	0.002154486
NEIL2	0.711907607	1.00E-12	RMST	-0.780908894	0.024893356
FAH	0.711869491	1.52E-18	AC021087.5	-0.781268093	0.000122711
CU633906.7	0.71158421	3.50E-07	TMEM53	-0.781698343	0.001485617
GNL3LP1	0.710462489	0.000171561	AC093673.1	-0.782248	0.000590743
CEP85L	0.710229741	1.30E-06	EMID1	-0.782398945	2.14E-06
NOP14	0.709526525	3.46E-67	NUTM2B	-0.782796358	0.006185304
ATP11C	0.709001621	6.33E-53	TPM2	-0.782930777	5.24E-21

TRMT10C	0.7090007	3.72E-54	AC132872.4	-0.783270559	0.000678889
NT5DC3	0.708693451	1.02E-08	AL162458.1	-0.78375398	2.11E-10
CDC42EP2	0.707846604	0.000496707	ACKR3	-0.784239206	7.87E-24
SERINC5	0.707818554	1.82E-49	CCDC24	-0.78492719	5.64E-07
DOCK4	0.707814576	0.001072126	ARID5B	-0.786556518	6.23E-26
TAF5	0.707757382	1.48E-10	CYP4V2	-0.787120157	7.28E-10
C20orf27	0.706238004	2.43E-82	TTC39B	-0.787199249	0.030636191
ISCU	0.706080617	2.41E-49	S100A10	-0.787673977	1.92E-162
VPS9D1-AS1	0.705815141	4.39E-27	CCN3	-0.787703268	0.019540271
TIPIN	0.705652871	4.35E-12	SH3BP5-AS1	-0.788731891	3.16E-10
UTP25	0.704322607	2.79E-23	RARA	-0.789668592	1.02E-98
TAF1A	0.703223462	6.36E-09	SARDH	-0.789923077	0.040919347
UNKL	0.702381459	1.67E-16	MAST4	-0.790482136	7.59E-65
XYLB	0.70229961	1.13E-05	RAB19	-0.791487642	0.001242179
CTPS1	0.702003478	2.71E-58	BBOF1	-0.791576313	1.58E-06
DDI2	0.701419757	1.84E-35	MAN1A1	-0.791581183	4.87E-36
NSUN2	0.700183235	7.21E-103	EHBP1L1	-0.791799832	8.49E-69
MRPL4	0.699705266	3.51E-75	INPP4B	-0.792482809	5.12E-65
MBTD1	0.699271224	8.06E-31	IL6R	-0.792933726	1.33E-06
ANKRD27	0.6990105	3.85E-66	TNNT1	-0.79315613	5.14E-31
RNF145	0.698686902	6.04E-09	GPRC5C	-0.793457299	1.34E-25
ZNF639	0.697772989	4.12E-34	GPC1	-0.793533889	1.36E-22
DUS3L	0.696981059	1.34E-29	NPC2	-0.793606907	1.57E-30
KLHL23	0.696977971	6.25E-11	ZNF792	-0.794540786	0.033769686
EBNA1BP2	0.696960595	3.63E-75	CD63	-0.794953239	6.49E-205
IMP3	0.696828745	8.71E-47	MAGI2	-0.79539299	0.000101985
AC002316.1	0.696282256	2.55E-42	TENT5A	-0.795399235	1.38E-35
QTRT2	0.696161518	5.06E-55	TSPAN31	-0.795486078	3.50E-25
TUBBP5	0.696160976	0.039733611	EPHA2	-0.7962114	8.56E-61
ABCE1	0.695978436	6.52E-126	ZNF467	-0.796925039	1.96E-23
CCDC59	0.695408731	1.82E-38	FGB	-0.797499721	0.005619407
GTF2F2	0.695202301	2.91E-32	LYRM9	-0.797744889	0.003583711
NUP188	0.69421085	2.89E-78	RPS10P7	-0.797954318	0.001605431
CDR2L	0.693511385	1.19E-25	CCDC88B	-0.798273996	0.022354572
KATNAL2	0.69098105	0.004232629	IFI27L1	-0.798485445	0.022178932
ASH1L-AS1	0.690432226	0.001142808	GALNT10	-0.798554661	4.28E-88
CEBPA-DT	0.689593993	0.00323748	TRGV9	-0.799425627	0.006231083
RPF2	0.688257031	7.14E-35	SOCS2	-0.800866338	6.23E-07
BRIX1	0.687864189	1.33E-45	HID1	-0.801923104	4.67E-88
GLRX3	0.687544359	4.21E-62	PAQR7	-0.80351316	0.000451807
Z83844.3	0.68724281	2.58E-08	PDLIM2	-0.803817967	1.61E-19
UTP4	0.686937758	1.32E-72	UPK3B	-0.805398823	1.36E-23
EIF1AX	0.68685549	6.89E-100	GNAO1	-0.805430601	0.037328745
DCTPP1	0.68522205	9.71E-106	FGD3	-0.805584692	1.85E-38
ZNRD1	0.685136579	1.56E-15	BTG1	-0.805645675	7.49E-68
CLUH	0.684530744	5.61E-81	AC007406.5	-0.806298443	0.026929606
ARL6	0.684388152	4.90E-08	HSH2D	-0.806378219	1.01E-15

DGAT2	0.684264678	0.034782893	CITED2	-0.806555792	2.75E-45
SERF1B	0.684191172	1.41E-21	TMCO3	-0.807899543	1.61E-45
PIM2	0.684135173	9.94E-12	DNAJC4	-0.808542121	3.76E-19
URB2	0.68299682	6.82E-25	OPN3	-0.808721213	2.16E-21
KDM1A	0.682632427	6.45E-77	ASB16	-0.808972409	0.024797017
ALKBH2	0.682069418	5.56E-21	TMPRSS3	-0.809314138	0.004960792
EOMES	0.681424973	7.05E-06	LIMD2	-0.809769273	0.027237782
MTRR	0.680609281	2.53E-26	AC008556.1	-0.810282524	0.004941234
UTP23	0.680042863	5.34E-32	ENPP5	-0.810437758	3.61E-09
TOMM40	0.679871176	2.72E-82	OCEL1	-0.8113264	1.72E-05
POLR3A	0.679665573	2.59E-38	ARHGAP4	-0.812126071	9.71E-06
LYRM4	0.679555487	9.56E-16	HDAC5	-0.812263087	2.11E-07
MCEE	0.67950619	0.000648301	CLIP4	-0.812462437	0.035405582
UCKL1-AS1	0.677699007	0.011701154	FAM214B	-0.813089765	1.33E-06
YBX1	0.677508587	1.59E-189	MAB21L4	-0.813718622	1.82E-05
ETV4	0.677245766	0.000462109	GSEC	-0.814440923	3.47E-11
FKBP4	0.675385468	7.49E-147	ZBED5-AS1	-0.814553392	0.000790587
ZIC2	0.675313433	2.70E-10	AGRN	-0.815035923	6.95E-234
GTF2H2C	0.675146626	9.98E-39	CLIP2	-0.815194532	0.000171949
ELAC2	0.675007498	1.17E-63	FAM189A1	-0.815256647	0.01852835
NOC2L	0.674961546	1.16E-93	PALLD	-0.816222854	1.71E-52
TMEM158	0.674916054	0.00341067	SLC1A4	-0.816254127	5.48E-10
MEMO1	0.674750015	5.80E-34	ZNF606	-0.81694213	0.002511451
AP000648.4	0.674413673	0.014672217	TMEM198	-0.817033454	0.013497696
SMG1P1	0.6742397	2.02E-13	TTYH3	-0.81832544	6.26E-48
CBR1	0.67403174	6.42E-55	LINC01213	-0.819520601	0.020544665
DLAT	0.673930481	7.14E-44	SPSB4	-0.820420839	0.037067086
DUS2	0.673920817	1.00E-25	PRSS30P	-0.821680143	0.000212585
LRIG3	0.673665394	2.30E-08	OLFM1	-0.821691127	1.52E-29
FBXO25	0.673638204	1.74E-18	SMIM14	-0.821829014	3.11E-73
TNFRSF10A	0.673472967	5.04E-21	SH3TC1	-0.822277887	1.22E-06
IARS1	0.673419634	1.09E-145	TGFBI	-0.822481485	2.59E-23
IPO11	0.6730885	4.55E-40	KIAA0319	-0.822746383	0.001913258
MRPS27	0.673045789	1.64E-73	CCDC170	-0.822942318	1.27E-15
PARPBP	0.672986118	6.66E-20	PRRT2	-0.824941199	4.41E-05
NOM1	0.671993077	3.33E-54	MT1X	-0.824974869	2.19E-21
RMND5A	0.671878054	1.54E-35	PLCD1	-0.825171006	0.005728621
PHGDH	0.671602459	4.36E-41	RPRM	-0.825356724	0.001194996
CARD9	0.671355001	2.51E-05	SLC1A2	-0.826079383	0.00724922
OTUD6B	0.671345484	6.91E-31	ERRFI1	-0.826093378	3.20E-36
EXOSC7	0.670746626	1.33E-27	EPAS1	-0.826628867	5.37E-162
NDUFAF1	0.669331733	3.17E-13	GEM	-0.826971695	0.000552632
ZNF74	0.668960697	5.04E-18	DNAH10OS	-0.827127724	1.91E-05
TIMM17A	0.668910554	3.74E-58	MOSPD3	-0.82744351	1.31E-21
ZNRD2	0.668867586	4.23E-22	SERPINB8	-0.827488151	5.66E-05
TRIM65	0.668800857	5.95E-36	DGCR6	-0.827593086	0.001433052
MRPL15	0.668619532	1.44E-37	ZNF211	-0.827698142	0.00053682

TBRG4	0.667065976	1.25E-57	TK2	-0.827761919	9.26E-08
ZNF749	0.665700394	4.28E-05	PPL	-0.828501534	2.39E-182
PPTC7	0.665576545	2.14E-27	ANXA6	-0.829741961	1.10E-38
PRMT3	0.665312631	1.62E-35	RRAS	-0.829801732	6.32E-12
CA5BP1	0.664970837	4.20E-06	PLEKHH2	-0.830124605	0.000178981
ZNF121	0.664955886	1.18E-24	C9orf72	-0.831563932	0.042773029
GNL2	0.664899381	2.82E-54	CCNG2	-0.832052879	2.61E-48
KLHL8	0.664310709	9.85E-38	CCDC18-AS1	-0.832373309	0.000115432
ZSCAN12	0.663546138	0.029758458	ADGRF4	-0.833582173	5.52E-12
NT5DC2	0.663277282	1.24E-54	KRT8	-0.833911195	0
CLNS1A	0.66299891	4.27E-82	DARS-AS1	-0.833989658	0.002017363
MRPS12	0.662948455	3.41E-43	LINC01135	-0.834217506	0.01075325
HMOX2	0.662571809	5.50E-59	ACSS2	-0.834390559	1.30E-26
SHQ1	0.662427034	3.29E-28	ETHE1	-0.834683969	7.22E-11
PAK1IP1	0.66228849	1.61E-28	ST3GAL3	-0.835269861	0.000453747
KATNB1	0.662146299	8.77E-24	LYST	-0.835330951	6.05E-06
COA6	0.661526765	8.47E-14	PIGZ	-0.836158187	0.000141559
MTIF2	0.661298342	3.39E-47	IDUA	-0.83644418	2.11E-09
AC253572.1	0.660595196	0.004690786	PODNL1	-0.836929366	0.000812282
AGMAT	0.660348341	4.00E-11	FP671120.8	-0.837089059	0.026317718
PIGW	0.660021359	7.58E-22	SEMA3F	-0.837429828	4.01E-20
RPRD1A	0.65937344	4.19E-61	HDAC6	-0.837865862	2.73E-28
ARL5A	0.658785525	1.27E-50	AC022034.1	-0.83876036	9.09E-08
AEN	0.658456717	3.87E-44	SSPO	-0.838919718	6.66E-06
EXPH5	0.657812684	0.003667641	AL021392.1	-0.838953719	0.015283003
TXNL4B	0.657645392	5.97E-15	SCARF2	-0.83933633	0.010703198
CD320	0.657348394	2.18E-34	S100A2	-0.839385403	0.025413628
AC140479.2	0.657098849	0.02826578	KNDC1	-0.839569321	0.014734684
DYRK3	0.65702964	5.88E-08	PPP1R18	-0.839962862	1.96E-29
NOL9	0.656827133	4.70E-23	PLCXD2	-0.840236264	1.59E-06
DNAJA3	0.656288109	1.62E-79	LHPP	-0.841177416	3.14E-16
TSR1	0.656254232	9.91E-84	MATN2	-0.841311612	4.38E-17
DUXAP9	0.656112508	4.33E-13	MEAK7	-0.843319008	4.25E-33
FBRSL1	0.655836704	9.68E-63	SLC25A35	-0.843916905	0.030548763
SLIRP	0.655836626	1.84E-37	HIVEP3	-0.84423049	7.86E-07
KHDRBS3	0.655591988	0.00029782	ANO8	-0.844483603	3.07E-17
MIS18A	0.6554594	5.62E-21	ACHE	-0.845026204	2.11E-09
EIF5A	0.655278202	1.35E-114	ADIRF-AS1	-0.845999688	7.49E-07
GPT2	0.655197346	1.57E-48	UBA7	-0.846622516	7.33E-05
MYCBP2	0.65516844	3.16E-38	LYSMD4	-0.846947019	7.16E-09
PPAT	0.655016517	2.33E-39	AC096677.1	-0.84805379	0.007478463
HYAL3	0.654784305	0.001689087	SLC25A29	-0.848437312	7.95E-41
CCDC78	0.654168474	7.60E-21	PRAF2	-0.84875874	3.14E-20
WDCP	0.653842327	1.91E-10	BMP8B	-0.848926298	3.57E-05
DDX49	0.65365741	1.68E-54	LINC00482	-0.849289194	0.045119696
AC113189.4	0.653394436	5.09E-05	CACFD1	-0.849302686	4.40E-24
ZNF614	0.653212433	0.003972277	AFAP1L2	-0.849735115	2.68E-06

IGSF9B	0.653208076	3.63E-05	ASMTL-AS1	-0.850694499	0.016803453
NAMPTP1	0.652911965	2.58E-11	GYG2	-0.851233482	5.84E-05
AHCTF1	0.652633707	7.27E-50	ZNF703	-0.851588414	1.07E-41
PRMT1	0.652439035	6.29E-104	AC104452.1	-0.851880553	0.000111966
HAUS7	0.65203838	1.26E-10	TIMP1	-0.852178042	4.15E-72
FAM86B1	0.651727762	7.86E-09	RNF224	-0.852641125	0.001231025
AP001505.1	0.651553449	0.00848467	NRP1	-0.854025912	5.49E-152
C12orf45	0.651006939	1.46E-11	TIAM2	-0.854818342	0.000748264
CEBPA	0.650824302	9.35E-11	KIAA1324	-0.855857212	9.43E-27
ELOA	0.650571792	1.11E-58	MUC20P1	-0.856506726	0.019217717
KDM1B	0.650202373	1.94E-18	SCARA3	-0.856657551	3.08E-09
ZNF37A	0.65010432	4.63E-20	LINC00511	-0.856713548	0.000122951
KPNA3	0.649828893	7.31E-48	ARSA	-0.856768777	2.65E-23
POLR3K	0.649590434	1.61E-44	SYTL2	-0.85722052	1.70E-147
FAM222A	0.649522868	1.28E-09	TMBIM1	-0.857841802	0.002389688
SMIM13	0.648809804	5.33E-19	DIRC3	-0.857878818	0.002552948
MRPS26	0.647967719	1.44E-32	GPRC5A	-0.858153796	6.91E-206
OIP5	0.64756134	1.69E-05	RUNDC3A-AS1	-0.859566149	1.50E-08
SRPK1	0.647387644	2.85E-70	NINJ2-AS1	-0.859965466	0.000111669
SLC18B1	0.647075718	1.81E-24	BAIAP3	-0.860474733	9.23E-06
DHX34	0.646678845	2.53E-21	RAP2C-AS1	-0.860596755	0.0076252
NUDT4B	0.646387935	0.000728636	ARSD	-0.860904555	3.00E-55
PA2G4	0.646301117	2.54E-148	ADIRF	-0.861141395	1.43E-18
ZNF598	0.644234535	2.04E-67	TPO	-0.861195831	2.50E-05
SLC25A30	0.644046362	3.21E-10	FHL2	-0.861371343	2.09E-32
AK6	0.64395615	1.23E-31	H4C8	-0.86167152	1.73E-09
MTERF3	0.643643082	3.97E-15	ECE1	-0.861756372	1.05E-162
MTFMT	0.643418192	6.00E-17	ETNK2	-0.861780726	1.05E-22
SLC35F2	0.643392612	2.33E-26	NOXA1	-0.862577773	3.18E-13
FKBP11	0.642688196	1.49E-20	PHF1	-0.863058716	3.51E-29
ISM1	0.642499961	0.008242614	LRRC29	-0.865525826	0.014038716
USP46	0.642440935	1.65E-25	TCAF2	-0.866065842	1.71E-11
SMPDL3B	0.642131243	4.82E-13	SEMA4B	-0.866742251	1.61E-73
CHORDC1	0.641543708	2.28E-43	ARTN	-0.867184567	7.19E-10
DOC2A	0.641274675	1.72E-10	PAPSS2	-0.867804092	1.92E-151
NOB1	0.641099301	4.75E-65	FRG1BP	-0.868529811	4.46E-50
TMEM267	0.641093785	1.67E-12	AC244090.1	-0.868683762	6.65E-05
YDJC	0.640231395	5.03E-36	CLEC2D	-0.868849695	0.000288793
ABCF2_2	0.64016963	2.81E-82	ADAMTSL5	-0.868969608	9.82E-08
FAS	0.639813746	2.35E-17	SHISA4	-0.86955969	0.026430377
SH3GL3	0.639760111	0.005991536	SPACA6	-0.869866216	8.97E-11
NTN1	0.639437527	1.20E-07	JPH2	-0.869869276	8.49E-45
CCDC138	0.639133439	5.88E-08	LINC00461	-0.870045254	0.013156065
TMEM201	0.639083121	4.15E-19	BAIAP2-DT	-0.870900269	4.36E-20
ZNF770	0.639036	5.95E-34	PLXDC2	-0.871721526	8.63E-06
SCO1	0.638640364	6.00E-28	MTMR11	-0.871971188	2.24E-24

EEF1E1	0.637936451	2.31E-24	PTGR1	-0.872095413	0.000130396
ZPR1	0.637800345	5.22E-26	PLCG1-AS1	-0.874211284	0.026513071
THAP2	0.637775063	0.001733038	SLCO3A1	-0.874340586	1.98E-06
EIF3C	0.637625689	2.10E-21	CACNG4	-0.876063866	7.89E-21
NUDCD1	0.637614988	8.08E-59	CEMIP2	-0.876307633	1.45E-82
KARS1	0.637557218	3.96E-132	SEMA4F	-0.876446997	3.21E-10
PTRH1	0.637049205	1.88E-08	EFNA2	-0.876984816	0.042813059
EIF3J	0.63679699	1.23E-62	PPP2R5B	-0.87795368	1.10E-13
OXNAD1	0.636671017	2.13E-16	C5orf38	-0.878432531	2.42E-34
NR6A1	0.636395574	0.000276722	PKIB	-0.878444886	8.19E-14
TATDN2P2	0.636171531	0.017751517	APCDD1	-0.879438018	0.001794722
TOMM34	0.63548982	9.56E-49	SOCS3	-0.880025898	0.009795134
POP7	0.635234426	3.26E-53	YPEL3	-0.880258994	2.10E-34
STMN3	0.635100687	6.07E-05	SDC2	-0.880511629	3.08E-06
EFNA3	0.634813889	1.11E-07	GPX3	-0.881011253	1.85E-15
DDX31	0.634742855	2.33E-27	NEDD9	-0.881173293	1.07E-05
NME1	0.633271137	1.15E-120	RHOB	-0.883876153	2.77E-77
HSPA9	0.633127493	5.24E-157	TMC4	-0.884179623	1.61E-47
PCGF1	0.632848483	8.98E-14	EGLN3	-0.884282104	2.49E-32
UCHL5	0.632682126	6.08E-55	FUT8-AS1	-0.884834469	0.000732663
GAR1	0.632609895	3.76E-25	PTK6	-0.885390585	7.28E-22
AC017083.3	0.631927087	0.019520858	RECK	-0.885922706	0.000211943
AC022966.1	0.631816225	2.81E-44	KRT223P	-0.886064591	7.33E-07
TEX10	0.631516157	3.26E-42	WLS	-0.886330253	0.000129537
SEPTIN6	0.631226739	0.00967856	VSIR	-0.886873025	0.018360576
DBF4	0.631080003	6.36E-44	AC121757.2	-0.887866927	0.013953691
POLR1A	0.630136383	4.81E-52	ABHD17C	-0.889453496	3.90E-92
MRPS23	0.630130149	2.78E-63	CYSRT1	-0.88950985	8.00E-14
AC068547.1	0.629897174	6.51E-08	MICALL2	-0.890336121	3.55E-66
MRPS5	0.629890871	2.01E-40	SNAI3-AS1	-0.890865193	0.003111181
AL445423.3	0.629780464	0.01833967	SH3D21	-0.892782224	3.40E-24
SPATA5	0.629326955	5.64E-08	AK8	-0.892827605	0.017051425
NADK2	0.629163821	6.73E-20	KIF9-AS1	-0.892856383	0.00449598
DCAF4	0.629039537	5.64E-28	LMCD1	-0.89339331	5.81E-12
FP565260.1	0.628704059	8.94E-34	PDZD7	-0.89377512	0.013618654
NEU3	0.628543434	1.62E-20	S100A6	-0.894486925	3.11E-151
NAMPT	0.62809735	5.94E-96	FLNA	-0.895299741	2.45E-256
SLC7A6	0.627973884	7.08E-59	HLA-DQB1	-0.896181305	1.41E-41
NHP2	0.627620323	1.50E-118	LINC00894	-0.897285063	0.012839189
PEX5	0.62704597	1.52E-43	CASTOR3	-0.897808739	7.43E-27
C12orf73	0.626677426	1.16E-07	GLRX	-0.897972382	0.008949304
PLCG2	0.626343272	1.78E-05	SOCS1	-0.898314788	0.027494545
ATXN7L2	0.625882342	8.96E-08	OBSL1	-0.898326442	2.35E-139
GART	0.624804384	2.14E-85	RAB26	-0.898357022	5.73E-17
CDK5R1	0.624463667	0.008724759	ENO2	-0.901269609	6.40E-13
RRS1	0.62433157	3.34E-31	COL6A1	-0.901281316	0.000394305
UTP14A	0.624211534	7.43E-65	H3C6	-0.901383683	4.38E-08

ZDHHC23	0.623966523	1.28E-26	ALDH1A3	-0.901938155	6.29E-35
RNF138	0.623788672	1.43E-27	MDK	-0.902757541	9.75E-44
SMAD6	0.623224712	1.44E-05	HMCN1	-0.903649564	6.94E-14
TSKU	0.622880195	5.76E-07	TPBG	-0.903651614	1.36E-186
TTC27	0.622479206	1.03E-22	PEG10	-0.904861412	7.31E-43
LRPPRC	0.62225029	8.78E-131	SCNN1A	-0.906281445	2.47E-09
KIAA0930	0.621979912	4.98E-41	TFF3	-0.907225411	3.26E-35
RPARP-AS1	0.621816547	4.59E-07	TXNIP	-0.907662193	5.69E-116
EPB41L4B	0.621402849	5.04E-31	C8orf58	-0.907913755	1.15E-20
PAM16	0.621230152	2.11E-18	UNC5A	-0.909195701	0.000923733
RIOX1	0.621043839	1.61E-21	IGSF8	-0.909547533	9.30E-24
ZNF587B	0.620982001	4.50E-08	CCN5	-0.910049317	1.20E-116
ADSL	0.62013226	5.83E-56	ALDH3B1	-0.910294607	3.96E-32
CNKSR3	0.619763438	2.05E-06	CNIH2	-0.910448894	3.58E-05
MTFR1	0.619344289	5.21E-43	SLC12A6	-0.910505108	1.56E-16
NTPCR	0.619226918	4.97E-17	LAMB2	-0.91069631	3.29E-99
SRPX	0.619004556	0.001756013	FAM20C	-0.910734809	4.42E-10
LTV1	0.618989503	8.71E-51	CDKN2D	-0.91282336	0.000247481
SPESP1	0.618813842	0.000479072	HDAC11	-0.913537942	7.38E-16
ANKRD16	0.618325076	2.10E-08	LTBP2	-0.914907994	9.45E-09
AC146944.3	0.617886863	0.009026955	SERPINA3	-0.915033639	0.000170936
NAA15	0.617343617	1.11E-68	BX470102.2	-0.915462492	0.016244534
TBC1D30	0.617164309	3.16E-46	PBLD	-0.916196213	2.05E-10
CCNB1IP1	0.616475016	6.88E-21	ST6GALNAC4	-0.916420494	3.16E-07
HSPE1-MOB4	0.616108044	0.000139371	EGFR	-0.916509967	1.98E-28
SUV39H2	0.615950351	1.11E-21	ITGA3	-0.916688955	1.14E-190
SMG1P3	0.615870057	0.0011639	TSPAN14	-0.916795864	9.80E-129
NOL11	0.61586933	3.66E-82	CBFA2T3	-0.91796575	5.90E-36
POLR3E	0.615255415	8.49E-45	AL670729.3	-0.918292814	0.006484422
ME1	0.614919884	7.53E-32	MICB	-0.918952454	9.94E-06
TRMT6	0.614699304	3.94E-16	AMOTL2	-0.918999271	3.80E-78
GATAD2A	0.614625863	1.28E-49	SPATA6	-0.919589099	0.003429369
CIART	0.614443675	4.66E-09	LINC00271	-0.920754106	0.020649736
DGKE	0.614253959	5.21E-20	CXCR4	-0.920819249	2.11E-70
EEF2KMT	0.613661231	6.22E-25	QDPR	-0.921049977	3.19E-14
EDA2R	0.612547133	7.31E-12	GALC	-0.921966375	0.002570655
LRRC59	0.612308412	6.08E-115	AP001453.5	-0.922256001	0.011644266
SMYD5	0.612137848	1.44E-20	PXDC1	-0.92241945	4.07E-07
GUF1	0.611495103	4.18E-24	SELENBP1	-0.922629093	5.98E-10
DYRK2	0.611493945	5.68E-33	DRC3	-0.92300212	0.000835881
ZBTB44	0.61133672	1.78E-26	ISG20	-0.923100675	1.61E-07
DDX18	0.611296075	9.36E-78	COL6A2	-0.924097657	6.21E-07
MRPL1	0.610928074	1.11E-25	AC016831.6	-0.924999669	0.019410888
FUT10	0.610766194	1.90E-05	ABCA7	-0.925758436	1.54E-08
POLR3D	0.610509332	1.61E-22	IQCD	-0.925936353	0.000315899
CTSC	0.61012671	4.51E-18	LXN	-0.926241283	5.91E-76
RDH13	0.609883174	4.34E-14	CLDN9	-0.927297038	2.67E-18

PCSK6	0.609864788	9.48E-13	AL139385.1	-0.927847885	0.002412509
CUTC	0.609743917	2.13E-10	LDHD	-0.928731085	0.000481525
EFHD2	0.609392119	5.26E-34	CYP46A1	-0.928760171	0.006167656
ANGEL1	0.609297215	2.35E-38	AC010168.2	-0.929630106	6.08E-05
DDX51	0.608865927	3.29E-19	PDK4	-0.930175257	0.001941865
ZNF589	0.608701642	1.65E-06	MYZAP	-0.930968755	0.000116136
SNHG21	0.608494983	0.009543184	AC244197.3	-0.931273766	3.84E-12
GPATCH4	0.608186982	6.55E-55	AC007114.1	-0.931960689	0.006258314
CCNJ	0.608145	3.24E-15	RTKN2	-0.932782748	5.58E-18
WDR17	0.608136246	1.14E-05	AC008014.1	-0.933739592	0.001602725
PSPH	0.608047238	2.38E-15	AC007319.1	-0.933923744	0.000385824
KNSTRN	0.607451166	8.10E-15	TAT	-0.934465811	0.024263778
SLC27A2	0.606728845	3.58E-06	L1CAM	-0.935518425	8.11E-157
TRMU	0.606362826	2.89E-28	VAMP1	-0.935841004	4.47E-11
STX6	0.606187231	2.38E-44	TFPI	-0.935995616	3.59E-38
SIK1B	0.606141004	2.62E-17	AC098582.1	-0.936345578	0.001547279
AC091959.3	0.606005315	1.65E-05	IL11	-0.936512086	0.006441311
LYPLA1	0.605721762	2.05E-50	ALPK3	-0.937015054	1.39E-23
SDAD1	0.605583928	2.59E-46	KRT19	-0.937316269	0
NOP2	0.605428289	5.08E-60	MELTF	-0.937919021	5.65E-31
KTI12	0.605260079	3.48E-08	ABCC13	-0.938053056	0.010302068
SRM	0.604730668	8.04E-70	TMEM45A	-0.938059815	2.03E-07
HSPE1	0.604489196	3.34E-101	PRKCA	-0.938871516	2.62E-50
MAD2L1	0.604402663	1.30E-42	TLCD2	-0.940365015	5.69E-10
BEGAIN	0.604123458	0.037568782	RAI2	-0.94039923	0.009458437
CDK8	0.603634562	9.11E-24	AC007686.3	-0.940807301	0.00646438
PRXL2C	0.603501815	5.65E-13	GRAMD2B	-0.942216245	6.64E-27
ZNF711	0.602768933	0.026209687	SLC66A3	-0.943325067	2.78E-22
ALKBH8	0.602265111	1.16E-09	C1QTNF6	-0.944848252	1.42E-172
LSM11	0.601962726	4.85E-11	AC080112.5	-0.946294338	0.011450665
NAA50	0.601209222	2.53E-118	SMPD1	-0.9470936	5.21E-48
PPIH	0.600922429	9.69E-11	PAPLN	-0.947335528	4.30E-06
ZNF420	0.600153404	7.03E-05	DNMBP	-0.948378967	2.58E-25
LIMK2	0.59964608	6.44E-24	CLTCL1	-0.948696299	3.10E-06
AHSA1	0.599407201	8.45E-98	AC027601.1	-0.948775965	2.55E-05
SKA3	0.599368935	2.71E-19	SLC25A24	-0.949536308	6.21E-182
AK2	0.598934584	2.39E-75	F2R	-0.949612869	2.19E-12
PACRGL	0.597765284	2.39E-08	NAV2	-0.950461217	8.02E-228
MAX	0.597293167	5.01E-38	ITGB4	-0.950679306	2.32E-144
LETM1	0.596983911	1.18E-53	ST8SIA6	-0.952005756	1.63E-36
IGSF9	0.59696201	8.55E-16	S100A14	-0.952244757	6.10E-21
MRM1	0.596326208	8.43E-07	ENTPD2	-0.953706738	5.27E-06
MTPAP	0.596008282	1.23E-21	PRKD1	-0.954106337	1.98E-11
PRELID1	0.595124856	9.06E-121	KIFC3	-0.955099418	3.43E-27
NOP58	0.594506309	2.55E-63	CTSK	-0.955309572	0.000235557
MAPT	0.594430596	2.02E-13	ARRDC3	-0.955568309	8.21E-14
PROCR	0.594067877	9.29E-05	POLD4	-0.955761686	1.08E-39

ZNF593	0.594055967	1.12E-12	COL5A2	-0.956109197	3.18E-13
RHBDF2	0.593583217	8.54E-24	GPR137C	-0.956369915	5.23E-05
MAP7D3	0.593387927	1.44E-28	KPNA7	-0.956485881	0.012121951
XRCC2	0.592957222	1.47E-18	NAALADL2	-0.95683193	0.001614339
TUFM	0.59283605	1.50E-136	MICAL1	-0.95688843	6.57E-25
GRK3	0.592503558	1.72E-06	ADAMTS13	-0.956889981	2.85E-12
DTWD1	0.592445594	7.69E-23	TNFAIP2	-0.957459144	0.00081734
TSFM	0.59202564	1.13E-30	PDGFB	-0.95765405	3.17E-35
SERTAD2	0.591914472	4.33E-14	SLITRK6	-0.959831342	1.83E-11
IFRD2	0.591628926	4.78E-42	PDE11A_1	-0.961418463	8.79E-05
MARC1	0.591469642	3.63E-11	FAM110C	-0.961709228	1.24E-54
GRWD1	0.591395189	1.45E-29	BCAR3	-0.962888577	4.38E-32
ATP5MC1	0.591322727	9.70E-32	PALM2AKAP2	-0.963292626	0.000748492
C16orf91	0.590428991	3.55E-12	KRT7	-0.964202122	1.06E-93
WDR97	0.589871455	3.94E-08	ABTB1	-0.964312542	1.91E-13
TFAM	0.589805993	5.21E-53	IER3	-0.964969614	1.01E-122
PDCD5	0.589749901	3.66E-28	IFITM10	-0.965167253	1.64E-07
TMEM102	0.589707917	6.99E-08	RHCG	-0.966079894	0.009470302
KIAA1958	0.589471964	3.82E-12	PRR36	-0.967624485	0.000280882
ATP13A3	0.589058264	9.49E-91	CPE	-0.967664415	1.55E-66
GTF2H2B	0.58845236	0.000205951	STX1B	-0.967690177	0.000545376
MFHAS1	0.588087285	3.19E-20	SPOCK1	-0.968651476	4.35E-31
MRPS31	0.587623087	1.09E-09	TSPAN5	-0.96938097	1.99E-26
THAP11	0.587357086	8.51E-26	PGM5	-0.97044187	2.08E-07
SPG21	0.587122198	7.59E-43	SEC14L2	-0.970773417	2.62E-18
PPT2	0.58708764	2.54E-13	PRR15	-0.971861725	1.40E-12
ZBED6CL	0.586971301	2.35E-06	SNED1	-0.972108894	4.72E-06
FBXL4	0.586507277	1.07E-09	DDX60L	-0.97211796	1.73E-05
POLH	0.586386105	1.99E-24	APH1B	-0.972855203	3.41E-09
ZNF114	0.586378042	0.000508406	TUBA1A	-0.97326681	6.15E-57
CDC25A	0.585677141	2.05E-09	GLB1L	-0.974404571	1.20E-06
DKC1	0.585016179	1.65E-73	SLC22A18	-0.976026624	8.29E-20
EXOSC3	0.585003207	3.83E-19	AL390038.1	-0.978415654	4.33E-06
			MRPL23-AS1	-0.979556013	0.000102776
			SLC16A4	-0.980850952	1.32E-05
			PBXIP1	-0.98137028	9.20E-63
			FAM102B	-0.982653882	3.90E-123
			GTF2IRD2	-0.982910374	1.05E-10
			PRICKLE2	-0.983628768	2.27E-05
			ATP1B1	-0.983722545	2.20E-39
			H2BC21	-0.983772757	9.96E-63
			SPAG4	-0.984325929	5.58E-11
			DNAH1	-0.984428587	0.000550516
			EFHD1	-0.98444481	3.52E-11
			LHX2	-0.984671399	0.000551853
			CCDC151	-0.98527942	0.008917696
			MATN3	-0.987212831	0.002850517

PRKAA2	-0.987329188	4.70E-05
MAP1B	-0.987854508	0.000121004
TGFB2	-0.988153124	1.81E-08
PTK2B	-0.988292689	3.59E-07
ABLIM2	-0.988604986	0.009055127
FAM114A1	-0.988712158	1.91E-13
AP001816.1	-0.990193617	1.99E-13
AHRR_1	-0.990773281	0.001468151
PTPRE	-0.991138641	5.04E-24
PCOLCE	-0.991553475	3.08E-14
SLC25A42	-0.992770357	2.50E-17
DAPK2	-0.994320251	1.32E-28
FOS	-0.995003681	4.20E-105
TNS1	-0.995968468	1.69E-05
VPS9D1	-0.99794637	2.77E-13
FRMD3	-0.999505025	0.003374447
MUC20	-0.999806002	2.03E-19
TRGC1	-0.99986839	1.01E-36
FIBCD1	-1.000022068	2.43E-105
NRM	-1.000185505	4.15E-08
EBF4	-1.000551535	0.000425287
TCEAL3	-1.001992229	1.70E-40
CRIP1	-1.002417344	0.000890266
NHS	-1.002643713	3.22E-82
ANXA3	-1.003647517	1.99E-29
GNG7	-1.003819604	3.57E-07
EDN1	-1.005038854	2.01E-08
SELENOM	-1.005531153	0.000596863
LONRF3	-1.005566162	8.07E-16
ALOXE3	-1.005712794	3.64E-07
SCART1	-1.006196872	1.49E-05
DMPK	-1.006881081	4.34E-161
AC016682.1	-1.008363858	1.59E-05
AC126564.1	-1.008464125	8.31E-07
AC108047.1	-1.008513247	0.000892911
DNAJB5	-1.008802244	0.000100931
IRS2	-1.008819057	2.13E-21
NEURL1B	-1.009135013	1.22E-12
ZNF365	-1.009148876	5.39E-19
ARHGAP33	-1.010088353	2.59E-09
PLEKHG2	-1.010525335	1.89E-28
RHOBTB2	-1.012162691	7.32E-103
EHD2	-1.01443977	1.90E-20
GFRA1	-1.014840712	0.004963315
TOX2	-1.015313208	2.20E-05
NPAS2	-1.015926959	1.58E-102
AL137003.1	-1.016765684	0.000214257

	AL359258.2	-1.01783557	2.43E-07
	SLC22A17	-1.019723291	1.31E-06
	RAB9B	-1.020043195	4.81E-08
	AL590004.3	-1.021572059	3.16E-34
	MLPH	-1.022413585	6.98E-290
	MIR210HG	-1.022727	9.46E-10
	GMDS-DT	-1.022773873	0.000790462
	AC021066.1	-1.023463513	9.23E-70
	AC099568.2	-1.025514789	0.008239526
	PLAUR	-1.025937542	8.21E-19
	GPR37L1	-1.026318975	2.32E-13
	PLD1	-1.027938937	5.90E-17
	ANXA1	-1.028417516	3.37E-05
	TENT5C	-1.028640778	2.26E-10
	LINC00888	-1.028706258	4.44E-09
	GLP2R	-1.028962635	1.20E-06
	BEST1	-1.032013674	0.002395962
	HEG1	-1.032582507	1.22E-08
	VAMP5	-1.03408942	2.79E-05
	MEIS3	-1.036137348	1.33E-16
	ECHDC2	-1.036708777	1.68E-32
	SEMA5B	-1.038898576	7.49E-27
	SNTB1	-1.039035439	1.27E-18
	BDKRB2	-1.040306358	3.33E-20
	RUNX2	-1.043052613	6.43E-08
	AC144450.1	-1.043475692	1.67E-09
	ADAM12	-1.043770662	0.001081413
	CTSO	-1.044994418	8.04E-11
	LINC01963	-1.046112042	2.95E-08
	AC007541.1	-1.046952814	0.003896249
	LINC00365	-1.047663425	1.27E-12
	LINC01257	-1.04788999	0.000375019
	TMEM40	-1.049124762	3.09E-10
	MPZL2	-1.049233801	4.67E-39
	AC015802.6	-1.049324559	0.00046244
	TMOD1	-1.050658582	0.000395969
	KLK6	-1.050856687	2.36E-05
	IZUMO4	-1.055242628	0.008899422
	SCX	-1.05527439	1.22E-10
	PPP1R14B-AS1	-1.055417683	5.56E-07
	CAPN9	-1.056566936	0.000424543
	CGNL1	-1.057216749	0.000214069
	DDAH2	-1.057328587	6.52E-45
	DOK7	-1.058078925	2.64E-18
	LOXL2	-1.058174411	2.71E-241
	PTAFR	-1.05888192	1.98E-09

COL4A5	-1.059423019	1.35E-12
RET	-1.060030859	3.55E-11
SDCBP2	-1.060098688	0.000240587
C5	-1.063425723	1.57E-17
C14orf132	-1.065733449	3.63E-155
LMO7	-1.066062477	4.37E-104
GSN	-1.066410336	5.17E-201
CD109	-1.067613808	1.07E-116
PLTP	-1.070087861	0.00345868
AC023158.1	-1.070456043	3.50E-12
MAN1C1	-1.070583723	0.004601515
NRCAM	-1.072411925	1.22E-55
ITGA5	-1.073936569	1.69E-54
PDGFC	-1.077228469	0.000733653
IGFBP3	-1.077763884	1.63E-21
IGFBP4	-1.078732076	4.07E-38
ZG16B	-1.081072758	1.38E-22
ST8SIA4	-1.083059048	0.003391242
TNFRSF11B	-1.083891233	6.78E-13
CHST3	-1.085332146	9.61E-08
STIMATE-MUSTN1	-1.086080326	0.00210221
TLE6	-1.087518153	0.005320396
ZNF185	-1.088096722	1.08E-73
SCNN1D	-1.089371517	0.000448474
SEMA3C	-1.090043891	1.04E-241
EFEMP2	-1.090395031	0.004046018
RAP1GAP	-1.090475729	4.19E-51
AC068580.4	-1.092007569	1.56E-26
EGR3	-1.093050655	1.07E-10
SYT16	-1.09646784	0.001484698
GSTM2	-1.098980578	0.000280419
MKRN2OS	-1.099477362	0.000206982
SLFN5	-1.099787386	5.74E-12
CEACAM6	-1.102473991	4.88E-48
AL359258.1	-1.104058469	4.15E-08
PKD1L2	-1.104562169	2.05E-08
AC141930.1	-1.104962347	0.000835594
PROS1	-1.106948478	0.000204389
C9orf106	-1.107422057	0.00448266
LIPA	-1.109658024	9.55E-67
SYNGR3	-1.11209336	3.21E-14
GDPD5	-1.11216413	1.33E-14
DLGAP1-AS1	-1.112729821	0.001422376
PRSS23	-1.11723797	1.78E-109
PLA2G10	-1.120022879	0.000733887
DAB2	-1.121634062	9.00E-21

ARHGDIB	-1.121737641	4.07E-05
BMERB1	-1.123382284	1.67E-29
ULBP2	-1.124015059	2.34E-32
GOLGA8O	-1.126440899	0.001031885
LRRC6	-1.128301382	0.00051614
PTPRM	-1.128370629	3.78E-52
ADCY5	-1.129533817	1.29E-134
SUSD2	-1.130089197	4.98E-05
MAPK11	-1.130743871	7.04E-27
TRIB2	-1.13192809	1.34E-10
BTC	-1.133938722	5.99E-24
RAB4B	-1.134894557	6.84E-12
B4GALT1	-1.135252171	0
TTLL7	-1.135319326	1.12E-05
CMAHP	-1.135651931	0.000437268
ARNT2	-1.136509844	2.66E-99
CMYA5	-1.138140419	3.77E-19
PTGS1	-1.138394802	7.33E-07
MST1	-1.14121229	1.81E-05
ABAT	-1.146611152	8.50E-104
KLF6	-1.147226429	1.04E-39
F2RL1	-1.147295497	1.63E-97
RTN2	-1.14951986	1.00E-07
ATP2A3	-1.150029201	0
ANGPTL4	-1.151240036	5.33E-69
TMEM8B	-1.151384572	2.98E-07
CAPS	-1.151447867	0.000175171
MEGF6	-1.151616883	1.98E-93
LTC4S	-1.152400114	0.000897201
PHLDA1	-1.154182732	6.63E-77
PYROXD2	-1.160254415	6.02E-18
TMEM45B	-1.161873964	8.47E-71
PCDHAC2	-1.168865494	1.66E-07
FER1L4	-1.169828295	3.61E-148
MYEOV	-1.169836743	7.55E-144
ITGB5	-1.174251226	1.26E-271
SLC22A18AS	-1.177415889	1.30E-09
CCDC96	-1.177509277	1.70E-05
CLCF1	-1.177573282	2.79E-19
EFEMP1	-1.177860876	3.79E-38
AC079834.2	-1.177910295	0.00104899
TUBB3	-1.178830627	3.92E-247
DOCK2	-1.178978199	8.45E-05
HPGD	-1.180220522	0.001099621
AP003419.1	-1.181444875	9.94E-10
VSIG10L	-1.181981393	1.13E-08
RPS6KA2	-1.184820223	1.92E-30

	CLU	-1.185349236	0
	SERPINA11	-1.188857794	2.86E-08
	CAPN2	-1.191341575	7.58E-54
	FAM155A	-1.191933603	0.001189207
	CD59	-1.193552792	0
	MAP2	-1.193633317	1.97E-13
	AC080112.4	-1.196171133	2.63E-05
	CDK14	-1.199142492	0.001313388
	SYTL5	-1.199749516	2.80E-56
	FN1	-1.201151436	1.60E-178
	CCDC9B	-1.202624691	0.00013118
	VTCN1	-1.203481776	9.88E-46
	TMSB4X	-1.204811158	0
	AC011498.7	-1.204940624	0.001046642
	REEP1	-1.205738723	5.04E-08
	LYPD1	-1.21214165	2.13E-06
	CCN2	-1.215564154	0.00014635
	LAMA3	-1.216635466	1.82E-12
	ALOX5AP	-1.223346324	0.000496447
	COL12A1	-1.223594157	5.50E-30
	CASTOR1	-1.229525006	1.82E-05
	ALOX5	-1.232539765	4.59E-09
	SYT8	-1.233330633	6.41E-07
	NTN4	-1.234345408	9.70E-145
	PSCA	-1.234396006	6.36E-07
	SCN1B	-1.234735123	2.61E-12
	SYTL4	-1.24133737	3.29E-25
	ITGB6	-1.242533251	7.96E-266
	RASSF8-AS1	-1.244687642	6.26E-07
	TFF1	-1.245883689	9.31E-23
	CLIC3	-1.245945578	1.84E-88
	TP53INP2	-1.24907749	1.70E-82
	EMP1	-1.249907866	7.07E-07
	KCNN4	-1.253764819	5.01E-75
	DUSP4	-1.254188752	1.58E-74
	NR2F1	-1.259284788	1.65E-72
	PLCH2	-1.263478641	3.71E-05
	MIR9-3HG	-1.26571405	1.16E-26
	MB	-1.26725239	8.28E-37
	LYPD3	-1.268122398	1.00E-84
	LAMB3	-1.269892112	1.15E-27
	SLC25A24P1	-1.270139376	1.53E-08
	CAMK2N1	-1.270648861	0
	TSPAN1	-1.272903002	3.67E-39
	MYO16	-1.273528105	8.70E-07
	MYPN	-1.276091327	2.70E-22
	CALML5	-1.277680716	5.38E-05

IL1RAPL2	-1.277802717	1.15E-05
GSTM4	-1.281498702	6.88E-27
C15orf48	-1.283533152	3.71E-06
TIMP2	-1.284146669	1.95E-66
MAGED4	-1.285774771	0.001826075
AC135048.1	-1.288238981	0.000185801
AGAP11	-1.292992034	3.42E-05
AC005077.4	-1.293081673	5.00E-05
DBN1	-1.298708056	9.56E-95
CYP26B1	-1.299260435	6.30E-10
IL18	-1.301257706	1.02E-07
DUSP6	-1.302924888	2.33E-09
FGD5	-1.303308336	1.61E-14
C1orf116	-1.312235834	0.001382223
ADORA1	-1.312296535	6.04E-08
DUSP5	-1.312830961	6.21E-28
PADI1	-1.314075751	7.21E-29
SHISA2	-1.314141156	4.13E-06
LINC00346	-1.314439316	0.000185734
AC007743.1	-1.315741157	9.13E-05
MVP	-1.325585211	6.97E-107
ZMAT1	-1.328951239	7.06E-08
MXRA7	-1.329116652	7.54E-08
TNIK	-1.329373427	4.72E-11
SSPN	-1.331643398	3.18E-23
ITGA2	-1.336844923	4.80E-281
AC068580.3	-1.341007567	1.73E-10
MDGA2	-1.341961538	3.52E-08
LMNTD2-AS1	-1.344957686	6.65E-11
MUC1	-1.348592537	9.96E-58
MYO15B	-1.350039951	2.98E-30
PWWP3B	-1.35308147	4.14E-08
SMOC1	-1.354862021	1.01E-10
AC006372.1	-1.356667321	1.77E-06
KRT15	-1.356759747	2.25E-38
ARHGEF40	-1.358442972	9.12E-07
IGF2	-1.365256653	5.41E-06
AREG	-1.368436787	1.85E-51
TLE4	-1.368780983	5.10E-09
SPNS2	-1.368883157	6.60E-254
AC110619.1	-1.368936564	4.34E-43
LY6D	-1.371104347	1.56E-07
TRIM6	-1.372444927	1.80E-05
WSCD1	-1.37301073	0.000111794
INHA	-1.375529241	1.69E-23
LOXL1-AS1	-1.375657052	1.09E-32
GIPR	-1.377182384	6.61E-06

NT5E	-1.382575044	1.60E-116
TH	-1.383183828	8.57E-11
ITGA6	-1.383458878	4.01E-63
TMEM139	-1.383828717	1.38E-09
AGR2	-1.389380168	6.59E-189
CTSD	-1.393175573	1.17E-147
FSCN2	-1.396255501	7.92E-08
SNAI2	-1.39706152	6.03E-13
AC144831.1	-1.397938462	5.64E-12
SPEG	-1.40492618	5.82E-14
TRIM29	-1.407244799	1.76E-42
SEMA5A	-1.410663254	1.18E-12
ABCA4	-1.414487154	2.21E-58
SEMA3B	-1.42182957	6.68E-134
NR2F1-AS1	-1.424046756	1.82E-30
C19orf33	-1.430253452	3.26E-80
SERPINE1	-1.44785977	4.50E-06
PADI3	-1.448725241	7.80E-21
MST1R	-1.450151216	9.56E-27
KIAA1210	-1.47651881	7.66E-47
MGP	-1.481671255	1.75E-65
AL359258.3	-1.487667441	1.93E-08
PADI2	-1.489907634	5.04E-55
LGALS1	-1.495955713	4.03E-269
ANOS1	-1.502916068	1.57E-09
GREB1	-1.505236579	0.003298798
AL354740.1	-1.507407552	5.63E-08
THSD4	-1.508972917	4.61E-156
EFR3B	-1.514173541	1.89E-18
STEAP4	-1.517424097	8.18E-40
LINC02747	-1.518237904	4.75E-13
ABCC3	-1.523152162	0
CDH5	-1.525819687	4.57E-13
BANK1	-1.526951352	8.46E-11
BEX5	-1.538050173	4.76E-13
CEACAM5	-1.553352993	3.83E-06
SLC1A1	-1.570638826	2.21E-06
AC006372.2	-1.578424464	1.49E-21
TCIM	-1.585160411	4.90E-51
SLC34A3	-1.586044103	4.87E-07
MT2A	-1.590291053	1.98E-175
ST6GALNAC2	-1.590832707	6.20E-37
PLAU	-1.595981565	2.28E-06
GLRA3	-1.598434811	6.35E-22
AL157935.2	-1.600207141	2.13E-06
EDIL3	-1.603923183	9.41E-78
NBPF4	-1.615014793	4.32E-60

	LINC02015	-1.629046717	9.28E-13
	EPGN	-1.636755785	1.40E-21
	MIR503HG	-1.63884573	2.42E-23
	COL5A1	-1.645493624	1.75E-163
	LAMC2	-1.65792692	2.39E-83
	LHFPL6	-1.665586801	5.35E-107
	SLC16A2	-1.67723997	2.36E-18
	MALL	-1.688436007	0
	PLXNA2	-1.688876036	2.36E-26
	KRT87P	-1.719505073	4.45E-14
	CAPN8	-1.726291568	1.59E-89
	IL1R2	-1.735584545	4.03E-07
	WNT9A	-1.743968325	9.76E-08
	ZNF175	-1.77143255	1.04E-111
	SLCO2A1	-1.77904464	1.20E-66
	LOXL1	-1.79377765	2.79E-30
	RASD1	-1.795056556	4.30E-67
	INHBA	-1.802737	3.47E-45
	ITGB2	-1.81361342	6.95E-32
	AC010735.2	-1.814787546	1.72E-18
	CEMIP	-1.815763871	7.77E-134
	BHLHE41	-1.857791935	6.24E-51
	SH3PXD2A	-1.875814987	1.91E-13
	AQP3	-1.900488735	0
	KRT81	-1.950926333	8.78E-232
	CRAT	-2.076159493	1.98E-19
	PARM1	-2.082062945	1.41E-36
	MAPK4	-2.093529367	2.45E-12
	LRRC15	-2.12138021	3.36E-223
	NBPF6	-2.197294403	4.67E-12
	MUC5B	-2.251527635	5.86E-37
	PTGES	-2.305958301	1.98E-25
	PHLDB2	-2.31875021	3.49E-43
	KRT16	-2.384858374	8.04E-77

Table S6: mRNAs significantly up- or downregulated (≥ 1.5x fold change) in MCF-7/pRTR-c-MYC cells (*AP4* KO/*p53* wild-type).

Significantly up-regulated mRNAs			Significantly down-regulated mRNAs		
Gene symbol	Log_2 fold change	padj	Gene symbol	Log_2 fold change	padj
MYC	3.28722142	0.004774	NUF2	-0.585194531	0.019781
AC026786.1	3.067044988	0.007369	HMMR	-0.585649869	0.0002701
GAL	2.895081475	7.91E-07	ARL6IP1	-0.586071579	1.68E-10
CR2	2.893193773	0.003379	TNRC6C-AS1	-0.586342387	0.0048751
FABP5	2.827023559	3.26E-05	ENO2	-0.586561705	0.0048265
USP2-AS1	2.764689266	0.000323	RIMS4	-0.58667388	0.0006572
CPNE7	2.708385505	6.36E-05	EIF2AK3	-0.586834017	2.67E-11
PDE4A	2.462555678	0.000161	MMUT	-0.587238821	3.43E-08
GPD1	2.401137118	0.005829	FARP1	-0.58763445	1.20E-13
WNT10B	2.377748655	0.002143	UBALD2	-0.58769105	6.44E-08
DUSP2	2.164117992	3.17E-05	ILK	-0.587856789	8.10E-07
AC040162.1	2.043756195	5.21E-07	ITGA6	-0.588262834	0.0087272
MATK	2.03481721	6.54E-07	CYTH2	-0.588590914	1.70E-10
HOXC8	1.897315082	1.39E-09	TJP3	-0.588844155	4.96E-05
TRPM6	1.889774923	2.87E-08	TTC30A	-0.589001424	0.0008014
DNAH17-AS1	1.858670302	1.52E-07	SDHAP3	-0.589062408	0.0221487
TMEM52	1.853805859	2.94E-15	DUSP18	-0.589134763	0.0037348
KCNQ4	1.844297876	5.33E-11	RAD51AP1	-0.589136739	0.0037607
TBC1D4	1.820300463	0.000297	KIF14	-0.589272508	5.55E-05
SLC16A1	1.818299478	1.12E-05	ZNF718	-0.589377836	0.0002968
POLR3G	1.817744895	2.60E-06	SORT1	-0.589678644	5.44E-18
HS3ST3B1	1.798972438	4.60E-10	PALM	-0.58981179	2.24E-08
HPDL	1.794283491	6.98E-34	AC067930.8	-0.589929475	0.0303275
ADGRE2	1.784735316	5.36E-13	OSCP1	-0.590081749	0.0288053
AC007342.4	1.78282138	5.63E-11	ALCAM	-0.590295472	7.03E-12
PCOLCE2	1.772590627	3.75E-05	SDSL	-0.590624219	0.0002452
RPP25	1.748080926	1.82E-37	C2orf68	-0.590678067	2.61E-07
PLD6	1.735383838	4.24E-19	PPP2R3A	-0.590702838	6.05E-05
SORD	1.725743307	1.36E-09	SGCB	-0.591068966	6.58E-06
SNAI1	1.702042049	9.32E-06	SLX4	-0.59109456	1.34E-05
SORD2P	1.683398855	5.59E-06	KIAA1324L	-0.59122286	6.68E-11
NR1D1	1.67222758	2.16E-12	CENPF	-0.591387104	4.13E-08
EFCAB10	1.670373525	8.62E-07	OPTN	-0.591441975	2.61E-09
EN2	1.666839744	2.33E-18	BCAM	-0.591621365	3.34E-06
SLC27A5	1.664851052	1.57E-34	MARCHF2	-0.592245745	2.44E-05
FJX1	1.651654105	2.49E-25	KIF11	-0.592671584	4.87E-06
CENPV	1.646990504	4.18E-12	MRC2	-0.592766849	3.42E-05
EMSLR	1.639336351	1.97E-45	SP1	-0.592789614	7.35E-16
PPARGC1B	1.599259412	3.83E-13	KIF2C	-0.592820248	8.48E-05
TERT	1.58946981	1.38E-05	AL499602.1	-0.592967326	0.002566

MIR17HG	1.575170715	3.98E-05	ZIC4	-0.593104782	0.0053402
CAMKK1	1.574602519	3.01E-11	PNPLA8	-0.593873716	1.36E-11
MXI1	1.559532892	2.80E-06	TSPAN14	-0.594005337	2.67E-08
ACSM3	1.556688446	0.000453	SH3BP5-AS1	-0.594179384	0.0088305
VWCE	1.554310344	4.68E-06	TAPBP	-0.594900611	1.33E-13
RNF125	1.546454531	3.23E-16	MC1R	-0.595049252	9.49E-08
AC073896.1	1.53923272	7.24E-05	IQSEC2	-0.595210772	0.0001137
TRNP1	1.499832437	5.31E-14	MINDY1	-0.595640108	1.54E-05
PODXL2	1.49824917	1.19E-28	LRRC37A16P	-0.595805179	0.0195539
EEF1AKMT4	1.498208157	1.05E-30	MEGF8	-0.596273646	4.29E-11
FAM89A	1.48336387	5.45E-11	ATAD2	-0.596552323	1.62E-08
ADAT2	1.48281648	2.92E-34	ANKRD50	-0.596894916	4.28E-10
GALNT18	1.451764515	6.00E-19	DLGAP5	-0.596969169	2.89E-05
NPM3	1.449073199	5.11E-45	KIAA1109	-0.59729344	1.48E-06
ADAMTS17	1.445313916	1.64E-06	FRYL	-0.597561402	6.53E-09
FAM216A	1.440379133	4.81E-22	ANK3	-0.598570768	2.62E-09
CMTM8	1.433236888	1.29E-12	ANKMY2	-0.598940285	0.0004811
COQ8A	1.430760124	1.19E-23	TCIRG1	-0.59923007	6.92E-05
RAB3IL1	1.427436541	3.60E-27	CDK2	-0.599700897	1.78E-06
LRFN1	1.413375976	4.16E-08	MMP16	-0.59972659	0.0143787
RRP9	1.404975368	1.59E-38	CTTNBP2NL	-0.599755941	7.90E-10
GFOD1	1.398059447	2.53E-21	IRF9	-0.599887039	0.0077813
PFKM	1.391478931	1.31E-22	SEZ6L2	-0.600074799	8.63E-16
TAF4B	1.388361753	2.01E-14	ATP9A	-0.600132039	8.28E-10
AP002387.2	1.38593386	5.89E-11	BCAR3	-0.600199348	2.61E-05
GUCY1A1	1.384472475	8.23E-05	TCAF1P1	-0.600349729	0.0002596
SCO2	1.376258614	1.39E-24	PPARD	-0.600400518	3.26E-07
SNHG30	1.368243782	4.37E-20	MAN2B2	-0.600519856	1.06E-08
RABEPK	1.367789337	2.61E-24	TRIM16L	-0.601369852	2.18E-06
CCDC85B	1.362418165	3.46E-41	STMN1	-0.601390993	0.0247041
PITX1	1.360694337	8.70E-43	SEPTIN8	-0.601678322	4.19E-13
RPL23AP7	1.360079621	2.23E-07	PKIA	-0.601722225	0.0017552
ARID5A	1.358173391	5.59E-15	GNAI2	-0.601731807	1.64E-08
RGS16	1.34403611	7.04E-17	DTL	-0.602740392	1.21E-05
FTL	1.34263755	1.84E-30	AC073508.3	-0.602875342	0.0128145
SLC25A19	1.341983569	6.46E-28	AC006372.2	-0.603192185	0.0448173
SLC29A1	1.337967449	3.20E-43	ZCCHC24	-0.603198731	0.0227024
ANKRD13B	1.330896753	6.95E-24	RNF19A	-0.603554092	3.44E-11
SLCO4A1	1.325203228	8.20E-20	NPTXR	-0.603577571	7.18E-06
AP001505.1	1.319656909	0.000117	KIAA0232	-0.603870971	4.78E-12
NANOS1	1.316193813	1.05E-11	SLC37A1	-0.60408708	2.97E-13
METTL8	1.311854351	4.76E-25	KIF15	-0.604607165	0.000715
RPIA	1.311267276	3.31E-28	TPM4	-0.605136905	1.96E-14
GADD45A	1.306643902	3.26E-13	TBC1D2	-0.605138878	7.50E-08
NTHL1	1.30511695	4.85E-23	KITLG	-0.605310787	0.0003848
CTU1	1.299344114	5.97E-21	PLEKHA6	-0.605423086	1.26E-06
TWNK	1.289199139	5.19E-23	CEP162	-0.605484082	0.0002419

NRARP	1.288543898	5.05E-24	SLC6A8	-0.606560699	0.001108
PPIF	1.288487073	4.65E-31	YBX2	-0.606631099	0.0008482
SH2D5	1.282740526	6.70E-11	VSIG10	-0.606703527	8.14E-11
AC105052.2	1.282140629	0.000603	EVPLL	-0.607169064	0.0482804
GALNT14	1.274237499	1.37E-16	DEF6	-0.6078563	0.0310042
RNF145	1.262683985	5.66E-10	CARD14	-0.607942141	5.67E-06
MNX1-AS1	1.259501477	1.15E-12	RIMKLA	-0.608541418	0.0391861
CCDC86	1.258712146	2.81E-41	PRIMPOL	-0.608660842	0.0016256
SMKR1	1.250419826	5.89E-17	IQCC	-0.608928427	0.009649
NALT1	1.249513336	0.003448	TNFAIP8	-0.609446236	0.0002895
AP006333.1	1.248889885	8.46E-08	FOXO3	-0.610075259	2.13E-12
AC020763.4	1.24827274	0.000179	LRRC23	-0.610339005	0.0111596
CIART	1.243784492	9.23E-19	CCN1	-0.610397986	1.15E-08
CDKN1A	1.236199599	1.18E-22	MOCS1	-0.6106855	0.006916
OAF	1.235822723	1.84E-16	LYRM9	-0.610762388	0.0135043
LYAR	1.235507806	1.68E-27	LINC00265	-0.610905805	0.0033015
DLEU1	1.231379986	1.23E-14	NEBL	-0.610987846	5.11E-15
DPH2	1.227320672	5.15E-25	UGDH	-0.611745841	4.93E-10
AL139353.1	1.226774652	0.000896	WSB1	-0.61198826	5.27E-14
PCOTH	1.224880414	0.001134	FADS3	-0.612084703	1.77E-06
OVGP1	1.222580923	0.001936	USP46-AS1	-0.612224911	0.0170569
EPOP	1.216638451	2.51E-28	S100A10	-0.612244568	7.37E-13
ASPHD1	1.214072397	6.72E-15	CPS1	-0.61312191	0.0002333
ACTL8	1.209700497	0.000385	PMM1	-0.613152396	2.49E-05
PRR5	1.201741186	6.61E-29	NIT1	-0.613670611	2.60E-06
CARMIL2	1.201459024	6.93E-12	FLNB	-0.613701783	2.53E-21
ZIC5	1.201353466	1.09E-11	CARD10	-0.614248099	0.0032164
RPUSD1	1.200206481	7.62E-46	IFT22	-0.614416356	5.65E-13
AMER1	1.198888568	3.09E-15	SHANK2	-0.614571773	8.48E-07
CD320	1.197479994	1.62E-31	LINC01515	-0.61463009	0.0423735
SH3GL3	1.197474866	0.004398	ZFP36	-0.615000937	2.85E-05
YRDC	1.197119482	5.95E-23	PDLIM1	-0.615255713	3.54E-07
ZNRF2P1	1.191061568	0.000104	KIF1A	-0.61528533	0.0144543
AL118516.1	1.190590288	2.79E-10	ZNF268	-0.615374829	1.63E-07
PNP	1.188060542	3.20E-22	C1orf116	-0.61558339	0.0376709
MLKL	1.18676147	7.29E-16	BACE1	-0.616040816	5.00E-10
KLHL21	1.182219901	1.50E-19	RAB26	-0.616751252	1.20E-05
SLC25A32	1.182142934	4.82E-20	MEIS3P1	-0.616975741	0.043423
ZNF296	1.181144874	4.08E-14	AC002116.1	-0.617261252	0.0476524
CD3EAP	1.180917944	1.58E-33	MAP3K6	-0.617336252	1.84E-07
NDUFAF2	1.180207288	2.07E-19	RAB5B	-0.617670749	1.96E-16
PAQR5	1.179849092	0.004067	CENPE	-0.617825676	6.27E-06
ARC	1.178032841	2.06E-06	PAXIP1-AS2	-0.618017994	0.0001318
RPL23AP82	1.175992731	1.07E-16	RECQL4	-0.618322174	1.38E-06
MON1A	1.175583208	6.19E-18	ZNF680	-0.619415794	0.0003551
BTG2	1.174242283	0.005663	SEC14L2	-0.619986816	0.0011616
FIRRE	1.173406796	1.61E-12	SEC24D	-0.620447915	1.40E-11

NDUFAF4	1.172147067	2.53E-33	CAP2	-0.621564388	1.68E-05
NOP16	1.171949904	5.56E-34	NCOA1	-0.621884579	5.00E-09
SPHK1	1.171664785	1.28E-22	LINC01719	-0.622169907	0.0163759
FAM131C	1.162199262	3.11E-06	TBC1D8B	-0.622651575	6.92E-06
TRMT61A	1.161214153	6.10E-34	ANXA2	-0.622995135	2.24E-14
TFB2M	1.161077044	8.60E-23	KCTD11	-0.623001698	1.04E-06
AC009831.1	1.157663445	8.16E-05	GRIN2D	-0.623278019	0.0390749
MAP3K21	1.157415783	1.17E-11	FZD2	-0.623461382	3.50E-06
MCRIP2	1.152807386	2.77E-31	SESTD1	-0.623536501	3.39E-09
PER1	1.151213325	1.42E-10	HIVEP1	-0.623557746	0.0002435
L3HYPDH	1.148106633	3.24E-13	VMAC	-0.624440758	0.0298819
FAM162A	1.13869177	6.71E-19	TAF9B	-0.624555901	2.07E-09
ELL3	1.1383217	1.48E-10	CCDC50	-0.625059804	4.47E-14
RPP40	1.135767332	2.24E-24	TMEM35B	-0.625219452	0.0015663
EXOSC4	1.135244238	4.11E-34	ASAP3	-0.625602596	3.98E-05
NAT8L	1.133493975	2.18E-18	GTSE1	-0.626105795	1.18E-06
AC048338.1	1.13335003	0.001351	PLXNA3	-0.626696078	5.89E-11
PDXP	1.122382575	3.15E-17	PDE5A	-0.626866352	0.0008145
BAG2	1.122121931	4.59E-07	PTPN21	-0.627599113	1.21E-05
ID2	1.121294738	7.36E-13	PREX1	-0.627715016	1.64E-10
C1QBP	1.119408913	7.79E-31	MOSPD2	-0.628331101	4.68E-06
MRPL36	1.119236163	4.39E-38	MXRA7	-0.628529	0.0038965
RPUSD4	1.116886435	2.18E-25	ZNF606	-0.628726049	0.0428702
AL161772.1	1.115781925	5.03E-08	EHHADH	-0.629001051	0.000542
AC027097.1	1.115016235	0.014134	ERBB3	-0.629801199	1.95E-16
SLC19A3	1.11245269	0.001355	ODF2L	-0.630453466	0.0014305
SFXN4	1.112364353	3.24E-21	ATXN1	-0.630799334	0.0001248
EDA2R	1.112345466	5.39E-15	PXDN	-0.630818886	5.23E-11
GRIN2C	1.11073004	2.26E-06	CD82	-0.630866262	0.0096753
NFE2L3	1.102018942	1.56E-13	PDP1	-0.631640862	0.0001056
EXOSC5	1.101974285	2.49E-38	ZNF793	-0.631829705	2.77E-05
F12	1.101137214	1.37E-18	MELTF	-0.632048762	7.50E-06
RAB3A	1.096977876	2.59E-08	AC084018.2	-0.632838222	0.0367127
RRP1	1.094049833	1.24E-29	FIG4	-0.633559271	9.46E-09
ATP6V1C2	1.093193368	1.80E-05	UPK2	-0.633826587	3.13E-06
CDC42EP1	1.092881879	8.05E-27	AC005332.6	-0.63418726	1.18E-15
TRAP1	1.092662863	3.83E-35	GTF2IRD2	-0.63432981	0.0030059
JAG2	1.091354061	2.82E-14	ACSF2	-0.634471616	3.36E-06
DEPTOR	1.090391269	4.14E-11	SLC25A29	-0.634593733	8.36E-09
GRPEL1	1.088411894	1.06E-23	RMI2	-0.634665992	8.57E-05
CRYM-AS1	1.084264196	0.003332	SUOX	-0.634667646	4.26E-06
FXN	1.082937163	1.22E-14	IGSF3	-0.6347843	1.58E-21
SLC9B2	1.08288479	8.91E-14	EPG5	-0.63516212	5.80E-07
ANP32A	1.0814946	1.21E-18	TENT5C	-0.635660031	0.0401221
LINC01703	1.081169536	0.01098	AGRN	-0.637519949	4.44E-18
PAM16	1.076793154	9.44E-28	SNX10	-0.637820176	2.70E-05
DIMT1	1.075245762	7.45E-22	LINC00888	-0.63794871	0.0016431

CHCHD4	1.074634863	4.70E-19	SRGAP2C	-0.637984637	2.20E-08
TMC5	1.072684543	6.60E-06	VEZF1	-0.638049261	2.41E-12
RNASEH1-AS1	1.072402708	9.96E-15	GLIPR2	-0.638673397	0.0052211
C15orf61	1.071264926	5.65E-13	SLC46A3	-0.638742459	6.59E-06
MRPS30	1.070747694	4.47E-26	SKIL	-0.638821105	1.29E-15
GEMIN5	1.070670613	8.59E-19	CREBRF	-0.639284286	0.000522
C12orf73	1.067533142	7.00E-16	LINC02591	-0.639432545	0.0031182
E2F5	1.06659861	1.85E-21	PDZD4	-0.639575919	0.0366444
REXO4	1.063996905	3.02E-21	KLF4	-0.640761613	2.17E-06
IMPDH1	1.063641501	7.02E-24	GUSB	-0.640980043	7.23E-12
LYSMD2	1.060069201	1.33E-19	HSD17B1	-0.640997961	0.0005454
PSMG1	1.059992331	2.02E-28	ABTB1	-0.64146377	3.29E-05
PDCD2L	1.059401709	7.16E-15	C5orf34	-0.641504962	0.0138703
UNC93B1	1.056623239	1.06E-18	FOXJ2	-0.641555004	5.00E-09
SSC4D	1.056463496	0.017125	EPHX2	-0.642445187	0.0075576
POLR1C	1.054196706	2.52E-25	PARVA	-0.642560143	6.66E-12
BOP1	1.053321407	4.65E-27	PRR15L	-0.642761057	2.47E-09
HMGA1	1.050691386	3.42E-28	ZNF596	-0.642848622	0.0094089
ZNF239	1.047615316	3.00E-16	AC005332.5	-0.643169976	0.0052823
MPP6	1.043414446	3.71E-10	ZBTB10	-0.643528794	1.37E-07
NLN	1.041464672	1.45E-18	KLHL5	-0.643551605	6.54E-12
FAM117B	1.037888252	2.13E-14	MREG	-0.64356262	0.0138472
SNRPA1	1.036348345	4.52E-36	KIFC1	-0.643797438	3.17E-05
PALD1	1.036081946	2.14E-05	AC010186.2	-0.644192532	0.0022786
SMG1P2	1.035683918	2.99E-08	POT1	-0.644310133	3.36E-05
C19orf73	1.03407095	0.028396	DOP1B	-0.644550108	2.32E-14
CHCHD10	1.033002986	8.20E-20	NUTM2B	-0.645238906	0.0194109
PFDN2	1.032216242	2.82E-26	TEDC2	-0.64595867	0.0011374
THAP2	1.031603362	1.78E-05	SFXN2	-0.64598128	0.0002233
ICAM5	1.031452019	4.68E-06	RND1	-0.646041114	2.35E-05
SLC25A22	1.030189611	2.04E-19	SPSB1	-0.646217568	0.002824
MARS2	1.029982767	2.86E-17	ST3GAL3	-0.646701817	0.0123661
ADORA2B	1.029256129	1.51E-11	GALNT10	-0.646717252	3.40E-09
CMSS1	1.029077278	7.79E-31	CHRD	-0.647074664	0.0005365
NEIL2	1.028034805	2.95E-15	HECTD2	-0.647361785	0.0004764
UCK2	1.027736343	1.73E-22	PARP11	-0.647586346	0.000709
PUS1	1.027437	7.23E-27	TMEM254	-0.647590051	5.40E-07
MRPS12	1.02740622	2.54E-32	PHYH	-0.647609483	2.60E-05
CA11	1.026510966	5.85E-05	POT1-AS1	-0.647811129	0.0124848
SIK1B	1.024763598	3.11E-09	LINC01547	-0.649946101	0.0006027
LYRM4	1.023549889	5.18E-17	BCL9	-0.650109825	2.35E-11
CNNM1	1.023283679	8.32E-09	XRN1	-0.650260423	7.99E-07
EIF3CL	1.020543315	0.000414	LDHD	-0.650296549	0.002359
AC009412.1	1.01852458	0.017049	ZNF112	-0.650784143	0.0443973
DGUOK-AS1	1.01599712	0.000561	ECHDC2	-0.651106178	1.34E-07
IL17D	1.015911917	1.20E-15	ZNF888	-0.651477935	0.0424153
SCARNA2	1.013580231	0.029727	MYH14	-0.651891346	1.01E-15

SOX12	1.011379082	8.19E-25	SPC24	-0.652035212	1.99E-05
NKD2	1.011263994	1.69E-08	WDR31	-0.652770654	0.0159281
FCRLB	1.008008645	0.001283	RCAN1	-0.653057035	3.82E-05
YBX3	1.005366009	3.12E-25	TPST1	-0.653146206	0.0008712
LINC01311	1.004997288	0.001698	EPS8L1	-0.653178879	5.97E-08
TOP1MT	1.002521345	2.16E-22	CALHM2	-0.653418758	4.19E-05
MRM3	1.002320882	3.11E-27	FUT9	-0.653501378	1.36E-07
PRR19	1.001150887	5.45E-08	CBX7	-0.653576638	3.77E-07
MRPS2	0.999140578	7.76E-30	TOM1L2	-0.653817913	1.06E-12
RIOK1	0.996880274	2.46E-21	LTBP2	-0.654035943	0.0051278
KCTD12	0.99426701	1.51E-07	AL049834.1	-0.654209226	0.0016314
SRPX	0.992311888	0.00018	KRT7	-0.654226414	4.14E-11
MBLAC2	0.991719327	2.27E-13	TSPAN31	-0.654300035	8.36E-11
YDJC	0.990837099	2.46E-25	MGAT4A	-0.654408549	2.60E-11
SUPV3L1	0.990000534	8.12E-16	DNAJC22	-0.654645256	2.19E-05
TRMT11	0.989007742	3.33E-11	FZD7	-0.654863851	0.0068826
SLC25A33	0.987814113	9.42E-25	EVI5L	-0.65487506	4.58E-12
COA6	0.98693319	4.49E-14	NOD1	-0.654891574	0.0209297
PPAN	0.986353729	1.16E-27	CDKN2D	-0.655673348	0.0057461
SLC19A1	0.984970721	8.94E-29	CDYL2	-0.655725183	1.51E-09
DDX10	0.977356332	1.10E-21	AC022107.1	-0.655769168	0.000106
ARMC6	0.976485995	1.54E-25	VAV3	-0.655840813	4.00E-06
GRB14	0.975544354	6.79E-12	LY6G5C	-0.65586162	0.0477048
POLR3K	0.974456674	1.34E-31	RBL1	-0.656006825	2.65E-07
NDUFAF8	0.973799368	1.57E-23	IFI35	-0.656182938	0.0024704
H2AW	0.972811911	4.53E-12	ZFHX3	-0.656348438	6.10E-10
EIF2B3	0.971961747	4.71E-19	JAK2	-0.656401237	0.000469
ZC2HC1C	0.971781284	0.019093	FAM214B	-0.656718589	0.0010487
ZIC2	0.967683704	2.45E-11	EML5	-0.656729041	0.0050488
FAM81A	0.963731151	1.39E-09	CEP97	-0.656910354	6.17E-06
SNHG4	0.962115582	3.42E-12	ZNF467	-0.656915957	4.86E-07
TRMT1	0.961286248	2.87E-30	TSPAN15	-0.657144777	4.79E-12
KRTCAP3	0.959314519	2.94E-10	RNF38	-0.657403849	2.06E-06
DOHH	0.958258763	6.77E-17	TTC30B	-0.658129768	2.74E-05
C20orf27	0.958110888	5.75E-42	COL9A2	-0.658190103	0.0019692
CEBPD	0.957678559	2.10E-09	IKZF4	-0.658998528	5.24E-05
PPAN-P2RY11	0.956901985	8.96E-06	DBN1	-0.659414475	1.50E-08
NHP2	0.95563234	1.40E-30	KIF20A	-0.65982978	1.46E-05
JPH1	0.95482623	3.63E-13	MBNL2	-0.65989914	7.83E-11
DNAJC2	0.951208629	4.27E-22	PRR11	-0.660074491	9.20E-07
SNHG10	0.947690422	1.74E-18	TIMP3	-0.660488829	0.0026778
UQCRFS1P1	0.947672647	0.000286	SYDE1	-0.660633291	1.36E-07
EXOSC7	0.946887118	2.09E-15	SIPA1	-0.661502057	0.0004533
LRFN4	0.945851058	7.54E-32	DRC3	-0.662184852	0.0412368
PHB	0.945820863	7.45E-36	DSN1	-0.662313459	1.02E-07
AEN	0.944215725	9.05E-30	CREB3L2	-0.662718927	7.14E-12
CCNG1	0.941860773	2.45E-12	DNMBP	-0.66340967	0.0001289

DMAC1	0.93926169	3.30E-19	TFAP2C	-0.663746728	7.77E-11
ALKBH2	0.937849222	8.44E-21	MYH9	-0.664237532	2.66E-12
PRMT1	0.937045843	2.53E-33	SMTN	-0.664582846	3.60E-08
IMP4	0.936478948	7.10E-36	PARD6A	-0.664854924	0.0006834
FAM174C	0.935808276	3.30E-24	GPRASP2	-0.664995226	0.0004103
PPM1J	0.935564315	2.63E-05	RGPD8	-0.665028999	0.0001009
DNAAF2	0.935359568	7.33E-27	PPM1E	-0.665705972	0.0098766
FKBP11	0.934778598	2.30E-18	HFE	-0.665910479	0.000231
HSPA4L	0.933382475	1.51E-16	POF1B	-0.666697276	0.0003621
UFSP1	0.93284268	7.16E-09	ARMCX3	-0.66711018	1.79E-08
ZNF593	0.932667683	1.57E-16	ZDHHC12	-0.667254485	1.63E-07
RITA1	0.930957306	1.29E-38	ZNF493	-0.667737326	0.0307379
MYBBP1A	0.93050646	2.69E-19	ZWINT	-0.668196288	2.01E-08
LDLRAD3	0.929319167	5.90E-11	AC027601.6	-0.668272089	0.004797
MCAT	0.929262089	2.12E-17	PBXIP1	-0.668351025	1.14E-09
FASTKD1	0.928063238	6.44E-14	TUFT1	-0.668411076	1.26E-09
ID1	0.927832292	2.14E-12	AGR2	-0.668499268	1.54E-06
ZNRF2P2	0.927360126	0.043429	MDK	-0.668952206	4.08E-09
SLC2A4	0.927193653	0.024271	FCHSD1	-0.669428172	5.97E-06
GLS	0.926802601	1.93E-15	ZNF75D	-0.669525999	1.33E-10
THAP4	0.924575111	2.53E-21	SKA2	-0.670856803	1.95E-12
SMAD6	0.924549753	2.82E-05	S100A14	-0.670889157	0.0206586
SLC6A6	0.924506104	0.030232	LMNTD2-AS1	-0.671671878	0.0159674
PPT2	0.923625104	3.01E-13	NCOA2	-0.671856747	7.85E-15
RRP12	0.923557041	1.55E-23	ZNF737	-0.672017386	0.0027173
PNO1	0.923525122	6.24E-22	SP2-AS1	-0.672628246	0.0158767
RRP1B	0.923200112	3.02E-16	RPL23AP87	-0.673749652	0.0327608
ECSIT	0.923187269	4.22E-17	BMF	-0.674019968	9.39E-05
NXPH4	0.922963307	3.12E-10	ZNF555	-0.675101457	0.0082951
ODC1	0.922687609	7.06E-23	TSPAN1	-0.675187941	1.95E-05
FAM189A1	0.922107987	0.049182	SNX21	-0.676242443	2.59E-12
ABCC4	0.919765591	1.71E-08	ANO6	-0.677309337	1.71E-05
CFAP157	0.919729037	0.00026	HOTAIR	-0.677541618	0.0337161
RINL	0.919573167	0.001637	MLPH	-0.677572034	4.01E-18
TYW3	0.918676082	3.14E-13	ZNF100	-0.677595365	1.65E-05
ANKRD16	0.91859061	3.11E-08	RAB30	-0.677915909	0.0001197
STOM	0.917884293	4.18E-12	TPM1	-0.67799199	4.03E-12
EIF4EBP1	0.917880237	6.58E-17	SEMA4F	-0.67863886	0.0001309
MCF2L-AS1	0.917574473	0.012199	CEP295	-0.67955695	1.82E-08
REXO2	0.916886014	6.21E-18	SSH3	-0.679609205	2.61E-21
KIF9	0.915869716	2.19E-07	SARM1	-0.680016775	0.0437175
DUSP23	0.915322938	1.96E-25	MB	-0.681013224	0.0039365
KBTBD6	0.914895413	1.91E-11	SPTSSB	-0.682054729	2.75E-28
ADRA2C	0.913517263	1.11E-09	PHC1P1	-0.682288927	0.0196312
MRPS17	0.91239572	2.48E-16	SLC16A6	-0.682302765	3.80E-05
NCS1	0.912135041	6.94E-23	SPRY4	-0.682333402	0.0002294
TFAP4	0.911800615	1.27E-18	KCNN4	-0.683272482	0.0002426

POLR1B	0.911514851	9.50E-20	DNAH10OS	-0.68345235	0.0016858
WDR3	0.911327995	1.69E-21	DHTKD1	-0.683536246	9.21E-09
RNF19B	0.911274268	2.92E-09	RESF1	-0.683973713	3.71E-05
TNFRSF10A	0.910833724	2.16E-11	TDRD7	-0.684691506	7.81E-12
AC012676.1	0.90965812	0.006078	CASP8AP2	-0.68485337	5.90E-11
BTN3A2	0.909656724	0.017155	LRP1	-0.68518988	0.0003407
CALML4	0.908795644	0.001462	TTC28	-0.685299448	0.0005099
PRELID3A	0.908207731	1.98E-07	ARHGEF38	-0.685423849	0.0132824
DHX37	0.907074523	1.26E-22	TM7SF2	-0.685437473	2.20E-11
CNKSR3	0.905669326	6.83E-06	ASF1B	-0.685999839	8.39E-06
NAGPA	0.902987661	4.22E-17	KIAA0319	-0.686454959	0.0381623
RPARP-AS1	0.902884917	2.19E-11	DZANK1	-0.686715819	0.0009751
CC2D2A	0.901778952	2.21E-10	PIGZ	-0.68686395	0.0086591
STC2	0.901315673	4.53E-25	AC004233.2	-0.686921292	5.11E-07
CBR1	0.90020381	2.76E-27	ALDH3B2	-0.687078201	4.21E-08
HDHD5	0.89916121	2.75E-20	ENPEP	-0.687221336	0.0205528
C15orf39	0.898269819	6.31E-12	STIM1	-0.68807972	2.56E-09
AL024508.1	0.897548105	9.95E-09	FLVCR2	-0.688158202	1.76E-13
PES1	0.897216632	7.97E-22	ERAP1	-0.689168329	1.31E-08
UBIAD1	0.896425904	3.81E-14	ZNF287	-0.689569116	0.0004638
SLC25A37	0.89600657	1.28E-16	IKBKGP1	-0.689576704	0.0404986
TIMM44	0.894806454	4.33E-20	GALNT4	-0.689900328	0.0007674
RPS27L	0.893258611	8.45E-19	AC068587.4	-0.69007514	0.025978
SLC27A4	0.892297046	3.66E-29	PLAUR	-0.690632686	0.0388236
BLMH	0.889271076	1.05E-21	MT1X	-0.691584859	0.0043729
MRPS26	0.889093032	4.56E-23	MOV10	-0.691957604	1.42E-17
CYCS	0.888931037	2.24E-31	SMIM5	-0.692110923	9.56E-08
GNB1L	0.888778685	1.91E-12	CUL9	-0.69214821	1.13E-13
CTU2	0.888480672	3.85E-16	FLT4	-0.69242029	0.0178075
PUS7	0.887938611	2.62E-15	HS6ST3	-0.692524014	0.000138
SPESP1	0.887138841	1.66E-05	KIAA1211L	-0.693045045	2.42E-10
MRM1	0.886137329	4.96E-10	MAST4	-0.693069667	1.07E-09
NFKBIB	0.885440558	1.73E-22	PTPRE	-0.693425219	9.36E-07
AKAP1	0.885407814	2.15E-16	FADS2	-0.693614143	1.42E-05
SIRT4	0.883824489	0.000648	RNASEL	-0.693883979	7.31E-07
NME1	0.882410515	6.92E-25	HLA-F	-0.693995834	0.0023212
HSPE1	0.882372246	1.27E-20	PCDHB9	-0.694342264	0.0424819
WDR74	0.881375902	7.05E-24	ICA1L	-0.694712231	0.005432
NAF1	0.880854854	9.18E-18	STX5	-0.694723273	5.54E-14
ACSS1	0.88023726	0.00089	TCTN2	-0.694779205	5.12E-06
AC012146.1	0.879537426	0.006983	JUN	-0.694805815	1.83E-05
USP31	0.879467933	1.26E-16	CYSRT1	-0.695096916	1.82E-08
ESRRA	0.878702662	7.50E-22	HPSE	-0.695149652	0.0075869
LBHD1	0.878480818	2.88E-07	MFGE8	-0.695320193	0.0003584
ENTR1	0.878103015	3.58E-34	CEP126	-0.695628865	0.0144971
CHAC1	0.877382097	2.18E-10	C22orf46	-0.695676134	2.02E-13
PDSS1	0.876236903	1.51E-11	IL4R	-0.695703793	2.25E-09

ADCY3	0.875871486	4.56E-23	BNIP3L	-0.695987268	2.64E-10
MRPL4	0.875429029	2.03E-29	TTC3P1	-0.696174768	8.69E-06
ALDH1B1	0.875031206	1.91E-17	LARP6	-0.696456508	0.0053991
SLC45A3	0.874959782	1.04E-08	SLX1B	-0.696772378	5.66E-09
SINHCAF	0.874820636	9.01E-12	FAM102B	-0.697066656	5.39E-11
RPL23AP42	0.874333731	0.030097	MNS1	-0.697412782	0.0030834
ACTL10	0.874181298	1.83E-07	TMEM45B	-0.697520764	0.000567
PSAT1	0.874171782	2.59E-28	BAZ2B	-0.697666252	7.53E-09
SLIRP	0.873324794	3.01E-13	SPC25	-0.697748532	0.000148
BCL11B	0.873048029	1.98E-07	GAB1	-0.697949519	2.14E-17
ASB13	0.872145486	3.37E-17	LMCD1	-0.698258742	9.63E-07
ACAT1	0.87129018	3.46E-13	STAT2	-0.698370415	4.84E-13
MRPS6	0.870554769	4.64E-19	CRYL1	-0.699131349	3.51E-06
FAM189B	0.869577005	5.58E-16	H3C6	-0.699310141	0.0011855
NCBP2AS2	0.868886591	6.93E-27	LINC01572	-0.700221027	0.0095345
SNHG21	0.867960915	0.001005	TSPAN9	-0.701521699	9.16E-08
CISD3	0.867627119	5.88E-18	SNX29	-0.702081004	6.56E-11
COTL1	0.866805437	3.91E-24	UBL3	-0.702259191	3.24E-25
MET	0.866474836	7.74E-09	RAB9B	-0.702642078	0.006617
RUVBL1	0.865559513	9.21E-25	SUCO	-0.702847076	2.37E-09
GNPDA1	0.864740411	8.34E-19	KIFAP3	-0.703206684	0.0001519
PIM2	0.86470734	1.50E-11	ARMT1	-0.703414782	0.0369137
S100A4	0.863935789	0.001399	FMO5	-0.703558836	0.0134373
TOMM5	0.863261781	8.43E-22	GUCY1B1	-0.703602366	0.0002771
FBL	0.863158672	4.61E-13	TK1	-0.703752365	3.06E-07
EEF1E1	0.86287856	4.17E-18	DIAPH3	-0.703790986	2.26E-05
TIMM13	0.862499154	1.50E-19	DMTN	-0.704037082	2.25E-09
KATNB1	0.862325997	7.71E-20	AL365181.3	-0.704056061	7.16E-05
DOC2A	0.861837551	6.27E-13	CLHC1	-0.704864703	0.0230009
AC109322.1	0.861446492	1.20E-05	GRB7	-0.705073491	8.12E-07
UBR5-AS1	0.860248957	0.007516	NAP1L2	-0.705195842	0.0031628
DUS3L	0.860015588	8.76E-20	FNBP1L	-0.706053465	3.83E-14
MRPL50	0.859332906	2.95E-15	SNED1	-0.706564281	0.0131906
AL445423.3	0.858805499	0.004876	RAPGEF2	-0.706868176	0.0080514
DCUN1D5	0.858600716	4.60E-14	AC004816.1	-0.707237246	2.79E-05
BOLA2B	0.858086851	2.60E-18	SMARCD3	-0.707695536	1.90E-08
AL645608.2	0.857882888	0.045464	ETV5	-0.708483008	7.39E-08
VPS9D1-AS1	0.857762685	8.87E-15	PHF1	-0.708783636	7.85E-11
STK32C	0.856067627	9.40E-13	FBXO41	-0.709319774	0.0012826
EBPL	0.856045001	3.09E-15	PARP14	-0.709484985	1.27E-16
CHCHD6	0.855682376	4.32E-13	TRIM38	-0.710125723	2.24E-07
IFI30	0.855373186	5.59E-28	LIN52	-0.710138415	3.36E-07
SHANK3	0.854883883	1.59E-05	ANKRD44	-0.710154617	3.88E-05
MRTO4	0.853684982	8.02E-25	QPCT	-0.710522941	0.0069826
YARS2	0.8524674	4.69E-16	TCEAL3	-0.711145448	1.96E-08
ZNRD2	0.852315435	8.52E-20	BTN2A2	-0.711262726	0.000198
RIOX2	0.851846713	4.35E-17	GOLGA7B	-0.711818042	0.0007818

WDR77	0.850068463	2.29E-29	DOCK8-AS1	-0.712190923	0.0068957
CUTC	0.848665396	4.77E-14	PCDHA11	-0.712310118	1.20E-09
SAPCD2	0.84834677	4.68E-26	HTR7P1	-0.712987651	0.0010706
MPP3	0.848277658	0.000174	GPR39	-0.713839001	0.0327191
DCAF4	0.847639484	2.42E-12	AR	-0.714020771	0.0003581
KLHL18	0.84731955	1.25E-13	SFR1	-0.714370058	0.0057499
FP565260.3	0.847304558	0.000286	ADCY5	-0.714868568	1.07E-08
TRIAP1	0.847275286	2.42E-15	ZCWPW1	-0.714983574	5.85E-05
DANCR	0.847020973	1.19E-25	NHLRC3	-0.715643347	5.30E-07
NOC3L	0.84697543	1.31E-19	SOX4	-0.715743562	2.56E-07
GPR135	0.846015789	0.001403	TSHZ3	-0.715817844	0.0119046
TMEM33	0.844959336	8.14E-16	COL27A1	-0.716047937	0.0003542
WDR4	0.844583933	5.11E-15	ENPP1	-0.716443536	6.49E-13
NOP56	0.84433671	1.03E-35	SLC30A3	-0.716641548	0.0014919
MAST1	0.843960805	0.001134	SDC4	-0.717247921	1.56E-17
PSMG4	0.843597013	1.26E-16	PARP10	-0.717398676	3.57E-11
PEX5	0.842841036	2.75E-15	TMEM164	-0.717664798	0.0370166
GARNL3	0.842789441	0.006639	IRF5	-0.718065972	1.32E-06
CCDC58	0.842514102	6.75E-12	AC008393.1	-0.719590454	0.0412377
FMNL2	0.840894934	2.92E-08	ANLN	-0.719896049	3.09E-10
BBC3	0.840670585	3.37E-08	GM2A	-0.720286779	4.12E-06
DNAH14	0.840560554	2.53E-06	CRABP2	-0.72080155	9.21E-08
C1orf109	0.837826877	2.24E-15	EFNB3	-0.721116098	0.0004949
GALK1	0.835043504	5.47E-28	SPAG4	-0.721360237	0.0008072
GLRX3	0.834388736	1.85E-20	PBX1	-0.72169864	1.20E-23
CDC42EP2	0.833828167	0.008239	AC108488.3	-0.72218	0.0048963
ATP6V0E2-AS1	0.833741535	9.25E-05	ERMAP	-0.722358493	3.73E-06
TIMM17A	0.83321889	1.22E-20	GMPR	-0.722771924	0.0215194
AC018645.3	0.833131685	0.033962	HIPK2	-0.723064168	4.13E-12
WDR43	0.833085867	2.07E-18	STIL	-0.723252578	2.55E-09
SH2B2	0.833073944	1.84E-08	PDGFRL	-0.72378802	3.09E-05
PLK3	0.832045831	8.33E-08	MGLL	-0.725332494	6.84E-05
POLR3E	0.831720816	1.76E-12	C3orf14	-0.725770463	4.14E-08
TXLNG	0.830296209	3.74E-12	SLC44A3	-0.725995101	0.0012912
RBM19	0.830232165	1.02E-12	FAM110C	-0.726009026	9.61E-12
CHN1	0.830225111	1.26E-16	KREMEN1	-0.727051851	8.81E-08
NOLC1	0.830092154	8.82E-23	ID2-AS1	-0.727761124	0.0084823
PINX1_2	0.829547018	9.18E-14	RECK	-0.728016064	0.0157965
IFRD2	0.828556039	2.32E-29	MIR210HG	-0.728531191	0.0069005
DDX21	0.827789718	7.27E-23	NUSAP1	-0.72872668	9.42E-11
SRM	0.827716524	9.03E-32	TES	-0.729067842	1.65E-14
TNFRSF10B	0.827348936	3.88E-14	WDR76	-0.729072794	5.33E-07
TYMSOS	0.826677847	0.001519	TLN2	-0.729117454	6.25E-07
SLC18B1	0.826381025	1.65E-10	ALG10	-0.729277284	6.14E-05
WDR12	0.825827114	7.32E-15	FAT1	-0.729954026	2.65E-09
ZNF598	0.825784068	5.64E-28	MOSPD3	-0.731393176	1.60E-08
NLE1	0.825368958	4.10E-18	MOB3C	-0.732926019	4.51E-06

POP7	0.824712678	7.44E-23	SOWAHC	-0.732954453	4.08E-05
SLC39A4	0.8238293	1.70E-12	TMEM87B	-0.733287029	1.02E-16
SIM2	0.823336862	9.97E-06	TMCO3	-0.733429903	5.35E-10
LINC01534	0.823219688	0.041505	ZNF354C	-0.734049058	0.0036936
TYSND1	0.822984043	2.13E-23	HECW2	-0.734308375	0.0024963
NOB1	0.82295823	4.23E-28	INHBB	-0.734418951	5.19E-14
SLC16A10	0.821770945	0.022309	EMP2	-0.734688596	3.92E-13
RSL24D1	0.821707352	1.98E-18	BARD1	-0.735037909	1.07E-06
UBE3D	0.821303233	3.47E-06	TRIM66	-0.73524893	5.67E-06
EIF5A	0.819959952	3.94E-30	BCAS1	-0.735706402	0.0064702
CISD1	0.818848942	7.20E-11	HLA-DRB1	-0.735864622	1.77E-11
INO80B	0.818691426	4.36E-16	CDK19	-0.73617647	1.86E-15
ADSL	0.818634606	9.46E-23	PHF19	-0.736195806	6.04E-08
BAX	0.817830972	1.32E-23	PAQR4	-0.736343394	2.90E-07
WDR97	0.817652708	1.73E-11	SLC12A6	-0.737280396	9.51E-07
RSAD1	0.814535165	4.81E-15	ZNF862	-0.737321534	1.25E-11
SESN1	0.814430732	6.41E-11	CCNG2	-0.737531836	9.28E-10
TPST2	0.814219825	6.30E-12	LMO2	-0.737939051	0.0124147
FAM136A	0.81373465	4.55E-24	HPS3	-0.738534404	1.58E-18
HSPD1	0.812966122	7.88E-25	IL17RD	-0.739352384	0.0476436
FAM155B	0.812482857	2.90E-10	FAM111A	-0.739612536	6.56E-14
IMP3	0.812253231	8.22E-21	LETM2	-0.739687229	0.0034328
PWP1	0.810669724	9.44E-23	CTAGE4	-0.740165727	0.0001258
IL15RA	0.809352802	2.38E-08	NECTIN2	-0.7413259	1.29E-18
YBX1	0.80839745	8.14E-29	CASP4	-0.741480293	0.0011461
CARD9	0.807304946	5.39E-05	HOXC10	-0.741499697	0.0013514
SLC35F2	0.806878338	4.28E-15	CELSR1	-0.741595234	5.43E-16
FBRSL1	0.806729586	5.47E-22	CLSPN	-0.741652657	3.51E-08
CRACR2A	0.806689201	8.13E-07	KIF18A	-0.741828543	2.22E-08
DCTPP1	0.80501384	1.01E-31	CREB3L4	-0.742234852	4.22E-06
EPB41L4A-AS1	0.804349875	2.87E-13	FAM234B	-0.742968716	2.29E-07
PRKAR1B	0.80415657	8.78E-22	CENPU	-0.743134636	4.98E-07
MTRR	0.804040469	3.29E-11	SRGAP2B	-0.743670248	5.11E-08
KRT8P12	0.803956194	0.012215	NLGN2	-0.743745474	8.59E-15
TMEM63C	0.802819864	1.30E-06	PLD2	-0.744222259	2.33E-11
THUMPD2	0.799708293	1.44E-09	APLP1	-0.744390772	0.0002787
COQ3	0.799072591	1.28E-09	ZNF821	-0.744884417	3.90E-05
PAK1IP1	0.799067891	6.50E-17	KIF13B	-0.745540863	2.91E-11
TAMM41	0.798400553	6.23E-14	FGD1	-0.745796219	0.0493349
COQ10A	0.797906824	1.21E-06	C20orf204	-0.745984652	0.0415269
PM20D2	0.797266592	2.08E-11	CLDN23	-0.746237697	1.80E-05
CTSC	0.796422973	1.74E-13	NT5C2	-0.746295186	1.55E-15
LYRM7	0.796028343	2.84E-08	HDAC5	-0.747513125	6.67E-05
PHLDA3	0.794546353	5.56E-13	GPR158	-0.747659415	0.0044806
CYP2J2	0.793092589	0.030649	TRIOBP	-0.747796207	2.44E-17
NIP7	0.792612422	1.53E-24	CCDC191	-0.747879725	0.0003583
SERF1B	0.792461238	1.50E-15	REXO5	-0.749459717	9.61E-05

ADGRA3	0.791519864	9.46E-12	VAMP5	-0.749758755	0.0011767
GCFC2	0.7913081	2.80E-08	PRRT3	-0.750557206	4.07E-12
DVL1	0.790119102	2.69E-21	PCDH9	-0.751229508	0.0076491
CLPP	0.790110545	1.44E-20	JAK1	-0.751497949	1.63E-16
QSOX2	0.789900454	1.73E-16	CROT	-0.752156346	1.38E-08
TDRD1	0.78977435	5.97E-12	COL18A1	-0.752212623	1.06E-13
RASGEF1C	0.789475995	0.042634	SCN1B	-0.752613665	0.0040377
LINC01106	0.789321286	2.55E-06	MAP3K12	-0.75327179	1.91E-09
SNHG15	0.788860336	1.92E-08	ZC2HC1A	-0.754089878	4.49E-06
GTPBP3	0.78881422	4.91E-15	ATP2C2	-0.754405905	9.62E-11
ELOF1	0.787770565	3.24E-26	THNSL2	-0.75449044	1.77E-10
ZMAT3	0.787151438	3.31E-09	NR4A1	-0.754665898	8.79E-09
TBRG4	0.786986948	4.34E-19	TMPRSS13	-0.754876788	3.80E-08
TTLL12	0.786675821	2.06E-20	KRT87P	-0.75503181	0.0044064
SLC25A27	0.786032874	0.021499	KANK2	-0.75533781	6.58E-17
SLC9A3	0.785372978	1.02E-05	C21orf58	-0.75536086	4.36E-05
RSL1D1	0.785170974	1.38E-19	LASP1	-0.75549567	2.11E-24
THAP11	0.785129722	3.25E-14	CORO2A	-0.755602735	9.52E-09
PRRT1	0.784568012	0.039007	MIEF2	-0.756152702	5.11E-10
ZNF778	0.783277939	9.49E-16	AC023158.1	-0.756427766	0.0001377
HHEX	0.783259616	0.003149	ERICD	-0.756785536	0.000381
AHSA1	0.782343057	1.26E-24	TMEM107	-0.756893294	0.0019751
DIAPH2	0.78101633	1.58E-10	SDCBP2	-0.757269992	0.0136492
C16orf91	0.780987738	2.76E-12	GNAO1	-0.757316596	0.0468716
ASIC1	0.780062654	1.90E-06	AC112220.2	-0.757679899	6.95E-05
PDP2	0.77978593	1.08E-07	CCN2	-0.757725781	0.0326104
TUBE1	0.779477529	1.36E-06	ENPP5	-0.758018142	4.19E-05
C5orf30	0.779080036	1.13E-10	HLA-DQB1	-0.758280576	1.26E-08
AK2	0.778913463	1.97E-22	RBMS2	-0.759032541	3.23E-06
MAPT	0.778613092	7.88E-08	AC026748.3	-0.759104269	0.0002551
DFFB	0.777973642	5.46E-05	FAM189A2	-0.759256509	0.0008218
NOTCH1	0.777742367	5.89E-15	SEMA4A	-0.759433083	1.10E-08
ACOXL	0.776337769	0.012413	CD24	-0.759642507	1.87E-14
MGST1	0.774724894	2.99E-12	CCNE2	-0.7602986	1.27E-07
CDC123	0.77447893	1.09E-18	PSMG3-AS1	-0.760398434	5.74E-17
TSR3	0.774358795	6.24E-25	LOXL1-AS1	-0.760811691	0.0006345
GNAI1	0.773620137	0.001158	CHROMR	-0.760848209	0.0014533
CGREF1	0.773251	1.99E-08	CYP4V2	-0.760934054	5.89E-06
FOXN3-AS1	0.772463323	0.038468	KDELR3	-0.763056805	7.93E-06
AK6	0.77213731	1.24E-12	MAP4K2	-0.763857607	2.72E-09
PCSK6	0.771792749	4.08E-08	IQGAP1	-0.764131552	1.11E-21
KLHL23	0.770924951	2.90E-06	LRRC49	-0.764295766	0.0475331
TMCO6	0.770644778	7.86E-08	ITPR2	-0.764375545	7.12E-11
AC083899.1	0.769273101	0.019817	ASPM	-0.764521464	9.51E-10
TMEM161A	0.769082796	7.95E-14	ZNF713	-0.764534632	0.004842
BOD1	0.768877763	1.65E-18	NBEA	-0.765134593	3.98E-08
CLUH	0.768391889	2.52E-18	TRPS1	-0.765389307	4.09E-12

AC107871.1	0.768134741	4.40E-06	AP005329.2	-0.765569288	0.0415066
PNPT1	0.76686328	8.07E-16	FAM102A	-0.765705534	4.32E-11
GTF2F2	0.76611385	1.92E-13	CARF	-0.766017683	0.0031248
ASS1	0.76510058	9.42E-14	RHOB	-0.766426389	9.97E-18
ZNF511	0.764628898	9.88E-12	NINJ2-AS1	-0.76688834	0.0043813
NT5DC2	0.764486205	2.14E-21	C14orf132	-0.767394025	0.0082574
TMEM177	0.764479315	7.78E-10	FAM111B	-0.768466008	9.47E-09
MHENCR	0.764355828	0.00375	PRX	-0.768914933	0.0122767
AC010655.4	0.764253077	0.018459	TTC25	-0.769458838	0.0242557
IKZF5	0.763755865	1.07E-10	L1CAM	-0.769520827	0.0088719
RAB29	0.76311675	3.24E-13	EPHA1	-0.769912188	7.17E-12
DDX49	0.762122347	2.22E-25	CAPG	-0.770372787	7.75E-08
LAPTM4B	0.761673086	3.99E-17	KATNAL1	-0.77098266	0.0001903
C12orf29	0.761573012	5.11E-10	LAMB2	-0.770997441	1.31E-22
C12orf45	0.761178433	1.38E-09	GABARAPL1	-0.77145285	0.0198167
BCS1L	0.761104688	1.85E-21	FAM43A	-0.771676204	0.0008762
PTRH2	0.76083793	3.63E-18	CBLN3	-0.771766574	0.0475816
ISCU	0.760717529	5.57E-20	CABLES2	-0.771889695	1.93E-10
HOMER1	0.760657525	8.89E-12	IPP	-0.771892641	1.68E-06
ISOC2	0.760096229	3.00E-17	N4BP2L1	-0.77201325	0.0138647
RUNDC3B	0.759599234	0.013262	XBP1	-0.774564949	0.0228105
MRPL34	0.759379865	6.76E-16	AC010186.3	-0.775160544	0.0241445
KAZN	0.759341681	4.60E-11	C19orf33	-0.77541193	4.05E-07
CCDC78	0.75915151	1.43E-14	SRCIN1	-0.775940598	1.18E-08
SLC25A15	0.758988202	1.54E-20	SLC25A24	-0.776041699	2.33E-13
PRMT5	0.758239577	5.28E-22	LTC4S	-0.77642082	0.0385864
SLC4A2	0.75758895	6.51E-19	BRCA1	-0.776919877	5.56E-10
RHBDF2	0.757516437	7.42E-14	CPQ	-0.777277554	0.0152633
FBXO25	0.75699782	6.62E-11	KIAA1324	-0.777347214	1.50E-08
SMPD2	0.756957738	1.58E-12	TEAD1	-0.777708026	3.59E-14
ADM5	0.756671085	6.58E-05	AHNAK	-0.778188284	1.53E-21
FAH	0.756479323	2.85E-10	RAB27B	-0.778648659	7.69E-20
COA4	0.756433162	8.55E-17	LDLRAD4	-0.779249225	8.95E-10
NUFIP1	0.756410352	2.04E-08	ZNF217	-0.779979022	2.75E-21
POLR2F	0.755265458	5.00E-17	TMBIM1	-0.780052636	0.0011396
BEX2	0.754985207	0.017987	GSTM4	-0.780716826	0.0120055
AC068547.1	0.754929807	4.10E-09	RUNDC3A-AS1	-0.781052158	2.99E-07
TOMM40	0.754712437	2.40E-26	PROS1	-0.781242442	0.0268717
MRPL14	0.754236635	1.75E-21	RNF213	-0.781428806	3.83E-14
CHORDC1	0.75390445	9.00E-15	SH3D21	-0.781737789	7.32E-12
DYRK3	0.753268975	3.31E-08	PRKD1	-0.781900799	0.0004016
BEND3	0.752800998	5.73E-08	SHISA2	-0.782216336	0.043441
PRDX4	0.752453289	1.92E-16	PSMB9	-0.782438165	0.0007648
AK4	0.751542539	5.32E-09	WWC3	-0.782991986	6.26E-17
PTDSS1	0.751467694	1.96E-14	CLSTN3	-0.783202584	6.41E-08
TMC8	0.751091587	0.014274	NUCB2	-0.784452925	7.95E-07
LINC01123	0.750812022	0.001152	TLCD4	-0.785082808	2.19E-07

MEST	0.750810485	3.38E-16	MAGED4	-0.788225026	0.0188605
TIMM21	0.749560011	3.02E-12	TMEM198	-0.788443793	0.0048987
TOMM34	0.748941323	2.40E-13	DAGLA	-0.788924048	8.31E-06
PMPCA	0.748807721	2.18E-21	FER	-0.789017061	3.49E-08
AC019069.1	0.748795978	4.64E-06	PAQR8	-0.789209285	0.0178075
GEMIN4	0.748777854	3.25E-18	EFCAB11	-0.789685706	5.73E-08
CCDC59	0.748685718	2.79E-15	CLGN	-0.791521576	0.0384677
PPTC7	0.748438328	2.91E-09	NDC80	-0.791921763	1.70E-06
SACS	0.748345357	9.17E-08	KNL1	-0.791977996	2.07E-08
AIMP2	0.747920667	8.84E-18	SLC24A1	-0.792013955	1.66E-10
IMMP2L	0.747521624	1.42E-06	MAN1C1	-0.792131721	0.0232267
WNK2	0.747354634	1.17E-06	SYNE2	-0.792352295	3.56E-19
LSM7	0.747318388	3.55E-19	VIPR1	-0.792361484	0.0001237
PUM3	0.746722507	1.65E-18	RAB4B	-0.792498789	8.46E-06
MRPS23	0.746513516	1.61E-20	EFCAB6	-0.792984693	0.0156376
BRIX1	0.746140487	1.17E-18	CNTRL	-0.793666053	1.40E-05
CAMKMT	0.745468838	4.49E-05	AKAP5	-0.793866108	0.012323
MAX	0.744619848	2.70E-19	PHLDA1	-0.793877975	1.79E-08
BMP7	0.7443619	2.46E-13	MAGED1	-0.794185778	4.41E-17
RRS1	0.74409349	3.29E-17	KLC3	-0.794299736	0.0011933
METTL26	0.742820023	1.14E-17	DOCK8	-0.794580787	3.24E-08
NCLN	0.742403124	3.88E-23	EPB41L1	-0.79494848	2.57E-10
TRABD	0.74172081	3.22E-20	BMP8B	-0.795049554	2.71E-05
MRPS5	0.741670412	1.32E-11	TCF19	-0.795149007	1.09E-09
RNF126	0.740761389	1.42E-18	BHLHE41	-0.795436174	0.0223846
PRR7	0.740598107	1.30E-11	ZNF311	-0.796036153	0.0333859
AC125807.2	0.73992316	5.94E-06	KRT81	-0.796091696	1.14E-31
SLC39A14	0.739608062	1.23E-16	MAGED2	-0.796273935	4.31E-14
CTPS1	0.738903653	2.17E-26	AL358852.1	-0.796602594	0.0486052
PACC1	0.738782446	9.66E-07	EDN1	-0.797023591	0.0015215
RPS19BP1	0.738629418	1.44E-21	NFKBIZ	-0.797130813	7.07E-19
TMEM102	0.738434235	3.19E-08	MCOLN3	-0.798278983	0.0043714
POLR1E	0.737788614	3.60E-11	FRMD4B	-0.798312248	1.66E-05
AC021087.5	0.736951076	0.008232	EPB41L2	-0.798622908	5.25E-09
APTR	0.736230702	6.06E-07	CAPN9	-0.799390325	0.0028667
UBALD1	0.735034141	2.24E-15	ULK1	-0.799546027	3.40E-20
ZDHHC9	0.734901547	1.43E-13	TFPI	-0.799980709	2.19E-16
TXNL4A	0.734628698	1.44E-18	H6PD	-0.8001881	1.87E-10
TRIM65	0.734506699	6.24E-23	CDKN2C	-0.800212919	2.03E-05
PCGF1	0.73421949	1.92E-10	SYT12	-0.80074375	0.0083036
AL356740.1	0.733860016	0.035601	AC138028.6	-0.801562305	0.0141336
PPP2R3B	0.733858832	1.15E-12	MCC	-0.802062225	7.79E-05
ZPR1	0.733807371	2.74E-17	SLC12A5	-0.802651386	2.56E-07
MRPS24	0.733537952	1.58E-14	N4BP2	-0.803698948	3.41E-09
SNHG26	0.733516243	9.52E-05	RIN2	-0.803753978	4.05E-06
TIMM22	0.732865486	1.97E-13	SLC22A18	-0.803888825	7.35E-07
DESI1	0.732742005	3.33E-13	TOP2A	-0.806319635	9.25E-12

MRPL3	0.732062462	2.44E-19	DIPK1B	-0.806417489	5.25E-05
RBM28	0.731999696	1.33E-16	IQCD	-0.806708556	0.008763
UTP20	0.731523858	2.51E-10	CACFD1	-0.80694733	4.15E-15
WDR81	0.731381744	1.54E-11	SMAD3	-0.809573931	0.0014697
RXYLT1	0.73024384	3.93E-11	FRG1BP	-0.810090129	9.61E-10
MAPKAPK5-AS1	0.730168439	8.28E-12	KIAA1217	-0.810156179	1.15E-08
SCARB1	0.729360865	7.46E-12	FAM153CP	-0.810340361	0.0160494
NARS2	0.729065905	1.41E-10	ZBTB46	-0.810420111	1.91E-06
ABLIM1	0.728960991	9.33E-09	FAM161B	-0.810721476	1.45E-05
MLST8	0.728891023	2.29E-15	AC093512.2	-0.811026646	1.16E-05
B3GALT6	0.728363838	5.24E-15	BBOF1	-0.811433515	3.48E-06
WASF3	0.728257541	1.42E-06	HR	-0.811779022	4.30E-06
SNHG9	0.728112003	0.018861	ZSCAN30	-0.811945586	2.97E-06
ITPA	0.727874969	6.09E-18	SORBS3	-0.812598044	9.91E-12
HMOX2	0.726912499	1.29E-20	REEP1	-0.812842438	0.0028667
TMEM201	0.726477685	1.74E-13	IGFBP5	-0.813177538	1.13E-21
MTHFD2	0.725848914	9.21E-21	TMEM79	-0.81444793	7.31E-07
DDX31	0.72582628	5.62E-15	LRRC37A3	-0.814860802	7.79E-07
TCOF1	0.725388912	2.35E-19	RALY-AS1	-0.815805551	0.0004816
PMAIP1	0.725200653	0.00019	AC055811.4	-0.816180975	0.0045509
MIF	0.724961595	1.23E-15	RORA	-0.816254187	0.0011191
CRLS1	0.724304087	1.76E-09	SAMD15	-0.816279086	1.55E-06
SSBP4	0.724282386	4.55E-17	TAPBPL	-0.816747109	3.10E-05
PCCA-DT	0.724226982	0.000152	DSCAM	-0.816885911	0.005104
URB1	0.724098095	6.83E-11	ZFP36L2	-0.816982887	8.14E-29
FMC1	0.723872524	3.34E-09	SLC7A2	-0.817421834	1.25E-13
NOP14	0.723662967	7.41E-16	EIF4E3	-0.817623263	4.21E-11
INTS13	0.723266743	4.67E-14	OR2A9P	-0.818376606	0.0125287
RCL1	0.722711317	5.80E-14	NBPF4	-0.819393742	6.77E-05
UNKL	0.722683142	1.79E-13	AC068580.4	-0.819500948	0.001325
SLC12A8	0.722520777	1.07E-05	CRAT	-0.819678149	0.0395656
ATP1B3	0.722514543	1.30E-11	OLFM1	-0.820117406	1.57E-10
MAK16	0.722411976	5.53E-12	ZGRF1	-0.820659529	2.81E-08
AMPD2	0.722174886	4.74E-11	IL1RAP	-0.820776173	1.66E-05
ISCA1	0.721569822	6.44E-14	GPC1	-0.820904071	3.30E-13
CYC1	0.721341021	7.38E-23	OCEL1	-0.822623445	6.16E-07
TARS3	0.721222248	1.33E-07	KLF2	-0.822750729	0.0203546
KAT2A	0.721027381	2.21E-15	KRT19	-0.822796741	3.19E-10
HYAL3	0.720451227	0.001061	MICB	-0.822809622	0.0002928
NTMT1	0.719790894	1.58E-15	TINCR	-0.822846994	3.11E-07
POLR2I	0.719654542	2.88E-12	PRR15	-0.823086909	3.38E-05
SLC25A26	0.71906046	3.56E-10	AP001816.1	-0.823385998	9.22E-06
SNU13	0.718175891	6.15E-24	PMEL	-0.82447646	0.0034885
TANGO6	0.717984547	7.23E-09	KLF8	-0.825074631	0.0073373
UBASH3B	0.717874503	0.019764	IGF1R	-0.827073657	1.03E-35
MRPS25	0.717502139	1.45E-21	ALPK1	-0.827094916	8.47E-05
GLB1L2	0.717471208	7.95E-13	ULBP2	-0.827315538	3.76E-10

ATAD3A	0.716697349	2.84E-24	FOXP3	-0.827658927	0.0429899
SLC7A11	0.71659578	1.32E-07	MKI67	-0.828240271	5.11E-16
QTRT1	0.716432549	3.37E-17	C1orf115	-0.828443958	5.21E-08
TOMM22	0.716023856	4.09E-12	GOLGB1	-0.828618355	2.59E-23
ZNF639	0.71568147	5.57E-17	SYT1	-0.828773803	1.33E-05
ID3	0.715414405	1.29E-09	ERV3-1	-0.830067439	5.45E-06
DDX51	0.714751813	7.76E-16	FBXO27	-0.830297327	0.0436209
NEK6	0.714573598	1.37E-09	ST8SIA6	-0.830371289	4.07E-05
GCSH	0.714223469	2.36E-15	SMIM14	-0.830445384	1.09E-17
ESF1	0.71403578	8.94E-11	KRT8	-0.830974785	2.90E-11
CAB39L	0.713217611	4.52E-05	SENP7	-0.831543976	5.33E-10
GADD45GIP1	0.712957851	9.41E-16	ZNF362	-0.832100114	4.43E-10
XPO5	0.712935662	1.08E-13	EHBP1L1	-0.832874724	5.36E-13
RPF2	0.712189723	1.56E-11	MRPL23-AS1	-0.833179156	0.0010694
STK26	0.711637875	3.26E-12	RASSF8	-0.833450843	1.62E-07
EFHD2	0.711390733	3.54E-15	TCAF1	-0.833933438	5.66E-17
SCLY	0.71127163	6.76E-14	ADGRB2	-0.838749822	0.0098531
JMJD6	0.711221049	1.09E-12	KIAA0513	-0.839432715	9.37E-15
NT5DC3	0.710969307	4.68E-06	KRT80	-0.841125233	1.33E-11
MRPS34	0.710914449	7.61E-19	EPOR	-0.841700337	0.000379
TIMM50	0.710650923	1.13E-21	TK2	-0.843496944	8.30E-06
PEX10	0.709837364	2.56E-09	LIPH	-0.844258474	0.0011276
MRPL15	0.709649366	7.35E-16	CDK14	-0.844736495	0.0067762
SF3B5	0.709404237	2.47E-22	MYO7A	-0.844846229	0.0324271
CLNS1A	0.70882362	5.32E-12	OSBPL5	-0.845254738	6.76E-08
TSFM	0.7086269	2.60E-10	RASA4CP	-0.845370874	0.0147179
MRPL1	0.70858082	1.08E-12	MAF	-0.845445628	0.0065056
TUFM	0.708448525	1.88E-17	ABHD2	-0.845566574	0.0412739
FAM3C2	0.708103799	0.00366	ACSS3	-0.845941978	2.10E-08
TRMT10C	0.707820419	6.32E-11	AC006372.1	-0.845948427	0.0146324
ADAM11	0.706700862	0.000607	AGBL2	-0.846213098	0.0124754
NPM1	0.705823412	4.81E-15	ESPN	-0.847306667	6.80E-07
XPOT	0.705696875	1.10E-17	TNFAIP2	-0.847533217	0.0384402
POLR3D	0.70485217	2.48E-14	TTC21A	-0.847674006	0.0166096
NUDT19	0.704032669	1.31E-11	FUT8-AS1	-0.8478552	0.006042
PRMT3	0.703564018	6.33E-13	PLXND1	-0.848386054	1.45E-17
SRXN1	0.702662886	1.75E-13	PLA2G4F	-0.848521801	8.99E-05
GNL3	0.70220793	1.59E-16	TRIM62	-0.84913803	1.76E-12
B4GALT3	0.702106792	1.56E-17	GATA3	-0.849330888	1.63E-20
PAXIP1-AS1	0.702055693	6.38E-12	ITGA7	-0.850492855	0.0156913
ZNF330	0.701890894	4.27E-10	WLS	-0.850722109	0.0011032
GTF2H2	0.701819454	4.41E-12	KLHDC9	-0.85099221	0.0242706
ARHGAP39	0.70121393	1.74E-14	AL137003.1	-0.851052359	0.0470462
AC022966.1	0.700968592	1.85E-13	FRMD5	-0.852653075	0.0141318
LOXL3	0.700845268	5.97E-05	PRSS27	-0.852807511	0.017591
ERO1B	0.70063212	0.014082	CAMK2N2	-0.854062979	4.09E-06
NFIA	0.700576152	0.00022	SMPDL3A	-0.854636786	4.00E-07

NOCT	0.700515247	3.59E-07	GLB1L	-0.855051022	9.21E-05
FAM207A	0.700109356	8.30E-14	TMPRSS4	-0.855289668	0.0063721
AL358472.6	0.699769761	2.83E-05	KNDC1	-0.855329943	0.0007302
THOP1	0.699559444	4.17E-18	EMP1	-0.855547293	0.0055263
POP1	0.699137021	8.59E-16	KRTAP5-AS1	-0.856069758	1.35E-06
TMEM186	0.699022454	1.16E-09	AC139769.1	-0.85630836	0.0001489
B4GALT2	0.698842153	4.95E-16	SNN	-0.856573424	3.64E-07
HTRA2	0.698361461	7.05E-14	TMEM229B	-0.857196212	4.26E-08
ACTR3B	0.698136203	2.05E-13	RALGPS1	-0.857523203	6.53E-08
TMEM70	0.697185861	1.90E-14	ZNF658B	-0.85782226	0.0397839
VASH1-AS1	0.697124307	2.07E-05	NUTM2D	-0.858198591	5.56E-05
NOP2	0.696771759	1.84E-17	PLK2	-0.860064411	1.51E-25
DHODH	0.69558877	8.69E-11	FAM66C	-0.860577824	0.0020873
SLC7A6	0.695270501	3.94E-07	CU633904.2	-0.860860857	0.025038
ALG3	0.695180147	1.15E-16	HJURP	-0.861419982	8.15E-10
BYSL	0.694996976	1.88E-15	TMPRSS3	-0.862478098	0.0377129
LRIG3	0.693712831	0.000516	PLEKHA2	-0.863306236	3.14E-10
FAS	0.693315253	4.97E-09	BX322234.1	-0.86566341	0.0485586
MARC1	0.692783863	5.53E-07	BAIAP3	-0.866155169	4.62E-05
NDUFAB1	0.6927521	4.35E-15	HHAT	-0.866944444	1.98E-05
SMIM4	0.692497023	0.000168	AC104452.1	-0.867158567	0.0102589
TRMU	0.692237585	4.39E-13	SUCLG2-AS1	-0.867219501	0.0282116
NETO2	0.691729677	1.72E-16	RAB8B	-0.867279048	1.93E-10
CU633906.7	0.69157107	9.61E-05	CLU	-0.867302924	2.05E-19
PFAS	0.691283385	6.02E-12	SLC22A15	-0.867328255	5.25E-08
POLRMT	0.690461775	7.30E-15	PRODH	-0.86988568	0.018116
MPLKIP	0.69038057	2.53E-11	MTMR7	-0.870011155	0.0096972
TUBGCP4	0.688646309	2.08E-08	CCDC153	-0.870601697	0.0024667
GTF3A	0.688090824	6.41E-22	HDAC6	-0.870664701	3.23E-16
RAB17	0.688034802	1.62E-09	SELENBP1	-0.872450692	0.002947
CSPG5	0.687999717	0.00457	ETHE1	-0.87268505	4.35E-05
MRPL52	0.687528461	2.08E-10	CCM2L	-0.872783801	0.0110675
PDCD11	0.687488614	1.83E-13	CRYBG3	-0.873738484	0.0003015
NPIPB12	0.687094751	1.66E-06	KIF24	-0.874029984	6.27E-07
CHAC2	0.686912073	1.86E-08	COL5A2	-0.874239401	3.76E-07
URI1	0.686833632	3.33E-15	DSC2	-0.87472035	2.09E-17
SIX1	0.686692722	2.16E-06	TET1	-0.87481619	0.0389591
CEBPB	0.686613922	1.69E-11	TRPV4	-0.875206511	1.67E-06
EIF3J	0.685965072	4.22E-21	PACSIN1	-0.875213411	3.22E-10
AC097448.1	0.685041089	0.000384	NOS1AP	-0.875282312	0.0003615
SNAPC4	0.684941511	1.51E-12	ARHGEF10L	-0.875826478	7.92E-11
UTP25	0.68424474	8.44E-11	ERICH6-AS1	-0.876583873	0.0317528
ATP5MC1	0.684110699	2.21E-19	GDPD3	-0.877508888	1.25E-08
AL441992.3	0.683992796	0.001752	STX1B	-0.87792875	0.0122421
GALK2	0.683481518	4.51E-07	NEMP2	-0.878232548	2.95E-07
ELAC2	0.683388069	1.89E-17	HMGB2	-0.878323959	0.0083677
SNHG17	0.683138322	1.62E-09	ARHGAP29	-0.879046476	5.18E-11

CDK5R1	0.681780923	0.000984	TIGD7	-0.879119005	0.005432
BZW2	0.681007235	5.11E-16	SYCP2	-0.879471632	1.48E-15
CLCN2	0.680983764	3.78E-05	ADD3	-0.881333177	0.0001453
AGPAT5	0.680734456	1.54E-15	EPPK1	-0.881376938	9.87E-22
EMC8	0.678289436	9.94E-15	SLC8B1	-0.882137201	3.81E-06
ANKRD27	0.678127972	1.99E-09	PLAAT3	-0.882889275	8.37E-09
NDUFB9	0.677939916	2.80E-18	AC126564.1	-0.883052711	6.41E-08
CFAP97	0.677754028	2.17E-09	RAB32	-0.88390017	5.22E-12
EBNA1BP2	0.677350859	4.21E-19	SH3TC1	-0.884141133	3.23E-05
USP2	0.677254212	0.000575	BMERB1	-0.884930487	7.92E-06
PHKA1	0.677046029	9.55E-09	PLA2G10	-0.885254765	0.0104721
PROCR	0.676616358	0.000291	CYLD	-0.885381676	1.79E-14
RN7SL3	0.676463492	0.004307	KRT18	-0.885798912	3.30E-13
PRELID1	0.67608741	2.64E-23	SLC66A3	-0.88616191	5.51E-11
BRAT1	0.675717574	2.22E-16	LINC01137	-0.886169249	0.0037691
NOL10	0.675240472	1.06E-13	KAAG1	-0.88669885	0.0303421
FLVCR1	0.6749699	8.48E-06	F2R	-0.887500389	0.0001382
MRPS27	0.674613443	1.92E-13	MAP1B	-0.88767664	0.0164858
RRAS2	0.674305627	3.09E-14	CLDN9	-0.888013448	2.85E-08
EIF3B	0.673748137	1.97E-17	PCOLCE	-0.888057094	1.66E-06
NCL	0.673421576	7.95E-20	TRIM29	-0.888585056	0.0071131
RAPGEF5	0.672851738	0.008279	H1-0	-0.888817474	8.98E-05
MRPL32	0.672802009	1.91E-17	RALGPS2	-0.888841543	5.89E-09
ZNF276	0.672144068	2.26E-08	MBOAT2	-0.889877406	4.75E-18
TMEM147	0.671519083	9.33E-17	SEMA3F	-0.89008099	4.32E-11
KREMEN2	0.670728536	0.000401	HDAC11	-0.890815703	3.46E-09
PYCR1	0.670698532	6.95E-17	NAIPP2	-0.891245896	0.0013709
POLG2	0.67021337	1.36E-10	CTSD	-0.891417845	0.0469393
COA7	0.669837126	8.57E-14	GYG2	-0.891963704	0.0195031
NOC2L	0.669762025	1.63E-21	ACSS2	-0.892497759	5.58E-13
PRPS1	0.669488054	4.72E-11	DISP1	-0.893548194	0.0004502
AL121832.2	0.668469931	0.013652	ZG16B	-0.894669879	4.61E-08
SGTA	0.667934705	1.97E-18	SRR	-0.89487736	3.11E-07
CDR2L	0.667498056	6.72E-14	ARNTL	-0.895944126	3.22E-07
FOXO3B	0.667079063	0.000592	VWA5B2	-0.896713207	0.0254876
ABCF2_2	0.666965133	8.28E-23	CTDSP2	-0.896935845	1.18E-19
ATAD3B	0.66666867	2.08E-12	SLC16A13	-0.896955648	5.83E-06
ZNF749	0.666552031	0.000412	ATP6V0A4	-0.897021095	6.03E-11
ATP6V1E2	0.666085092	1.12E-05	ZNF365	-0.897661867	5.37E-05
ATP5F1D	0.665306144	1.18E-22	MAPK11	-0.898026638	2.29E-12
METTL5	0.664930418	3.70E-10	RAP1GAP	-0.898051482	7.92E-14
ABHD14A-ACY1	0.664607055	0.039628	ZNF678	-0.900411817	5.01E-06
DOCK4	0.664547002	0.016784	SCNN1A	-0.900415138	6.38E-06
DUSP14	0.663981142	7.92E-11	IFNLR1	-0.901559078	0.0489191
GID4	0.66392209	0.000177	NPHP3	-0.902946301	1.36E-10
FBXO31	0.663868933	2.42E-11	RRAS	-0.903100097	1.61E-11
ZNF584	0.661225778	5.20E-07	AC021066.1	-0.903274744	4.36E-22

MRRF	0.661111175	6.75E-14	POLD4	-0.904907727	3.60E-18
PDK1	0.660640643	8.88E-06	MICAL1	-0.905778189	1.42E-14
AC004980.1	0.659746848	0.029072	UNC13D	-0.907601799	1.17E-18
GATAD2A	0.659508825	1.99E-12	AL136295.1	-0.909047367	0.0394381
NDUFAF5	0.659111805	3.76E-07	PAQR7	-0.909192695	3.47E-06
PFDN4	0.659097177	3.00E-11	RHOU	-0.90929683	2.83E-11
SLC16A1-AS1	0.659020958	0.044182	ABLIM2	-0.909604151	0.0092706
RWDD4	0.658931064	1.13E-11	LINC01503	-0.909727904	0.0016431
KLF16	0.658858827	7.50E-13	MDGA2	-0.909965801	0.0055185
DNAJA3	0.658582367	2.25E-16	ZNF610	-0.910093358	0.0262654
POLR1D	0.658422768	1.10E-10	LNCAROD	-0.910545541	0.0051672
DNPH1	0.65831882	7.78E-12	C4orf33	-0.911645437	0.0009476
MEMO1	0.657908871	4.40E-09	DENND3	-0.911820867	1.34E-09
FBXO45	0.657902572	3.18E-20	SFXN5	-0.913262706	8.16E-10
GCAT	0.657832537	2.25E-07	PYROXD2	-0.914291775	1.18E-08
TEAD4	0.656728917	1.08E-09	SYNGR3	-0.914634289	1.73E-07
SAC3D1	0.656606381	9.73E-14	FOS	-0.914921929	4.71E-19
ABCE1	0.656321529	1.04E-14	TUBB3	-0.914958875	6.86E-17
WDR90	0.656271997	8.96E-05	MACF1	-0.915528596	2.86E-11
SEPTIN6	0.656041382	0.044072	PBLD	-0.91671231	5.82E-07
ENDOG	0.655701843	1.73E-13	TRGV9	-0.917281499	0.0014468
TSPO	0.655428689	1.18E-13	RGS12	-0.918459974	6.34E-07
ANGEL1	0.65520227	2.43E-07	DOCK11	-0.918619079	3.86E-09
MRPL21	0.654837279	2.34E-10	MIR9-3HG	-0.919082037	8.86E-05
CDK20	0.654433141	6.65E-10	MUC20	-0.919906952	5.00E-10
RALGDS	0.654201371	2.39E-10	MYOF	-0.920522598	5.37E-18
LONP1	0.653280701	2.21E-14	CCHCR1	-0.920790044	1.13E-05
GAR1	0.652878388	3.68E-09	ARHGEF37	-0.921883201	0.0037704
SETD6	0.652604186	1.35E-09	CLEC2D	-0.922058769	8.10E-05
GTF2H2B	0.652131425	0.002814	AL590822.3	-0.922840717	0.0301866
NUDT4P2	0.651913667	0.035694	CEACAM6	-0.92298567	1.98E-19
TSEN2	0.651380081	8.69E-11	MATN2	-0.923459191	3.04E-11
TBC1D14	0.651254097	6.79E-10	GPR137C	-0.923507575	1.53E-05
ABT1	0.65103584	2.85E-14	CLTCL1	-0.926037949	2.12E-07
TRIB1	0.650944105	5.35E-06	USH1G	-0.926260551	0.0006889
MTHFD1L	0.65071439	2.05E-19	ZNF879	-0.926876535	0.0076357
COX5A	0.650649574	2.64E-17	GNA14	-0.92737326	0.0281891
CCDC124	0.650196008	7.61E-16	TMEM8B	-0.927782979	0.0001469
AL034430.1	0.649919923	0.011925	SPATA6	-0.930813512	0.0034154
GPR63	0.649370542	0.018067	TRIM45	-0.931677477	5.65E-13
RTKN	0.649309382	1.45E-12	SYNE3	-0.931786548	0.0058224
SPG21	0.648995732	5.64E-14	ZNF66	-0.932026649	0.0020138
MFSD12	0.648231342	4.98E-12	CCDC170	-0.932656326	3.75E-11
FDXR	0.64819946	1.23E-10	TTC39B	-0.932873093	0.0205114
WDR46	0.646896949	1.90E-18	AL022069.3	-0.933128369	0.006951
CBWD6	0.64677294	2.27E-05	SRRM2-AS1	-0.933612829	0.0001783
SLC9A3R2	0.646583552	3.26E-11	AC016705.2	-0.933663847	0.0353214

DBP	0.646451045	0.003532	PCDHGB5	-0.934106057	3.49E-14
PPM1F	0.646426909	3.36E-14	EXT1	-0.934212915	4.28E-08
IFRD1	0.64642446	4.95E-20	RFTN2	-0.934291224	0.0019642
DEGS1	0.645764706	2.50E-10	GABRP	-0.934404822	0.01123
PRTG	0.645188119	0.000258	LOXL1	-0.936761445	0.0246641
KTI12	0.645121475	1.11E-07	CAPS	-0.936867875	0.0040866
BOLA2-SMG1P6	0.644979318	1.30E-12	PTK2B	-0.937159796	2.74E-07
GART	0.644711672	2.46E-13	Z99129.4	-0.938190652	0.0118253
SPR	0.644711386	3.33E-18	TMEM154	-0.938600614	0.0014534
SPATA5L1	0.644622803	2.74E-07	CYP4F11	-0.939436575	0.001459
ZNF787	0.644233578	3.09E-05	ZNF211	-0.939642423	0.0001106
UTP15	0.644080427	1.37E-13	ADSS1	-0.93991753	1.62E-08
SRRD	0.643906165	1.67E-09	C4A	-0.940440177	0.0035477
EFNA3	0.64374475	0.000715	VPS9D1	-0.941278814	5.60E-10
HAUS7	0.643499234	1.38E-10	SEPTIN10	-0.942009584	2.54E-07
TMEM243	0.642825808	1.42E-15	SAP30L-AS1	-0.942124501	0.0058494
ATP11C	0.642820712	6.54E-09	DDX60L	-0.943190493	0.004228
CA5BP1	0.642633187	7.00E-05	DNAH7	-0.944508999	0.0170941
IL27RA	0.642481649	1.50E-05	ZNF709	-0.947301128	0.014345
ACTR5	0.642050228	6.63E-06	TENT5A	-0.947384787	4.11E-09
ATP5F1B	0.64191339	2.39E-20	ALOX15	-0.948201042	4.62E-10
PFDN6	0.641791284	1.89E-12	CTSO	-0.948951539	3.09E-05
FCHO1	0.641331844	8.21E-09	RNF213-AS1	-0.950009908	0.0380117
CDV3	0.64124548	1.84E-18	HIPK1-AS1	-0.952644675	0.0231441
ALG1	0.640829485	1.97E-17	CIT	-0.952765466	1.31E-11
NUS1	0.640765319	4.40E-11	AC144450.1	-0.955166959	0.0040746
NAT9	0.640629453	6.67E-09	SLC22A17	-0.955478859	4.49E-05
MRPL16	0.640419406	9.13E-11	NRP1	-0.957505542	5.49E-19
PPAT	0.639516127	1.31E-17	PLTP	-0.95784707	0.0069826
DFFA	0.639492809	6.20E-13	RNFT1	-0.959073278	3.03E-06
TLCD1	0.639492337	2.81E-07	NAALADL2	-0.95911695	0.0391241
PPID	0.639347234	1.49E-09	DPYSL2	-0.959151972	6.81E-09
SEH1L	0.639149106	1.52E-14	S1PR3	-0.960313696	3.38E-15
MICOS13	0.638780059	4.22E-10	BRIP1	-0.961091602	4.82E-18
PPA1	0.638014656	3.97E-13	RNFT2	-0.961773411	6.58E-07
FAM210A	0.637527614	1.34E-07	RTN2	-0.962521319	0.0042487
DHX33	0.637526663	9.55E-10	LNCOC1	-0.962686561	0.0108158
CENPX	0.637094614	3.45E-20	FLNA	-0.962712571	4.11E-34
SURF6	0.636152866	6.69E-12	RGS9	-0.96285275	0.0095692
GEMIN7	0.635482171	1.46E-12	AP002761.4	-0.963300766	1.27E-17
MAFK	0.63545022	8.11E-08	TMEM139	-0.964503891	5.17E-05
TNPO2	0.634345261	1.88E-18	TPI1P2	-0.964925132	0.0083868
MRI1	0.634307512	3.83E-13	SRGAP2	-0.964994528	1.76E-16
AC006504.5	0.634054459	0.001416	NTN4	-0.965845063	0.0036184
HAGHL	0.633968693	4.19E-13	LRRCC1	-0.966161678	5.26E-08
ZNRD1	0.633835823	2.51E-06	CATSPERG	-0.966527763	0.0076745
LTV1	0.633806694	1.47E-14	ELFN1	-0.967005638	6.48E-09

ITGB1BP1	0.633518394	3.65E-14	RNF224	-0.968088273	0.0001042
ISYNA1	0.633504827	4.28E-10	RDX	-0.968163977	2.34E-34
ACTA2	0.633428713	0.000166	BTG1	-0.968165605	5.70E-20
C3orf52	0.633425171	0.008829	CKAP2L	-0.968557622	8.37E-08
ZNF485	0.632819117	1.07E-05	CTAGE15	-0.968757877	0.0195262
KARS1	0.632580798	3.71E-14	MYEOV	-0.969845081	5.21E-14
DGKE	0.632216338	7.58E-09	AMOTL1	-0.970006022	4.41E-13
PA2G4	0.632180996	1.27E-22	KLF12	-0.971287891	4.39E-07
GNAS-AS1	0.632147602	0.007982	HCAR3	-0.972270826	0.013065
RP9	0.632099945	1.09E-07	ANXA6	-0.97281881	8.34E-19
PAX9	0.631886644	2.81E-07	TRIM16	-0.972936599	1.07E-16
JTB	0.631390767	6.97E-15	ARID5B	-0.973994469	1.17E-12
PITPNC1	0.631235974	3.94E-13	AC004943.2	-0.974252486	5.77E-08
EIF1AX	0.631179694	9.83E-12	SOCS1	-0.97543058	0.0361948
USP18	0.63063419	5.18E-06	AC015712.6	-0.977860327	4.19E-05
UAP1	0.630385361	9.45E-11	SLCO3A1	-0.978375425	7.48E-07
FARSA	0.630238102	1.95E-20	ITPRIP	-0.978470739	7.73E-08
MRPL30	0.629709208	1.61E-13	LAMA3	-0.978745351	0.00319
PEBP1	0.629491965	4.25E-19	TNS3	-0.979659809	0.0008712
NDUFAF1	0.629077185	1.01E-06	PLA2G4C	-0.979867176	0.0006889
RUVBL2	0.629044335	1.55E-13	VSIR	-0.980846253	0.0087251
HSPA12B	0.628273086	0.035156	C3orf67	-0.981098048	0.0015398
SURF2	0.628272635	4.66E-11	SH3TC2	-0.982793655	0.0045543
ZBTB45P2	0.626953686	0.011216	KYNU	-0.983244227	2.01E-07
CLDN15	0.62630726	0.000105	AC159540.2	-0.983491984	0.0002564
PSPH	0.626174946	1.79E-09	STARD8	-0.983775296	0.0390749
NUDCD1	0.626088262	5.75E-16	MAPK8IP1	-0.983818818	0.000207
MTFMT	0.625980236	6.69E-08	IFITM10	-0.984059643	0.0287052
DUSP7	0.62588426	2.84E-05	AQP3	-0.985639407	2.93E-10
ZMYND19	0.625327182	8.26E-20	SYT10	-0.985863421	3.68E-11
CEBPZ	0.625135562	5.91E-13	CDC25C	-0.985956049	1.84E-07
WDR36	0.625031287	7.49E-10	TRGC1	-0.986147891	1.67E-33
LDHA	0.625019399	1.26E-08	ZSWIM4	-0.986275554	3.21E-07
EIF2A	0.624664799	3.46E-10	LINC01963	-0.987042625	3.17E-06
AUP1	0.624552014	1.51E-15	BTC	-0.988261384	2.75E-07
NDUFB8	0.624345273	2.99E-08	C1QTNF6	-0.988415842	7.47E-31
TIMM8A	0.624241897	2.19E-12	PSCA	-0.988495308	0.0001142
PRR22	0.624238361	0.004925	AP006222.1	-0.989338861	2.83E-07
PSMB5	0.62416494	1.14E-14	MARCKS	-0.989597713	1.50E-17
MRPL12	0.6239444	2.50E-10	GRHL3	-0.989723025	3.43E-28
EMC6	0.623700299	7.55E-12	PCDHA12	-0.990669733	0.0019249
ANKS3	0.623239228	5.81E-09	AC015802.1	-0.991440545	0.0388354
AC114271.1	0.622958439	0.015309	SYNPO	-0.992586686	2.00E-05
H2BC11	0.622767019	0.019936	SSPO	-0.992826808	6.59E-05
APIP	0.622374609	7.01E-06	APCDD1	-0.992828611	6.32E-05
NOC4L	0.622302703	4.44E-11	VASH1	-0.994393664	1.33E-12
FASTKD3	0.62180329	7.17E-05	STARD13	-0.994730992	0.0007905

EXOSC3	0.621626931	2.41E-10	GPX3	-0.994774264	7.65E-11
RPL22L1	0.621585192	1.37E-08	ZNF682	-0.995247749	0.0044368
RRP7BP	0.621516684	5.36E-05	CCDC171	-0.997166888	0.0193131
AC002310.5	0.621401402	0.009302	EHD2	-0.997901997	1.46E-09
XPO6	0.620850243	1.79E-14	MUC1	-0.997992192	6.83E-07
SMIM20	0.620537121	2.68E-05	NGEF	-0.999068191	0.0145388
NT5C3A	0.620245901	6.79E-09	ENTPD2	-0.999475794	8.40E-05
DKC1	0.619408643	4.95E-20	ADAMTSL5	-1.000342141	4.42E-06
MTHFD2L	0.619295051	3.25E-05	ACOT4	-1.000668481	1.61E-07
TASOR2	0.6189657	1.07E-07	CALML5	-1.001766493	0.0015944
INTS10	0.618933383	3.39E-10	GMDS-DT	-1.002011811	0.0142927
MRGBP	0.618705438	6.38E-12	CCDC15	-1.002730346	0.0002875
UQCC3	0.618107567	1.32E-09	CMTM3	-1.002740243	0.0052823
CAD	0.617308914	2.55E-13	LINC00461	-1.003019559	0.0034559
UBE2S	0.617140785	6.45E-11	MBOAT1	-1.003227997	0.0150623
SLC30A1	0.61681753	3.27E-08	COL6A2	-1.003283171	1.21E-05
MAPK15	0.615751443	0.000932	HSPA2	-1.003297222	4.62E-08
WDR18	0.615697355	6.99E-12	AL139385.1	-1.003805644	0.0010118
FAM86B1	0.615596919	6.07E-06	KRT15	-1.004003746	0.0320217
TFR2	0.615484992	0.001332	ZNF750	-1.004086946	0.0045225
FTSJ1	0.61484435	2.82E-12	E2F2	-1.004469997	1.19E-06
PGAM5	0.614432026	1.18E-14	HERC6	-1.004678046	3.60E-07
DMKN	0.614307812	9.28E-15	CASTOR1	-1.004830014	0.000941
COX10	0.614091764	2.23E-07	FAM66D	-1.005207646	0.0009111
IPO5	0.613609812	1.54E-10	LINC00689	-1.005274421	0.0197789
AGTRAP	0.613131502	1.35E-08	SLCO4C1	-1.005356091	7.72E-06
ACBD6	0.613033833	8.55E-13	GNG7	-1.006183365	0.0002284
MRPL45	0.612448136	3.33E-09	DIRC3	-1.00634567	0.0011593
SMYD5	0.611950691	7.02E-11	MT2A	-1.006598072	0.0030086
WDR35	0.611628815	1.20E-07	ATP2A3	-1.006613783	5.92E-57
PTDSS2	0.611294292	1.72E-10	BMP8A	-1.007205292	0.0242706
PDCD5	0.611249193	1.39E-12	ZNF462	-1.007773024	5.37E-15
TOMM6	0.611160888	5.41E-11	ADORA1	-1.007906838	0.0372645
ASH1L-AS1	0.610256394	0.012467	PAPLN	-1.00792924	8.63E-06
FBXO22	0.610127615	4.72E-15	C2orf72	-1.009667176	0.0001767
DHX34	0.609436855	3.98E-09	SLC25A42	-1.009977191	1.30E-12
MDN1	0.60889431	4.84E-07	CEACAM5	-1.010227784	0.0026929
NUDT5	0.608823752	1.37E-13	ITGA5	-1.011490865	4.70E-19
NPIPB11	0.608735227	0.005712	PRRT2	-1.012601992	2.61E-06
RPL14	0.608479674	1.84E-12	ARSD	-1.012632602	1.83E-24
STOML2	0.608233059	7.79E-13	BDKRB2	-1.012914428	4.58E-13
CEP83	0.608220744	4.24E-08	NCAM2	-1.0130818	7.08E-18
FBXL4	0.606977119	5.49E-07	MYZAP	-1.013541053	2.78E-07
TIMM23	0.606898149	3.09E-14	SLC2A10	-1.014390919	7.22E-17
TTC27	0.606125554	6.61E-08	TIGD3	-1.014988782	0.004624
RPRD1A	0.605805453	5.96E-11	AC104825.1	-1.016530884	0.0032191
MNT	0.605786773	1.02E-05	ITGA2	-1.016970199	9.03E-23

UTP4	0.60565262	1.33E-15	HIVEP3	-1.017280733	0.0001509
NTPCR	0.605614759	4.64E-09	C18orf54	-1.01769134	1.01E-06
RELT	0.605378171	2.13E-07	IKZF2	-1.018439787	1.02E-09
RABGGTB	0.605334614	8.17E-12	DGCR9	-1.020001572	0.0072835
TFRC	0.60447394	1.52E-10	ARL4C	-1.02062517	8.60E-07
AC008966.1	0.604127581	0.000978	SLC25A35	-1.022191837	0.0016001
ZNF331	0.604048861	1.46E-05	SGMS1-AS1	-1.022544104	0.0006452
PRDX6	0.603817992	2.55E-15	HEG1	-1.022609759	6.78E-06
UTP6	0.603814783	1.75E-11	PLCD1	-1.022915689	0.0017578
TRAF4	0.603230068	2.88E-09	RAB19	-1.023021508	0.0001244
PRKX	0.602817194	3.31E-09	CEMIP2	-1.023466088	1.60E-27
PPT2-EGFL8	0.602728999	0.005262	PLEKHO2	-1.024849616	0.000206
NDUFAF7	0.602625936	2.85E-06	PRIMA1	-1.025396782	0.016811
WDR27	0.602588255	2.76E-07	RARA	-1.026862341	0.0034908
SETDB2	0.601677547	0.000293	AL359258.3	-1.027423568	0.0085551
NOL7	0.601420563	1.84E-12	HELZ2	-1.030018674	2.29E-15
UTP23	0.601218689	9.43E-09	IGDCC3	-1.031327911	0.0002921
SRPRB	0.600982155	1.68E-12	AC245297.1	-1.032800431	0.0067687
TIMM9	0.600755713	5.28E-08	PPL	-1.032868343	2.44E-42
LRPPRC	0.600504511	3.88E-12	CCDC69	-1.035766547	3.54E-07
ZNF581	0.600162328	3.96E-05	ALDH6A1	-1.035936196	6.90E-13
SLC25A12	0.599834967	4.60E-05	P2RX2	-1.036758938	0.0044631
ANTKMT	0.599644257	2.30E-10	ALPK3	-1.037087978	2.01E-05
PDHA1	0.599276488	1.37E-16	ZNF117	-1.037371828	2.71E-14
ZNF770	0.598899163	1.85E-07	AC068580.3	-1.039807181	0.0250683
WRAP73	0.598194121	6.53E-08	ZBED6	-1.039884427	0.0119865
SPATC1L	0.597829887	0.000216	PCDH1	-1.040647521	1.25E-16
MRPL46	0.597639328	2.13E-09	SYNM	-1.040700356	2.38E-07
FP565260.1	0.597564255	9.47E-13	IL1R1	-1.041942965	1.96E-17
RP9P	0.597265698	0.000512	HIP1	-1.042058635	1.28E-12
SNHG16	0.5971842	1.71E-09	AC100810.3	-1.042138266	0.0030245
PARVB	0.596943273	0.005913	GPR37L1	-1.043220505	1.36E-07
AGAP3	0.595686128	9.66E-14	BAIAP2-DT	-1.04415111	7.07E-13
MBTD1	0.595519871	7.02E-07	BSN	-1.044385577	0.009302
LETM1	0.595301597	2.65E-11	SELL	-1.044654094	0.0092183
ARL5A	0.595036511	6.84E-07	C9orf152	-1.045243903	5.09E-06
ME1	0.594631439	8.59E-11	PRKACB	-1.045849018	8.70E-06
RBM38	0.594283699	1.23E-11	SPEF2	-1.049065907	4.59E-05
ETS2	0.594182938	8.95E-06	B4GALT1	-1.049742105	1.61E-15
TSR1	0.594152248	4.80E-17	LHX1	-1.05027377	0.0001388
ZNF692	0.59391395	2.28E-07	CNIH2	-1.050531085	0.0005067
FEM1A	0.592642741	2.74E-05	IQCN	-1.051014609	0.0322729
ALG5	0.592215056	4.76E-08	FMNL1	-1.051867422	1.79E-09
MTURN	0.591828271	1.14E-05	C1orf226	-1.052398433	0.0061773
CTC1	0.591412747	5.23E-09	RIMS3	-1.052624081	2.65E-15
GRWD1	0.591345679	4.79E-13	AMTN	-1.053151659	0.000523
MPST	0.591294826	1.43E-09	ARHGDIB	-1.053577036	0.0100562

DLAT	0.590930523	1.48E-12	DIO2	-1.054618768	0.0020212
DGAT2	0.590651514	0.003249	ZMAT1	-1.056174906	0.0007464
ZNF696	0.590383865	6.91E-13	PIP5KL1	-1.057792371	0.0098184
C3orf33	0.590279663	0.000171	SDC2	-1.057826555	1.99E-09
TXN	0.589917504	5.53E-10	DUSP4	-1.058984747	4.69E-47
GPT2	0.589668154	1.15E-15	CA12	-1.05937714	0.0368233
DDX39A	0.589574401	4.63E-21	DMC1	-1.060880246	0.0373942
PHB2	0.58881731	6.44E-14	ESR1	-1.060986249	6.02E-23
EIF3M	0.588311098	1.07E-12	PLEKHA4	-1.061755881	0.0180404
DUS1L	0.588186917	1.64E-18	AC098934.1	-1.061882255	3.54E-06
OSGIN1	0.588129041	4.51E-09	ABLIM3	-1.061994299	8.16E-16
VDAC2	0.58786334	5.14E-14	MCTP1	-1.062656608	0.0039973
AC093323.1	0.587840628	4.50E-07	AMIGO3	-1.062781113	0.0177516
CYP1B1	0.587258861	7.10E-06	LRRC75A	-1.063057679	0.0002197
VARS1	0.587099852	2.25E-14	SLC16A4	-1.063357658	0.0059086
GPR155	0.587017807	0.041874	AC139099.1	-1.063664936	7.81E-05
SSB	0.586815767	3.33E-11	ABCC3	-1.063695943	1.08E-19
NXT1	0.586633497	1.30E-08	MTMR11	-1.06382291	2.42E-12
CACYBP	0.586584339	5.66E-13	AC244197.3	-1.064338419	1.04E-06
RPP38	0.586458116	7.14E-06	REEP2	-1.06507589	0.0006946
UQCRH	0.586427481	1.33E-12	ABCA7	-1.06552685	1.32E-10
PLA2G12A	0.585762084	5.99E-09	NAV2	-1.065695594	9.01E-30
GPATCH4	0.585659356	4.82E-11	AL031123.2	-1.065894569	0.0014876
KRI1	0.585546807	5.45E-10	ITGB2	-1.065976977	0.0079224
TGFB1	0.585321851	1.06E-11	FGF12	-1.067427496	3.58E-05
OGFRP1	0.584991427	1.39E-05	SP6	-1.067524872	0.0013946
EEF1B2	0.58498141	3.86E-14	MVP	-1.068360135	2.75E-21
			MX1	-1.068666971	0.0035294
			AC092821.4	-1.068925477	0.0045974
			MYBL1	-1.069350863	7.36E-06
			MEGF6	-1.069586288	3.56E-24
			GGT6	-1.070041266	4.23E-05
			TIMP2	-1.070372531	4.35E-25
			APH1B	-1.07045256	5.07E-07
			FAM114A1	-1.071484662	5.06E-08
			CDKN2B	-1.071694044	0.0011582
			AC015813.6	-1.072798926	2.12E-17
			PLXDC2	-1.074706255	4.57E-05
			AC096677.1	-1.075028141	0.0046113
			TPO	-1.075256569	0.0007732
			AL365181.2	-1.07582689	4.61E-07
			ZBTB38	-1.076167688	2.92E-09
			CES3	-1.076240892	0.0010146
			ARNT2	-1.077886795	2.43E-36
			AHRR_1	-1.078441534	0.0024667
			IGF2BP1	-1.081918104	0.0198702
			PLEKHH2	-1.082584417	1.17E-05

APOBEC3F	-1.084165378	0.0046201
CLCF1	-1.084582152	1.02E-05
MALRD1	-1.086326456	0.0062085
AGAP11	-1.086892224	8.28E-06
CCDC96	-1.088937634	0.0005606
FHDC1	-1.089108277	1.31E-11
MAPK4	-1.090309141	0.0006147
STOX2	-1.090340559	5.93E-05
AL031123.3	-1.09058119	0.0237322
A4GALT	-1.091683214	0.0029988
SQOR	-1.092066609	3.25E-13
SUSD2	-1.092384017	1.82E-06
DAPK2	-1.094846544	1.85E-09
VGLL1	-1.095085177	0.000567
LPP-AS2	-1.096427371	1.31E-05
NRM	-1.096657567	3.69E-07
SOWAHB	-1.097126298	9.22E-09
CYP46A1	-1.097745584	0.0012795
SLC22A18AS	-1.097908585	0.0023414
STON1	-1.098955007	9.25E-07
USP51	-1.099228987	0.0010065
KCND1	-1.09939395	0.0004447
LINC02321	-1.101377771	0.0075574
CELSR2	-1.102470271	3.25E-06
DLC1	-1.103855505	2.58E-10
LUM	-1.105060825	1.94E-07
FA2H	-1.10851202	1.48E-05
TMEM40	-1.108516431	4.02E-07
GPR132	-1.108935861	0.0050039
NHS	-1.110016714	3.65E-20
TMSB4X	-1.110088157	6.52E-19
TXNIP	-1.110389615	4.08E-39
SPEG	-1.110657219	9.80E-08
AMZ1	-1.110975576	3.76E-09
CBFA2T3	-1.113051147	0.0021034
EEPD1	-1.11355592	0.0011589
PRR36	-1.113608938	0.0001438
TTYH3	-1.113709303	1.62E-17
SLC34A3	-1.114239915	0.0003899
ITGB4	-1.117105286	4.69E-46
DDX60	-1.119110062	1.09E-05
SBK1	-1.121205753	5.66E-09
PALLD	-1.121379289	2.12E-29
LHX2	-1.121671624	5.45E-05
LAMB3	-1.122224518	0.0003031
ANXA3	-1.122960837	9.40E-13
MST1R	-1.124188167	3.18E-06

IFI27L1	-1.124815404	0.0007215
SALL2	-1.125785962	0.0226822
SLITRK6	-1.126285028	0.0005864
AC007743.1	-1.127913996	0.0062788
AC093001.1	-1.128113527	3.02E-21
ZFHX2	-1.128641887	0.0001018
C4orf19	-1.129703441	1.80E-11
AC027601.1	-1.131137977	0.000567
PIM1	-1.131203489	2.07E-06
SLC1A4	-1.132068625	6.23E-12
SLC16A2	-1.133064212	0.0002216
THBS1	-1.133631192	8.38E-20
FER1L4	-1.13401301	5.14E-18
FAM47E-STBD1	-1.134324424	2.25E-05
DDAH2	-1.134498913	0.0052406
CISH	-1.135093332	0.0422715
AC040970.1	-1.137260911	0.0003736
TNFAIP8L1	-1.137297222	4.70E-06
MAFF	-1.138209152	1.33E-05
ANOS1	-1.138597237	8.12E-05
ADGRF4	-1.138904017	7.45E-05
MYO15B	-1.141172055	1.86E-13
IRF7	-1.141810678	3.79E-09
FRAS1	-1.142038379	0.0002551
TCHH	-1.142153177	0.0001336
GALC	-1.145917556	1.10E-06
GSTM2	-1.14872767	0.0141674
FGD3	-1.150631025	2.35E-18
DUSP10	-1.151074582	0.0001526
KIAA1210	-1.153699295	0.0009056
ZNF396	-1.154498733	0.0004553
CEMIP	-1.155100732	2.82E-10
GPX8	-1.155421918	2.61E-12
ANKRD6	-1.159799482	0.0041901
CLIP2	-1.159908236	6.70E-08
TTLL7	-1.160790217	3.13E-06
PPP1R18	-1.161090414	3.09E-14
ADGRG6	-1.164403615	3.27E-47
LMO7	-1.164599655	1.49E-43
CTSV	-1.164775597	4.66E-06
CDKL5	-1.165661593	4.10E-10
MAFB	-1.169211766	0.0001881
PXDC1	-1.169629839	0.0004005
MYO16	-1.171503362	0.0033989
MUC3A	-1.175267863	0.0009307
FBXO48	-1.175781863	0.001104

	LINC02620	-1.175880012	0.0373771
	PLEKHG1	-1.176644335	0.0004664
	OPHN1	-1.177583275	1.17E-07
	MAOA	-1.17883456	7.72E-10
	ADGRA2	-1.179667588	0.0002951
	RPS6KA2	-1.179820605	2.81E-10
	PEG10	-1.183213413	4.41E-09
	AMOTL2	-1.18341318	8.03E-31
	GPRC5A	-1.1845458	1.71E-31
	SYTL2	-1.184936965	3.24E-51
	AC015802.6	-1.187167831	1.45E-05
	ETNK2	-1.188296001	9.29E-08
	HEYL	-1.189175791	5.37E-06
	APAF1	-1.189594736	2.23E-13
	STEAP4	-1.190813336	6.25E-07
	LGALS1	-1.191976227	6.23E-19
	MAB21L4	-1.192817004	6.56E-11
	INPP5J	-1.19304953	3.66E-07
	THSD4	-1.19324444	1.97E-21
	SEMA3C	-1.193470102	1.04E-19
	MYLK	-1.193767012	1.09E-05
	RIBC2	-1.19555008	9.57E-07
	LYPD6	-1.198024121	4.51E-09
	CLIC3	-1.199036184	6.50E-17
	GPR173	-1.199233233	3.95E-05
	CCDC103	-1.199472463	0.0048922
	EGFR	-1.200558333	1.12E-11
	ITGB6	-1.200916622	2.03E-29
	CRISPLD2	-1.201705215	4.03E-06
	FMN1	-1.203698039	1.73E-10
	TUBA1A	-1.203947475	1.65E-16
	AC079414.2	-1.206283737	0.0007247
	ZNF792	-1.207162305	0.0001852
	SYTL4	-1.208867737	2.97E-08
	IQGAP3	-1.209998343	2.59E-18
	OAS3	-1.210569549	1.27E-20
	SPSB4	-1.211731314	0.0022813
	DCDC2	-1.211873342	0.0004202
	AL109918.1	-1.215181288	1.23E-05
	CORO1A	-1.215928529	5.14E-06
	RET	-1.216872982	0.0290891
	ZNF836	-1.217010791	0.0018655
	PALM2AKAP2	-1.221358201	0.0007292
	ACACB	-1.221668606	3.13E-05
	C2CD4C	-1.222024245	8.88E-05
	CENPI	-1.222407046	6.23E-12
	AC080112.4	-1.229252785	0.0037451

BTBD8	-1.229635955	0.0005727
KLK6	-1.231758376	0.001082
PGM5	-1.23272551	3.00E-06
PRKG1	-1.235619935	0.0106166
SYNE1	-1.236933285	4.54E-06
EFR3B	-1.237662425	1.02E-05
SPOCK2	-1.237996281	0.0066552
PLEKHG2	-1.238648875	8.38E-17
ERP27	-1.239516642	3.36E-07
S100A9	-1.246885963	1.17E-05
RTKN2	-1.247708599	1.06E-13
CLDN1	-1.250046706	3.85E-15
ATP2B4	-1.252655218	3.96E-06
CACNG4	-1.254050852	5.00E-22
FAM122C	-1.256348598	3.07E-08
MKRN2OS	-1.260264281	0.0007572
LINC02732	-1.263288988	3.38E-15
C5	-1.264116509	1.29E-12
LHFPL6	-1.265162873	5.44E-17
PTGR1	-1.26631109	0.000327
KLF6	-1.267158518	9.67E-18
RASGRP1	-1.273390684	3.35E-07
MALL	-1.278804473	3.78E-28
CSRP2	-1.27924869	0.0005886
RIMBP3	-1.282724192	0.0044134
SH3PXD2A	-1.283020466	0.0004075
PTPRG-AS1	-1.284319464	1.76E-11
LYN	-1.287137654	0.0002217
CAPN5	-1.288851221	7.29E-19
EDIL3	-1.288889212	3.32E-05
CAV1	-1.298254692	1.08E-25
FAM167A	-1.299639126	2.46E-05
TLCD2	-1.301134208	3.46E-13
CCDC88B	-1.301678204	0.0011255
PTAFR	-1.30181785	0.0001266
COL5A1	-1.302979657	7.64E-18
COL12A1	-1.304038497	1.04E-19
GCOM1	-1.30419098	8.36E-05
AC012513.3	-1.306983133	1.30E-05
PDK4	-1.307588307	0.0001563
ESAM	-1.311099843	4.37E-06
ARRDC3	-1.311616331	1.40E-06
LRRC15	-1.315017358	9.26E-07
AC010735.2	-1.316869893	0.0010006
PRICKLE2	-1.31750117	4.86E-08
FRK	-1.324435972	3.42E-06
ABCA4	-1.326880958	0.0118682

MID1	-1.330743294	6.48E-33
TOX2	-1.331454678	4.30E-05
NEDD9	-1.333553933	4.18E-07
RAP2C-AS1	-1.337921091	0.0014534
PCLO	-1.339273851	8.67E-14
MEIS3	-1.34668861	4.83E-15
EFEMP1	-1.347792425	3.03E-13
LYPD3	-1.34851276	1.71E-16
EPHA4	-1.348884428	3.42E-22
PRLR	-1.351292457	7.56E-15
KLF7	-1.351734982	4.33E-05
NFATC2	-1.357170566	3.14E-17
SERPINA1	-1.36042563	0.0003843
AC121757.2	-1.362353677	0.0014876
HSPB8	-1.363391621	0.0080537
MAP1A	-1.366699321	0.0013502
FREM2	-1.367801809	3.42E-19
STRIP2	-1.370945358	0.000384
WNT9A	-1.371173662	0.0001794
ULBP1	-1.372404132	6.38E-08
SCARA3	-1.375180368	1.21E-14
ZNF185	-1.376944408	7.80E-44
MATN3	-1.37743638	3.19E-11
TGM1	-1.381397824	2.47E-15
TMEM47	-1.381525257	0.0005805
INPP4B	-1.382192743	2.14E-27
BANK1	-1.383860116	8.57E-06
PAPSS2	-1.385994798	4.39E-38
AL157935.2	-1.387071813	0.0006359
TRIM2	-1.39088128	2.08E-06
AC110619.1	-1.392054106	1.47E-30
ARHGAP33	-1.396428518	2.19E-12
FAM155A	-1.403543405	1.23E-10
MRAS	-1.407566231	9.09E-06
ST6GALNAC2	-1.4184371	2.29E-14
AL732372.1	-1.419449317	3.90E-08
UBA7	-1.428273984	1.67E-08
AC144831.1	-1.428532717	1.39E-06
PHLDB2	-1.432163784	8.49E-06
LAMC2	-1.438238035	3.82E-24
RIPOR3	-1.438761807	8.66E-16
AC022034.1	-1.439845731	1.55E-09
SAMD9	-1.443182628	1.98E-08
SPOCK1	-1.445448209	2.05E-13
FN1	-1.447996957	2.46E-29
AFAP1L2	-1.451571186	2.80E-06
CMYA5	-1.456388452	2.95E-19

FAT4	-1.45969351	1.01E-09
SLFN5	-1.460018489	6.26E-15
ACHE	-1.468918718	0.0129768
MAGED4B	-1.471500191	5.07E-07
LINC01213	-1.476513236	0.0011895
LINC00514	-1.477809233	2.38E-05
SCN8A	-1.504828688	1.66E-07
DNAJB5	-1.507789507	3.87E-09
LRRC46	-1.511248933	1.29E-06
UGT1A6	-1.51657415	2.91E-08
CLIP4	-1.525497977	2.90E-05
DKK1	-1.533439215	2.18E-25
TCIM	-1.534584571	2.09E-13
TENT5B	-1.548757542	3.30E-05
LIN7A	-1.553234322	3.54E-13
PRSS23	-1.576228826	0.0017715
SEMA3B	-1.59353442	0.0002001
TNFRSF11B	-1.60089026	3.65E-08
ABAT	-1.601198193	4.95E-10
HRH2	-1.603049002	4.91E-05
AL158211.5	-1.605155207	5.21E-05
TNS1	-1.606276241	1.97E-08
SYTL5	-1.611897144	0.0028675
AC131649.2	-1.616861543	1.71E-08
NEURL1B	-1.617289529	2.14E-17
CYP26B1	-1.621818248	2.01E-06
LINC00365	-1.622308347	7.19E-05
CGNL1	-1.637984036	2.56E-08
JPH2	-1.645609675	7.60E-09
CMPK2	-1.64637252	3.46E-05
MYPN	-1.652377022	4.61E-22
NRCAM	-1.670340979	1.71E-25
PLXNA2	-1.682577561	6.97E-16
PAOX	-1.691481159	1.87E-06
SERPINA3	-1.692498893	5.78E-07
LINC02747	-1.69682187	1.24E-10
APOL6	-1.706216815	6.00E-18
APOBEC3B	-1.737546365	6.12E-13
MAP2	-1.74476769	2.08E-14
PCDHAC2	-1.76459825	4.53E-14
ABCC13	-1.792301823	0.0001054
PSG5	-1.798457024	4.54E-06
DHRS2	-1.806317385	2.22E-08
INHBA	-1.829807877	3.80E-18
AL354740.1	-1.84221277	3.52E-08
SLCO2A1	-1.853260427	9.76E-10
TGFB2	-1.860669311	2.17E-17

	SNAI2	-1.894166773	2.66E-06
	PSG9	-1.913367873	2.04E-05
	NT5E	-1.94169842	1.03E-35
	MIR503HG	-1.969192754	3.11E-15
	AL590004.3	-2.005858889	1.11E-16
	HMCN1	-2.028877911	4.52E-07
	SLIT2	-2.036898066	1.22E-10
	NECAB1	-2.193808119	0.0007335

Table S7: mRNAs significantly up- or downregulated (≥ 1.5x fold change) in MCF-7/pRTR-c-MYC cells (*AP4* wild-type/*p53* -/-).

Significantly up-regulated mRNAs			Significantly down-regulated mRNAs		
Gene symbol	Log_2Fold Change	padj	Gene symbol	Log_2Fold Change	padj
AC026786.1	4.168743284	1.60E-51	MR1	-0.585044157	1.34E-07
MYC	3.903295402	0	TFPI	-0.58520387	8.18E-16
PDE4A	3.001954731	1.49E-52	RABAC1	-0.585262689	6.60E-11
GAL	2.756563751	1.17E-187	KIFAP3	-0.585284581	0.000472111
USP2-AS1	2.745916792	2.34E-29	ATP6V0A4	-0.585721812	6.63E-09
FABP5	2.724558312	1.20E-265	FUT4	-0.585882024	0.002397269
CR2	2.338092286	3.71E-12	TTC39A	-0.585887917	2.66E-12
KCNQ4	2.185403978	3.15E-20	POLR3GL	-0.585953519	2.10E-07
DUSP2	2.136294355	1.63E-45	RHOBTB2	-0.58624241	1.88E-13
POLR3G	2.091549742	1.32E-125	EMP2	-0.586464975	1.89E-20
LRFN1	1.899460333	8.76E-24	SENP7	-0.586645492	4.78E-08
WNT10B	1.863022183	7.92E-39	GLMP	-0.586852439	9.38E-09
PLD6	1.858176844	8.99E-64	PRSS27	-0.587725767	0.048251373
ADGRE2	1.857540829	8.04E-10	RORA	-0.588366593	0.031748198
RAB3IL1	1.794692082	4.70E-43	C2orf72	-0.58863795	0.010419683
SLC16A1	1.791770196	1.15E-212	EVI5L	-0.588716547	8.34E-12
NPTX1	1.784690357	6.08E-08	GPR158	-0.589054098	0.012884772
TBC1D4	1.751374829	4.41E-77	MAN1A1	-0.589188988	2.03E-07
CPNE7	1.744356869	3.52E-16	TMEM87B	-0.589268102	8.11E-14
MATK	1.738545515	4.85E-86	ERMARD	-0.589515099	1.21E-06
PCOLCE2	1.720706642	1.32E-37	SSH3	-0.589951119	2.45E-17
PPARGC1B	1.705575216	8.12E-31	MEGF8	-0.590484584	2.78E-11
TMEM52	1.683241341	3.78E-09	MT1X	-0.59056572	0.047671617
EN2	1.625584562	3.94E-54	FZD2	-0.590828125	7.83E-06
SORD	1.621020622	9.78E-122	INSR	-0.591322886	4.86E-06
HOXC8	1.547256336	1.42E-05	LFNG	-0.591488612	8.43E-08
HRK	1.54488201	1.64E-05	TRIM34	-0.591855078	0.005426254
TERT	1.54118703	3.66E-08	TECPR1	-0.59196299	9.41E-08
RAB3A	1.537641498	2.26E-22	BHLHB9	-0.592233054	0.018931373
PAQR5	1.534887099	1.80E-07	TMCO3	-0.592514557	1.45E-13
EMSLR	1.532995273	3.11E-80	IRF5	-0.593687122	0.000164952
TAF4B	1.522432721	1.66E-67	MINDY1	-0.594131369	1.07E-06
RPP25	1.481613063	5.44E-61	B3GNT3	-0.594135034	1.34E-08
DLX2	1.464265311	2.35E-06	IRF2BPL	-0.594567063	4.76E-23
KCTD12	1.461378578	6.91E-27	LINC00888	-0.59531163	0.001924064
SLC29A1	1.459293514	1.07E-111	ZKSCAN3	-0.595433244	0.009979342
AC040162.1	1.437841716	1.08E-18	VPS35L	-0.595754392	4.77E-10
RNF125	1.428496731	1.64E-16	PLEKHA2	-0.595764009	1.01E-06
AMER1	1.409844045	1.95E-42	AL049834.1	-0.596016008	0.005149988
CENPV	1.406190586	1.12E-23	TRPS1	-0.596223352	2.86E-17
FJX1	1.402221192	1.85E-44	FAM161B	-0.596930945	0.001086243
LYAR	1.401034028	5.08E-93	TMEM219	-0.597125137	4.17E-10

HPDL	1.395315807	3.14E-70	AKAP5	-0.597976948	0.036577878
MAP3K21	1.389916636	7.90E-28	RHBDF1	-0.598328191	3.61E-22
ACTL8	1.388052051	3.47E-06	MIEF2	-0.598465121	8.46E-08
CDC42EP1	1.387989629	1.94E-84	MREG	-0.598618331	0.001976676
HS3ST3B1	1.370410811	9.47E-09	HLA-C	-0.598879153	2.96E-23
CARMIL2	1.342906197	1.40E-10	OR2A9P	-0.599017282	0.043273894
ADAT2	1.326172319	3.53E-37	MIDN	-0.599036258	1.44E-17
GFOD1	1.308494275	1.97E-27	EGR1	-0.599069163	0.004744943
SLC19A3	1.291542102	0.000137158	ZFHX2	-0.599604127	0.043193141
PPIF	1.281441935	2.36E-97	EGFR	-0.5999695	2.22E-05
RRP9	1.280738481	8.99E-64	PRSS16	-0.600229677	0.012771903
ANKRD13B	1.278552645	2.81E-37	CRYL1	-0.600438388	1.77E-07
ARID5A	1.273774072	4.79E-15	CAPG	-0.600442204	5.31E-19
PODXL2	1.266758468	6.68E-47	ZNF287	-0.600656364	0.001359099
SLC25A19	1.260927197	1.67E-47	AC016747.1	-0.601166817	0.001230974
AC007342.4	1.258360566	0.000730911	CDKL5	-0.601420352	0.001408544
JPH1	1.258113821	3.03E-29	MXD4	-0.601548984	9.26E-19
PFKM	1.25562162	1.31E-90	SLITRK6	-0.601639081	0.004625962
MPP6	1.246682766	2.85E-47	MATN2	-0.601824268	1.54E-06
NPM3	1.24593111	4.27E-87	AC026748.3	-0.60233853	0.001066852
NRARP	1.230978078	5.62E-77	GHDC	-0.602405728	6.22E-07
SLC27A5	1.22648696	3.48E-17	ZNF606	-0.603389387	0.01607702
NFE2L3	1.220789336	7.82E-22	ZNF737	-0.603581542	0.007069973
GEMIN5	1.217565663	3.11E-70	CDC25C	-0.603688729	2.62E-05
GPRC5B	1.210266495	2.95E-05	WIPI1	-0.60393687	1.33E-11
JAG2	1.210104722	2.15E-47	TMEM107	-0.604107229	0.000838196
TWNK	1.208697354	6.60E-72	FADS3	-0.604359784	2.64E-10
PITX1	1.206161534	1.71E-40	DSCAM	-0.604372694	0.000177436
MXI1	1.204172477	5.94E-53	PACSIN1	-0.604918372	1.21E-09
ABCC4	1.196223303	1.07E-32	TMPRSS13	-0.604959939	3.41E-08
RGS16	1.196139256	2.82E-28	PLAAT3	-0.605008193	1.50E-08
DUSP9	1.195538598	4.54E-05	OLFM1	-0.605090653	7.24E-11
LRP4	1.191184302	2.88E-06	CD59	-0.605447406	3.79E-23
FAM81A	1.178296339	1.31E-12	GABARAPL1	-0.605702697	0.043496794
NAT8L	1.177162139	4.29E-34	DOCK11	-0.606166035	2.44E-07
KLHL31	1.176125873	0.009580722	SHFL	-0.606468367	4.16E-06
MIR17HG	1.175548265	0.002610658	ZNF333	-0.607038428	0.016474662
SACS	1.168582882	1.20E-39	TOM1L2	-0.607103538	8.41E-12
SNAI1	1.168225541	0.000145496	ZBTB22	-0.607450752	1.57E-09
FAM216A	1.160159107	6.94E-14	FHL2	-0.60748465	4.20E-08
FAM89A	1.157729288	4.64E-10	LMNTD2	-0.607648066	0.02324875
PALD1	1.156654877	4.73E-05	PGAP3	-0.607785513	7.19E-05
GALNT18	1.152843041	1.33E-43	CAPRIN2	-0.608227945	0.010990887
GALNT14	1.152730767	3.02E-24	TAPBPL	-0.608352584	2.84E-05
NOP16	1.151147592	6.26E-98	THBS3	-0.608793857	1.18E-08
CHCHD4	1.14372346	7.21E-57	ACER2	-0.608817614	0.045190394
IL10RB-DT	1.143640996	0.006601152	SFR1	-0.609851069	0.004273291

NR1D1	1.142948435	2.76E-12	ERV3-1	-0.610709936	3.07E-06
YRDC	1.139111698	9.16E-28	ARID5B	-0.610844713	4.08E-12
AC009831.1	1.138558853	0.000570686	BICDL2	-0.611013123	3.39E-15
BEND3	1.137910206	2.21E-25	TP53TG1	-0.611131074	1.84E-05
SLCO4A1	1.135432641	2.72E-33	MELTF	-0.611339624	1.67E-10
PNP	1.133570925	1.91E-62	AL162458.1	-0.611655716	5.76E-05
RPIA	1.132057549	1.94E-66	GOLT1A	-0.611807852	0.00245137
CCDC86	1.128193087	1.02E-86	ACOT4	-0.612085298	0.021285016
FAM131C	1.127678412	8.63E-05	CRIP2	-0.612467082	2.98E-28
AC106820.4	1.12101298	0.007184018	BAIAP3	-0.612853671	0.001117946
METTL8	1.120634306	4.40E-47	RAB5B	-0.612986407	4.57E-21
RNF145	1.114117236	1.16E-08	TSPAN9	-0.613012092	3.60E-08
ZNF296	1.114041546	1.50E-15	PTPRG-AS1	-0.613103915	1.03E-05
BTN3A2	1.11393867	0.001814134	RAB8B	-0.613119244	1.67E-06
AC146944.3	1.106956496	0.000122779	COL11A2	-0.61365434	4.06E-05
SNHG30	1.10182859	3.82E-16	SYT12	-0.614617422	1.38E-19
AEN	1.101204624	4.84E-48	TRIM45	-0.614660121	3.48E-10
FERMT1	1.098118093	9.85E-10	AL031777.2	-0.614811161	0.023802977
MLKL	1.097584124	3.74E-42	MICALL2	-0.615207532	1.97E-14
BAG2	1.09688704	3.28E-18	SAMD15	-0.61536236	0.000907885
LOXL3	1.094441204	3.35E-12	COL4A5	-0.615397758	3.59E-05
DBF4P1	1.092911211	0.003237982	SLX4	-0.615898149	4.06E-10
SMKR1	1.092743136	5.04E-22	GPR37L1	-0.616054573	0.000184434
CD3EAP	1.091162071	5.22E-61	ZFP36L2	-0.616456344	3.38E-44
DNAJC2	1.090362486	6.77E-89	FAM43A	-0.616503722	0.005666298
EEF1AKMT4	1.081558871	6.07E-44	AMOTL1	-0.617065443	1.88E-18
MON1A	1.081314928	5.00E-24	TRIOBP	-0.617372635	3.79E-16
RABEPK	1.080057391	1.01E-46	PNPLA7	-0.617505484	0.006791136
PSMG1	1.079562019	1.86E-57	TNFAIP8	-0.617959571	4.56E-06
EPOP	1.079112399	1.47E-54	PRR15L	-0.61806454	2.50E-10
NDUFAF4	1.078433652	4.79E-33	NAGK	-0.618324885	1.91E-16
CAMKK1	1.077802743	3.79E-07	EFNB3	-0.619842237	0.001107915
DPH2	1.075255563	2.77E-60	EFEMP1	-0.62037173	6.49E-11
LDLRAD3	1.068563865	2.57E-20	ALDH4A1	-0.620468803	1.01E-17
LYSMD2	1.064817872	7.98E-28	KLC3	-0.620656276	0.020743313
AC012510.1	1.06466799	0.008234892	SPRY4	-0.621172854	0.043477491
AC006111.2	1.06242595	4.43E-05	DNASE1L1	-0.621906895	3.70E-14
NLN	1.061487359	6.04E-64	PLIN3	-0.622186652	3.04E-16
SLC25A32	1.054916767	9.36E-46	TRPT1	-0.622865627	1.37E-09
RPUSD1	1.048547124	1.81E-63	CTDSP2	-0.62291706	2.22E-19
DNAH17-AS1	1.047099487	0.001777912	PRSS8	-0.623065386	9.33E-16
WDR3	1.046118453	1.65E-78	BBOF1	-0.623169815	0.000205469
CDIP1	1.041764984	0.004450896	DENND3	-0.623296923	1.88E-05
RRP1B	1.037989811	9.81E-66	TMEM256	-0.623541165	4.02E-05
L3HYPDH	1.035925757	7.33E-21	ABHD2	-0.623608196	5.77E-15
NDUFAF2	1.033695268	6.28E-38	SYNGR3	-0.623797927	0.000249545
AL161756.1	1.03291938	0.005364383	LINC00365	-0.623903278	0.001026369

DDX21	1.030082276	1.87E-105	POC1B-GALNT4	-0.624115714	0.049128784
GBX2	1.027567816	0.000528841	ITGA3	-0.624183119	4.81E-25
SFXN4	1.027359964	1.79E-37	IL17RE	-0.624791635	0.012561153
GRPEL1	1.024983938	3.30E-56	AL359258.2	-0.625051251	0.025697343
ADAMTS17	1.024658758	0.000240665	NRSN2	-0.625255585	0.032425041
WDR43	1.022096705	7.19E-81	TMEM191B	-0.62540433	0.043013981
CACHD1	1.021388418	0.001968313	AC027601.1	-0.62541398	0.019695035
DDX10	1.021167503	5.36E-44	AC092718.3	-0.625527554	0.015476564
UTP20	1.020278332	3.88E-38	TSPAN5	-0.625593875	0.013597851
AC103706.1	1.016098304	0.00651578	SEZ6L2	-0.627270663	2.49E-25
CRYM-AS1	1.014011259	0.008001121	TCTN2	-0.627301295	2.27E-07
TXLNG	1.00713091	2.97E-51	PTK2B	-0.627502882	0.001343659
REXO4	1.007009327	3.48E-65	PPARA	-0.6275209	0.032528897
ANP32A	1.006670083	3.30E-56	PCDHGB5	-0.627580834	7.66E-06
SLC25A33	1.006056711	4.04E-37	PYCARD	-0.627891871	3.39E-14
AL445423.3	1.005221119	0.001324991	KIAA1211L	-0.628061917	2.07E-11
ASB13	1.003595484	1.70E-25	S100A16	-0.628115916	5.54E-29
ZNF239	1.000685201	2.01E-11	CLTCL1	-0.628122158	0.000770816
NANOS1	1.0000073	1.06E-08	FAM102A	-0.628180511	4.82E-15
AP006333.1	0.99982688	0.000648592	LPP-AS2	-0.628228225	0.012937665
COQ8A	0.99923375	4.76E-33	LINC01232	-0.628406535	0.032133886
SINHCAF	0.998277536	2.99E-52	CCDC24	-0.628698102	0.000170481
EIF2B3	0.997774284	1.79E-25	NUCB2	-0.629446729	2.95E-13
TRMT61A	0.997697153	1.20E-44	DNAJB2	-0.62950241	5.71E-15
KLHL21	0.993411544	4.72E-29	IFI27L2	-0.630010993	2.46E-05
NOLC1	0.990694894	8.38E-95	TMEM79	-0.630337473	6.69E-10
E2F5	0.987890173	3.20E-25	ACSS2	-0.631055236	2.26E-12
ICAM5	0.987533939	9.39E-05	RPS10P7	-0.631340892	0.013253396
CHN1	0.986571817	2.81E-21	PIM1	-0.631474037	0.000433134
HMGA1	0.986043938	1.65E-74	PRSS22	-0.632148648	4.62E-10
RRP1	0.985842642	1.95E-56	NFATC4	-0.632217332	0.011612319
PDCD2L	0.984478555	4.44E-24	AGRN	-0.632448679	6.37E-19
UCK2	0.98441605	7.61E-56	ZNF446	-0.632834882	0.001037485
HHEX	0.982054448	0.00047084	FARP1	-0.633032655	1.56E-14
SCML2	0.981400413	9.86E-09	AC244197.3	-0.633348114	0.001242208
TCOF1	0.980906815	1.39E-67	MAN2B2	-0.633628066	1.27E-09
SCO2	0.979039692	5.39E-22	SMIM29	-0.634600025	1.03E-06
PPAN	0.978340928	4.66E-44	IFITM1	-0.635554741	0.001176269
DUSP7	0.976588342	1.18E-17	IL1R1	-0.635983163	3.60E-09
RPL23AP7	0.971367696	9.90E-05	NRBP2	-0.635992714	9.95E-10
SLC16A1-AS1	0.971279794	0.00014125	ARHGEF10L	-0.636650531	8.43E-08
KLHL23	0.96661664	2.51E-17	GRB7	-0.636740196	4.94E-07
RPP40	0.964409562	9.47E-24	ZDHHC12	-0.636992013	5.87E-17
SKP2	0.961245259	2.14E-21	KCNK15	-0.637250958	2.32E-20
TMEM158	0.961011392	0.001778693	BHLHE41	-0.637409491	0.025720503
PUS7	0.960808323	9.06E-62	ACADS	-0.638909218	0.000624209
TRMT11	0.960328932	1.42E-32	TTC30A	-0.639489634	0.000279709

CCDC58	0.959977431	2.98E-30	AC005821.1	-0.639934025	0.00498031
AC011603.3	0.958630824	0.004423583	SELENBP1	-0.640294069	0.032290407
LAMC3	0.958330866	0.001761234	SELL	-0.640338364	0.002860034
POLR1B	0.95820442	3.71E-58	SEC14L2	-0.641744652	0.002423724
TFB2M	0.957192901	2.59E-26	GRAMD2B	-0.641856896	8.14E-08
SNHG4	0.956949853	8.26E-22	CEMIP2	-0.641971703	2.03E-17
FAM117B	0.956316626	9.76E-19	TTLL1	-0.64246778	0.000216795
SNHG26	0.954780041	2.71E-06	IDNK	-0.643201677	0.002142416
CYCS	0.952690168	1.62E-76	H2BC21	-0.643486239	8.26E-18
RIOK1	0.952430158	4.24E-43	PCDH1	-0.644011411	1.31E-12
IFRD1	0.952003866	1.09E-60	NRM	-0.644065345	7.55E-05
TYRO3	0.951913718	1.14E-23	OAS1	-0.644124736	1.23E-05
CCDC85B	0.950855449	3.44E-66	ARSA	-0.644831046	7.44E-09
DLEU1	0.949832954	1.91E-13	ALDH6A1	-0.644891954	3.21E-13
CDR2L	0.948666222	7.78E-39	STAT2	-0.645430057	7.30E-15
DIMT1	0.947625328	7.55E-48	CLDN23	-0.645759076	3.70E-06
ADAM11	0.947044091	4.32E-08	IL4R	-0.645769443	2.01E-11
SUPV3L1	0.945371579	1.22E-36	ZBED6	-0.645960271	4.74E-12
TASOR2	0.944986792	2.66E-12	ZDHHC2	-0.64609104	0.033783312
NFKBIB	0.941599972	8.00E-24	LINC01547	-0.646739376	2.60E-05
BCL11B	0.938960581	7.33E-09	ZNF747	-0.646817917	2.41E-05
THAP4	0.937767128	3.15E-45	COL9A2	-0.647019324	0.000251021
AC125807.2	0.936783504	5.01E-12	TSPAN31	-0.647970472	8.64E-11
C15orf39	0.936697816	1.92E-34	LINC00638	-0.648408364	0.034648062
ODC1	0.936308697	1.09E-65	DIO2	-0.648516401	2.82E-12
UBE3D	0.935344352	2.27E-08	TMEM86A	-0.64868648	0.000328898
DGUOK-AS1	0.931973327	0.003144912	EPHA2	-0.64884693	3.05E-17
SLC39A14	0.928135101	5.53E-41	UPK2	-0.649709318	6.73E-11
GLS	0.92723611	2.50E-58	WDR31	-0.649743096	0.011447094
SNRPA1	0.926608743	8.67E-64	MYADM	-0.649969811	1.05E-11
PNO1	0.923873503	1.20E-41	ALPP	-0.650015004	0.003131963
ZBTB2	0.923526976	5.18E-45	GPX8	-0.650523513	1.33E-09
NT5DC3	0.921788997	3.17E-10	WDR66	-0.650908728	0.037417547
FMNL2	0.921046413	2.61E-22	RAB26	-0.650931348	2.60E-09
POLR1C	0.919447407	5.20E-46	REEP1	-0.651152518	0.020091214
NCS1	0.916638757	7.61E-51	CYLD	-0.651320845	1.31E-10
CALML4	0.914021986	0.001139166	ST6GALNAC4	-0.65193393	0.002002803
CNTNAP2	0.913846732	0.003290861	SRRM2-AS1	-0.652408343	0.013458648
PDSS1	0.911850531	1.79E-22	OBSL1	-0.652463245	2.14E-29
CNKSR3	0.911359291	1.43E-07	MAPK4	-0.652512764	0.047965054
PRR5	0.911022135	7.13E-31	FCHSD1	-0.652866609	5.24E-08
IMPDH1	0.907852928	2.04E-55	SFXN3	-0.65373942	2.93E-07
NTHL1	0.906509843	8.43E-24	FIBCD1	-0.653989309	1.16E-09
SLC35F2	0.905359616	1.33E-32	GATA3	-0.65410395	1.17E-49
MRM3	0.905105817	1.44E-37	RPRM	-0.654416137	0.023240542
NUDT4P2	0.904828288	0.000223142	PLAUR	-0.655798553	0.013428242
SLC12A8	0.904756336	1.87E-08	LRRC23	-0.655939052	0.008874137

ADCY3	0.902227285	1.41E-43	SH3BGRL	-0.655941099	5.95E-17
RNA5-8SN1	0.90136834	0.005900427	S1PR3	-0.655952988	4.65E-13
TOP1MT	0.900634435	8.13E-55	TES	-0.656031156	6.40E-16
SIK1B	0.89888919	3.82E-22	MCOLN3	-0.656489563	0.009875578
FZD9	0.898693138	0.006692309	MOSPD3	-0.656703476	6.49E-10
NUFIP1	0.898203201	7.94E-21	LAMA3	-0.656862169	0.008180159
PFDN2	0.897671126	1.15E-32	AC008014.1	-0.657143972	0.015128399
FASTKD1	0.896372216	3.54E-22	SMPDL3A	-0.657169245	1.63E-05
BLMH	0.89626238	1.88E-31	C4orf19	-0.657510721	4.00E-11
RAPGEF5	0.89281739	0.000299315	C5AR2	-0.657805662	0.019586294
SLC9B2	0.89256409	9.63E-10	AC021087.5	-0.657808391	0.003787764
AL391244.2	0.892442329	0.003344699	TNRC6C-AS1	-0.657823503	0.000717655
SLC19A1	0.89122131	8.23E-38	RNF215	-0.658506861	2.53E-08
FXN	0.889602438	3.52E-17	RNFT1	-0.658742445	2.72E-08
PRR19	0.888346148	3.85E-06	CLCN4	-0.659079545	0.030310346
AP002387.2	0.888062986	6.64E-05	LHX2	-0.659556136	0.005911418
SCLY	0.886923144	1.26E-26	KLF4	-0.659953042	1.62E-13
PSAT1	0.885983294	6.95E-57	ZNF117	-0.659991653	6.25E-08
WDR12	0.885872875	7.40E-41	DHRS12	-0.660779798	0.013421505
AL118516.1	0.882206331	2.76E-06	C20orf204	-0.660780252	0.048186707
CEBPD	0.878470276	3.66E-10	PXDN	-0.660920685	5.27E-18
PPTC7	0.875224841	6.30E-28	SLCO4C1	-0.662257548	0.021449729
ISM1	0.873322755	0.010184143	AL162258.1	-0.662673839	0.035307741
CDC42EP2	0.873224463	0.000294462	CAV1	-0.662842743	3.83E-20
TRAP1	0.872944348	3.78E-79	MTMR7	-0.66311857	0.032305768
KBTBD6	0.872877158	4.28E-20	RNFT2	-0.663177003	0.000627445
NAMPTP1	0.872026008	4.63E-13	SH3TC2	-0.663608138	0.03144905
ATP6V1C2	0.872004412	0.000946923	TTC30B	-0.664075658	0.000541879
POLR3K	0.8712951	1.94E-46	PNPLA8	-0.664769685	9.21E-22
MCTS2P	0.870268579	0.019456982	TRIM62	-0.665370348	7.05E-10
NOP56	0.869963352	1.43E-62	LARP6	-0.665738285	0.008519193
DIXDC1	0.865166266	0.000289186	COL18A1	-0.666301468	8.34E-17
BOP1	0.864213445	3.83E-79	TPO	-0.667006267	0.015903696
C1QBP	0.862623105	4.06E-71	BNIP3L	-0.667190587	8.99E-16
XPOT	0.86161174	7.43E-78	EDN1	-0.667439173	0.003023683
PUM3	0.861260851	8.50E-41	ADIRF	-0.667532666	1.03E-09
MTHFD2	0.860830383	2.68E-71	PPOX	-0.667713696	7.58E-06
RPUSD4	0.859932391	1.66E-24	SULT2B1	-0.667732495	9.22E-06
SLC25A22	0.859326055	1.57E-36	MYO16	-0.667991516	0.021838327
FTL	0.859127443	2.49E-47	TBC1D2	-0.668445173	1.30E-07
AC097448.1	0.858425995	2.65E-06	VMAC	-0.668966763	0.027427861
NCL	0.858242771	3.16E-65	AC244090.1	-0.668983052	0.01542087
ZC3H8	0.857553925	9.73E-19	TMSB4X	-0.669446441	3.10E-51
ADGRA3	0.856276365	6.66E-35	SNAI3-AS1	-0.669716141	0.032425041
SLC7A11	0.855990354	2.74E-12	ATP2B4	-0.669813189	0.002279983
PRELID3A	0.855687339	2.92E-07	ANXA9	-0.669867261	7.08E-13
PER1	0.853367079	4.08E-12	SQOR	-0.670002842	3.11E-10

IMP4	0.851828242	3.00E-59	MOCS1	-0.670400968	0.000117463
NOTCH1	0.851228186	2.06E-20	ARNTL	-0.670442774	9.63E-07
GRK5	0.850113684	7.54E-05	EVA1B	-0.670908558	0.002362178
PMAIP1	0.849932914	1.18E-06	PHF1	-0.671445417	1.45E-11
AC011462.5	0.84976626	0.046213411	SCN8A	-0.671480073	0.011982044
MARS2	0.849401297	7.68E-23	AP006222.1	-0.671889342	0.000687555
GNB1L	0.848576949	3.97E-16	ANKRD20A5P	-0.672722848	0.008925979
C1orf109	0.848157926	1.50E-19	S100A9	-0.672891634	5.67E-05
NKD2	0.848082699	2.76E-06	AL137003.1	-0.673529592	0.043730754
MRPS2	0.847860989	5.41E-52	UPK3BL2	-0.6741818	0.035112137
DHX37	0.84735292	2.72E-45	ARHGAP29	-0.674263806	2.44E-15
CTU2	0.846590069	1.62E-24	CCDC162P	-0.674914987	0.010033943
CTU1	0.846039676	1.44E-11	OCEL1	-0.675013577	0.000380721
FBXO45	0.842162642	2.01E-54	RALY-AS1	-0.675256422	0.005195751
NOC3L	0.839295201	6.38E-31	YPEL5	-0.675332306	4.25E-17
TTLL12	0.837740191	5.08E-60	SYTL1	-0.675831329	1.08E-07
MYBBP1A	0.83560049	1.22E-48	BMF	-0.675922823	0.000345197
PCSK6	0.834825268	1.57E-12	ARFGAP3	-0.676046207	2.24E-11
AGPAT5	0.834019767	6.70E-38	MDK	-0.676856034	2.44E-22
FKBP11	0.832255026	2.78E-18	PRKACB	-0.677320319	0.00469611
TANGO6	0.832120592	3.15E-15	IDUA	-0.677321743	0.000375215
HSPE1-MOB4	0.831880147	3.65E-06	AL354707.1	-0.677473277	0.002997606
ZIC5	0.831755368	2.01E-08	CUEDC1	-0.677484218	1.06E-12
PDXP	0.831743422	7.25E-21	ERRFI1	-0.677532705	9.63E-06
DNAAF2	0.829886125	1.63E-26	SLC16A13	-0.677726932	0.001227367
LRRC3	0.829210627	0.000937852	RSPH3	-0.678119881	2.58E-07
NIP7	0.828857826	1.66E-49	TRG-AS1	-0.678634133	0.023546182
C12orf29	0.828386148	1.97E-17	CCT6B	-0.678852132	0.047819763
TRPM6	0.827416771	0.018918766	AMOTL2	-0.678875089	8.74E-20
NXPH4	0.827194105	1.47E-11	MTMR11	-0.67894957	5.72E-10
TAF1A	0.827041376	1.39E-08	UPK3B	-0.679183482	2.01E-12
HOMER1	0.826022106	4.49E-31	SEMA7A	-0.680135924	0.000283588
PTDSS1	0.825986673	1.13E-50	RIMS3	-0.680233712	1.64E-12
WDR74	0.82492759	3.14E-44	RMST	-0.680558796	0.038383176
CD320	0.824290425	4.22E-26	AC022400.7	-0.681598094	0.021612218
GARNL3	0.82325485	0.009308968	GALNT12	-0.681691472	3.45E-05
SEH1L	0.823220503	1.74E-37	AP3B2	-0.682004616	0.047091808
MAK16	0.823200912	2.45E-24	VGLL1	-0.682445323	0.001836436
THAP11	0.822734186	3.24E-29	CU634019.2	-0.682614206	0.036678346
AHCTF1	0.821909701	6.35E-34	AHRR_1	-0.682727555	0.000880965
PUS1	0.821817402	6.05E-34	FLNA	-0.683742783	2.18E-26
TNFRSF10A	0.821743613	2.58E-15	HLA-F	-0.684262273	0.000720842
C12orf73	0.821191541	8.02E-10	USP35	-0.684746103	2.94E-05
CMTM8	0.820099803	0.000118496	MAGED1	-0.685009598	8.48E-32
EXOSC4	0.819165443	9.59E-39	ZNF713	-0.68587083	0.001785481
NCR3LG1	0.819136266	0.030007308	AL365181.2	-0.685908176	0.002079514
AC022384.1	0.818740721	0.002542558	RAB19	-0.686367006	0.018533172

RIOX2	0.817979348	3.17E-39	PARP10	-0.686665896	7.79E-22
INTS13	0.81740508	2.45E-30	TCAF2	-0.686710558	0.008112666
EEF1E1	0.817398878	5.17E-23	NEU1	-0.686751029	9.28E-22
HSPD1	0.816595003	1.16E-71	AC023158.1	-0.687037658	0.002761818
CHORDC1	0.815912787	4.76E-36	H6PD	-0.687209697	1.84E-09
SLC27A4	0.815775564	2.36E-36	MARCHF2	-0.688173947	4.91E-06
EIF3J	0.815402881	7.55E-48	TGFBI	-0.688918379	0.003643243
CDV3	0.815391171	2.18E-52	CALCOCO1	-0.688970624	1.79E-12
MDN1	0.814390068	7.90E-21	ADAMTS13	-0.689101298	3.54E-05
USP31	0.813684643	2.31E-21	CDKN2B	-0.689202861	2.05E-13
RBM28	0.813565575	8.15E-43	DSC2	-0.689292903	1.35E-15
RSL1D1	0.81162889	5.49E-76	RSPH1	-0.689326319	0.000356024
SLC25A15	0.811272096	1.61E-36	ATP2B1-AS1	-0.689849972	0.00813413
VPS9D1-AS1	0.81024368	4.09E-24	INHA	-0.689912801	0.000666342
MAST1	0.809185992	0.005890739	OSBPL5	-0.690069365	1.43E-06
CCDC59	0.808475612	4.64E-32	PPP1R14B-AS1	-0.690117022	0.04921954
C16orf46	0.808453558	0.047369044	BASP1	-0.690133857	1.25E-33
NT5C3A	0.8084032	3.36E-24	USP51	-0.69065145	0.003022498
WNK2	0.808094535	1.76E-21	CTSO	-0.690674303	0.001323587
SLC25A37	0.807240759	1.79E-23	CD82	-0.690690232	0.00012874
COQ10A	0.807154814	1.90E-06	FO681492.1	-0.690727395	0.002208194
MRTO4	0.806946301	1.34E-41	PLEKHB1	-0.69166829	0.004183953
RUVBL1	0.805561877	5.01E-53	PPP2R5B	-0.69204367	8.74E-07
PM20D2	0.804812226	5.34E-23	LINC02600	-0.692347092	0.026494564
APTR	0.804798835	4.87E-12	ST3GAL3	-0.692469358	0.013174561
KLHL18	0.804088072	1.47E-21	VPS9D1	-0.692479828	1.14E-07
ADRA2C	0.803834681	3.53E-13	UNC13D	-0.692689124	9.00E-22
RPF2	0.803565162	2.53E-30	DBN1	-0.692718587	8.79E-17
AP001505.1	0.802762352	0.028798038	LXN	-0.692726673	4.28E-06
ZNF639	0.799102905	2.49E-31	OAS3	-0.692754671	2.79E-28
FAM189B	0.798106968	2.90E-22	SLC24A1	-0.693101071	1.24E-11
DGKE	0.797170211	3.93E-21	TSPAN1	-0.693214619	4.31E-06
BRIX1	0.797099967	2.62E-38	NECTIN2	-0.693290754	1.36E-29
XPO5	0.79706311	6.89E-44	TENT5C	-0.694952327	7.08E-05
CMSS1	0.796821292	1.20E-32	PBLD	-0.695545491	6.43E-05
QSOX2	0.796651925	1.70E-40	ERP27	-0.695721239	5.95E-05
CTSC	0.796590694	2.49E-17	ADIRF-AS1	-0.69580214	0.00013942
TIMM44	0.796361284	1.02E-28	EPS8L1	-0.696012808	5.86E-16
SOX12	0.796328096	2.19E-32	PALLD	-0.696265018	4.88E-23
LYRM4	0.794050721	1.83E-11	ACACB	-0.697372484	1.07E-06
TEC	0.793079431	0.000200216	APOBEC3B	-0.697667522	7.10E-05
DHX33	0.79224587	5.88E-27	OSCP1	-0.698680708	0.016855578
PACC1	0.791542851	4.83E-08	FAM221A	-0.698829554	0.001023019
ELL3	0.791057606	2.63E-08	NFKBIZ	-0.698902111	2.22E-11
DEPTOR	0.790676912	3.14E-06	TRIM16	-0.699155372	5.96E-24
ENTR1	0.790118188	5.09E-40	TRIM2	-0.699320115	0.000622016
ZIC2	0.787453596	1.70E-12	LYN	-0.699468407	0.02391598

DCUN1D5	0.786664328	1.21E-34	TP53I3	-0.699981565	5.08E-05
FLVCR1	0.785830606	9.45E-10	DUSP5	-0.70010079	6.04E-07
MTCP1	0.785515987	0.025848853	GALC	-0.700149619	0.01505069
NPL	0.78453819	0.045097623	AC093001.1	-0.701482979	8.54E-12
SHANK3	0.784450527	4.08E-07	LAMB2	-0.702130113	2.28E-34
NUDT19	0.784358396	1.04E-22	C1orf115	-0.702167387	1.27E-05
MIR4458HG	0.783596587	0.014756497	ETHE1	-0.702608193	4.25E-06
PNPT1	0.783171705	7.53E-39	MLPH	-0.702948874	1.67E-39
RNA5-8SN2	0.782705405	0.007404306	DAPK2	-0.703001831	1.27E-08
RTN4RL2	0.782511967	0.030947942	FRG1BP	-0.703110617	1.50E-12
ESF1	0.781845333	1.53E-30	PKD1L2	-0.704262579	0.01239411
CFAP97	0.781012169	5.53E-33	GLB1L	-0.704848247	0.003940069
GAR1	0.780499667	1.07E-14	DOCK8-AS1	-0.70486539	0.011292924
GNB4	0.779668419	0.000330964	CACFD1	-0.704917361	4.42E-12
IL17D	0.779572732	7.39E-10	LRP10	-0.705023082	1.14E-31
RNF138	0.779297959	2.70E-27	OAS2	-0.705587972	1.09E-06
RP9	0.778767491	1.74E-12	AC006372.2	-0.705810092	0.017014091
CISD1	0.777157489	4.20E-16	LMCD1	-0.706016442	6.18E-06
MPP3	0.777004584	1.06E-05	WLS	-0.70618595	0.01416594
HSPE1	0.77635068	3.16E-56	KIAA0513	-0.706317536	2.27E-14
YDJC	0.775572091	1.81E-33	GOLGA7B	-0.706788717	0.000189338
ABLIM1	0.774799004	1.97E-28	OPHN1	-0.708041178	6.36E-08
GRB14	0.773208452	4.29E-10	COL4A6	-0.708565776	0.005370012
Z83844.3	0.773156983	9.66E-06	PCDHA11	-0.708608715	2.51E-09
PAK1IP1	0.77219936	1.04E-23	SMAD3	-0.708824186	5.19E-20
KAZN	0.771754586	1.35E-16	CAMK2N1	-0.709952413	4.67E-21
AC107871.1	0.770726545	1.48E-06	NFATC2	-0.710048772	4.22E-06
RPARP-AS1	0.769209634	6.14E-09	DGCR6	-0.710075991	0.002423178
GLRX3	0.769126388	6.96E-39	TENT5A	-0.710464012	1.31E-14
ADM5	0.768004385	0.000104913	DNAH10OS	-0.710518544	0.000922585
CC2D2A	0.767926901	1.09E-06	SMIM14	-0.711221526	6.23E-28
POLR1E	0.767373623	4.26E-17	PRRT1B	-0.711328933	0.025631145
THRA	0.767021821	5.03E-05	EPHA1	-0.711471588	3.49E-17
RNA5-8SN3	0.766742972	0.008676367	C1orf226	-0.711840115	4.30E-05
LTV1	0.765914483	8.86E-31	CPEB4	-0.712833718	6.56E-10
AKAP1	0.764709113	2.10E-44	CD109	-0.713168015	1.95E-05
DNAH14	0.764344091	9.27E-06	SDSL	-0.714094399	1.60E-16
MBLAC2	0.763452733	1.74E-08	KIAA1217	-0.714619729	7.45E-11
NPM1	0.763196594	9.93E-64	HDAC5	-0.715904126	1.12E-05
WDR4	0.762545016	7.82E-15	CREB3L4	-0.716009127	1.91E-14
ADORA2B	0.762146838	2.54E-07	GPR173	-0.717548617	0.02629132
DFFB	0.761850344	2.95E-05	YPEL3	-0.717700088	4.08E-12
FP565260.1	0.761700119	3.24E-28	CTSK	-0.71872055	0.048187344
STOM	0.761563064	3.46E-31	LRTOMT	-0.718784964	0.000291773
CIART	0.760651851	2.78E-11	PRLR	-0.719005755	1.28E-16
FAM155B	0.760566064	5.07E-12	SMPD1	-0.71911358	2.72E-19
PDP2	0.758686319	7.37E-16	PCLO	-0.719983919	0.000380538

CDC25A	0.757674923	3.86E-14	PCOLCE	-0.720867857	0.000225817
PPID	0.756557821	1.35E-31	CES3	-0.721076574	0.030840339
NOP14	0.755497486	5.58E-43	SPSB2	-0.721190553	0.00026106
PINX1_2	0.755268517	1.04E-11	LIPH	-0.721606896	0.001617841
HEATR1	0.754612492	2.63E-29	SLC46A3	-0.721896534	8.04E-05
BOD1	0.754591554	4.86E-47	MUC3A	-0.722063025	8.01E-07
GATAD2A	0.754346692	5.24E-45	HELZ2	-0.722150037	5.39E-21
C12orf45	0.753511219	6.97E-10	KCNN4	-0.724075016	3.71E-09
TUBGCP4	0.752434409	4.43E-21	MAGED2	-0.724110997	5.22E-26
EXOSC7	0.752202902	5.08E-19	MFGE8	-0.724577119	2.53E-05
ECSIT	0.751517703	2.40E-26	TMEM154	-0.725638696	0.00977576
PPAT	0.750953351	2.45E-35	PLA2G4F	-0.726387101	0.014327778
CNNM1	0.749511987	6.41E-07	HID1	-0.726660031	4.26E-28
TFAP4	0.749351224	2.76E-21	AC092117.1	-0.72723822	0.000142774
AC125257.1	0.74915626	0.003749388	ABCD1	-0.727338801	4.12E-07
C20orf27	0.7491526	4.11E-47	ZNF703	-0.727548448	0.000218228
RNASEH1-AS1	0.74848059	1.11E-08	AC090114.2	-0.727595816	3.32E-09
URB1	0.747267295	1.72E-19	CELSR2	-0.727658359	1.68E-07
PRMT5	0.746872567	6.48E-47	HERC6	-0.728890475	2.91E-07
MRPL50	0.746647624	3.74E-27	LRP1	-0.728965198	0.000272085
SNHG21	0.745809015	0.00254813	KIF13B	-0.729119751	3.33E-11
TYW3	0.744126467	5.69E-17	ENPP5	-0.730162541	1.11E-05
NOB1	0.743434982	7.69E-42	PMEL	-0.730638134	0.018166583
CCT6P1	0.742934703	0.00045588	ZNF658	-0.731728313	0.014238365
GTF2F2	0.742690015	2.57E-23	THNSL2	-0.73176302	1.46E-09
TMC8	0.741013519	0.003918342	CLEC2D	-0.732032075	0.000211622
BZW2	0.740861991	9.47E-31	SPAG4	-0.732261624	0.003781858
SNHG10	0.740712048	9.10E-12	ZNF596	-0.732736391	0.002413609
CDK5R1	0.739362895	0.001976414	MCC	-0.73420192	0.001214136
AP000648.4	0.739328218	0.049371246	PTPRE	-0.73482253	1.32E-06
FOXO3B	0.739050671	4.75E-05	PRPF40B	-0.734835336	6.31E-07
NAF1	0.738408543	1.80E-14	CHMP2A	-0.73515233	4.94E-22
WDR35	0.738101343	1.31E-14	JAKMIP2	-0.735468958	0.019248463
PRMT1	0.737832325	7.39E-42	BCAS1	-0.73599539	3.87E-25
SAPCD2	0.737829969	2.15E-37	KRT7-AS	-0.736173315	0.02113065
CCDC88C	0.737157902	5.46E-22	CMPK2	-0.736352523	0.005619612
NFIX	0.736498354	1.79E-10	C17orf82	-0.736489198	0.008652504
C5orf30	0.735869348	1.58E-17	S100A14	-0.736537803	7.50E-05
PES1	0.735632461	3.94E-48	PLD1	-0.736720237	0.000453442
PDCD11	0.735004333	1.77E-30	KRT80	-0.736879285	9.68E-29
DLX1	0.734949637	3.10E-08	PALM2AKAP2	-0.73845072	0.005686575
TAF5	0.734195065	1.67E-10	KRT18	-0.738819287	1.80E-07
MRPS12	0.734144018	3.53E-30	HLA-DQB1	-0.738862115	3.38E-09
DIAPH2	0.733168145	3.55E-11	PLTP	-0.73891163	0.027524001
PARVB	0.73284775	0.000352986	LYPD6	-0.739115727	8.86E-12
AK6	0.732520992	5.29E-24	TK2	-0.739566241	6.07E-05
TIMM17A	0.732256744	1.60E-30	DYRK1B	-0.739625818	2.40E-10

DCTPP1	0.732183512	1.60E-45	C2CD4C	-0.740199328	0.013999747
GTF2H2	0.731883373	5.98E-24	SMOX	-0.74053881	0.01883898
GTPBP4	0.731556938	2.15E-44	ANXA3	-0.741174798	1.91E-13
ZBTB24	0.731542727	2.18E-18	ULBP2	-0.742683371	1.24E-07
GEMIN4	0.729835355	2.56E-29	SEMA4F	-0.742890643	2.16E-05
PIM2	0.729627225	1.22E-08	CMAHP	-0.743056044	0.022576642
FBL	0.729572063	8.90E-29	NAP1L2	-0.743858604	0.009223137
LRIG3	0.729384509	2.31E-07	GNG7	-0.744117315	0.003040604
FASTKD3	0.728269348	5.59E-10	SLC28A1	-0.745003047	0.033829282
MARC1	0.72800537	4.32E-11	RIN2	-0.745016656	2.15E-09
CDC123	0.727912027	5.62E-38	CBLB	-0.7452978	7.70E-08
SLC19A2	0.727582411	3.81E-15	LINC01521	-0.745313705	1.49E-05
PAM16	0.727290932	1.16E-19	ULK1	-0.745585791	5.10E-33
C15orf61	0.726597172	1.22E-06	FAM214B	-0.745650971	0.003084751
RAB29	0.726324167	1.22E-14	FXYD3	-0.746134433	3.95E-23
ICOSLG	0.726075673	1.59E-05	C9orf106	-0.746367805	0.030582129
FBRSL1	0.725535022	8.88E-41	PHLDA1	-0.746969346	0.000867991
DOHH	0.724705376	1.30E-12	BAIAP2-DT	-0.747562824	2.77E-12
DUS3L	0.72438753	1.55E-20	AC159540.2	-0.747750039	0.034678204
RPRD1A	0.723692555	1.94E-32	AMZ1	-0.748095396	5.07E-08
CHAC2	0.722801264	4.75E-10	F2R	-0.748321257	0.001594142
NOP2	0.721986035	1.66E-38	WDR78	-0.749235196	0.020841609
CEP83	0.721731988	1.13E-14	PTGR1	-0.750683479	0.005280979
UTP4	0.721097922	9.28E-35	ALDH1A3	-0.750686453	1.77E-09
AHSA1	0.721058811	4.18E-49	MYLK	-0.750742604	0.011765441
MRPS30	0.720452874	2.08E-24	GABRP	-0.751335905	7.51E-10
TUBE1	0.718937189	1.03E-06	SERPINB8	-0.751381579	0.019784783
ARMC6	0.718420257	1.97E-24	ZBED5-AS1	-0.751648548	0.014974447
MEST	0.717714786	4.67E-41	ETV5	-0.751938669	0.005498879
NT5DC2	0.717087161	8.93E-30	PRRT2	-0.75206839	0.000208828
CTPS1	0.715579133	1.00E-34	APOL6	-0.75214333	2.04E-09
SIM2	0.715418752	1.34E-05	HSH2D	-0.752168448	1.33E-07
GNPDA1	0.71522927	6.20E-21	GPR132	-0.752723007	0.049522251
PHB	0.714165409	1.40E-54	PROB1	-0.752767715	0.001143458
AC022966.1	0.712506798	9.21E-29	SLC31A2	-0.752972379	4.08E-09
COA7	0.712484937	1.12E-23	ORAI3	-0.753131761	1.34E-08
THAP2	0.710757278	0.002949135	ZNF396	-0.753200837	0.017705786
TOMM5	0.710733547	2.14E-26	LPXN	-0.753810875	0.048371388
STC2	0.710715154	2.11E-33	CCDC9B	-0.754177844	0.030987532
JMJD6	0.709972297	8.11E-18	TCEAL3	-0.754269538	9.86E-16
POP7	0.709958338	2.90E-31	TOX2	-0.755074503	0.022400592
RPL23AP82	0.709752552	6.78E-09	HEG1	-0.755372006	0.000666111
PEX5	0.709226656	9.06E-27	TRGV9	-0.755632732	0.000215299
EIF1AX	0.708927723	5.97E-39	PRX	-0.756078661	0.000957401
TRMT1	0.708110319	3.62E-28	MB	-0.756431926	1.36E-08
MRPL36	0.707608835	4.27E-24	LDLRAD4	-0.756553382	9.17E-10
EFHD2	0.707592882	2.85E-25	ADSS1	-0.756825926	1.92E-07

PWP1	0.707194252	2.63E-31	MBOAT1	-0.756948421	3.68E-10
SMAD6	0.705149935	9.88E-07	CLU	-0.756951225	1.27E-59
UBIAD1	0.704421954	2.92E-16	FAM167A	-0.756966054	0.000629052
TRNP1	0.703897086	4.24E-06	EMID1	-0.757425587	2.33E-06
ARL6	0.703095749	2.91E-06	LRRC56	-0.757773383	0.002639825
SMG1P2	0.702610491	4.95E-09	LINC01137	-0.758318156	0.015754245
IPO5	0.70230032	5.36E-44	RAP1GAP	-0.758859174	6.69E-13
CLUH	0.701892693	4.76E-33	UGT1A6	-0.759279199	4.04E-08
TAMM41	0.699450559	3.06E-12	LINC01503	-0.759709195	0.000703015
NME1	0.698966608	1.78E-38	KRT8	-0.760119352	2.42E-34
MTHFD1L	0.698456571	3.07E-41	EPHA4	-0.760234734	5.35E-13
REXO2	0.698110291	1.11E-11	PRICKLE2	-0.760277859	0.00665984
IFRD2	0.696929262	9.72E-36	ITGA7	-0.76057162	0.005551066
CSPG5	0.696907603	0.020384418	RTN2	-0.760779583	0.007751881
CACYBP	0.696616027	2.17E-31	IRS2	-0.762406565	1.24E-08
TDRD1	0.695924884	3.33E-14	TNFAIP8L1	-0.762428099	0.00024482
CRACR2A	0.693043304	0.000305841	ADGRA2	-0.762566254	0.004548434
FAM222A	0.692932745	2.16E-12	LOXL2	-0.762641184	1.64E-07
AMD1	0.692905499	5.97E-41	MST1	-0.763296295	0.010052429
EXOSC5	0.692063059	5.87E-21	CASTOR3	-0.763635781	1.97E-10
FAM136A	0.69194404	9.99E-42	AC139099.1	-0.76369449	0.00581445
TOMM40	0.689950637	3.33E-43	SCARA3	-0.763966916	3.63E-17
CEBPZ	0.689786154	1.78E-31	IER3	-0.764346074	0.008509454
UTP25	0.689703446	5.69E-17	ANXA6	-0.764562747	4.67E-31
SLC7A6	0.688977929	7.01E-18	TMEM198	-0.765694304	0.020441502
RSAD1	0.688263354	2.39E-29	MAP2	-0.765849962	0.000325821
TMEM201	0.686962312	1.04E-15	CDKN1A	-0.765969534	7.18E-09
PRMT3	0.686363449	2.41E-19	CHST3	-0.766424617	0.003721493
HSPA4L	0.68636289	3.49E-16	FLT3LG	-0.766851162	0.040904886
TBC1D14	0.685560816	5.89E-23	MRPL23-AS1	-0.766852217	0.005782865
MAD2L1	0.684919599	1.18E-32	SLC66A3	-0.767219636	3.92E-13
NOL10	0.684913307	2.04E-28	IGFBP3	-0.768678541	0.028708204
ACTR3B	0.684336724	1.39E-14	NUTM2A	-0.769087809	0.035404454
NAA25	0.68399609	1.29E-24	PAPLN	-0.769230119	0.00050395
DKC1	0.683946611	1.67E-33	CLSTN3	-0.770106822	4.14E-08
NLE1	0.6819368	3.42E-14	CCDC153	-0.770302388	0.002356503
NDC1	0.681656865	1.98E-25	LINC00514	-0.770399334	0.008849367
SHISA9	0.681641998	6.96E-15	FBXO48	-0.77040864	0.020375685
GPR135	0.680676866	0.020435896	AC110285.6	-0.770485397	0.03466857
SLIRP	0.680493942	1.08E-15	PSCA	-0.770685398	1.35E-06
PDCD5	0.680020132	7.62E-25	OPLAH	-0.771271569	1.82E-05
ANKRD16	0.679440807	2.03E-06	IL17RC	-0.771276175	1.37E-17
PA2G4	0.678674691	1.48E-51	AC126564.1	-0.771355238	9.53E-06
MEMO1	0.67786767	1.23E-19	NBPF4	-0.771547255	7.06E-05
ZNF778	0.677230021	1.54E-12	ZNF555	-0.771558606	0.00090212
PGAM5	0.676230804	8.82E-34	EPOR	-0.772103972	3.66E-06
NR6A1	0.676226851	0.001046373	WBP1	-0.772194165	2.28E-25

MRPS26	0.675812805	7.66E-25	FAM47E	-0.772406526	0.006423099
LYRM7	0.67546603	5.47E-10	SH3PXD2A	-0.772838607	0.010396251
PRKAR1B	0.674755618	2.18E-26	ZNF610	-0.772969291	0.026541533
DANCR	0.674388348	1.04E-24	AL139385.1	-0.773955918	0.0199304
RRP12	0.672522277	8.28E-32	PYROXD2	-0.774241854	1.45E-06
SRXN1	0.672223226	2.82E-24	USH1G	-0.774482591	1.07E-05
FAM210A	0.672056795	1.32E-18	PPP1R18	-0.775621162	9.43E-13
SLC5A3	0.671988738	1.59E-16	CACNG4	-0.775657278	7.90E-18
IL15RA	0.670707119	1.01E-06	GPX3	-0.77568785	1.41E-07
ATP11C	0.67063192	5.02E-23	EHD2	-0.776167779	3.29E-05
TEAD4	0.670097971	1.17E-13	KLF6	-0.776377152	1.20E-11
EIF3B	0.669926196	1.81E-50	ADAMTSL5	-0.776664876	6.15E-05
RRP15	0.669646465	1.17E-14	FGF12	-0.776916214	0.00516551
ZNF598	0.669579201	9.59E-34	ARSD	-0.776976641	1.98E-24
UNKL	0.669474676	3.23E-15	LMNTD2-AS1	-0.778337979	0.002544187
DTX4	0.669135995	5.40E-09	CLDN9	-0.779242439	1.07E-11
CAD	0.669107005	1.08E-24	WNT4	-0.779254322	0.026731554
LARP4	0.669080435	2.78E-36	DNAJC4	-0.779834108	5.54E-12
GLB1L2	0.668859002	3.86E-14	GMPR	-0.780011375	0.003749388
NEIL2	0.668179799	6.61E-06	UBA7	-0.780565678	7.04E-07
MTFMT	0.667806801	3.80E-12	AC079414.2	-0.780624079	0.01642161
POP1	0.66780153	3.48E-24	MAGI2	-0.781194158	0.001949409
ABCE1	0.667656095	1.97E-37	LINC01569	-0.781783217	0.001321259
DLAT	0.666858465	4.31E-26	PAQR7	-0.782201022	0.000379669
IL27RA	0.66590957	3.31E-13	TTYH3	-0.782441582	2.91E-18
GTPBP3	0.665839566	1.21E-16	SGMS1-AS1	-0.782488257	0.004580459
PCGF1	0.665479007	2.14E-11	TMC4	-0.78289845	9.99E-17
MRPS6	0.664566026	9.27E-20	AC022034.1	-0.784407319	0.000243437
PPAN-P2RY11	0.664173227	0.001146879	BTG1	-0.785565868	6.10E-38
AK4	0.664085782	2.99E-16	OPTN	-0.786525202	5.43E-19
LRRC58	0.662153894	1.51E-33	GSTM4	-0.786562641	1.68E-16
AC105339.2	0.661260648	0.033420199	RNF223	-0.787041879	0.000417988
LRRC59	0.66074248	2.66E-42	TMEM40	-0.787235428	0.000135403
RBM19	0.659644873	5.19E-24	CMYA5	-0.787275738	6.69E-07
BYSL	0.659484153	2.52E-24	RRAS	-0.787375323	3.28E-09
ZNF593	0.659192392	1.67E-11	FMN1	-0.787534877	3.25E-06
COQ3	0.658938979	5.07E-09	LTBP2	-0.787647891	0.00090529
SETDB2	0.658003397	2.09E-05	TM7SF2	-0.78783865	3.69E-29
TATDN2	0.65784983	1.76E-26	KIFC3	-0.788082593	2.24E-11
TSEN2	0.657562669	1.02E-16	LINC00461	-0.788461231	0.030653274
ISCA1	0.657372473	3.92E-21	AP001816.1	-0.788628121	6.71E-09
UCHL5	0.656896368	9.06E-27	AMOT	-0.788728356	0.038763417
GPR63	0.654342078	0.026923528	FUT8-AS1	-0.790539352	0.037828589
METTL5	0.654135034	1.99E-16	DDX60	-0.790897286	1.89E-09
CHCHD10	0.654022072	4.31E-13	CCDC170	-0.791385765	0.000703467
SPHK1	0.653922816	4.17E-18	FBXO41	-0.791807878	0.001575833
TSR1	0.653833316	4.56E-39	IRF7	-0.792011642	1.03E-08

DESI1	0.653576224	7.50E-25	SEMA3C	-0.793574855	3.08E-35
MRPL1	0.653168469	3.39E-17	SLC2A10	-0.793888998	8.91E-23
FUT10	0.652687535	0.000382503	EDIL3	-0.793920861	1.02E-07
TBRG4	0.652205983	1.86E-28	ZSWIM4	-0.794085774	0.000205466
YBX3	0.651663624	3.48E-43	FAM110C	-0.794717593	7.89E-19
OAF	0.651585818	2.65E-07	ADCY5	-0.795372462	4.06E-07
ID2	0.651056533	9.88E-08	PLXND1	-0.795434792	4.63E-22
KANK1	0.650149372	9.40E-12	PLEKHA6	-0.796289347	6.74E-20
KAT2A	0.649935355	2.62E-24	BTN2A2	-0.796435736	0.000112086
COX10	0.649825093	1.59E-09	PDGFB	-0.79777257	1.59E-11
RCL1	0.647504157	4.33E-13	LOXL1-AS1	-0.797948328	0.005503294
CLNS1A	0.647058387	4.00E-30	DUSP10	-0.798418836	0.002409115
SIX1	0.646599258	1.51E-08	SDCBP2	-0.79943072	0.037202602
NOL8	0.646531076	2.38E-26	CMTM3	-0.799572787	0.025254715
MRPL4	0.646005311	3.76E-33	KRT81	-0.800761387	3.09E-49
EBNA1BP2	0.645556353	2.52E-30	GSTM2	-0.802363223	0.019682676
NSUN2	0.64554078	2.38E-37	AC079848.1	-0.802798488	0.011184355
SELENOI	0.64537985	7.50E-24	GFRA1	-0.802965309	0.002403961
SPATA5L1	0.645080593	4.95E-07	PRRT3	-0.803740795	1.53E-18
ATAD3A	0.644924087	1.70E-17	PLA2G10	-0.803901235	0.003766232
KLF16	0.644922279	9.06E-18	UPP1	-0.803964318	5.22E-06
TMEM33	0.643289182	6.44E-22	SARDH	-0.805164829	0.014330434
KDM2B	0.642381152	8.21E-24	TXNIP	-0.805426489	4.37E-57
EIF5A	0.64183286	4.23E-48	MYOF	-0.805734776	1.01E-54
ALDH1B1	0.641526638	8.07E-16	ENTPD2	-0.806152839	0.006597109
CDK8	0.641227845	2.99E-16	H2AC13	-0.807129558	0.010143263
SNHG17	0.640948807	1.50E-14	HDAC6	-0.807640599	1.85E-19
RSL24D1	0.640135948	3.50E-37	IQCN	-0.808725254	0.048186707
ATXN7L2	0.640072215	2.47E-07	SYT10	-0.809460794	3.61E-12
NOCT	0.639892259	6.24E-06	RHOB	-0.809622866	9.76E-34
ZNF30	0.639687849	0.000416248	TJP3	-0.809707979	6.11E-13
TBC1D30	0.639486887	7.90E-25	MAOA	-0.809917455	0.013798134
ME2	0.638973023	8.25E-13	PSMG3-AS1	-0.810180239	6.77E-19
NAGPA	0.638851835	9.24E-12	LINC01135	-0.811572742	0.025973819
EMC8	0.638569294	2.14E-22	TP53INP2	-0.811876299	6.21E-06
RP9P	0.637912585	1.49E-05	PTAFR	-0.811955404	0.00481118
ARC	0.637355817	0.011205026	H3C6	-0.81196697	9.57E-05
MRM1	0.637175438	7.61E-07	AL022069.3	-0.812958038	0.023672705
CBR1	0.6353054	1.54E-27	ALS2CL	-0.81406633	8.56E-08
POLR3E	0.633968854	3.00E-24	MX1	-0.81563036	0.003531459
LINGO1	0.633767421	5.71E-05	AC021066.1	-0.815734342	8.33E-35
THUMPD2	0.632834521	1.57E-06	NEDD9	-0.817081303	1.91E-06
SKA3	0.63230629	7.78E-21	BAALC-AS1	-0.817107942	0.003653395
IKZF5	0.630870913	4.22E-13	PLA2G4C	-0.81755041	0.00104919
KBTBD8	0.630448143	2.84E-05	ESR1	-0.818183825	8.70E-07
URB2	0.629812688	5.59E-16	SFXN5	-0.818350988	3.16E-10
ABCF2_2	0.628624907	6.21E-31	AL109918.1	-0.820590998	0.000449557

SUV39H2	0.628441635	3.83E-17	SLC16A4	-0.820939621	0.030051271
ALKBH2	0.628439093	5.09E-15	STX1B	-0.821644469	0.010184143
GNL3	0.627891889	5.21E-31	NTN4	-0.821869219	0.001761234
RINL	0.627411568	0.013416366	RAI2	-0.822021885	0.027487525
WDR77	0.627174645	3.64E-28	ZG16B	-0.822705507	3.05E-11
NAA15	0.626973137	2.16E-31	MID1	-0.822822269	9.59E-21
NXT1	0.626577404	3.55E-13	CYP4F11	-0.823045335	0.006315896
AATF	0.625790992	5.26E-22	Z97634.1	-0.823189479	0.014794489
SNAPC4	0.625714539	1.74E-17	GLDN	-0.823338265	0.009080118
UBE2S	0.625631806	3.63E-27	SSPO	-0.823501385	0.003847075
UTP15	0.625424244	2.56E-16	ZNF599	-0.823618594	0.012545643
AC027228.2	0.624963193	2.76E-05	STARD13	-0.82398334	4.72E-06
MRPL3	0.624436867	7.66E-34	IL17RD	-0.824039671	0.030395553
NUDCD1	0.62441637	2.38E-30	LINC02732	-0.826150057	1.26E-10
CFAP157	0.624124431	0.021664837	AC015712.6	-0.827662048	7.38E-11
FP565260.3	0.6239515	0.0004123	HTR7P1	-0.827794693	4.36E-05
RFK	0.623804365	2.51E-21	KRT7	-0.828883515	5.29E-24
TFAM	0.623307174	1.45E-27	AMPD3	-0.829084924	0.036126793
NIFK	0.622533342	2.06E-21	PBXIP1	-0.829238903	4.64E-21
SMAD1	0.622442563	4.05E-10	MEGF6	-0.830056906	8.21E-19
TRMT5	0.621321138	3.38E-16	C8orf58	-0.830337758	8.94E-08
GCFC2	0.620535994	4.67E-09	LMO7	-0.830339422	2.44E-26
MNX1-AS1	0.620392718	4.14E-05	CCDC69	-0.830366803	0.00132017
OXNAD1	0.619735564	3.29E-09	RAB11B-AS1	-0.830418892	0.026542754
WDR75	0.619632276	8.79E-21	ABTB1	-0.830673977	5.51E-09
MRPS25	0.619072199	1.98E-26	RIMKLA	-0.83082357	0.024169049
AL359922.1	0.61762421	0.047021199	PADI1	-0.830824967	0.034729747
GPATCH4	0.616168421	2.14E-20	GPRASP1	-0.830983417	0.009766763
COLGALT1	0.61612888	1.54E-30	HIVEP3	-0.831168068	6.81E-06
FAM162A	0.615583627	1.82E-12	KCNAB3	-0.831827074	0.043237448
POLG2	0.615516299	4.05E-14	CLGN	-0.832321692	0.02723187
CCDC138	0.615458677	1.80E-06	IRF9	-0.832333003	6.51E-20
FKBP7	0.614600308	0.038440008	RPS6KA2	-0.832459449	2.11E-07
OTUD6B	0.614542666	1.83E-16	TIMP2	-0.832973783	1.42E-21
DDX51	0.613802785	2.55E-10	SNX33	-0.833081538	2.57E-06
ZNF695	0.613625685	0.008497997	SCN1B	-0.833307932	0.001204186
NUP35	0.613160708	8.46E-11	FAXDC2	-0.834196098	0.038440008
MTRR	0.612763962	1.86E-15	BCAM	-0.834539085	7.44E-19
NCLN	0.612172523	3.32E-20	PPL	-0.834734238	2.83E-55
B4GALT5	0.612155641	2.34E-25	CCDC159	-0.834791576	4.16E-05
ZNF511	0.612064791	6.73E-14	SAMD9	-0.835084133	8.58E-19
SNHG15	0.611675326	7.02E-06	ITGB4	-0.835736144	1.07E-30
XPO4	0.611452158	4.39E-12	IFI6	-0.835971518	2.29E-05
JADE1	0.611128648	4.59E-13	MAPK11	-0.836257441	2.77E-12
SH2D5	0.610672516	0.021356289	AC068580.3	-0.836274152	0.005573908
RAI14	0.61058517	6.37E-12	CAMK2N2	-0.838628792	4.22E-05
POLR3D	0.610446289	1.91E-14	SLC25A42	-0.839058633	1.49E-08

NDUFAF5	0.610331573	7.34E-08	FAR2	-0.839128366	0.042952522
TOMM34	0.61032017	3.44E-26	HLA-DRB5	-0.839594644	0.005530151
APIP	0.610174761	2.67E-09	LNCOC1	-0.839757456	0.019415318
DDX39A	0.60977119	2.91E-36	TMEM125	-0.839920065	9.71E-08
ARHGEF4	0.609589319	0.008165741	SYTL4	-0.840647068	0.000110573
MRPS23	0.609536967	3.67E-26	NOX5	-0.84179546	0.018168319
PPT2	0.60945226	3.09E-10	ALPK3	-0.842125421	2.69E-07
FAIM	0.609042836	5.99E-08	CCN5	-0.842592943	1.83E-14
EBPL	0.608867813	1.74E-16	MRAS	-0.843441358	0.01318507
CHD1	0.608712351	2.24E-23	SOWAHB	-0.843659842	3.43E-06
TRIP13	0.608035103	9.03E-18	DCDC2	-0.845013858	3.96E-23
PTRH2	0.607053867	1.92E-23	LINC02015	-0.845078592	0.001380928
UBE2O	0.606951612	2.32E-20	MAB21L4	-0.845353567	2.02E-10
CCDC137	0.606421232	2.15E-20	RAB43	-0.845425501	0.01247616
YARS2	0.605958263	2.35E-10	ARRDC3	-0.846438922	3.66E-06
ZNF587B	0.605922499	1.48E-06	MYO7A	-0.846449425	0.027371772
HSPBAP1	0.605699656	0.000570686	PLEK2	-0.846877973	0.000688518
MCRIP2	0.605671812	1.72E-13	CCNG2	-0.847002553	2.43E-21
ZNF330	0.605543751	9.56E-18	TSHZ3	-0.850195786	0.000488337
ALKBH8	0.604334849	6.81E-08	ATP6V1B1	-0.851515993	0.032528897
MRPS17	0.604170192	1.10E-07	SHISA2	-0.852110975	0.001624779
ETS2	0.60387076	2.07E-10	LEPR	-0.854507704	0.044379636
DDX31	0.603771679	8.34E-17	IQCD	-0.85484047	0.001620622
TMEM70	0.603764211	6.70E-15	B4GALT1	-0.855516074	7.46E-11
F12	0.603603443	4.29E-12	AL021392.1	-0.855716916	0.036065899
ATP13A3	0.603372106	1.03E-23	RARA	-0.855922004	6.48E-05
MCAT	0.603221446	6.05E-15	BMERB1	-0.857534247	8.06E-05
DCAF1	0.602936096	1.97E-16	TMEM229B	-0.858915557	4.73E-12
NAT10	0.602864537	1.17E-27	GRIN2D	-0.859149111	0.000191222
ZDHHC18	0.602295898	3.70E-15	LINC01963	-0.859905826	0.000291568
CDCA7L	0.602155215	3.41E-13	FER1L4	-0.860908317	9.61E-07
STK26	0.601948194	3.88E-23	COL6A2	-0.861510285	0.000243889
MRPL14	0.601683415	3.97E-23	TUBB8P7	-0.861969001	0.02291124
NPIPB12	0.601571829	1.09E-07	APAF1	-0.862631124	2.85E-07
EIF5	0.601472548	2.15E-30	A4GALT	-0.862643905	0.019161368
AK2	0.601366212	4.00E-32	PCDHA12	-0.862737218	0.014808228
NETO2	0.600818694	1.23E-21	PADI2	-0.863453608	0.004699916
ANKRD27	0.600717838	2.10E-25	VSIR	-0.864071819	0.013648696
MGST1	0.600377194	1.03E-20	ATP2A3	-0.864403163	1.89E-53
BAZ1A	0.599802249	7.73E-35	F3	-0.864471639	0.000103256
TIMM13	0.599790137	2.61E-13	AC144831.1	-0.865045614	3.62E-05
IMP3	0.599374341	5.18E-19	KREMEN1	-0.86506355	8.34E-09
NAA50	0.599287118	3.04E-37	FAT4	-0.865063784	7.47E-05
SHLD2	0.599060178	6.17E-19	ELFN1	-0.865329176	1.42E-05
ADGRL1	0.59900817	3.99E-28	PPM1K	-0.866103286	0.018908518
NFIA	0.599005882	9.72E-07	SRR	-0.866624624	7.29E-09
NOP58	0.598833214	1.36E-28	LDB3	-0.867851854	0.013222972

GALK2	0.598269049	1.74E-08	LOXL1	-0.868370717	0.036123142
NOM1	0.598167276	6.07E-23	TNFAIP2	-0.869374633	0.000329097
LRFN4	0.598069154	9.65E-24	NHS	-0.869753624	1.28E-12
QTRT2	0.598060822	1.55E-22	SLC22A18	-0.870006811	5.35E-11
NUS1	0.597735553	1.80E-25	ESPN	-0.870547014	3.48E-17
SMIM13	0.597546746	5.66E-11	CORO1A	-0.87313455	5.18E-14
B4GALT3	0.59731909	4.93E-16	MT1F	-0.874046476	0.008563142
ENOPH1	0.59728863	2.03E-24	FAM114A1	-0.875568612	7.12E-06
TIMM22	0.597179341	4.19E-14	AL670729.3	-0.875626194	0.021514485
KDM1A	0.595978452	3.72E-27	EFR3B	-0.875660566	0.000539962
RIPK2	0.595861574	9.79E-14	NOXA1	-0.875689436	1.59E-08
TFRC	0.595748186	2.75E-35	LAMB3	-0.875709346	2.11E-06
CUL4A	0.595725539	8.88E-25	DDX60L	-0.876639944	0.000124277
RRN3	0.595692459	7.16E-26	TTLL7	-0.876691894	0.023023146
XPO6	0.594213794	5.48E-32	SBK1	-0.877592791	4.95E-07
TRMU	0.593742454	7.01E-18	SLC4A3	-0.879108364	0.001345417
AC068547.1	0.593526105	2.00E-05	ARHGAP33	-0.88007469	7.79E-10
SNRPD1	0.593028966	3.15E-22	PIP5KL1	-0.881098512	0.005214447
FAM72A	0.592547081	1.96E-07	AC144450.1	-0.881269476	0.000577247
UTP6	0.592492886	1.98E-15	XBP1	-0.881308006	0.000969922
CCDC85C	0.591685904	2.77E-27	MAST4	-0.883462946	1.51E-27
DBF4	0.59165275	8.75E-23	TCHH	-0.884655032	0.01013653
RELT	0.59125709	3.31E-08	CPHL1P	-0.885097084	0.04093286
TRIM65	0.590566585	9.12E-18	FRMD4B	-0.885443687	1.09E-06
NME1-NME2	0.590408373	0.000775985	AC015802.6	-0.885589564	0.004121639
METAP2	0.590303324	1.88E-23	HSPB8	-0.886056406	0.000299315
KARS1	0.589553778	6.60E-40	MARCKS	-0.886853645	9.96E-09
AC113189.4	0.588986664	0.02362539	APCDD1	-0.887353436	0.00038554
RNF126	0.588946497	1.24E-15	TUBB3	-0.887913661	1.95E-26
NAMPT	0.588406667	2.49E-31	VPS37D	-0.888249903	0.001949664
TNPO2	0.588363355	7.78E-28	SPEF1	-0.889025939	0.041604725
WDR36	0.588344366	2.19E-17	C14orf132	-0.889159151	0.00103081
INTS10	0.588291199	5.87E-17	TUBA1A	-0.889228586	4.67E-23
PRPS1	0.588195336	1.04E-23	SOCS1	-0.889532276	0.002597811
GPT2	0.588187036	5.19E-22	TAT	-0.892097855	0.028085817
TSFM	0.587517147	5.47E-18	BTC	-0.892154176	8.38E-07
TENT4B	0.586791404	2.78E-10	STEAP4	-0.892786021	5.92E-05
MRPL15	0.586742728	1.10E-17	TFAP2A-AS1	-0.894921851	0.040227528
ATP1B3	0.586625847	4.91E-24	AC099343.4	-0.895145042	0.015036351
GART	0.585354871	1.13E-30	PRR36	-0.89516714	0.002618507
			ALOX15	-0.895619016	1.46E-13
			COL5A2	-0.896770474	5.48E-05
			RAB4B	-0.896860123	2.25E-06
			CEACAM5	-0.897748252	1.55E-05
			IGFBP5	-0.897769122	6.99E-33
			PXDC1	-0.89890327	2.10E-06
			ITGA5	-0.899098173	4.38E-12

	SCNN1A	-0.89930545	0.016447154
	RAP2C-AS1	-0.899457702	0.006347046
	NES	-0.907119437	7.50E-05
	KRT19	-0.907144891	7.24E-45
	ZNF185	-0.907466832	1.53E-28
	ST8SIA6	-0.908799223	0.002225865
	RASD1	-0.910110453	0.011682666
	POLD4	-0.910119702	4.13E-14
	TMEM45B	-0.910662938	1.41E-10
	SCIN	-0.911223324	0.010649133
	MKRN2OS	-0.911456062	0.030987532
	CTSD	-0.911553532	3.62E-07
	TGM1	-0.911560981	2.16E-09
	AFAP1L2	-0.913352668	4.53E-06
	NEURL1B	-0.913437015	9.30E-20
	LRRC46	-0.91645566	0.002289924
	ABCA4	-0.91689467	0.000333493
	CNTNAP1	-0.917252594	0.026000291
	SPATA6	-0.917669096	0.006005605
	HMOX1	-0.91823255	0.000267028
	SLFN5	-0.91884027	1.30E-09
	C19orf33	-0.919487621	4.72E-11
	PAPSS2	-0.920168926	5.62E-50
	AL031123.3	-0.921693992	0.035843553
	MICAL1	-0.922077579	7.60E-14
	NATD1	-0.922314625	2.23E-08
	PLA2G6	-0.922499075	4.07E-07
	TENT5B	-0.923333853	0.001020286
	TGFBR2	-0.925346913	3.14E-06
	TNFSF15	-0.92714817	4.21E-07
	MIR210HG	-0.927631678	0.000880424
	ZNF365	-0.927758561	0.000156384
	ANKRD6	-0.928236186	0.001929136
	AC006372.1	-0.929815072	0.025823332
	IGFBP4	-0.929961853	0.015212964
	COL12A1	-0.930340507	5.06E-09
	HIPK1-AS1	-0.930398405	0.02454828
	APH1B	-0.932146126	0.000102133
	SYTL2	-0.932349201	4.69E-69
	LGALS1	-0.933914424	1.52E-26
	ABCA7	-0.934140771	4.27E-09
	CATSPERG	-0.934725535	0.006350276
	CCDC96	-0.934943561	0.003440927
	LINC00885	-0.936047571	0.007344663
	NRCAM	-0.936961508	8.71E-32
	PCDHAC2	-0.936969893	0.000375259
	ZIC4	-0.937840779	9.40E-05

NAV2	-0.938627125	1.36E-05
GPC2	-0.943179638	0.011444855
DDAH2	-0.946126132	4.09E-17
EPGN	-0.947255487	0.003354853
UNC5A	-0.948164384	0.006318087
MUC20	-0.949197183	2.34E-12
ITGA2	-0.949298106	1.98E-26
THSD4	-0.949925702	3.65E-14
CYP26B1	-0.950745335	0.000743853
AC245297.1	-0.952202386	0.011671937
AC131649.2	-0.954238654	0.010462853
RBM11	-0.956278834	0.005168278
CFAP206	-0.956410885	0.001116629
RNF224	-0.956486041	0.000100294
MATN3	-0.957722901	6.01E-05
KLF8	-0.959313325	0.00660486
LRRC75A	-0.960057692	0.000607584
PLEKHG2	-0.960832361	5.98E-21
C1QTNF6	-0.961066861	6.94E-73
THBS1	-0.96183383	2.75E-29
GPRC5A	-0.962604378	8.93E-61
CLCF1	-0.964839951	1.27E-06
COL5A1	-0.964890541	2.34E-29
DKK1	-0.965106694	6.59E-24
GGT6	-0.965125294	1.12E-11
SLC22A17	-0.965287804	0.000212299
SLC16A2	-0.967937295	3.24E-05
FRK	-0.969163922	0.000463501
CCM2L	-0.969907506	0.004418907
CCN2	-0.970509163	0.000550326
GRIK3	-0.970577844	0.015950171
CLDN1	-0.970677378	1.98E-15
FILIP1L	-0.971402588	0.00033001
LMO2	-0.973674471	0.000457058
PLEKHG1	-0.973697891	0.005027543
GYG2	-0.974273811	0.002844995
KIAA1210	-0.974522723	0.000692469
KIAA1324	-0.97559232	9.72E-27
PLAAT4	-0.975654817	0.010197497
MYPN	-0.975717617	1.41E-12
GLRA3	-0.976222203	0.002156324
WNT9A	-0.977039934	0.000908026
MGP	-0.977965158	5.31E-16
BEX5	-0.978170633	0.004910045
HDAC11	-0.978908375	9.02E-11
CEACAM6	-0.981654731	1.16E-44
CGNL1	-0.985409483	4.16E-06

	SP2-AS1	-0.986404505	7.04E-05
	ZNF175	-0.987528558	2.11E-06
	AC092070.2	-0.9878503	0.007175746
	SPNS2	-0.989736402	1.75E-05
	ITGB2	-0.989936861	0.011636236
	MYZAP	-0.993824444	6.75E-08
	DNAH7	-0.996547599	0.010094796
	RASSF8-AS1	-0.997448332	0.000233073
	AGAP11	-0.997476176	0.002638907
	CRAT	-0.999743647	1.79E-05
	CLIP2	-1.000375347	3.14E-05
	MT2A	-1.002310207	0.019641429
	DEGS2	-1.003934734	0.011124382
	MUC20P1	-1.00511966	0.009712922
	MYEOV	-1.006597408	3.42E-26
	PRODH	-1.006908203	0.003316981
	AOX1	-1.007387605	0.021040369
	LRG1	-1.009739302	0.002770209
	MSH5-SAPCD1	-1.010746582	0.005499358
	FGD3	-1.011445955	2.49E-07
	JPH2	-1.011677544	4.67E-24
	GDF15	-1.012707475	3.34E-19
	SPEG	-1.013734108	1.72E-05
	ARHGEF40	-1.014691298	0.002787701
	ACKR3	-1.014748582	1.51E-14
	ULBP1	-1.014902551	1.65E-06
	GMDS-DT	-1.01563784	0.008623212
	INPP4B	-1.015659185	1.83E-37
	KRT15	-1.017124601	7.80E-14
	MMRN2	-1.017127546	0.017757604
	PTK6	-1.017525176	1.44E-17
	ENTPD3-AS1	-1.017676746	0.002451884
	CASTOR1	-1.02317211	0.000753462
	AQP3	-1.023710999	3.01E-05
	STX16-NPEPL1	-1.026620349	0.00853654
	FN1	-1.027265876	3.65E-08
	MAFF	-1.027516361	1.42E-06
	MICB	-1.027600662	0.000156074
	LINC00482	-1.028147457	0.024949091
	AP003419.1	-1.028257478	0.00367535
	FOS	-1.029443531	6.36E-07
	ETNK2	-1.030430167	6.12E-17
	MEIS3	-1.031845616	3.09E-10
	LHFPL6	-1.032891031	0.010408788
	TCIM	-1.033124138	6.39E-10
	TMEM8B	-1.034156251	5.23E-05
	SLC22A18AS	-1.034917561	0.001026369

TH	-1.035073958	0.00293351
ANOS1	-1.036088482	0.000181106
ZMAT1	-1.039160434	1.24E-05
ABCC3	-1.040930959	1.32E-06
MVP	-1.042143771	7.69E-39
KRT13	-1.042355605	0.001353512
BEST1	-1.044753746	0.000291529
FA2H	-1.04661145	6.42E-06
MYO15B	-1.050691061	3.06E-10
TNFRSF11B	-1.052095807	1.01E-07
SEMA5B	-1.052159136	2.13E-07
CAPN5	-1.052828695	4.54E-23
CALML5	-1.053000036	1.45E-06
APOBEC3F	-1.053626097	0.000299028
BDKRB2	-1.055586632	1.11E-06
VWF	-1.057718185	0.00367246
AL031123.2	-1.058649766	0.000539962
AC110619.1	-1.058945204	7.74E-31
CLIC3	-1.059088039	4.33E-55
GPR39	-1.066282891	0.000241803
MUC1	-1.067411162	7.78E-28
ITGB6	-1.069663137	9.68E-10
DIRC3	-1.070946983	0.002168062
DNAJB5	-1.071395378	9.24E-05
JAG1	-1.072171983	0.002422273
TGFB2	-1.072183358	8.42E-09
GSN	-1.07325871	4.63E-81
LYPD3	-1.074930732	1.66E-28
MIR503HG	-1.075192531	4.92E-13
LIF	-1.081803293	8.56E-14
RIPOR3	-1.083777183	2.66E-11
SUSD2	-1.08394175	1.17E-11
EMP1	-1.087367828	0.000173704
AL157935.2	-1.088466947	0.006606276
LRRC15	-1.091028486	0.001518422
AL158211.5	-1.094558918	0.00014793
MAGED4B	-1.096464787	0.000630182
ZNF750	-1.096600979	1.92E-05
MALRD1	-1.10244636	0.016815272
ABCC13	-1.103322492	0.000423579
ST6GALNAC2	-1.105298463	1.19E-18
DUSP4	-1.10576258	4.08E-12
AMTN	-1.106736625	8.89E-05
DHRS2	-1.107042051	7.38E-13
GRAMD2A	-1.1084185	0.000164905
DGCR9	-1.110793104	0.008074279
SH3TC1	-1.117052997	5.40E-10

	TP53INP1	-1.11863113	5.54E-06
	SLC1A2	-1.120535123	3.80E-05
	LINC02620	-1.130800307	0.005020231
	CAPN9	-1.139872912	7.66E-05
	MAFB	-1.145042248	3.29E-05
	SNAI2	-1.147843156	7.91E-07
	KLF7	-1.153621509	0.000265906
	CYSRT1	-1.154427455	1.11E-13
	RUNDC3A-AS1	-1.154780267	8.46E-10
	CBFA2T3	-1.15897656	0.001405412
	NR2F1	-1.160501052	1.31E-08
	IFITM10	-1.161105631	7.27E-09
	ACTA2	-1.164308165	1.33E-05
	SEMA3B	-1.165338413	9.74E-08
	TNS1	-1.166385526	6.34E-08
	ANKRD24	-1.166778651	0.00265528
	INHBA	-1.168709867	6.73E-13
	ISG20	-1.172308671	9.75E-07
	TRIM29	-1.180709572	8.01E-05
	PRSS23	-1.184859666	3.59E-06
	LAMC2	-1.185477746	3.07E-18
	TIGD3	-1.186317375	0.000506742
	AL590004.3	-1.18688627	0.001424549
	HMCN1	-1.187593834	1.24E-08
	SPOCK1	-1.189143352	1.76E-18
	ABCA12	-1.192185988	0.000331633
	FOXI1	-1.197236327	5.26E-06
	SLC34A3	-1.199937119	0.000177387
	BANK1	-1.200971389	4.19E-07
	CAPN2	-1.205008348	7.39E-17
	ADORA1	-1.206425389	0.001168634
	ZNF709	-1.209920028	0.00197716
	KLHDC9	-1.210016583	0.000237067
	MST1R	-1.234664761	2.33E-08
	PRR15	-1.239670838	6.31E-08
	PHLDB2	-1.240962177	1.14E-07
	AC010735.2	-1.246154289	3.46E-06
	STIMATE-MUSTN1	-1.246918174	0.000188977
	KLK6	-1.249745678	3.53E-05
	C9orf152	-1.251761496	6.17E-05
	IFI27	-1.252260188	0.000103791
	ABAT	-1.254492326	3.54E-51
	DOK7	-1.287210741	0.003256135
	LDHD	-1.292397327	6.78E-08
	PLXNA2	-1.296941146	7.66E-13
	ACTG2	-1.297528785	1.16E-05

	RET	-1.300504238	0.007734095
	SERPINA3	-1.308871453	9.24E-05
	SYTL5	-1.309806811	1.37E-06
	NT5E	-1.313115995	5.48E-44
	AL359258.3	-1.314995883	0.00190009
	SLCO2A1	-1.322100414	4.07E-06
	ADGRF4	-1.334900215	7.58E-10
	CEMIP	-1.334904026	0.032531246
	RASGRP1	-1.340736581	4.34E-17
	AC008556.1	-1.354978053	1.53E-05
	KCNMB1	-1.356655106	1.94E-05
	CISH	-1.362207601	0.00690796
	KNDC1	-1.362323601	5.83E-05
	PAOX	-1.374332656	0.000111863
	MALL	-1.37595949	6.68E-47
	CYP24A1	-1.38200716	0.000184516
	KRT16	-1.388943724	2.51E-17
	KRT17	-1.412793387	6.41E-10
	AREG	-1.453935003	0.001107915
	LINC02747	-1.482761639	0.000415819
	PSG9	-1.485899116	2.36E-09
	PTGES	-1.577125601	3.83E-05
	EGR3	-2.046502579	0.040859521

Table S8: mRNAs significantly up- or downregulated (> 1.5x fold change) in MCF-7/pRTR-c-MYC cells (*AP4* KO/*p53* -/-).

Significantly up-regulated mRNAs			Significantly down-regulated mRNAs		
Gene symbol	Log_2 fold change	padj	Gene symbol	Log_2 fold change	padj
MYC	4.167260266	0	GADD45B	-0.5853533	1.85E-08
AC026786.1	4.124040958	5.17E-239	NLRX1	-0.585709861	2.17E-14
CTRL	3.601294943	9.14E-46	PCYOX1L	-0.585920005	3.58E-11
PDE4A	3.446885938	1.33E-94	CCDC69	-0.585955072	0.003163952
FABP5	3.188506776	3.79E-112	TSC22D2	-0.585961161	1.04E-30
GAL	3.022289043	0	PI4K2A	-0.586362733	4.69E-14
CR2	2.73504122	4.86E-44	SP2-AS1	-0.586726432	0.021529526
HS3ST3B1	2.673459104	8.48E-27	GSEC	-0.58675942	0.000358986
DUSP2	2.5827638	3.24E-71	TMEM140	-0.58680189	1.58E-05
POLR3G	2.551053106	2.92E-253	TRADD	-0.58730785	2.73E-15
PLD6	2.301153868	1.61E-125	ATP2C2	-0.587318979	8.49E-11
TBC1D4	2.205894498	5.40E-200	ATXN7L1	-0.587949727	2.97E-05
SORD	2.189827081	9.41E-68	ERVK13-1	-0.588348351	5.46E-05
HOXC8	2.180284092	5.84E-13	SHANK2	-0.588433595	1.99E-16
KCNQ4	2.15695759	5.08E-19	TBC1D8	-0.588772612	2.26E-13
WNT10B	2.133754824	6.46E-49	BACE1-AS	-0.588872469	0.037903203
CPNE7	2.119732291	5.16E-27	YPEL2	-0.588887105	1.73E-07
TERT	2.112713842	6.38E-12	MIF4GD	-0.589464762	5.46E-12
SLC16A1	2.095697655	0	KDELR3	-0.589599039	2.60E-07
RAB3IL1	2.011081879	1.86E-85	SH3BP5	-0.589788142	1.70E-66
RNF125	1.981591856	4.14E-56	AC026471.1	-0.589869425	0.000487668
PPARGC1B	1.965141668	7.39E-69	TPST1	-0.589878592	0.000218189
SORD2P	1.849616813	4.79E-12	NUTM2D	-0.590107585	0.002897891
AC007342.4	1.847173942	7.08E-10	HES1	-0.59053327	2.31E-22
AC040162.1	1.834654439	1.30E-36	SEMA4C	-0.59084077	6.99E-23
RPP25	1.833185012	1.73E-149	MAMDC4	-0.591072602	1.60E-05
SLC19A3	1.832146424	4.78E-09	SLC6A8	-0.591100966	6.65E-06
ADGRE2	1.823419258	8.02E-26	JAK1	-0.591203571	8.83E-46
SLC7A11	1.811035481	2.31E-70	SAT1	-0.591263177	2.27E-21
TMEM52	1.772524984	2.43E-13	ZNF333	-0.591504839	0.013074569
EMSLR	1.736910924	1.81E-164	GMPPB	-0.59157568	1.42E-15
MIR17HG	1.689194918	8.80E-07	ST3GAL4	-0.591728494	3.91E-31
CMTM8	1.681194148	3.61E-22	ITPRIP	-0.591844318	4.08E-06
ADAT2	1.675636771	1.68E-92	DNAH5	-0.591930982	0.001631734
LRFN1	1.661666047	1.03E-26	AC005229.4	-0.591960909	0.001478488
ACTL8	1.66038723	1.49E-11	LMNA	-0.592138776	1.81E-83
AMER1	1.650959405	2.38E-90	AC005332.6	-0.592601839	9.21E-25
MATK	1.634481721	1.25E-110	PIGQ	-0.592684202	1.57E-21
ID2	1.61588637	3.05E-76	BST2	-0.592890343	1.14E-23
EN2	1.607673494	2.95E-71	TECPR2	-0.593007762	6.96E-15
RGS16	1.605806631	7.41E-67	ISG15	-0.593347328	3.41E-22
DNAH17-AS1	1.601583462	1.53E-08	IER5L	-0.593475648	1.15E-48

PCOLCE2	1.580123972	2.81E-46	IFT22	-0.593976953	2.57E-21
TAF4B	1.576386291	6.35E-110	NICN1	-0.594669566	0.004704443
FJX1	1.568263931	9.44E-63	FAM187A	-0.594918367	0.021889704
PFKM	1.548933199	2.22E-189	KCNAB2	-0.594924225	9.47E-08
PITX1	1.540201322	4.03E-108	ZBTB4	-0.595391011	3.36E-19
PODXL2	1.538909671	7.43E-117	SH3BGRL3	-0.595838127	6.26E-18
ARID5A	1.524734157	2.12E-28	TBC1D3L	-0.596010498	6.30E-06
METTL8	1.51735701	1.81E-113	GLI4	-0.59605901	1.21E-05
ZIC5	1.507387866	6.56E-25	RNF19A	-0.596093619	1.33E-20
HPDL	1.498607336	9.41E-103	ETV5	-0.59611932	1.40E-13
ANKRD13B	1.495856119	6.57E-78	DDB2	-0.596132353	2.15E-07
LYAR	1.485646615	5.45E-143	PHLDA3	-0.596215175	1.35E-15
NRARP	1.484131369	1.53E-133	AC009005.1	-0.596378729	0.019914023
SLC29A1	1.466158087	7.38E-194	IL10RB	-0.596499336	2.16E-12
SNHG30	1.464057589	2.04E-42	MYLK	-0.596733823	0.021761621
MPP6	1.43879113	2.10E-114	FAM214A	-0.596797638	1.42E-27
MLKL	1.422811397	5.80E-88	LUM	-0.597424377	0.006489557
RNF145	1.418391491	4.46E-25	UCP2	-0.597562053	7.19E-25
NLN	1.409250023	2.90E-223	TRIM62	-0.597652698	7.66E-11
GFOD1	1.400559139	6.64E-48	JUND	-0.597797615	1.29E-55
FAM89A	1.399060885	2.88E-16	SULT1A1	-0.59786313	2.03E-10
PPIF	1.397085198	2.32E-232	REX1BD	-0.5980511	1.53E-10
SLC25A32	1.39033954	9.77E-150	SLX4IP	-0.598057678	1.97E-05
CRYM-AS1	1.388592113	3.77E-05	YJEFN3	-0.598158713	5.17E-06
RPIA	1.386003014	4.20E-164	SNX33	-0.59867581	9.31E-05
MXI1	1.385357585	6.10E-102	RHBDL1	-0.598786879	0.001404136
JPH1	1.372662988	1.15E-47	ZFP36L1	-0.598955431	1.28E-25
GUCY1A1	1.371373102	5.30E-06	ERBB3	-0.599100432	1.16E-39
CAMKK1	1.3688632	4.39E-13	RCAN1	-0.599666005	4.21E-18
NPM3	1.367519266	1.92E-126	PFKFB4	-0.59967822	3.98E-06
NR1D1	1.363861388	5.52E-20	SAMD4A	-0.599853144	8.83E-10
AC138894.1	1.363425832	0.029202721	PBX1	-0.600328827	2.27E-37
ABLIM1	1.35873986	3.20E-112	SFXN3	-0.600806322	3.58E-10
IFRD1	1.356575559	5.67E-165	TACSTD2	-0.601153806	1.91E-59
YRDC	1.355305687	1.44E-74	C3orf14	-0.60126027	7.46E-38
NFE2L3	1.35474349	5.64E-38	PGPEP1	-0.6016626	3.00E-18
CHAC1	1.354270954	7.73E-33	SIGIRR	-0.601816606	1.47E-14
CHCHD4	1.354020246	1.17E-93	LAMA5	-0.602178764	3.11E-30
MAP3K21	1.35071825	2.31E-32	ZNF219	-0.602268887	3.34E-09
CHN1	1.349153646	1.31E-58	RAB27B	-0.602303107	1.44E-21
ODC1	1.34108909	2.91E-240	CDK2AP2	-0.602476653	9.88E-28
SENP3-EIF4A1	1.333965057	0.019065022	AL354707.1	-0.603138864	0.008164638
CENPV	1.33126905	1.39E-31	JMJD7-PLA2G4B	-0.603153571	0.000407866
SLC25A19	1.328884592	3.34E-89	ZDHHC8	-0.603236193	0.000556289
CCDC86	1.328627167	1.04E-193	PLEKHO2	-0.603911035	0.006879597
SLCO4A1	1.328541872	1.86E-49	NSUN7	-0.603919766	0.000826469
PSAT1	1.318107973	5.96E-176	RSPH3	-0.604155562	2.42E-07

RRP9	1.317637321	3.08E-109	GAB2	-0.604254813	0.000486918
HHEX	1.31612689	3.55E-12	NDRG4	-0.604504542	0.000167206
TWNK	1.315034634	2.92E-110	VAV3	-0.605255144	8.11E-09
EEF1AKMT4	1.313950782	3.01E-85	ZNF350	-0.606294434	0.015533406
FAM117B	1.296472992	9.21E-40	RASSF8	-0.606401554	7.48E-10
CARMIL2	1.294436393	7.75E-20	JAZF1	-0.607881613	0.003248433
NDUFAF4	1.291106076	1.94E-61	AJUBA	-0.608024739	6.34E-24
ZNF296	1.290168477	4.80E-27	ADAM8	-0.608437805	0.000952282
CACHD1	1.289478629	1.21E-06	CDK19	-0.608491243	2.29E-18
SMKR1	1.287819134	7.46E-37	SLC30A3	-0.608623091	0.005059186
PMAIP1	1.285011907	9.64E-17	AOPEP	-0.608780535	8.67E-15
PSMG1	1.2817345	8.77E-130	CELSR1	-0.608806402	2.95E-19
CDC42EP1	1.281419667	1.66E-160	TMEM121	-0.608815077	1.33E-05
RPP40	1.279165783	7.27E-41	HIPK2	-0.608844747	7.18E-06
DNAJC2	1.267065503	5.45E-156	ZFP14	-0.608966161	0.002244882
RRP1B	1.259631481	1.43E-161	C21orf58	-0.609296012	6.80E-07
RABEPK	1.25810531	1.99E-86	CCS	-0.60943011	3.70E-13
BAG2	1.248647495	4.36E-33	SHLD1	-0.609534128	0.038669878
PNP	1.24517478	1.75E-111	GPR137C	-0.610183786	1.04E-05
SACS	1.239585544	6.51E-75	LMBRD1	-0.610254332	5.22E-19
GEMIN5	1.236984226	1.95E-166	HSD17B1	-0.610598186	3.45E-05
USP2	1.232067123	1.00E-09	RNF213	-0.610713422	6.88E-22
RIOK1	1.230989773	7.95E-84	FRMD6-AS1	-0.610812089	0.025030943
AL358113.1	1.230644965	1.26E-05	ENPEP	-0.61083958	0.029852565
KCTD12	1.22855912	1.48E-22	TGFBR1	-0.610997061	8.52E-26
CDC42EP2	1.220400428	3.59E-11	TSPAN15	-0.611226937	1.35E-40
SLC25A33	1.21792528	7.09E-72	ATP7A	-0.611300455	1.05E-07
AL445423.3	1.217895249	0.000200717	RELL2	-0.611405781	2.83E-06
GBX2	1.217146208	5.33E-08	KIFC2	-0.611661267	3.41E-06
SLC27A5	1.215264061	8.02E-23	CDYL2	-0.611679284	1.80E-32
ANP32A	1.214019006	2.32E-175	LINC01503	-0.611708433	0.019494884
FASTKD1	1.211646286	9.69E-51	PARP14	-0.612703061	1.81E-23
TMEM158	1.204359846	0.000476261	H3C10	-0.612767025	2.54E-05
FAM131C	1.20227226	5.00E-07	MPZL2	-0.613154425	4.25E-41
NTHL1	1.201646025	5.43E-41	RAB24	-0.613372586	5.79E-21
CD3EAP	1.201491748	2.10E-118	MBOAT1	-0.614142198	6.07E-07
GPR63	1.200218768	1.73E-09	MEGF8	-0.614374272	3.52E-16
AP002387.2	1.188215989	1.20E-12	MYH9	-0.614446644	4.16E-16
NAT8L	1.187447028	8.26E-45	AP003108.2	-0.614955883	0.000295036
FAM81A	1.183513753	3.30E-14	FBXO32	-0.615107088	2.57E-05
GALNT18	1.182847752	2.55E-70	FBXO41	-0.615398291	0.002675973
EPOP	1.18076485	6.97E-102	NUTM2B	-0.615574393	0.013424628
LOXL3	1.17980504	3.62E-17	FRMD4B	-0.616003714	9.46E-06
SFXN4	1.175951521	1.51E-63	ERAP1	-0.61611155	2.24E-16
CTU2	1.171517809	1.68E-52	HGSNAT	-0.616247718	8.83E-10
REXO4	1.166925956	6.77E-175	JUP	-0.616554747	2.43E-69
SNRPA1	1.161369807	9.68E-136	SLC2A1	-0.617158782	1.01E-51

TFB2M	1.160086334	5.15E-60	BHLHE40	-0.617296466	2.07E-42
CCDC58	1.158983013	3.89E-49	LRRC27	-0.61734013	7.48E-05
DIMT1	1.158644317	3.67E-122	TMEM170B	-0.617579812	1.75E-07
WDR3	1.157009719	1.99E-155	DUSP5	-0.618060365	2.26E-06
ADAMTS17	1.153179885	8.51E-05	GABBR1	-0.618431201	0.00033958
GALNT14	1.152910618	7.25E-41	SLC16A3	-0.618524425	0.003240731
TRMT11	1.15247867	2.94E-56	AC105219.4	-0.618627758	0.018903225
SUPV3L1	1.151952553	1.93E-79	C3orf67	-0.61869749	0.028651182
DPH2	1.151375564	1.49E-108	STAG3L5P	-0.61876046	0.002367631
MON1A	1.150819064	2.56E-40	NRM	-0.618811986	2.56E-07
ASS1	1.150724438	1.12E-138	MSRB2	-0.618952898	3.34E-10
CCDC85B	1.142523522	1.96E-146	PLCB3	-0.619043638	3.71E-39
CDKL1	1.142014794	0.004947484	TRPS1	-0.619401433	5.79E-38
WDR43	1.141229839	3.46E-185	HS1BP3	-0.619533098	1.65E-09
MTHFD2	1.139864299	7.71E-217	SRR	-0.619847096	7.03E-06
NOC3L	1.139862503	8.80E-91	ZNF548	-0.620079067	0.008071748
GRB14	1.13963617	1.04E-21	PHYKPL	-0.620227829	2.27E-11
NOP16	1.139624389	9.47E-136	ALKBH6	-0.620270701	1.43E-10
DDX10	1.139041075	1.00E-66	RGS12	-0.621018897	7.12E-07
NDUFAF2	1.135357211	1.15E-56	SDCBP	-0.621299083	4.05E-25
GLS	1.132534187	1.24E-146	NAGLU	-0.621402128	2.54E-20
PALD1	1.131302193	1.03E-09	RIMS3	-0.623028619	1.13E-10
DDX21	1.129848318	7.59E-200	GATA3-AS1	-0.623394182	0.003447654
SMG1P6	1.129334212	0.007039814	CADM4	-0.623776403	0.000188954
ASB13	1.129324529	8.44E-54	AC074143.1	-0.623923809	1.97E-06
NOLC1	1.12855734	2.25E-188	GOLGA2P5	-0.623982796	0.000785122
NCR3LG1	1.12845201	0.001036546	SUSD1	-0.624005093	0.000107332
C1orf109	1.127899112	1.15E-45	HTRA1	-0.624116691	0.001632741
ABCC4	1.126918452	1.15E-57	ING4	-0.624284761	6.30E-06
IMPDH1	1.125672746	1.25E-146	TMUB2	-0.624458605	6.04E-09
DCUN1D5	1.124986656	1.24E-108	SLC27A1	-0.624597374	0.012835098
TXLNG	1.124589467	2.21E-97	FLNB	-0.625123253	2.06E-53
ZNF778	1.121465992	9.79E-107	LINC01569	-0.625129904	0.030522291
PNO1	1.118925282	2.28E-83	LINC00205	-0.625140249	7.29E-19
GNB4	1.115156605	6.73E-10	LPIN3	-0.625159864	9.22E-24
PDCD2L	1.113260862	3.12E-41	PKD2	-0.625361774	3.28E-12
JAG2	1.112555718	1.48E-75	GSTZ1	-0.625539903	2.56E-18
RAB3A	1.111105816	4.22E-11	HSD17B11	-0.625870626	0.018497288
EIF2B3	1.111057075	2.13E-46	ATP2B1-AS1	-0.625980836	0.003921009
KLHL21	1.109811794	2.12E-78	GHDC	-0.626529384	2.01E-09
BEND3	1.109173086	1.84E-30	STAG3	-0.626565758	0.007802965
CMSS1	1.107605796	1.45E-83	CASP4	-0.627996308	0.00221885
FBXO45	1.105841787	6.71E-175	DNASE1L2	-0.628095121	0.016753515
UCK2	1.102671853	2.37E-122	HSPB1	-0.628096615	5.18E-55
SLC39A14	1.101795932	6.72E-97	PARP11	-0.628556082	0.000429702
GPRC5B	1.100948537	0.000412286	MPND	-0.628678881	1.67E-09
RPUSD1	1.100852601	9.06E-139	ZNF329	-0.628740299	0.005913125

HOMER1	1.100544512	1.91E-84	BAALC-AS1	-0.629090895	0.03755858
RNASEH1-AS1	1.099838159	2.21E-21	IL10RB-DT	-0.629208568	0.016902677
TASOR2	1.097735049	2.94E-140	ITGA7	-0.629273733	0.031437223
AL024508.1	1.097189477	2.16E-23	FHDC1	-0.629333972	5.02E-06
RPUSD4	1.092946361	2.04E-42	GRIPAP1	-0.629459378	1.30E-23
ZIC2	1.0907191	2.14E-22	PHETA1	-0.629813512	5.35E-08
RP9P	1.09025839	1.87E-16	PREX1	-0.630053813	1.72E-57
FTL	1.08995073	1.01E-129	PDIA3P1	-0.630171059	0.000126996
RRP1	1.0884505	2.41E-105	AGER	-0.630765891	0.013838384
FAM216A	1.088218433	1.47E-19	CSRP1	-0.630823553	8.81E-80
SCML2	1.086309949	3.30E-15	TNNT1	-0.630833855	1.99E-16
POLR1C	1.085840263	1.73E-112	MSRB1	-0.631334909	6.88E-25
COQ3	1.085563152	2.14E-25	MYADM	-0.63134981	5.01E-13
UTP20	1.085131247	1.66E-98	PSD4	-0.631717005	5.43E-26
GRPEL1	1.084353203	7.95E-99	KCTD21	-0.631873547	4.88E-06
DIXDC1	1.078095401	0.000418417	BMP1	-0.631883518	1.85E-12
TIMM44	1.07712375	9.71E-82	TSNAXIP1	-0.632209396	0.049934813
AEN	1.076909945	1.00E-72	SMIM5	-0.632377999	1.01E-06
ZNF639	1.075782985	6.11E-58	LMF1	-0.632838793	8.43E-08
ELL3	1.074357591	1.60E-14	ATP6V1FNB	-0.633029372	0.039542045
MRPS30	1.071575952	4.68E-90	TSPAN4	-0.633077459	4.95E-14
MRM3	1.071418722	2.66E-64	CD82	-0.633318188	3.78E-06
KLHL23	1.070180606	4.75E-23	TMEM143	-0.633427648	0.012107109
HMGA1	1.070037926	6.68E-195	SH3BGRL	-0.633750071	6.06E-35
MAK16	1.067308334	3.33E-67	ZNF641	-0.633995664	1.98E-09
NANOS1	1.063697047	4.67E-15	CKB	-0.634756686	0.000153266
USP31	1.061533404	2.57E-60	LINC00963	-0.635852873	1.59E-25
UBE3D	1.061204402	1.65E-11	TMEM87B	-0.636027155	1.64E-21
TOP1MT	1.06020807	4.27E-90	EFCAB6	-0.636271586	0.045961377
SINHCAF	1.05842097	4.82E-55	ACADVL	-0.6364753	8.31E-46
POLR1E	1.056421658	6.07E-36	LINC01116	-0.636543614	0.013393722
FMNL2	1.053363794	9.74E-28	CCHCR1	-0.637179476	3.33E-08
COQ8A	1.051263535	1.25E-51	FAM66D	-0.637897572	0.047186204
RINL	1.049646002	2.08E-05	ABHD2	-0.638127276	2.62E-22
RSL1D1	1.049026511	6.40E-182	SPSB1	-0.638273823	0.000394863
KAZN	1.049014485	1.66E-42	PTPN21	-0.638385325	4.45E-16
WDR12	1.048691187	1.70E-80	CABLES2	-0.638436842	6.41E-15
PRR19	1.045561127	5.24E-11	GRIN1	-0.638509153	2.73E-05
TRAP1	1.045215884	5.07E-188	SLX1A	-0.63885549	1.52E-05
XPOT	1.042848708	1.81E-236	DOCK11	-0.639133903	1.34E-12
SLC43A1	1.042805281	0.002410094	LINC01089	-0.639473593	0.038539971
OSBPL6	1.040561561	5.19E-14	PDE5A	-0.639582607	2.51E-05
TUBE1	1.037823973	4.57E-12	AC073957.3	-0.639768653	0.002648823
SH2D5	1.036061888	9.57E-07	CYP4V2	-0.640096149	2.41E-06
DNAH14	1.034035978	2.10E-12	ZNF774	-0.640179832	0.00692373
PRR5	1.030188055	1.99E-61	LINC00847	-0.640298283	2.47E-07
LDLRAD3	1.028550037	9.28E-18	GPAA1	-0.640333652	8.55E-50

BLMH	1.027827212	2.17E-49	AC022400.9	-0.640560538	0.019150744
FXN	1.026408568	1.58E-27	ENO2	-0.640579225	2.01E-05
ESF1	1.023815451	2.18E-85	PPP1R15A	-0.640746646	5.15E-06
NT5DC3	1.022080072	2.52E-17	OPHN1	-0.640802473	3.82E-07
KIF9	1.020116553	7.36E-14	NMB	-0.640809398	1.89E-23
RBM28	1.019240851	2.85E-73	CFAP410	-0.641224308	3.02E-15
ADORA2B	1.018776988	4.86E-17	LRIG1	-0.64163368	1.26E-13
ID1	1.017982857	1.46E-131	EML2	-0.641681444	8.02E-17
FAM155B	1.017330394	9.51E-27	DNAL4	-0.64215129	1.33E-08
RPL23AP7	1.016800391	6.24E-05	H4C11	-0.642628753	0.033227256
CAMKMT	1.016665057	6.43E-10	FAM174B	-0.642709089	1.57E-51
DEPTOR	1.016649917	4.18E-10	NCSTN	-0.643022155	9.47E-22
CHAC2	1.012037942	1.36E-25	UBL3	-0.64319536	2.57E-57
CDV3	1.009560007	6.33E-143	SERPINB1	-0.643259695	1.01E-12
PNPT1	1.009204259	5.71E-87	AC244090.1	-0.643364653	0.00290113
THAP11	1.008917116	6.82E-82	CARF	-0.644418061	0.000763131
CRACR2A	1.008016651	3.30E-21	LIPH	-0.644450406	0.00782212
MARS2	1.00786106	3.79E-45	GABARAP	-0.645177046	3.75E-34
PPTC7	1.007782179	4.01E-53	WDR13	-0.645214703	3.52E-25
NUFIP1	1.007418798	1.74E-37	CXXC5	-0.645229831	1.16E-55
AC009831.1	1.007234455	0.001111071	CRELD1	-0.645402316	2.18E-07
CNKSR3	1.006466246	4.38E-09	KLF12	-0.645837593	0.000919505
ZNF30	1.006169114	8.09E-11	LNX1	-0.646279953	2.09E-05
PDSS1	1.005884226	1.03E-35	TMEM79	-0.646341726	4.05E-12
THAP4	1.003949831	5.40E-101	ITPR2	-0.6464927	2.77E-12
KLHL18	1.003390979	2.56E-49	IGSF3	-0.646500733	3.90E-55
CCDC59	1.002945433	4.16E-57	GOLT1A	-0.646574708	3.58E-05
BRIX1	1.001511397	1.24E-55	SLC46A3	-0.646722429	1.66E-06
NOTCH1	1.000973153	2.61E-46	NUDT16	-0.647373581	4.98E-21
NT5C3A	0.999658214	1.05E-63	ANKRA2	-0.647386934	1.87E-06
SLC16A1-AS1	0.99907774	0.000240484	BAD	-0.647416203	3.30E-20
GTF2H2	0.998957591	3.79E-65	C19orf57	-0.647728377	0.000708889
MRPL1	0.998707678	4.39E-50	SLC25A5-AS1	-0.648397437	0.035860955
MNX1-AS1	0.9982327	1.68E-12	PROB1	-0.648868557	0.003490105
AC107871.1	0.998058676	1.95E-09	RMND5B	-0.649089688	9.42E-63
TCOF1	0.997198778	4.03E-129	PLIN3	-0.649190477	1.43E-27
SOX12	0.99640963	1.50E-77	FICD	-0.649290338	1.06E-06
DNAAF2	0.996303968	1.53E-58	ATF7-NPFF	-0.649403903	0.026892191
LYSMD2	0.995865184	2.02E-41	CHST14	-0.649434696	2.46E-11
INTS13	0.995259854	3.91E-63	PPP1R3E	-0.649608599	7.46E-05
ACTR5	0.995200183	1.36E-17	CNPY4	-0.64969212	2.26E-10
RIOX2	0.991930998	6.26E-87	ODF2L	-0.650140824	0.000687331
POLR1B	0.990824199	4.50E-104	MAN2B2	-0.650333834	4.92E-13
TRPM6	0.990320651	0.000120433	PMS2P4	-0.650377081	0.039130248
LYRM4	0.989896365	1.40E-23	TBC1D8B	-0.650527925	1.33E-08
FASTKD3	0.988959644	2.26E-15	P3H1	-0.650823169	4.10E-09
DLEU1	0.988585406	8.51E-20	TSC22D3	-0.650833135	2.06E-39

PFDN2	0.987362626	2.98E-70	SLC22A4	-0.650847608	0.048323392
SMG1P2	0.986994829	8.84E-17	PLEKHM1P1	-0.650958664	1.87E-11
MRPS2	0.986602112	3.86E-112	BDH2	-0.650994409	0.035946472
PAK1IP1	0.986443321	1.59E-50	XYLT1	-0.65104501	0.032229336
ZC3H8	0.986154375	9.01E-31	LINC00886	-0.651064653	0.000164232
C1QBP	0.984938501	1.12E-157	GM2A	-0.651079095	1.52E-08
SLC35F2	0.98421611	7.85E-52	RHOBTB1	-0.651157708	4.28E-05
C5orf30	0.982795015	2.28E-36	DPYSL2	-0.651206405	1.57E-14
NOL10	0.981250048	6.03E-77	SYNC	-0.651253448	0.002218689
CHORDC1	0.976130833	1.72E-63	FAM47E	-0.651330735	0.045961377
GTF2F2	0.975713602	2.96E-56	QSOX1	-0.651743376	7.59E-72
GNPDA1	0.974267928	1.20E-49	TCAF1P1	-0.651965099	6.86E-08
TYW3	0.973899914	6.94E-35	NPEPL1	-0.652627879	1.26E-41
RUVBL1	0.973707284	1.29E-99	CTNNBIP1	-0.652773488	5.55E-14
ADAM11	0.971486959	5.05E-06	CERCAM	-0.653598939	7.23E-36
DHX33	0.971110572	9.37E-58	RNASEL	-0.654180732	2.12E-06
MRTO4	0.970951044	5.89E-85	STOML1	-0.654251987	7.39E-06
CTU1	0.970390504	1.04E-14	ICA1L	-0.654710478	0.001858005
PHGDH	0.969803559	2.20E-120	TBC1D9	-0.654943026	3.51E-27
NFKBIB	0.969397039	4.63E-47	FLVCR2	-0.655214836	2.38E-25
NIP7	0.968644191	1.11E-126	SMTN	-0.655377784	2.39E-27
SNHG4	0.968450199	3.65E-23	OPTN	-0.655784816	1.01E-28
GLRX3	0.967764181	6.46E-96	S1PR3	-0.655844469	7.60E-19
ZNF239	0.96753087	1.53E-16	CIC	-0.656707677	5.84E-34
KBTBD6	0.967246659	1.64E-28	ARPC4-TTLL3	-0.656995412	0.010595614
STOM	0.966409756	4.25E-82	TRPV1	-0.65728681	0.00031463
TMEM33	0.965488876	2.90E-65	ALG10B	-0.657452993	1.97E-08
AL118516.1	0.964350503	1.87E-07	TMEM187	-0.658150751	5.05E-05
E2F5	0.963666845	1.89E-37	CORO2A	-0.658507622	4.13E-08
WDR4	0.962573756	1.08E-29	N4BP3	-0.658916084	7.48E-40
CYCS	0.96127361	1.69E-104	FAT1	-0.659016543	3.75E-38
EIF3J	0.961225766	8.66E-96	AC010184.1	-0.659023958	0.013615844
CIART	0.960121347	8.40E-20	CTSK	-0.659524272	0.031484192
TIMM17A	0.959911796	6.10E-78	PDLIM2	-0.659744188	9.38E-12
HSPD1	0.958731196	1.31E-157	BMP8B	-0.660127159	8.74E-05
DUSP7	0.957707376	7.50E-18	PRLR	-0.660195575	3.69E-06
ECSIT	0.956522196	1.85E-65	RHPN1	-0.660773102	9.33E-38
PACC1	0.954978453	6.66E-14	RNF207	-0.66088262	5.13E-07
PPID	0.954276562	1.80E-73	HERC6	-0.662280577	7.73E-06
RAB29	0.9530415	1.17E-44	RHOBTB2	-0.663205638	2.79E-27
THUMPD2	0.952860026	2.73E-17	CLTB	-0.663267388	1.92E-92
PPM1M	0.951336015	4.69E-06	CCDC157	-0.664577464	0.000860955
PRMT5	0.950319554	9.31E-119	PXMP4	-0.664633853	1.29E-12
DGKE	0.949550115	1.76E-40	HS6ST3	-0.664643243	2.21E-08
NAF1	0.94908458	4.55E-36	MSH5	-0.665030788	0.0025942
SLC16A10	0.948015844	0.000480013	MFAP2	-0.665071268	0.000931786
CDC123	0.94728095	2.54E-104	AK9	-0.665209152	3.25E-06

ICOSLG	0.947050182	1.05E-09	SLC38A10	-0.665639416	2.68E-44
GATAD2A	0.945852363	2.18E-120	FBXL2	-0.665817317	0.022507235
LTV1	0.945450652	2.95E-71	PLSCR3	-0.666133736	2.26E-20
SHISA9	0.94495779	1.06E-13	NUAK1	-0.666182461	1.27E-07
FP565260.3	0.944652178	8.11E-10	HEG1	-0.666662286	2.39E-05
COA6	0.943682894	3.00E-19	INPP5J	-0.666706241	0.015096359
CDC25A	0.942874415	3.64E-30	TEP1	-0.666847577	3.59E-10
PRMT3	0.942661896	1.06E-49	RBM43	-0.666865775	0.000171646
GNB1L	0.941967002	3.79E-26	CIRBP	-0.667007843	3.46E-34
WDR74	0.941497461	3.86E-86	H2BC18	-0.667012088	0.012194788
IMP4	0.941131774	3.18E-137	TRIM21	-0.667493465	3.07E-09
CCDC138	0.939815606	1.84E-16	RHOC	-0.667963229	1.27E-65
PDP2	0.937597935	2.63E-41	ZNF217	-0.669081159	1.48E-72
PPT2	0.936023387	6.84E-20	TNFRSF12A	-0.669136126	1.16E-11
MRPL50	0.934932141	5.77E-47	QDPR	-0.669259849	1.83E-08
PER1	0.934196071	5.96E-15	ZBTB38	-0.669367185	3.80E-09
TRNP1	0.933810375	4.82E-08	GRAMD1A	-0.669383029	2.27E-11
TNFRSF10A	0.932123392	1.28E-29	GIHCG	-0.669788962	0.007450422
SIK1B	0.932052191	5.89E-33	ALCAM	-0.669972033	1.20E-47
SLC25A15	0.930707522	3.45E-67	AL365181.3	-0.671189656	7.58E-07
SEH1L	0.930583554	4.34E-71	IFT140	-0.671616293	7.74E-21
LRIG3	0.930568828	6.20E-12	ZBED6	-0.671716443	2.29E-05
RNF138	0.929557367	1.30E-52	HR	-0.671909055	8.19E-13
ASNS	0.92927061	7.44E-106	GRIN2D	-0.672313783	0.005306656
GTF2H2B	0.929047751	4.59E-08	ENPP5	-0.672322111	0.000265041
PCOTH	0.927694622	0.013241972	ADCY6	-0.67243079	1.17E-36
GCFC2	0.927691503	4.00E-24	FRAT1	-0.672784472	2.39E-05
MET	0.927065114	5.60E-18	TGFBR2	-0.67282182	9.49E-05
TRMT61A	0.926959938	2.20E-48	FAM111A-DT	-0.672918446	0.002442749
PUS7	0.925990752	1.67E-88	BBS1	-0.673045692	1.06E-06
KRT8P12	0.925700343	0.00090267	PXYLP1	-0.673072832	5.34E-05
NREP	0.925177682	0.01429461	IQGAP3	-0.673272346	6.95E-28
PUM3	0.924745386	2.86E-70	GAMT	-0.673344424	7.95E-05
SLC25A37	0.924397425	9.64E-50	PLEKHA4	-0.673377947	0.032800521
TDRD1	0.924294994	1.88E-33	LRRC37A3	-0.673991424	2.08E-05
SMAD9	0.923698487	0.006996801	TMEM44	-0.674000454	2.88E-08
MARC1	0.923496331	2.81E-23	MARCHF9	-0.674859446	3.51E-12
AK6	0.923481654	3.08E-66	SLC26A11	-0.674873061	8.44E-10
GRPEL2	0.922656759	1.07E-30	TMEM107	-0.674945441	2.06E-05
SETDB2	0.921479509	3.60E-12	CSGALNACT1	-0.675502099	8.35E-06
DUSP9	0.921379799	0.002823524	PIK3R3	-0.675584207	3.46E-31
ZBTB2	0.921046586	4.68E-66	MAP3K6	-0.67576463	5.12E-12
C15orf39	0.919702321	3.57E-54	KANK2	-0.675798407	2.16E-30
RPF2	0.919664006	1.09E-60	AC015813.6	-0.676218362	1.13E-08
HSPA4L	0.918602406	2.38E-38	MR1	-0.676281778	7.10E-08
UCHL5	0.918353627	3.35E-85	STAT2	-0.6763569	3.17E-19
PRELID3A	0.918160671	6.17E-10	MAP4K2	-0.676710389	3.97E-10

PWP1	0.917794997	4.10E-91	ALDH16A1	-0.676810891	2.22E-26
USP18	0.91742954	2.88E-16	MOV10	-0.677282047	9.09E-33
AC083899.1	0.915912238	0.000587223	MMP25-AS1	-0.677401016	7.48E-09
PHB	0.915185686	9.37E-160	SCARF2	-0.677704389	0.031478584
EEF1E1	0.915106968	9.11E-33	CCDC189	-0.677847801	6.03E-05
ADM5	0.914380554	1.12E-06	SEMA6B	-0.67795652	2.77E-05
NPM1	0.913900944	5.43E-141	TAX1BP3	-0.678111748	3.81E-53
MAPRE2	0.913650143	2.40E-06	SLC22A23	-0.678279398	1.03E-13
EIF1AX	0.911052345	8.89E-92	AL109918.1	-0.678458343	0.001179876
NFIX	0.91022717	4.09E-22	LAMB1	-0.678757022	1.44E-48
GNL3LP1	0.910132548	8.01E-07	NPNT	-0.679043713	2.20E-39
POLR3K	0.909827097	1.05E-67	ACAD11	-0.679642739	3.66E-12
FAM136A	0.908305636	5.76E-122	LINC00265	-0.679751674	0.000691294
AHSA1	0.908247718	1.87E-97	TPBG	-0.680592608	1.93E-54
WNK2	0.907535959	9.27E-24	S100A16	-0.68076479	2.10E-77
SLC9B2	0.90557871	2.08E-14	TAPBP	-0.680892752	1.40E-28
ATP11C	0.905392015	3.96E-57	CCDC68	-0.681379215	0.000171432
AGPAT5	0.904793071	2.30E-67	TTC9	-0.681819471	4.91E-26
JMJD6	0.904386094	6.95E-37	HSF4	-0.681974833	1.35E-08
RSL24D1	0.903931105	3.15E-106	RNF223	-0.681975149	2.10E-17
AL161772.1	0.903642626	8.90E-13	AC055811.4	-0.682438382	0.004195018
SCO2	0.902752034	4.66E-21	DENND1C	-0.68262544	4.06E-05
PPAN	0.902291137	5.04E-14	RAB13	-0.683621464	4.78E-40
RN7SL3	0.901913229	0.032256434	KRTAP5-AS1	-0.683953817	6.04E-10
FLVCR1	0.900831988	3.44E-14	MALRD1	-0.684416284	0.017262773
NUP35	0.900043602	2.55E-36	RBM11	-0.68457035	0.0378601
OAF	0.896305233	1.94E-13	KIAA0895L	-0.684796105	2.18E-11
ZBTB24	0.894679243	3.21E-38	ZNF226	-0.68501928	0.000161348
KBTBD8	0.894302589	7.84E-12	RNFT1	-0.68567815	5.62E-13
TENT4B	0.89389658	3.37E-36	TIGD7	-0.68574751	0.009592552
ZPR1	0.892759727	2.34E-43	RGS9	-0.686093305	0.02010857
MRPS6	0.8903131	3.43E-48	NINL	-0.686340914	7.34E-15
RP9	0.890239681	3.14E-26	HSPA2	-0.686437697	3.47E-09
RPARP-AS1	0.890016836	7.02E-14	GNAI2	-0.686496029	1.15E-47
DUS3L	0.888397545	5.20E-40	FOXO4	-0.686568772	0.000839887
OXNAD1	0.888137381	3.57E-29	CDKN2C	-0.687023501	3.94E-12
NCS1	0.885866004	3.26E-83	AHNAK	-0.687654386	3.81E-19
C12orf29	0.8857381	1.20E-30	ARHGEF25	-0.688125713	2.43E-05
BOP1	0.885716461	1.20E-128	CHKB-CPT1B	-0.688954455	1.57E-05
CEBPZ	0.885654932	2.92E-74	PHLDB1	-0.689288413	5.56E-12
TRMT1	0.885603318	5.14E-71	PIM1	-0.689660551	1.71E-05
NCL	0.885234256	4.00E-147	CSKMT	-0.689724358	9.08E-08
LARP4	0.884239495	1.17E-130	RWDD2A	-0.691292768	6.32E-09
MTHFD1L	0.884000218	8.25E-86	AL031123.2	-0.691472923	0.027152624
BCL11B	0.882886441	1.24E-12	PMEPA1	-0.691520671	2.60E-83
NOB1	0.882838987	1.23E-98	TMCO3	-0.691820664	1.02E-22
PTDSS1	0.882444964	5.39E-112	IER3	-0.691843124	3.85E-33

CDK8	0.880869975	1.33E-43	TBC1D2	-0.692717291	6.71E-11
CD320	0.880805346	1.85E-40	SDHAP3	-0.693186162	0.006519705
XYLB	0.880290645	6.17E-09	SLC25A45	-0.693229157	9.32E-05
SIX1	0.880002897	1.86E-19	VAMP1	-0.693258442	0.000739763
VPS9D1-AS1	0.87920076	8.06E-48	PLEKHA2	-0.693383713	4.36E-08
DANCR	0.879089704	3.56E-60	CYFIP2	-0.693831174	1.43E-15
DGAT2	0.876900686	3.15E-08	IRS2	-0.693911628	3.19E-09
NUDT19	0.876465711	2.84E-37	CD27-AS1	-0.694005829	0.000515505
Z83844.3	0.87627266	0.000376955	PARD6A	-0.69427476	1.03E-05
FAM162A	0.875506024	5.35E-43	PCAT7	-0.694407466	6.23E-07
C12orf45	0.875375509	1.96E-17	C10orf143	-0.694757729	0.001118761
CFAP97	0.874908094	4.75E-67	FIG4	-0.694973117	2.25E-15
CACYBP	0.874172456	9.71E-66	SPSB4	-0.695171853	0.032807974
SAPCD2	0.873387875	1.52E-95	CCDC71L	-0.695403592	6.22E-10
TMC8	0.872904865	0.003596138	KLHL24	-0.695534118	4.26E-11
PSPH	0.87185691	1.58E-26	ZNF362	-0.696152522	1.10E-12
MRPL36	0.871003472	2.09E-37	ATF6B	-0.69616818	9.92E-29
TRIAP1	0.868740837	9.07E-36	STX5	-0.69630759	9.73E-25
TAMM41	0.867325783	9.08E-26	TMEM132A	-0.696391719	9.94E-54
DGUOK-AS1	0.866492343	0.000501153	ARTN	-0.696987161	0.007100428
SLC25A22	0.866354971	8.59E-64	MYL5	-0.697029926	0.001308655
ALKBH2	0.865666921	9.42E-39	CADM1	-0.697099495	7.84E-21
YBX3	0.863719642	4.18E-100	H1-2	-0.697122587	3.75E-05
FIRRE	0.863275005	4.54E-08	TMEM129	-0.697155133	4.70E-13
AC125257.1	0.86278449	0.000193279	FARP1	-0.699149011	2.63E-32
TMC5	0.862203049	7.17E-05	SLC25A24	-0.699575739	2.30E-73
ADGRA3	0.861815822	2.89E-45	KHNYN	-0.699730215	2.63E-28
MRPS12	0.861643116	1.87E-52	PGAP6	-0.700055346	2.05E-44
RPRD1A	0.861474184	6.41E-84	RTKN2	-0.700807794	8.64E-10
APTR	0.860843479	2.42E-15	F8	-0.700903188	0.002743843
MTIF2	0.860251942	1.09E-60	CAPN1	-0.700925232	5.60E-84
COX10	0.86021684	1.78E-17	AGAP2	-0.701309976	5.16E-14
SPRY1	0.860208011	1.22E-07	TMSB15B_1	-0.701336081	0.041048487
CDR2L	0.859936934	1.82E-45	GLIPR2	-0.701374077	0.000991152
EXOSC4	0.858094544	2.33E-57	CPE	-0.701458099	1.88E-26
PPAT	0.857569714	8.53E-68	IRF2BPL	-0.701491013	1.82E-61
LRRC58	0.857247982	1.87E-97	TINF2	-0.701773331	7.04E-16
EXOSC7	0.856206227	3.05E-28	OTUB2	-0.701801405	0.009037828
TAF5	0.855465288	8.31E-14	ZBTB46	-0.701931093	2.80E-07
PES1	0.855146944	3.41E-98	N4BP2L1	-0.70308753	0.016332733
NOP56	0.853468898	2.77E-102	WWC3	-0.703257028	1.81E-60
HELB	0.853357337	2.86E-08	PTOV1-AS2	-0.703415717	0.024398384
ABCE1	0.852914073	6.59E-119	CDC20B	-0.703748609	0.000544047
ZHX1-C8orf76	0.852345479	1.10E-05	PDK2	-0.704106972	1.51E-12
MEST	0.851346002	5.64E-96	OPN3	-0.704367179	1.08E-10
DHX37	0.85034933	1.05E-78	MIR34AHG	-0.704486132	0.000525472
INTS10	0.849662127	1.38E-44	IFT27	-0.705048674	1.21E-10

EPB41L4B	0.849371616	3.67E-57	ZDHHC2	-0.705239375	0.004830848
PM20D2	0.848773218	1.05E-35	AL365181.2	-0.705421145	0.001116807
DYRK3	0.848738267	1.13E-11	SMPD1	-0.705449422	5.61E-26
AL161665.1	0.848706325	0.047002971	GOLGB1	-0.705585328	3.73E-55
SLC27A4	0.848529961	1.02E-59	AC112220.2	-0.705683645	2.11E-05
MPP3	0.848395582	2.36E-07	CNKSR1	-0.705780016	1.45E-08
TUBGCP4	0.848289988	1.84E-40	PGAP3	-0.705832575	3.08E-07
ADCY3	0.846510779	4.14E-75	SLC22A15	-0.705966098	4.94E-06
TFAP4	0.845300186	1.74E-28	PXDN	-0.708302842	7.69E-45
KDM4A-AS1	0.844960335	0.010132057	ENPP1	-0.708453987	3.48E-21
DFFB	0.844640441	7.30E-08	MALAT1	-0.708803129	6.06E-43
SNHG10	0.844579855	5.25E-18	PHYH	-0.70959715	3.03E-08
NUDCD1	0.844333154	5.78E-94	ACADS	-0.709735735	1.60E-05
RRP15	0.842820424	3.48E-29	SPACA9	-0.709795485	0.002125438
MRPL3	0.84213863	7.85E-105	GOLGA8N	-0.709862703	0.019892359
SCLY	0.84148306	8.73E-26	TMEM198B	-0.709995467	1.59E-06
RSAD1	0.840803015	1.45E-61	IQCD	-0.710544551	0.006660541
MYBBP1A	0.840177503	4.26E-102	WDR91	-0.711117287	6.23E-10
GRK5	0.839417059	3.68E-07	PPP3CB-AS1	-0.711127014	0.006279793
TYRO3	0.83884823	1.06E-25	TSTD1	-0.71198977	2.65E-14
NDC1	0.838623461	1.92E-66	MCOLN3	-0.712028395	0.002734577
TOMM5	0.838565337	1.05E-54	DENND6B	-0.712626464	5.15E-06
ICAM5	0.838453909	0.000375137	PLCD3	-0.71305323	1.58E-56
FKBP11	0.838016004	2.56E-22	FZD7	-0.713188417	0.000361703
C12orf73	0.837957455	1.62E-12	DTX3	-0.71349103	6.62E-10
NOP14	0.837556148	2.95E-77	IGFLR1	-0.713589614	0.02017191
PINX1_2	0.837182802	2.32E-19	JUN	-0.713600597	9.46E-24
ZNF511	0.836441791	3.98E-28	REEP6	-0.714414515	1.55E-08
KTN1-AS1	0.835237409	1.65E-07	TIMP1	-0.714469789	4.99E-28
SOD2_1	0.835028175	3.60E-55	CPEB2	-0.714594544	6.90E-05
IL17D	0.833789825	6.04E-18	CUL7	-0.714668134	8.75E-32
DNAJA3	0.833565619	3.18E-106	LINC00888	-0.714680272	9.54E-05
CISD1	0.833498873	2.69E-23	IL11RA	-0.715210868	0.046890786
MTRR	0.83185427	1.82E-22	GLB1L	-0.715329947	0.00122599
TIMM21	0.831572529	2.89E-29	TMEM35B	-0.715347162	6.07E-05
NXPH4	0.831166571	9.07E-12	SDC3	-0.715555899	9.49E-15
LINC01311	0.83038185	0.00252794	SLC2A11	-0.716716501	6.63E-10
SRPK1	0.830168487	4.71E-64	SLC37A1	-0.716808023	2.91E-40
CUTC	0.82946661	3.09E-19	GPR153	-0.717052554	2.12E-06
ENTR1	0.829131126	9.87E-74	IKBKGP1	-0.717358057	0.00294455
NARS2	0.828110016	5.77E-33	PINK1	-0.717599674	1.87E-26
NLE1	0.827736858	9.31E-28	PEG10	-0.71773191	4.73E-09
AC146944.3	0.827521565	0.002080075	BTN2A2	-0.718098527	0.000414468
GPT2	0.827438606	2.05E-59	TRPV4	-0.718130669	7.38E-06
TANGO6	0.825130693	3.14E-18	CTAGE4	-0.71820279	0.000832351
FAM156B	0.825107241	2.55E-05	RHOU	-0.718513945	2.66E-14
CTSC	0.824678607	2.07E-23	SLC44A3	-0.718514332	0.000824129

NFIA	0.82340629	6.24E-17	TMEM63A	-0.718787887	1.65E-28
FAM210A	0.823358658	1.13E-35	KAT2B	-0.71941412	1.03E-20
PPM1J	0.823142843	0.001427762	MIDN	-0.720276272	1.08E-64
MRPS23	0.822990001	2.27E-66	CYLD	-0.720780105	1.69E-14
REXO2	0.822609578	3.65E-26	TCEA2	-0.720816907	9.26E-15
IKZF5	0.822139546	2.47E-30	AP002761.4	-0.720963588	1.93E-16
SEPTIN6	0.821958083	0.002328581	RNFT2	-0.721173917	1.13E-05
ARL6	0.821809676	6.88E-09	HOTAIR	-0.721706887	0.025393617
FAM189B	0.821412988	5.46E-36	ANXA2	-0.721826096	1.40E-95
IPO5	0.821131009	4.15E-95	ZNF75D	-0.722225329	3.83E-16
THAP2	0.820869504	0.000297063	USP46-AS1	-0.722338981	0.003201709
XPO5	0.819695482	1.59E-104	NBPF4	-0.723496246	0.000134368
ADSL	0.819622052	2.08E-72	SOX4	-0.723584629	6.26E-41
ENTPD1-AS1	0.818012972	1.18E-23	NPHP3	-0.723757049	5.88E-11
BOD1	0.817615448	4.70E-66	C1orf115	-0.724283386	4.32E-07
PDCD11	0.815812045	4.67E-81	CNFN	-0.724289304	0.037039852
PEX5	0.814970551	1.59E-46	FREM2	-0.724418289	1.10E-20
RFESD	0.814052566	0.005264993	S100A6	-0.724523969	2.54E-33
AHCTF1	0.813333163	2.23E-71	RAB5B	-0.724644886	1.94E-55
PRPS1	0.812731651	3.55E-67	S100A9	-0.725092698	0.002390025
HEATR1	0.812377184	1.43E-69	RTL8C	-0.725097923	8.46E-40
COA7	0.812307855	3.71E-45	SRRM2-AS1	-0.725549238	0.005971486
ZNRF2P1	0.811927555	0.000335976	ZNF750	-0.725669167	0.011314412
QRSL1	0.81153395	5.58E-25	PPDPF	-0.726270477	7.70E-59
UTP15	0.811250025	7.68E-45	SNX21	-0.726580313	4.45E-17
MBLAC2	0.809842693	3.85E-15	GLRX	-0.727150866	0.011549464
FBRSL1	0.80934377	2.06E-88	ATG16L2	-0.727172952	2.00E-11
RPL23AP82	0.808921385	1.02E-13	GRHL3	-0.727291229	6.62E-40
DLAT	0.808882948	4.30E-53	MYL6	-0.727696337	6.59E-110
SKP2	0.80776896	1.93E-13	HECW2	-0.728117731	0.000156862
CCDC112	0.807227634	1.23E-12	TUFT1	-0.728356554	5.75E-35
DIAPH2	0.806725718	1.31E-23	LINC01001	-0.728556253	0.005942497
AKAP1	0.806518284	1.14E-85	JMJD1C-AS1	-0.728587729	0.033319116
TRIP13	0.806268964	2.15E-30	ZDHHC12	-0.728808183	3.92E-29
TIPIN	0.805982847	2.57E-21	NUCB2	-0.728925819	3.23E-32
EXOSC5	0.805443484	8.84E-46	ANO9	-0.729046849	6.02E-10
TOMM40	0.804191779	4.54E-94	GPRASP1	-0.729441883	0.040347527
UBIAD1	0.803708724	8.12E-22	AC026748.3	-0.729532293	0.000242956
DHODH	0.803168373	2.19E-26	MGLL	-0.729795489	2.22E-10
SHLD3	0.80245723	0.000150685	IGF1R	-0.729806248	2.00E-50
NGDN	0.802281016	1.43E-28	PIEZO1	-0.730191083	1.24E-87
STK26	0.802218707	1.93E-60	MTHFR	-0.730241968	5.46E-10
DBF4P1	0.801724399	0.006083011	OLFM1	-0.730474111	4.38E-29
NOL8	0.801562705	1.94E-52	AC093512.2	-0.730846496	7.32E-07
MAD2L1	0.801511635	1.50E-56	MEAK7	-0.731161884	2.60E-17
AC018645.3	0.800834265	0.021347566	GALNT12	-0.731314502	1.00E-07
GTPBP4	0.800260584	1.02E-81	DISP1	-0.731650689	0.008192574

RBM19	0.800193417	1.01E-39	MXD3	-0.731765618	3.43E-17
RMRP_2	0.799509929	0.002192081	MVD	-0.732071127	2.67E-32
PA2G4	0.798146814	8.51E-125	GRN	-0.732117944	1.79E-65
SLC45A3	0.798072747	4.33E-09	LINC01138	-0.732148356	0.014074348
TBRG4	0.797835172	2.06E-72	AAMDC	-0.732376959	0.000147687
NKD2	0.797199244	2.00E-05	CPT1B	-0.733241123	1.18E-08
RRP12	0.79713335	8.80E-70	RAPGEF2	-0.733250803	9.19E-44
METTL5	0.797069124	7.26E-30	PDGFA	-0.733683943	2.52E-13
GFM1	0.79682094	1.39E-75	ZNF311	-0.734038964	0.021889704
CTPS1	0.796796101	1.14E-77	FKBP2	-0.735110251	2.66E-20
ENAH	0.796503531	2.51E-48	MINDY1	-0.73540275	9.37E-09
HSPA14_2	0.79576732	4.26E-32	RAB8B	-0.735415326	2.86E-10
PGAM5	0.795296851	6.94E-82	AGO4	-0.735558599	2.26E-07
NAA15	0.795090834	3.82E-85	CCDC102A	-0.73568212	3.04E-05
FKBP4	0.794407801	6.28E-120	HEYL	-0.735943061	0.003953179
MRPS27	0.794272529	7.00E-79	GRAMD2B	-0.736104582	2.13E-12
MEMO1	0.794083668	7.37E-40	SLITRK6	-0.73688922	0.000265014
NAA50	0.793937388	1.08E-147	ALPK1	-0.736905635	1.56E-05
TMEM70	0.793579979	3.64E-33	ROGDI	-0.73703567	3.44E-22
MRPS5	0.792867734	2.79E-53	LINC01547	-0.737068588	1.19E-09
SUV39H2	0.792860044	7.63E-43	CLTCL1	-0.737334481	0.000119214
EIF5	0.792175132	1.13E-143	AL031777.2	-0.737502337	0.041700073
UTP4	0.792135752	2.54E-94	DUSP1	-0.737911375	4.18E-17
GALK2	0.791684347	3.66E-19	SAMD15	-0.738013294	7.87E-06
AL139353.1	0.79109856	0.027046958	PCED1A	-0.738448185	4.42E-13
POP7	0.790735516	1.25E-54	BACE1	-0.738500055	3.30E-46
UAP1	0.789762562	5.82E-42	LFNG	-0.739845993	1.44E-15
C15orf61	0.789677119	6.42E-10	H2BC8	-0.740937584	0.000188964
ZNF770	0.788183715	3.35E-42	PLXND1	-0.740974291	6.13E-34
DKC1	0.788112321	9.66E-72	SRCIN1	-0.741173798	1.87E-09
DTX4	0.788103268	1.03E-10	TTLL1	-0.741245266	4.07E-06
RCL1	0.787212103	5.40E-29	CCDC162P	-0.741505867	0.001387519
PDXP	0.786922531	3.06E-31	P2RX4	-0.742235331	4.85E-21
C20orf27	0.7855568	4.04E-71	NEK11	-0.7423966	2.10E-05
L3HYPDH	0.784872554	3.59E-21	ANO6	-0.742788903	1.04E-46
ALDH1B1	0.784743814	7.31E-36	PROM2	-0.742918376	1.31E-56
ARMC6	0.783594165	7.70E-59	GTF2IP13	-0.743645573	0.004470696
KARS1	0.783385332	4.05E-112	LMTK3	-0.74371304	2.57E-12
FAH	0.783187129	3.13E-16	FGFR2	-0.74387893	3.50E-24
ACAT1	0.782976527	2.05E-35	S100A13	-0.744005518	6.93E-50
LINC-PINT	0.782723375	0.01988894	LMF2	-0.744056308	2.51E-31
AP001505.1	0.781935716	0.002736259	PCDHGB5	-0.744511912	1.02E-11
HSPA9	0.78192604	5.76E-108	C5orf38	-0.744667288	1.25E-11
ELOA	0.781420581	3.92E-53	S100A14	-0.744974854	1.42E-52
STC2	0.781361514	1.83E-54	ACTR1B	-0.745271689	3.26E-35
LYRM7	0.781230488	3.01E-15	DGKQ	-0.74535583	1.90E-12
SLIRP	0.78052347	5.79E-38	TRIM34	-0.746182851	0.000786635

SLC19A1	0.780516485	6.90E-51	CALCOCO1	-0.746274882	1.69E-19
WDR75	0.78049632	8.23E-59	PRSS27	-0.746677111	0.029844612
HSPE1-MOB4	0.780386961	0.045872843	TMEM219	-0.747697505	1.07E-13
TTLL12	0.779950299	3.35E-104	MRAS	-0.748368589	0.007828533
YARS1	0.779346801	5.68E-84	H4C15	-0.748521466	0.004026235
MRPL15	0.779186988	1.39E-49	SYTL1	-0.748547983	2.97E-09
RHEBL1	0.778940042	0.02067077	ARSJ	-0.749451122	1.47E-05
MRPS17	0.77851611	2.87E-18	ZIC4	-0.749515877	0.000712273
PRMT1	0.778246968	3.71E-102	ST3GAL3	-0.749834033	0.003069923
RRN3	0.778118338	1.53E-71	AL499602.1	-0.75022565	5.95E-05
PAM16	0.777452562	2.91E-25	AR	-0.750467301	0.000271693
TRMT10C	0.776519197	1.64E-36	PLEK2	-0.750890093	3.02E-05
PPP2R1B	0.776272772	9.33E-28	MAN1C1	-0.7510807	0.017914986
NSUN2	0.776120382	1.52E-67	SEC14L2	-0.751542344	1.11E-06
PUS1	0.775994839	3.60E-41	AC093673.1	-0.751688042	0.016658064
TTYH2	0.775939731	0.015752118	ARRDC3	-0.751758806	1.29E-05
MCAT	0.775595663	2.37E-25	ARHGAP42	-0.752506533	0.000818982
AMD1	0.77521606	1.23E-94	SPTSSB	-0.752701817	6.78E-145
ISCA1	0.775149407	4.73E-54	MBNL2	-0.753504545	4.73E-21
RRAS2	0.774965755	1.33E-38	PCDHGB1	-0.754101847	0.000258645
TATDN2	0.773778277	3.12E-71	TES	-0.754526467	1.90E-29
CALML4	0.773504325	0.003049647	VPS37D	-0.754578649	0.003198248
NOCT	0.772774119	2.09E-13	NCAM2	-0.75521818	9.23E-27
FKBP14	0.771746504	1.58E-19	UTRN	-0.755288512	1.22E-27
DCTPP1	0.771161369	1.41E-99	SLC24A1	-0.756049311	9.20E-15
SRXN1	0.770429461	1.25E-52	UBE2Q2P1	-0.75646039	0.010993625
CBR1	0.770342019	2.86E-61	PRSS30P	-0.756545417	0.001719258
AC011462.5	0.769887054	0.013398986	FRS3	-0.757118563	0.004707667
GARS1	0.769721749	2.96E-83	CTDSP2	-0.757191274	4.52E-51
GTF2H2C	0.76869193	9.78E-43	SLX1B	-0.757301041	1.46E-29
CLNS1A	0.768659347	1.01E-77	TTC30B	-0.757482868	1.04E-06
F12	0.768528038	6.26E-21	TRIM5	-0.757607159	8.21E-10
URB2	0.768465734	1.07E-30	TRIM16	-0.75779605	2.02E-35
GNL3	0.768237521	2.45E-71	SEPTIN1	-0.758214377	0.049991882
RAI14	0.767808956	4.48E-18	ANGPTL4	-0.75855144	2.39E-11
SPHK1	0.766706893	2.66E-34	SLC31A2	-0.758721128	2.02E-08
ENOPH1	0.766697866	4.41E-64	CFAP44	-0.758750859	9.61E-08
OTUD6B	0.766431191	1.00E-32	SGCB	-0.758928465	2.23E-12
UTP6	0.766307362	6.41E-34	HLA-C	-0.759135414	7.09E-56
NR4A3	0.765141678	9.15E-06	C20orf204	-0.759179764	0.026386712
EIF5A	0.764971568	1.52E-111	CDKN2D	-0.75965292	0.000470477
EMC8	0.764728904	5.61E-45	PLAUR	-0.760368554	6.56E-05
NOP58	0.764019911	1.61E-72	EIF4E3	-0.760545439	1.83E-15
GTPBP10	0.763585267	1.29E-41	MFGE8	-0.761162811	9.37E-08
SHLD2	0.763377551	1.62E-43	ARL4C	-0.761368094	1.63E-09
DDX51	0.761784954	2.19E-33	ADGRB2	-0.761678943	0.006952269
MRPL4	0.761772635	3.00E-64	LARP6	-0.761750037	0.000102729

TSR1	0.761752335	2.05E-67	MARCHF2	-0.761804036	3.09E-08
TTC27	0.761329049	2.67E-28	PLA2G6	-0.761879297	3.21E-05
DFFA	0.760550614	7.74E-52	KLF4	-0.762024294	8.61E-30
LRRC59	0.760523302	1.60E-104	PODNL1	-0.762602729	0.001637309
NHP2	0.757590295	3.88E-100	IL4R	-0.762887397	2.86E-21
TMED5	0.756637296	4.71E-40	PYCARD	-0.762943193	5.35E-21
ZNF598	0.75611909	1.15E-74	CRABP2	-0.762954791	1.00E-86
WDR77	0.755841644	4.85E-60	NPAS2	-0.763050756	1.71E-30
PTPN2	0.755153017	1.73E-37	RIBC2	-0.763064962	0.000157978
GPR135	0.754780236	0.004988853	LRRC23	-0.763971334	0.000910481
SIAH2	0.753927595	2.52E-44	TVP23C	-0.764165977	0.016505543
MND1	0.753631189	7.28E-13	ITGB5	-0.765432375	2.96E-98
ISM1	0.753508242	0.008606522	KLRG2	-0.765660195	0.03167788
METAP2	0.753088405	2.03E-67	AP3B2	-0.765709038	0.019524946
FKBP5	0.752788477	3.70E-46	SPRY4	-0.76669535	5.39E-06
AC012676.1	0.752373938	0.025713984	GRB7	-0.766710888	7.09E-12
NME1	0.752334389	1.65E-74	IFI35	-0.766821825	4.75E-06
KHDRBS3	0.751233575	5.77E-05	PHC1P1	-0.768167671	0.013526643
IPO11	0.75075318	2.67E-42	ZNF497	-0.768534673	4.93E-05
FAM72B	0.750672214	8.49E-05	PRPH	-0.768670883	0.020031783
RDH13	0.74958844	1.81E-22	SHISA2	-0.768702317	0.017637738
NOP2	0.749424207	2.95E-49	BASP1	-0.768872593	7.58E-64
LARP1B	0.749334778	1.93E-25	FAM47E-STBD1	-0.769499383	0.000245184
PARPBP	0.749318151	1.25E-22	SPIRE2	-0.769861545	3.83E-15
XRCC2	0.748429443	4.54E-29	SNED1	-0.770008071	0.003137192
ABCF2_2	0.74824325	4.98E-79	RPS10P7	-0.770477783	0.004084793
ARC	0.74813936	0.00193275	AL022069.3	-0.771291862	0.026534414
NOC2L	0.747870936	3.96E-88	CELSR2	-0.771354315	1.81E-14
METAP1	0.747020378	2.04E-56	CREB3L4	-0.771890385	2.95E-25
PAICS	0.746496786	5.03E-90	HEXD	-0.771995878	4.74E-12
RTN4IP1	0.746335922	2.41E-11	ALDH4A1	-0.772615459	6.29E-25
AC022966.1	0.745564628	4.28E-53	CALHM2	-0.773074094	3.01E-17
SRPX	0.745006938	0.00590037	EFHC1	-0.773549351	5.78E-13
SMG1P1	0.744682831	0.000128559	TMEM234	-0.773595873	0.000173752
DDX31	0.744676848	4.99E-35	SLC4A11	-0.773752647	1.10E-08
NAA25	0.74384152	8.69E-46	GALNT10	-0.773888549	1.62E-70
HDHD5	0.743120851	4.76E-53	GPR158	-0.774177396	0.003909965
NAMPT	0.742975141	1.65E-99	PRKCZ	-0.774526287	9.54E-20
GEMIN4	0.742974287	4.07E-52	LASP1	-0.774687491	4.11E-73
WDR35	0.742860095	2.45E-23	EPPK1	-0.77469278	2.01E-09
HSD11B2	0.741524721	0.004439645	TRPT1	-0.775477827	3.96E-19
TCP1	0.741488293	2.38E-70	C1orf226	-0.775498308	4.88E-15
TOMM34	0.741169902	5.53E-50	AC133644.3	-0.775599111	0.000159235
KDM1A	0.74030394	1.39E-47	CCN1	-0.775765147	5.70E-24
TBC1D14	0.739918084	2.66E-53	EPS8L2	-0.776046499	3.40E-18
RBM38	0.739751435	7.66E-56	ILDR1	-0.776089658	4.91E-05
AC005831.1	0.739613732	0.023718936	ERBB2	-0.77641522	4.27E-77

DTWD1	0.739371548	6.60E-29	TTC30A	-0.776955769	1.48E-05
EXOSC3	0.739233492	2.74E-26	HBP1	-0.777217254	5.72E-45
UTP23	0.739178133	4.70E-28	AC068888.1	-0.777262283	1.17E-41
FZD9	0.738957005	0.007349074	CHMP2A	-0.777507959	4.50E-41
ZNRF3	0.738829281	2.04E-16	SLC25A35	-0.777592308	0.017334449
ZC3H15	0.738117733	2.18E-62	LRRC46	-0.777642445	0.017423903
FAM53B	0.73787786	8.31E-43	MARC2	-0.777656979	0.002644052
SLC18B1	0.737564412	9.28E-20	AC004816.1	-0.778587326	9.93E-08
GART	0.737403879	2.70E-71	GNAO1	-0.778770879	0.023997622
UNC5B	0.737234393	0.000598872	ALDH6A1	-0.778793769	1.84E-25
QSOX2	0.737098528	1.53E-52	DHRS2	-0.778803211	4.89E-08
NUS1	0.736624213	1.03E-77	RORA	-0.778834835	0.000368964
CERS1	0.736518799	1.24E-08	ADAP2	-0.779219953	0.03487717
NAT10	0.736322942	7.39E-73	BBOF1	-0.77934811	1.70E-06
KNSTRN	0.73605372	1.01E-19	PAQR8	-0.77961317	0.008813613
NR1D2	0.735994043	2.67E-37	PDGFB	-0.779858659	5.16E-28
IMP3	0.735827182	1.48E-44	STARD4	-0.780040578	1.10E-07
UTP25	0.735291345	8.44E-23	CLDN4	-0.78010931	1.63E-78
RBM18	0.735052387	2.15E-29	CCDC74A	-0.780355405	0.009514116
ME2	0.735030237	4.95E-22	PHLDB3	-0.780536419	5.88E-09
SAAL1	0.734781474	2.47E-21	SMPDL3A	-0.780768289	3.95E-08
ZNRD2	0.734529967	8.28E-26	MAPK8IP1	-0.780865807	0.00020918
NOL11	0.734434001	8.06E-79	ANKRD9	-0.780945751	1.50E-30
C8orf33	0.733456377	5.57E-65	DMPK	-0.781254474	1.65E-46
ZNF331	0.733121466	2.36E-11	SH3BP5-AS1	-0.781778803	1.39E-06
YARS2	0.733042794	4.95E-18	VMO1	-0.782106751	0.042943986
IL27RA	0.732919804	9.27E-20	PLA2G15	-0.782415371	1.68E-23
SLC5A3	0.732816833	1.00E-20	FHL1	-0.782570087	0.035470363
IFRD2	0.73263097	4.92E-63	ZFP36	-0.78260226	2.40E-14
KDM1B	0.732219631	3.29E-21	GPR173	-0.783140211	0.007154983
AIMP2	0.732115416	7.82E-38	TRIM38	-0.783219844	1.64E-09
NEIL2	0.732114368	1.86E-12	SRRM3	-0.783664727	2.27E-06
SDAD1	0.731167653	5.74E-50	TMEM45A	-0.783707668	0.030467287
DBF4	0.730578377	1.35E-54	AC008556.1	-0.783739975	0.001608018
ARL5A	0.730217135	3.77E-45	PRKACB	-0.784334619	0.000139961
TMEM201	0.730140101	5.21E-30	MAPRE3	-0.784571772	8.53E-10
ETF1	0.729650912	5.56E-92	SWT1	-0.784899686	0.023639927
ABCF2_1	0.729628667	1.95E-50	CRIP2	-0.784917789	4.25E-45
XPO4	0.729628506	1.62E-39	EPHX2	-0.785156605	0.008539769
EBNA1BP2	0.729401297	3.87E-67	VAMP5	-0.785212274	0.000165575
KPNA3	0.729302216	7.94E-48	PDE9A	-0.785224917	1.64E-08
PFDN4	0.728626578	7.78E-66	CCDC120	-0.785611293	8.82E-27
CUL4A	0.728510102	5.98E-53	AC023509.1	-0.785663704	0.000315103
MFHAS1	0.727344892	2.62E-28	OSBPL7	-0.785841556	9.24E-13
SLC25A12	0.727256266	4.96E-11	ADM	-0.787009717	2.92E-11
TSFM	0.72662754	2.69E-36	SPPL2B	-0.787077218	1.70E-16
SMAD1	0.726597819	2.55E-17	ARMCX3	-0.787344657	3.77E-22

RWDD4	0.726294907	1.12E-29	FAM167A	-0.78799408	0.00526904
RFK	0.72623949	2.59E-47	SMIM29	-0.788877241	1.55E-10
XPO6	0.724732559	1.53E-90	MAN2B1	-0.789201475	4.28E-43
POLG2	0.724287038	1.82E-19	TSPAN14	-0.789773606	5.47E-82
FOXO3B	0.723703763	1.12E-06	CORO1B	-0.789883434	1.15E-64
ZDHHC9	0.723485689	7.97E-64	BNIP3L	-0.791533742	3.46E-36
SCO1	0.723320308	1.41E-32	GYG2	-0.791940779	0.032752399
RITA1	0.723024371	1.89E-42	LYSMD4	-0.792051603	5.10E-09
TPST2	0.722333638	6.62E-16	SLC4A8	-0.792194024	6.32E-08
B4GALT3	0.721873735	2.33E-35	TMEM238	-0.792879349	1.81E-08
HSD17B6	0.721832094	0.009044319	ERRFI1	-0.793586773	8.00E-58
NDUFAF5	0.721201265	3.65E-12	COPZ2	-0.793703992	9.57E-11
ANKRD27	0.71963832	4.39E-54	DNASE1L1	-0.793857344	2.42E-25
UBA2	0.719490149	1.22E-82	ZNF703	-0.794204729	5.60E-47
RMND5A	0.71922911	1.89E-32	ACSF2	-0.794861978	2.38E-23
TEAD4	0.71889431	8.40E-20	GALC	-0.795082292	0.007339064
RNF8	0.718773397	1.82E-22	ZSCAN31	-0.79522219	0.010791761
DUS2	0.718647581	3.13E-22	PRSS22	-0.795309845	5.19E-21
RAPGEF5	0.718181341	1.17E-05	ETHE1	-0.796289627	1.24E-06
IFI30	0.718086754	2.92E-42	FAM3C2	-0.796318791	0.000121833
PLIN2	0.717926898	1.37E-05	TSPAN5	-0.796748648	1.78E-06
NTMT1	0.717761574	4.83E-45	XBP1	-0.796786472	4.24E-26
MRPL30	0.717754561	7.60E-40	MAFA	-0.79776809	2.65E-05
DCAF4	0.717701791	3.72E-34	RGL2	-0.79801008	5.13E-64
HSPE1	0.717488324	8.43E-60	AIFM3	-0.798191585	0.000364618
UNG	0.71679982	6.76E-78	RDX	-0.798232709	2.61E-141
CRLS1	0.716183386	5.79E-26	LIMCH1	-0.79830426	9.98E-42
KANK1	0.715317569	1.18E-29	HOMER3	-0.799027951	9.51E-21
CDK5R1	0.715249433	0.000566985	HSPB8	-0.799095768	1.40E-36
PPA1	0.714922615	7.31E-67	AC006128.1	-0.799630969	1.42E-05
ATP6V0E2-AS1	0.714711786	0.001656126	WDR78	-0.799875974	0.025329446
NAGPA	0.713604374	9.70E-16	INHBB	-0.799877107	1.53E-71
TIMM22	0.712841897	3.25E-24	HID1	-0.800238133	6.51E-74
MDN1	0.712439643	1.66E-32	ZFP36L2	-0.800618611	4.76E-125
PRTG	0.711675533	2.12E-06	ZNF385A	-0.800680249	1.21E-36
LRPPRC	0.71133858	6.53E-102	NDUFA6-DT	-0.800743274	0.000352391
EIF2A	0.71068193	1.14E-61	H2AC19	-0.800917338	1.50E-24
ZNF330	0.710525955	8.31E-29	GOLGA6L5P	-0.800957336	6.26E-06
FYN	0.709962698	0.001099296	TFF3	-0.801335704	3.32E-07
SSB	0.709891028	7.31E-76	SYCP2	-0.80260326	9.76E-21
IARS1	0.709842003	2.68E-122	STARD13	-0.802672895	1.22E-05
MRPL45	0.709740744	3.39E-29	FOXJ1	-0.803345496	0.009015209
POP1	0.709508118	1.29E-41	PTGR1	-0.803381549	0.00132788
UBE2S	0.709491655	6.95E-65	SH3TC2	-0.803509076	0.008130328
SMYD5	0.709157502	4.03E-33	CROT	-0.803780326	4.17E-15
MRPS26	0.709071171	2.00E-41	DAPK1	-0.803850909	0.009638238
URI1	0.708840594	1.58E-63	RAB43	-0.803934497	2.91E-20

AC012146.1	0.708669057	0.035698288	ASB16-AS1	-0.804539785	0.000107241
LRRC8D	0.708231467	6.59E-31	ARHGEF10L	-0.80598969	2.57E-14
THRA	0.707782194	7.71E-05	GDPGP1	-0.806649471	0.001248628
PLK1	0.707536295	1.94E-87	UBALD2	-0.80726205	1.23E-27
URB1	0.70748369	4.90E-49	LRRC56	-0.807349084	0.001382311
CHCHD10	0.706473518	1.16E-23	MISP3	-0.807351741	4.83E-11
WASF3	0.706347181	6.27E-12	H2AC6	-0.807409564	4.91E-18
MTPAP	0.706209702	5.75E-27	GPC1	-0.807463066	2.04E-22
SLC25A26	0.705933641	1.84E-16	PPP1R13L	-0.807680628	1.74E-37
DDX18	0.70567669	9.32E-72	PPP1R14B-AS1	-0.807799832	0.002589063
GPR180	0.703906673	1.60E-15	LHPP	-0.808207547	3.99E-11
TFAM	0.702706541	1.04E-53	AC034193.1	-0.808338418	0.008986773
POLR1D	0.702348325	1.97E-49	KIF3C	-0.809142221	5.89E-09
SBDSP1	0.701770009	7.79E-26	MOB3C	-0.809495335	7.33E-08
EBPL	0.700983793	1.67E-25	HLA-DRB5	-0.809516286	0.039855522
USP7	0.700571165	4.70E-93	PLXNB2	-0.809595106	6.28E-102
GNPNAT1	0.700445487	1.39E-69	AL136295.7	-0.8098757	0.019494884
SKA3	0.700383897	4.88E-35	CCNO	-0.810287351	6.60E-12
VDAC2	0.700197053	9.17E-79	TECPR1	-0.811013164	2.30E-22
CYB5D1	0.699742849	1.13E-21	FBXO27	-0.811122928	0.043920216
RPL7L1	0.699688191	1.22E-95	GLP2R	-0.811392773	0.018081809
PFAS	0.699517329	1.24E-42	PRSS8	-0.812345604	4.14E-33
SMAD6	0.6990434	9.34E-06	OR2A9P	-0.812360192	0.006003401
FAM72D	0.698957352	5.33E-15	GUSB	-0.81247987	8.95E-61
CDCA7L	0.698744476	3.34E-36	CAPG	-0.812814795	1.19E-43
TEX10	0.698686516	4.17E-46	GMDS-DT	-0.812833183	0.037672574
SPATA5	0.698228409	3.20E-09	AC023158.1	-0.812976681	1.40E-06
TRMU	0.697994722	2.73E-30	MICB	-0.813000378	5.18E-09
AK2	0.69754213	2.64E-70	SYDE1	-0.814057145	1.52E-16
DNAJC11	0.696537446	9.84E-46	KCND3	-0.814390736	0.017239888
RPF1	0.696388328	2.97E-19	TCIRG1	-0.814614627	1.29E-10
ACTR3B	0.696341123	2.14E-21	REEP2	-0.81471001	0.000907678
ATP13A3	0.696066622	1.03E-88	NUDT18	-0.814867647	9.95E-07
STX2	0.695924496	2.91E-13	AC098934.1	-0.815235871	2.47E-05
TDG	0.695919477	2.83E-52	EPHA1	-0.815833194	1.83E-29
ELOF1	0.69525384	2.01E-47	RSPH1	-0.815964634	3.12E-06
GPN3	0.695183104	2.25E-16	LINC01719	-0.816482463	0.000700692
PRPF4	0.694562563	9.63E-47	FLNA	-0.816579083	3.29E-113
FUT10	0.694443795	6.04E-06	PRRT1B	-0.81693775	0.006260891
MTREX	0.694270959	4.21E-71	TRIM45	-0.817428573	2.49E-18
PSMD14	0.693401427	1.79E-57	PLXNA3	-0.817704355	6.88E-36
C1orf52	0.69334398	1.37E-15	BICDL2	-0.818433735	5.07E-25
EIF4E	0.692940154	1.70E-64	LHX2	-0.818541269	1.90E-05
MIEF1	0.692643084	1.28E-32	TMEM53	-0.818933116	0.000146479
AP000944.5	0.692447077	5.64E-08	KIF13B	-0.819023986	1.62E-16
CDIP1	0.69094957	0.044248896	AMZ1	-0.819280426	1.88E-11
QTRT2	0.690661389	1.56E-41	TMEM175	-0.82004884	5.39E-12

UTP18	0.69018648	3.38E-37	PTK6	-0.820676276	7.59E-18
ALKBH8	0.690178274	1.05E-11	IDI2-AS1	-0.821261503	0.004446749
GPATCH4	0.689963806	2.93E-44	TMPRSS13	-0.821497255	2.69E-17
MRPL32	0.689941891	1.91E-29	ZNF747	-0.821837831	1.12E-08
AC022384.1	0.688943368	0.00101599	TNRC6C-AS1	-0.821936634	0.000166316
SRPRB	0.68828594	2.80E-65	SEPTIN10	-0.823147985	4.51E-13
KIAA1958	0.688031766	1.42E-13	MAP3K12	-0.823177032	1.16E-14
LAPTM4B	0.687591747	1.83E-91	SNN	-0.82345253	9.63E-17
ZNF354A	0.687075221	7.40E-25	SARDH	-0.824228891	0.034702105
ZCCHC7	0.686132695	1.64E-14	OGDHL	-0.824493413	0.018719737
POM121	0.686001565	7.16E-57	GTF2IRD2B	-0.824547073	1.91E-18
POLR3D	0.685885764	4.80E-25	CRACR2B	-0.824764952	7.81E-13
TOMM22	0.685753185	1.99E-36	SH3D21	-0.824915444	4.99E-16
PDCD5	0.685668293	7.54E-35	FADS3	-0.824994754	9.24E-21
RIF1	0.685284251	5.46E-53	ARHGAP27P1-BPTFP1-KPNA2P3	-0.825051039	2.29E-08
NPL	0.685222339	0.035730096	BCL6	-0.825097392	1.54E-15
ZYG11A	0.685206823	7.66E-10	AL356056.2	-0.825586306	1.29E-06
NOL7	0.684613593	2.69E-51	SDC4	-0.826000337	1.16E-105
NOL9	0.683965722	1.75E-22	OAS3	-0.826199551	7.32E-31
CHRNA5	0.683492652	1.93E-26	FAM43A	-0.826979636	4.25E-05
ZFAND4	0.68277499	0.041522091	CD24	-0.827432068	2.39E-143
TRMT6	0.682661618	4.03E-17	FIBCD1	-0.827849698	1.32E-40
DUS4L	0.682555795	4.12E-15	PLEKHA6	-0.827855643	1.27E-22
ANKRD16	0.681954872	5.10E-10	SEMA4F	-0.827960183	3.21E-09
PRDM6	0.681738417	0.002756273	WDR31	-0.828133789	0.000245334
ATP6V1C2	0.681591103	0.010015623	CITED2	-0.828740968	7.75E-53
POLR3E	0.681310218	7.07E-39	GPRASP2	-0.829013754	1.42E-06
TRMT5	0.681230142	6.33E-25	B3GNT3	-0.829120924	1.30E-32
MRPL21	0.680967496	3.54E-28	S100A10	-0.829250649	2.70E-121
EIF5B	0.680582323	9.53E-68	QPCT	-0.830110706	0.00042374
PLCD4	0.679329784	0.00334183	DMC1	-0.830532417	0.017208009
KDM2B	0.678659872	1.73E-41	PLEKHB1	-0.830712226	2.50E-07
CCNB1IP1	0.678611384	7.68E-19	TENT5A	-0.831401478	1.24E-35
MRM1	0.678216583	2.30E-08	HLA-F	-0.831992442	1.53E-05
SLC30A1	0.678047903	1.13E-31	SMOC1	-0.832160029	0.007629378
PRPF39	0.677481692	3.54E-39	CUL9	-0.832204003	1.01E-26
BMP7	0.677398419	1.69E-56	CCDC159	-0.832431386	2.25E-05
MCRIP2	0.677190173	7.26E-24	AL132800.1	-0.833202434	0.022618851
METTL16	0.677166026	3.43E-39	SLC12A5	-0.833837263	5.68E-07
UMPS	0.676517161	2.21E-48	RBMS2	-0.833944826	7.18E-08
OSGIN2	0.676111602	3.62E-37	MIR1915HG	-0.834098039	2.66E-09
MGST1	0.67518167	4.31E-24	C4orf33	-0.834580288	0.000932219
RIOK2	0.674993025	1.42E-28	TCAF2	-0.835321951	0.003566457
RCHY1	0.674930959	1.83E-18	TMEM125	-0.835958571	8.63E-11
MICAL2	0.674878076	9.83E-08	MMP24OS	-0.836044348	1.59E-26

AC012360.3	0.674746683	7.71E-05	CRYBG3	-0.836172	5.10E-05
TIMM23	0.674666692	5.64E-41	TK2	-0.836701855	4.17E-07
LRRC61	0.67442726	1.07E-14	CLGN	-0.836882851	0.012117759
SLC9A3	0.67438408	3.25E-08	CCDC170	-0.837044155	1.53E-19
MTERF3	0.674201094	1.76E-16	ATP1B1	-0.838172495	3.55E-62
BAZ1A	0.67340969	2.82E-66	PLCH2	-0.838627197	0.015847327
MTFR1	0.673141697	2.89E-43	CFAP53	-0.838863697	0.033856505
TACO1	0.672947943	8.79E-33	SPINK5	-0.839246685	0.018481603
RNA5-8SN1	0.672318293	0.013730039	FMN1	-0.839357469	2.34E-10
EMG1	0.671953201	2.16E-35	AC068580.4	-0.839567792	2.91E-07
CHD1	0.671752113	5.04E-46	EPOR	-0.839904613	9.57E-06
MPHOSPH6	0.670764789	2.00E-21	IL17RC	-0.840011473	3.93E-19
HMOX2	0.670634593	4.01E-50	OPLAH	-0.840231164	5.19E-06
CA5BP1	0.670000977	2.44E-05	ARSG	-0.840397527	1.51E-09
FP565260.1	0.669908887	1.30E-28	AC067930.8	-0.84183557	0.002036732
LYPLA1	0.669735575	5.51E-46	KCND1	-0.84196927	0.005023782
UBE2O	0.669573012	2.20E-33	ATP2A1	-0.842283579	0.026638037
ZNF584	0.668465297	3.37E-09	ALKBH7	-0.842317935	7.51E-16
RPP38	0.667736149	6.66E-16	TEN1-CDK3	-0.842326352	0.016483883
DOHH	0.667323508	2.61E-16	VIPR1	-0.842344147	2.93E-07
DDIAS	0.667102795	2.35E-22	AC004233.2	-0.842646572	4.00E-12
TMEM38B	0.666991048	1.56E-28	DSC2	-0.843093099	2.04E-31
LIAS	0.666656019	2.93E-06	RAB32	-0.843276831	3.51E-17
GRSF1	0.666633849	1.29E-74	RABAC1	-0.843320491	4.24E-29
ZBED9	0.665755242	0.000475958	HIVEP3	-0.843427466	2.08E-05
UTP11	0.665417329	3.19E-21	LYPD6	-0.844531717	4.52E-18
KAT2A	0.665097597	8.45E-36	GPR37L1	-0.844693541	4.80E-08
KLHL8	0.664505013	3.44E-33	AL732372.2	-0.844765492	0.007860666
DOC2A	0.664345369	5.25E-08	SP6	-0.844864777	0.008238179
NOTCH2	0.664151861	2.17E-52	SDSL	-0.846105505	7.96E-30
SPAST	0.664067327	9.08E-19	ITGA3	-0.846240821	3.97E-104
STK32C	0.663897781	4.73E-14	IRF5	-0.846295313	1.85E-09
SRRD	0.663743625	2.14E-17	SLCO3A1	-0.846435322	3.13E-07
COQ10A	0.663205189	2.30E-06	LINC01963	-0.846574415	5.48E-06
EIF3B	0.663168182	6.37E-89	SEMA3F	-0.846693942	2.12E-16
TRAF3	0.662124899	1.73E-49	APOD	-0.846930203	4.74E-07
SGTA	0.662065093	9.47E-40	KREMEN1	-0.847106824	4.50E-12
OIP5	0.661819522	1.15E-07	AL118558.3	-0.847596057	0.004113998
NUP188	0.661572451	9.10E-64	BAIAP2-DT	-0.847879432	4.51E-12
GCSH	0.661502231	9.23E-59	ZNF112	-0.847964824	0.013103547
CHUK	0.661270494	5.64E-36	ZNF862	-0.848688304	4.73E-16
NETO2	0.660672888	5.91E-30	SPATA20	-0.84917444	7.49E-53
ZNF586	0.660479232	0.022991239	ADGRG6	-0.849296176	1.41E-119
ZBTB44	0.660315944	1.60E-23	AL441992.2	-0.849750029	0.003242605
PAX9	0.660260357	3.83E-11	NLGN2	-0.849776321	8.46E-32
SLC19A2	0.660122744	1.62E-15	DOCK8	-0.850276124	6.29E-15
RABGGTB	0.659703647	2.43E-29	H19	-0.850460945	2.87E-06

DCAF13	0.658570966	1.65E-40	SNX29	-0.850496966	2.79E-16
HMGXB3	0.658356233	2.37E-42	AL133216.2	-0.850723637	0.001417352
RRS1	0.658211188	8.00E-35	BAMBI	-0.851309498	1.68E-68
DPH5	0.658082578	1.63E-12	TMPRSS2	-0.851333814	2.30E-33
WDR36	0.657898622	3.53E-37	CCDC171	-0.852111536	0.021775451
HOMER2	0.657545136	7.13E-27	KLF2	-0.852378442	0.010490971
CEP85	0.65744828	2.82E-14	GGT6	-0.852505412	0.000330474
C9orf78	0.657396807	1.30E-50	RIMBP3	-0.852753883	0.02840143
ZNF593	0.657199392	9.43E-15	KIFAP3	-0.853121258	1.99E-07
CCDC66	0.656991335	1.48E-09	PACSIN1	-0.853486785	3.36E-14
SNRPD1	0.656274209	2.11E-37	BCAR3	-0.853810769	4.71E-16
YBX1	0.655586172	2.23E-87	LYN	-0.85411946	0.001830772
TFRC	0.655565233	2.87E-122	EXT1	-0.854392511	1.26E-12
CCT8	0.655356079	1.48E-61	MDK	-0.854793978	2.23E-48
EIF3M	0.65507588	2.50E-47	TSPAN9	-0.854939903	1.57E-16
GRWD1	0.654625486	2.85E-39	CD2BP2-DT	-0.855816246	0.013329451
SERTAD2	0.654615025	4.03E-15	LRP10	-0.856079099	1.21E-90
SDHB	0.653835858	3.79E-23	C1orf210	-0.856327879	1.22E-05
MRPL42	0.653711263	7.78E-36	SEZ6L2	-0.857157807	8.57E-63
CDK5RAP1	0.653696742	2.25E-20	CYTH2	-0.857176659	3.70E-31
CC2D2A	0.653177274	7.04E-07	CCDC153	-0.857270791	0.000746032
APIP	0.653159763	1.62E-15	GGACT	-0.857580886	0.001104253
B4GALT5	0.653115248	9.58E-56	ZNF365	-0.858335949	7.72E-07
BOLA2-SMG1P6	0.652697724	1.46E-27	CAMK2N2	-0.858444507	3.78E-08
CGAS	0.65257456	2.28E-16	AKAP5	-0.85892728	0.001022648
RXYLT1	0.652372704	7.34E-32	PLXDC2	-0.859119892	0.00092653
TRIT1	0.652164381	1.43E-12	TP53TG1	-0.859396955	2.91E-10
CNOT7	0.651622088	1.17E-48	SLC12A6	-0.859433034	7.28E-11
EXOSC2	0.650973861	8.15E-27	PTAFR	-0.860024739	0.000196066
H2BC11	0.650723112	0.037679114	CBX7	-0.860297892	2.66E-14
ZCCHC2	0.65049356	4.32E-14	RNF215	-0.860409553	4.16E-13
MZT1	0.650315182	1.69E-20	DLC1	-0.861863646	2.36E-08
DHX34	0.650306707	4.05E-22	ARHGAP29	-0.862225065	6.82E-26
SNHG17	0.649850065	5.36E-18	FAM102A	-0.862947105	4.39E-84
MAX	0.649817574	1.28E-35	SLC2A12	-0.86419993	0.003557251
AATF	0.649692166	7.66E-39	FAM114A1	-0.864285651	4.25E-07
FASTKD5	0.649118231	1.29E-13	IDNK	-0.86476815	1.24E-05
EFHD2	0.648051563	1.08E-27	MLPH	-0.864900516	1.20E-103
SELENOI	0.647699984	3.28E-46	PTK2B	-0.864915379	2.05E-06
EIF2S2	0.647686591	1.06E-51	SGMS1-AS1	-0.865747116	0.001695061
CCDC85C	0.647588273	2.07E-54	CDKN1A	-0.865802101	6.16E-28
YDJC	0.647574819	5.30E-29	BAIAP3	-0.865883629	3.81E-06
TIMM9	0.647551892	3.18E-17	NOX5	-0.866311278	0.013640338
NIPA2	0.647509734	1.31E-46	URAHP	-0.866325625	0.002963669
STARD7	0.64702182	8.20E-81	LRTOMT	-0.866593662	1.15E-07
BZW2	0.646723545	6.50E-48	C5	-0.866715541	7.31E-13
DDX49	0.646378737	2.73E-35	GGT7	-0.86694099	4.53E-11

GEMIN6	0.646327902	2.43E-14	TIMP3	-0.867085355	1.92E-05
GNL2	0.64608681	1.64E-42	CARD14	-0.867148429	1.87E-14
NIFK	0.64591869	2.42E-27	TMEM254-AS1	-0.867458752	0.000381327
AASDHPPT	0.645676976	2.02E-23	AC073508.2	-0.867669877	5.67E-05
ATP5F1B	0.645649998	4.96E-103	MAP1B	-0.867912602	0.012102944
TIMM50	0.645636309	1.42E-32	COL11A2	-0.868303562	2.76E-07
EIF2S1	0.645585925	4.13E-60	OSBPL5	-0.868338513	6.52E-12
NOM1	0.645353906	1.03E-33	ARHGAP30	-0.868913793	0.010881484
POLR3A	0.644738091	4.36E-29	SUOX	-0.868937633	1.11E-16
MTDH	0.643975019	6.33E-73	MAGED1	-0.869132248	2.67E-115
C16orf91	0.643358794	3.52E-12	KRT81	-0.869428121	2.10E-147
DDI2	0.642937013	1.07E-23	ERMAP	-0.869864503	2.77E-11
THOC5	0.642797093	2.06E-31	LPP-AS2	-0.870093414	0.00021265
CEBPG	0.642657847	2.31E-35	KRT80	-0.8701972	2.17E-182
ZNRD1	0.642321633	2.79E-10	FAM229A	-0.870219725	0.019796528
DNAJC7	0.642271072	3.07E-29	GSN	-0.870380594	1.83E-92
MYCBP2	0.641909571	5.99E-27	TSPAN31	-0.870730936	4.38E-24
FBXO25	0.641794306	1.50E-14	CD59	-0.871052852	1.06E-140
MRPS24	0.641777495	2.98E-34	FAM20C	-0.871238727	5.41E-10
PIGW	0.641777202	5.00E-21	F2R	-0.872058989	4.68E-06
ZNHIT6	0.641726747	1.05E-14	SEMA4B	-0.872377235	2.11E-35
FAM72A	0.640776831	1.18E-10	GATA3	-0.872718977	1.40E-115
NEK6	0.640589974	1.12E-21	NR4A1	-0.872836229	1.39E-34
SPATA5L1	0.64020801	2.07E-08	TSHZ3	-0.873026007	5.67E-05
BCS1L	0.640200123	1.83E-22	NINJ2-AS1	-0.874880633	0.003078658
GAR1	0.639307356	3.91E-06	FAM110C	-0.874891656	1.67E-35
TRUB1	0.639081861	3.21E-23	AC090114.2	-0.875436114	8.72E-17
NDUFC2-KCTD14	0.638929236	0.000652459	AC093001.1	-0.875592322	4.66E-22
IDH3A	0.638716746	2.21E-48	STAC3	-0.875680995	0.00890855
PTCD3	0.63796425	4.66E-36	SPSB2	-0.876009528	3.93E-05
PSME4	0.6379386	1.23E-49	B4GALT1	-0.876575392	1.74E-122
ATXN7L2	0.637371954	9.24E-10	ARNT2	-0.876656868	1.95E-51
MAP3K20	0.637286203	2.35E-24	DNMBP	-0.877149633	2.62E-12
TIMM13	0.636723456	8.44E-23	PTPRG-AS1	-0.877596751	2.46E-12
GTF3C4	0.636520753	1.10E-57	TOX2	-0.877842702	0.000306783
SERBP1	0.635681942	2.81E-93	PGM2L1	-0.878084148	1.00E-06
VKORC1L1	0.635497353	5.39E-48	PLEKHH2	-0.878157304	8.52E-06
GTF2E2	0.635471012	4.32E-24	ZNF517	-0.879503511	2.47E-11
PRKAR2A	0.635249489	2.22E-40	AP006222.1	-0.879599692	2.24E-06
MRPS35	0.634981622	2.68E-31	CLEC2D	-0.879694143	0.000241444
PLCH1	0.634728152	1.71E-10	APLP1	-0.879740453	9.50E-09
SLC7A6	0.63470643	8.16E-42	PLK2	-0.879836374	1.26E-78
AC068547.1	0.634035722	6.36E-08	MATN2	-0.880083616	7.07E-16
MRPL47	0.634023229	4.87E-26	RABL2A	-0.881184986	4.67E-05
PUS3	0.633940276	0.000198788	PLD2	-0.881348357	1.60E-18
AP000648.4	0.633651175	0.037359741	TTLL7	-0.881608884	0.000581931

DDX39A	0.633558101	2.95E-55	TCHH	-0.881991738	0.003823464
BMS1	0.632982955	1.65E-46	CCDC24	-0.882132994	1.97E-07
NDUFAF1	0.63279511	4.38E-10	ZNF493	-0.882659232	0.004960943
AK4	0.632643596	1.37E-35	RAB9B	-0.882699068	7.49E-05
ELAC2	0.632454647	8.70E-45	ADGRA2	-0.882791335	0.000303524
TUFM	0.632357156	4.76E-87	FCHSD1	-0.88287414	3.08E-15
CHKA	0.632061857	6.50E-20	ADIRF	-0.883238262	7.71E-32
TWISTNB	0.631578221	1.31E-20	FRK	-0.883765834	7.94E-05
CEP85L	0.631574006	0.000288871	TBX6	-0.884593637	0.004802935
CSNK2A1	0.631050072	1.01E-51	C4orf19	-0.884967945	3.44E-24
COIL	0.630869314	3.05E-30	TGM1	-0.885737511	1.16E-10
UTP14A	0.630780716	5.73E-42	NECTIN2	-0.885900493	1.09E-69
DYNC2H1	0.63068785	4.61E-10	CTSD	-0.8867854	1.66E-27
BIRC5	0.63037594	4.48E-38	AP000866.1	-0.886930202	0.020859715
SMS	0.630245294	1.58E-50	SMARCD3	-0.889163186	2.81E-14
PSMD12	0.629680629	4.53E-58	KCNK15	-0.889474119	1.85E-90
C16orf87	0.629565546	1.99E-11	BCAS1	-0.889799657	2.73E-144
PHKA1	0.629543893	6.31E-28	RAP2C-AS1	-0.890685468	0.010950333
EMC3-AS1	0.629073907	2.16E-05	SQOR	-0.890952938	9.14E-18
NUP155	0.628530518	5.69E-32	NDRG1	-0.891348774	2.70E-15
BRMS1	0.628262783	5.32E-36	ZNF821	-0.891978634	8.02E-09
MRRF	0.628190864	8.92E-25	ADIRF-AS1	-0.892155248	7.60E-06
CNTNAP2	0.628024156	0.024718716	OLFM2	-0.892341758	1.70E-07
CLUH	0.627755049	3.69E-59	ANKRD34A	-0.892564707	9.22E-05
FP236383.5	0.627710123	0.006329008	ADCK5	-0.893082222	2.70E-20
CDK6	0.627694795	2.69E-19	HDAC5	-0.893228427	1.30E-07
MRPL14	0.627256878	3.15E-33	TNFRSF19	-0.893666984	0.007040721
MRPS9	0.627249093	4.97E-23	AL162258.1	-0.89454449	0.001194765
RNASEH1	0.627239517	1.30E-20	SEMA4A	-0.894666225	1.57E-11
EXPH5	0.6269713	0.012835098	WLS	-0.894949975	1.90E-05
PRKX	0.626933947	4.65E-22	ATP6V0A4	-0.895031992	3.23E-12
NUP42	0.626734887	7.18E-12	IQSEC2	-0.895081421	1.91E-14
DDX56	0.626438153	8.55E-51	PTPRE	-0.896280407	2.41E-14
PDK1	0.625840368	1.35E-12	GDPD3	-0.896372366	7.17E-28
SSBP1	0.625685132	6.59E-38	PBLD	-0.896499863	9.46E-09
FBL	0.625160104	5.01E-16	PDGFRL	-0.897443189	4.24E-11
NAMPTP1	0.624824508	4.01E-10	FRG1BP	-0.898755633	1.04E-25
KRTCAP3	0.624696153	1.52E-05	PRAF2	-0.901048959	6.21E-20
MPHOSPH10	0.624652142	3.57E-33	MICALL2	-0.901356779	1.28E-35
CEBPB	0.624146314	1.05E-20	LINC01137	-0.901404683	0.001384218
NKRF	0.623023682	8.16E-38	VEGFC	-0.901422834	1.29E-08
MRPL48	0.622601354	2.11E-15	RARA	-0.903259819	6.54E-86
NDUFAB1	0.622218606	1.91E-34	CUEDC1	-0.90329119	4.33E-51
MRPL16	0.622061922	5.24E-33	ACCS	-0.903462999	3.37E-06
MRPL46	0.622046799	6.74E-16	TFPI	-0.903745082	5.94E-53
MCMBP	0.622037109	1.16E-57	ARHGAP4	-0.903928011	1.58E-08
MTURN	0.621999209	7.90E-11	PPOX	-0.903928027	1.63E-09

NHEJ1	0.621698191	1.53E-05	TMEM229B	-0.904588185	1.96E-22
RPUSD2	0.6214978	2.97E-08	ELF3	-0.904914654	3.70E-81
RIDA	0.62149086	2.98E-15	SLC2A10	-0.905655681	5.13E-42
FBXO31	0.621385482	5.78E-25	AC253536.1	-0.905676863	1.87E-05
FP236383.4	0.620721783	0.007166999	ESR1	-0.905966094	1.30E-86
BCCIP	0.620347514	1.17E-34	ECHDC2	-0.906157484	3.07E-16
STK17A	0.620319405	1.30E-11	AC108134.1	-0.906271679	1.92E-05
ANAPC1	0.62023076	2.66E-48	AL049834.1	-0.906440431	1.10E-05
PCSK6	0.620170799	5.32E-10	SPAG4	-0.906746036	1.72E-06
TMEM186	0.620146859	6.64E-17	DNAH1	-0.906882964	0.004632925
ELOVL6	0.619535488	9.19E-21	TOM1L2	-0.906993245	1.67E-72
AC125807.2	0.619417645	3.72E-06	BTC	-0.907839811	7.82E-11
GEMIN7	0.619258069	9.18E-19	MAGI2	-0.908552771	9.17E-05
DESI2	0.619174114	8.53E-18	AC104825.1	-0.910673494	0.012293894
MRPS34	0.618811119	3.21E-52	GPR87	-0.910929021	0.004900419
SMN1	0.61863713	1.18E-30	DNAJC4	-0.911435504	2.72E-14
MIS18A	0.618514301	1.28E-24	PCDHA11	-0.911641691	2.34E-16
HSPA4	0.618413653	1.14E-61	NEAT1	-0.911692336	5.31E-05
TMX2	0.618245545	2.88E-33	SLC66A3	-0.911780559	1.13E-24
RIPK2	0.61819146	4.39E-20	AC096887.2	-0.91231757	0.027775926
POLR3F	0.618027926	3.83E-17	LDLRAD4	-0.91276087	4.79E-24
EIF4EBP1	0.617699255	2.18E-37	PLA2G4C	-0.913551083	0.003415061
ATL2	0.617258775	8.11E-24	SELENOM	-0.913775068	0.000509625
MTFMT	0.616741332	1.65E-13	ITGA5	-0.913784488	4.54E-27
NFATC3	0.616723448	8.63E-31	KIFC3	-0.914653374	1.67E-22
COLGALT1	0.616348487	5.20E-44	FMNL1	-0.916035951	2.07E-08
FEM1A	0.615578919	1.32E-08	TTLL3	-0.916094681	9.57E-16
MRPL10	0.615512986	1.89E-31	LMNTD2	-0.916210282	0.000414693
CCT2	0.615319445	6.25E-66	CTSV	-0.91624371	1.36E-08
C2orf27A_1	0.614871254	1.53E-05	SMIM14	-0.917086821	4.69E-60
SPG21	0.614862072	3.79E-38	TRGC1	-0.917454011	1.14E-60
PRELID1	0.61485453	3.15E-63	DMTN	-0.917589829	8.37E-21
MAP4K5	0.614754991	1.52E-32	IL17RE	-0.918716923	4.77E-05
PRKAR1B	0.614715574	3.94E-38	PSD	-0.919203762	0.000125393
RPP30	0.614591893	1.22E-16	PRX	-0.919855136	1.42E-05
MPLKIP	0.614095372	1.92E-13	SYT10	-0.919896749	9.96E-32
SREK1IP1	0.614060146	4.17E-16	MYH14	-0.920061673	4.17E-81
TTC4	0.613914905	2.89E-15	CBLB	-0.920181185	1.37E-14
TARS3	0.613575812	7.80E-06	RALGPS1	-0.921083697	4.21E-13
METTL4	0.613554904	7.67E-08	OSCP1	-0.921339312	0.001486064
FP671120.7	0.613251518	0.008475425	AL732372.1	-0.92170391	0.000142841
ITGB1BP1	0.613223785	6.80E-38	DNAH7	-0.921762014	0.005760674
MAP7D3	0.61319251	8.35E-24	PIGZ	-0.921769539	0.000223288
NAA20	0.613022085	2.40E-50	GMPR	-0.921835275	0.000475919
COPS2	0.612301619	3.43E-27	CRYL1	-0.922257179	2.11E-16
FAM92A	0.612297229	5.72E-12	TMEM91	-0.922518836	0.010552074
RAP1GDS1	0.612221208	6.85E-24	ST8SIA6	-0.922562226	1.18E-19

RAD18	0.61129297	2.05E-21	SELL	-0.922826801	0.006660713
GMPS	0.611157989	6.05E-65	LINC00857	-0.923286312	0.008476885
NUP88	0.610839334	1.31E-27	FA2H	-0.923425826	1.06E-05
FAM207A	0.610722239	3.79E-21	CASTOR3	-0.923440029	9.11E-25
ALG5	0.610457824	3.39E-15	SERPINB8	-0.923457899	0.000184156
TXNL4B	0.610446269	1.86E-13	HLA-DRB1	-0.924436768	8.34E-27
ME1	0.609995799	1.21E-31	RHOB	-0.926018904	1.41E-63
NUDT15	0.609784138	6.18E-18	PRR15L	-0.926161972	2.55E-24
ENTPD1	0.609691198	5.23E-12	PRKD1	-0.926775579	7.56E-06
TARS1	0.60963798	9.56E-57	GDPD5	-0.926918181	9.01E-05
FOXN2	0.60956982	3.01E-14	FPR3	-0.927576724	0.007031462
UBE2K	0.609430818	6.66E-47	PNRC1	-0.928171947	3.82E-33
HAUS6	0.609087166	1.74E-25	CEMIP2	-0.928543052	1.20E-61
ZFAND1	0.60891811	2.47E-27	YPEL3	-0.929102701	4.33E-54
NSUN5	0.60879733	1.22E-34	DOCK8-AS1	-0.929441583	0.000173543
CBX2	0.608512447	1.65E-17	ANO8	-0.92979009	1.97E-12
REL	0.608018254	1.87E-10	CLDN1	-0.930081293	1.63E-45
C3orf52	0.607933021	0.002968407	LDHD	-0.932168341	0.000177852
RPL22L1	0.607923556	7.78E-25	MIR9-3HG	-0.932707329	2.85E-07
SMIM13	0.607687038	1.06E-15	FBP1	-0.932734646	4.21E-74
LMO4	0.60752519	4.68E-10	KIAA1211L	-0.932766191	1.40E-27
DYNC1LI1	0.607461474	7.09E-20	CD109	-0.932819729	8.64E-18
CFAP298	0.606840972	1.61E-32	CREBRF	-0.933043528	2.35E-11
AC097448.1	0.606349506	3.97E-05	UPK2	-0.933097915	1.76E-42
ABT1	0.606309656	2.16E-23	ACSS2	-0.933223728	9.51E-25
MALSU1	0.606100166	5.24E-13	ARNTL	-0.933245644	3.00E-12
LONP1	0.605990614	1.41E-43	PALLD	-0.933251583	4.82E-36
KANSL2	0.60558181	3.29E-15	TNFAIP8	-0.933388055	2.68E-22
PIM2	0.605565095	3.65E-07	LTC4S	-0.933536308	0.020366811
CCNJ	0.605120124	9.89E-15	AL359258.2	-0.934406633	0.001521589
PHB2	0.60481711	3.07E-43	BCL3	-0.934412912	1.84E-18
UBE2V2	0.604802474	3.21E-37	EVA1B	-0.934451856	1.76E-07
LSG1	0.604775367	6.75E-43	KRT7	-0.93455597	4.33E-44
MCPH1	0.604466312	3.44E-11	GCOM1	-0.935039329	1.80E-07
SET	0.604284394	4.77E-88	FAM161B	-0.936375375	1.25E-08
RAN	0.603616071	6.12E-73	MYZAP	-0.936446348	9.09E-11
AL358472.6	0.603545078	7.71E-08	WDR66	-0.937212901	0.000356055
APTX	0.603437274	4.54E-14	TRIM2	-0.937314899	0.000136732
LINC00662	0.603335807	5.84E-05	ALDH3B1	-0.937389985	1.99E-11
FRAT2	0.603039923	7.41E-18	ZNF117	-0.937439519	4.47E-18
HSPBAP1	0.60297429	0.00083735	ITGA6	-0.938009886	9.54E-12
PPP1CC	0.602771419	2.46E-58	ERV3-1	-0.938762521	5.08E-16
PITPNC1	0.602716383	2.43E-23	PRRT3	-0.939339239	5.26E-41
MTX2	0.602629753	3.92E-18	LAMA3	-0.94158743	0.00313427
PSME3	0.602577186	1.14E-52	DGCR9	-0.941723449	0.019663046
LSM11	0.602295786	9.41E-10	TRIOBP	-0.941938203	1.87E-40
ZNF674-AS1	0.602280712	0.000130086	FAM153CP	-0.942016579	2.47E-05

MYO10	0.602142606	5.84E-16	VASH1	-0.942918834	1.76E-14
ARIH2	0.601200536	3.33E-36	RHBDF1	-0.943351277	1.08E-57
RHBDF2	0.600896502	2.52E-19	NBEA	-0.944705028	1.07E-17
FAIM	0.600766848	3.39E-10	LXN	-0.94558303	2.04E-103
GEMIN2	0.60035202	1.33E-13	PADI2	-0.946166355	0.001845077
SNHG16	0.600285856	1.33E-29	DGCR6	-0.946405328	0.000379847
FP671120.4	0.60019633	0.024650089	SHFL	-0.947260392	2.12E-12
NCBP2AS2	0.600172187	1.91E-28	AC132812.1	-0.947517271	0.001076865
FGFR1OP	0.599625104	4.86E-16	SALL2	-0.947768824	0.01321344
CEP83	0.599587871	2.01E-13	NECAB1	-0.948517563	9.37E-10
STX18-AS1	0.598725105	0.041996295	CES3	-0.949049503	0.0026265
NCLN	0.598470877	7.45E-46	BTG1	-0.949100679	1.63E-63
CEBPD	0.597256159	9.52E-06	TP53INP2	-0.949388379	1.56E-33
CCNA2	0.596923604	3.80E-44	LINC01257	-0.950381317	0.028630836
DDHD1	0.595932655	2.90E-21	SSH3	-0.950955422	6.76E-78
KPNA4	0.595591199	1.86E-50	UNC5A	-0.951191357	0.005151264
EDRF1	0.595304451	4.08E-17	TMOD1	-0.952930983	0.00012292
CUL1	0.595159003	2.83E-49	CAPN5	-0.953242306	8.08E-33
PARL	0.594980844	2.26E-28	MIR210HG	-0.953600479	2.64E-05
PPIH	0.594781077	3.83E-12	HSH2D	-0.954188139	8.44E-19
ZNF589	0.594383738	5.47E-06	NAP1L2	-0.955457695	0.000707975
AC012615.1	0.593770075	0.002066648	GLDN	-0.955728349	1.77E-05
ATP1B3	0.593158661	6.42E-37	DENND3	-0.956481175	9.83E-13
EFNA3	0.593015954	1.14E-05	AC092295.2	-0.957281178	0.022763148
TBC1D30	0.593004722	2.43E-45	NEK8	-0.957470652	2.35E-08
ZNF695	0.592977646	0.000929548	AC018665.1	-0.957899752	0.001145826
ADSS2	0.592740618	4.09E-25	CASTOR1	-0.957978386	0.000393498
FARSB	0.592554167	2.57E-42	APAF1	-0.958550004	2.34E-11
DLX1	0.592325028	1.64E-06	MC1R	-0.958945622	2.97E-24
DDX28	0.592321885	2.46E-14	PALM	-0.960137004	4.20E-33
RFC3	0.592203075	2.87E-32	AC084018.2	-0.96019457	0.00740213
GID4	0.592042129	1.37E-07	ELF3-AS1	-0.961053822	7.25E-07
FAM86B1	0.591793603	3.25E-06	MAGED2	-0.961710837	5.40E-76
CLPX	0.591773881	1.24E-29	CAV1	-0.963307068	7.36E-71
PELP1	0.591597496	1.64E-49	EPHA2	-0.964568028	7.77E-56
CENPP	0.591443722	3.25E-09	COL18A1	-0.964572518	1.57E-36
PTGES3	0.59131953	3.41E-51	FXYD3	-0.965473885	6.00E-63
RWDD1	0.591301449	6.19E-22	SFXN5	-0.966389125	3.87E-17
DIS3L	0.591170774	5.10E-13	TLE4	-0.966907938	0.003409299
MAPKAPK5-AS1	0.590045592	3.12E-11	GOLGA7B	-0.967008945	1.06E-05
WDCP	0.589828161	8.93E-08	LINC00461	-0.968136977	0.006499806
SDHD	0.589756876	1.78E-24	SLC22A17	-0.968218125	6.38E-07
EFTUD2	0.589343656	1.97E-43	PHF1	-0.968263764	9.44E-27
HDAC2	0.589071421	1.70E-44	DCDC2	-0.969226031	1.83E-88
STRAP	0.588562393	1.75E-50	AP003498.1	-0.969228805	0.000936136
DNTTIP2	0.588240254	2.00E-29	PCDH1	-0.971336488	2.05E-32
EIF3E	0.587645401	5.85E-69	MIEF2	-0.971514936	5.93E-20

TBCE_2	0.587566766	2.58E-05	AGR2	-0.974906032	1.44E-58
SF3A3	0.587388626	1.73E-31	AC138028.6	-0.974920172	0.001052048
SRM	0.587295703	3.93E-54	CCT6B	-0.975120139	0.001417898
ATAD1	0.586880447	1.90E-27	MYOF	-0.975460976	2.08E-182
MAPK6	0.586838185	3.40E-62	HRH2	-0.975969012	0.01054778
SPESP1	0.586523974	0.008114329	NPC2	-0.976122017	1.56E-39
RAE1	0.586467095	1.53E-43	AL358852.1	-0.976272116	0.015275186
C1orf131	0.586237915	3.08E-09	KRT18	-0.976305882	1.48E-209
FCHO1	0.586179067	5.78E-16	ULBP2	-0.976526601	1.14E-16
USP10	0.585700343	2.92E-53	NRP1	-0.976761579	8.42E-147
R3HDM1	0.585415979	3.99E-30	TYMP	-0.977364094	0.00152586
			FAM66C	-0.979697684	0.000657035
			MAGED4	-0.979873415	0.000620581
			EHBP1L1	-0.980922997	1.07E-18
			TMBIM1	-0.982435304	0.000109918
			TIMP2	-0.983207767	2.97E-32
			ANXA3	-0.983261054	5.09E-48
			CLCN4	-0.983789212	0.000768469
			F8A3	-0.984009194	0.01181847
			MAPK11	-0.984051448	1.41E-20
			MYO7A	-0.985274311	0.007032051
			ALOX15	-0.986058159	9.07E-12
			PALM2AKAP2	-0.986062468	8.90E-05
			IDUA	-0.986490353	1.68E-10
			TAPBPL	-0.986647865	4.17E-08
			ACER2	-0.986706813	0.000522451
			AMTN	-0.987276919	0.000302677
			SDCBP2	-0.987286384	2.16E-05
			GABRP	-0.987608948	2.40E-09
			CCDC191	-0.987674333	1.34E-06
			LRRC75A	-0.987901751	1.03E-05
			FO681492.1	-0.988906401	0.000792666
			ZNF792	-0.988959944	0.000415845
			KIAA1217	-0.989539349	4.76E-16
			SPACA6	-0.989614531	0.00070475
			PAQR6	-0.991778744	2.79E-09
			AC018521.5	-0.991800764	0.014006021
			SYNE1	-0.99201731	0.000340449
			C2CD4C	-0.992465733	3.95E-05
			TNS3	-0.993895385	9.31E-85
			TENT5C	-0.995806695	6.52E-12
			SDC2	-0.997274155	7.57E-17
			GPR39	-0.997677016	0.002145192
			MCF2L	-0.998183234	1.42E-13
			PMEL	-0.998342811	0.000467453
			CACNG4	-0.998435195	2.43E-53
			RECK	-0.998747395	0.000168498

OBSL1	-0.999050722	1.06E-73
H4C8	-0.999137026	2.01E-08
H6PD	-0.999516143	1.44E-23
CFAP206	-0.999648591	0.001772257
NRSN2	-0.999991504	4.80E-06
IGDCC3	-1.000465092	0.000110101
ZNF462	-1.000660624	8.02E-18
AGAP11	-1.001311034	0.000284529
PALM3	-1.001337651	3.90E-10
SLIT2	-1.001481179	0.000315772
AC016682.1	-1.001707371	0.00434127
EMID1	-1.003467922	2.32E-09
FMO5	-1.003683264	0.000328136
LMCD1	-1.004081518	6.70E-25
STRIP2	-1.004428729	0.000751658
LINC01135	-1.004584139	0.004617675
TJP3	-1.005366709	9.92E-29
SERPINA11	-1.005620163	0.002423333
LRRC24	-1.007177034	4.89E-08
KLC3	-1.007562733	3.12E-05
GPR132	-1.007600646	0.002007152
AC126564.1	-1.007780452	3.74E-13
IL1R1	-1.008324028	5.51E-28
PPP1R18	-1.008644249	1.09E-39
CLIP2	-1.009109285	1.84E-06
AL158211.5	-1.010225167	0.001976678
CAPS	-1.010403685	0.001936425
H3C15	-1.010531863	0.000740516
SLC12A5-AS1	-1.010697517	0.003649791
PARP10	-1.011949751	8.08E-39
FZD2	-1.012108918	9.95E-17
DHRS12	-1.012580599	0.0005655
PCDH9	-1.013063779	2.71E-09
FAM122C	-1.01346941	1.35E-08
MCC	-1.013769509	2.08E-07
KRT8	-1.014126736	2.55E-219
SCART1	-1.014754267	5.50E-05
SLC28A1	-1.01543959	0.001476806
TMEM86A	-1.016096329	3.71E-05
SEMA7A	-1.01652304	6.80E-09
SBK1	-1.018031125	6.29E-12
IQCJ-SCHIP1	-1.018249515	9.32E-08
C8orf58	-1.018682031	2.54E-15
S100A1	-1.018687374	1.34E-06
AC046134.2	-1.019598195	0.0004366
CISH	-1.019862799	2.37E-16
GPX3	-1.020386959	1.69E-15

SNTB1	-1.021441479	3.62E-06
MEIS3P1	-1.022848279	0.00040941
EVI5L	-1.024275072	1.95E-40
HDAC6	-1.02437651	5.50E-31
KIAA0513	-1.025440596	8.29E-47
CPQ	-1.025512321	0.00679824
EHD2	-1.027237321	6.20E-14
PLEKHG2	-1.027538512	2.40E-33
ANOS1	-1.02790604	0.000125013
LAMB2	-1.027934594	5.40E-95
BCAM	-1.028428523	5.97E-22
MOSPD3	-1.028824231	3.38E-25
KRT7-AS	-1.029096261	0.000193949
IGFBP5	-1.030151758	3.80E-83
EPAS1	-1.03026496	2.58E-105
LY6G5C	-1.030294862	0.00231595
FRAS1	-1.030403397	6.69E-05
SMAD3	-1.03104264	4.53E-138
APOBEC3B	-1.03170309	9.84E-09
ARSA	-1.031715271	8.67E-21
THBS3	-1.031806726	6.75E-20
IFI27L2	-1.032261873	5.30E-14
ANXA6	-1.033105342	2.14E-57
KIAA1324	-1.034481961	2.42E-72
AP001816.1	-1.034880487	2.07E-10
AL162458.1	-1.03540131	4.19E-11
TIGD3	-1.036552134	0.001271138
TTYH3	-1.036668307	4.24E-44
IGFBP4	-1.0370309	5.23E-46
MUC3A	-1.03724001	4.20E-20
TCEAL3	-1.037687884	3.66E-45
PLAAT3	-1.037694074	1.95E-18
EGFR	-1.038163928	1.03E-14
CBR3-AS1	-1.038169341	5.95E-12
TNFAIP8L1	-1.038797918	8.57E-09
VTCN1	-1.039410017	6.74E-09
ZNF211	-1.040169104	8.53E-06
SLC22A18	-1.040281574	2.27E-14
KCTD11	-1.040851893	2.03E-26
UNC13D	-1.041327326	4.00E-105
LRP1	-1.042191133	6.54E-09
FGD1	-1.043704321	0.001166699
ABCD1	-1.043911209	3.31E-07
AGRN	-1.044120539	1.32E-42
H2BC21	-1.044291076	1.34E-38
ZNF467	-1.04471172	2.19E-27
ZNF446	-1.045398475	6.95E-08

CAPN2	-1.045696208	1.16E-25
RASA4CP	-1.046984788	0.00809348
AC005332.5	-1.047338896	1.96E-07
CXCR4	-1.047547294	2.67E-09
AC021066.1	-1.048022581	5.69E-73
THNSL2	-1.048034994	1.32E-20
DKK1	-1.04803766	8.06E-102
RAB11B-AS1	-1.048669123	0.000798713
FAM214B	-1.048754254	1.42E-08
UPK3B	-1.048928474	3.33E-42
PLBD1-AS1	-1.048935797	0.00092221
C1orf116	-1.049883169	3.29E-08
AL139385.1	-1.050626839	0.001075678
L1CAM	-1.052831187	1.36E-131
THBS1	-1.053536343	3.01E-66
SYNM	-1.054661774	2.86E-10
TRGV9	-1.055233284	0.000201183
SNAI3-AS1	-1.055398509	0.000258081
FANK1	-1.055445803	7.21E-08
GPC2	-1.055488848	0.001050994
AC027601.1	-1.055499909	0.000143791
LYRM9	-1.056420607	3.12E-05
EMP1	-1.05679326	7.54E-06
ACBD4	-1.057536816	1.75E-10
TPO	-1.058940432	0.0001676
HHAT	-1.059864306	6.80E-07
ZG16B	-1.060229582	3.94E-21
CAMK2B	-1.060784644	6.04E-05
ABCC13	-1.061133905	0.000236648
EFR3B	-1.061321363	2.82E-05
WBP1	-1.062140166	3.17E-11
SH3TC1	-1.064039832	2.39E-11
TMEM198	-1.064100722	0.000236366
AC069281.2	-1.06420289	0.007702591
ACKR3	-1.064321134	1.62E-14
ABLIM3	-1.064415714	5.06E-24
APOLD1	-1.065092172	2.03E-06
CCN5	-1.065336024	2.06E-28
C2orf72	-1.065996583	2.14E-07
AC015712.6	-1.066738137	7.35E-18
ERICD	-1.067257851	3.60E-07
TMC4	-1.067712828	1.49E-35
RTN2	-1.067845256	0.000713409
ELFN1	-1.069310938	1.93E-15
EFNB3	-1.06966578	1.41E-12
APOBEC3F	-1.070698311	0.000820336
NFATC4	-1.070712651	4.24E-05

SYT12	-1.071546001	3.64E-74
MID1	-1.071606819	3.59E-69
CPT1C	-1.073135968	0.005647792
SEMA3C	-1.073436539	1.74E-186
ASB9	-1.073813216	0.001312134
RASD1	-1.074051492	4.03E-54
MRC2	-1.075422197	1.53E-17
C9orf152	-1.075947034	3.20E-19
CGNL1	-1.076206557	2.45E-07
PNPLA7	-1.077896075	1.53E-06
EPS8L1	-1.078054023	7.98E-44
H3C6	-1.078432983	7.68E-06
SLC16A13	-1.079997575	4.86E-08
KRT19	-1.080330755	2.25E-224
PHLDA1	-1.080912636	1.63E-35
DUSP10	-1.080921643	5.32E-05
TM7SF2	-1.082159322	3.32E-64
ASIC3	-1.08278813	2.71E-08
ETNK2	-1.083620317	5.89E-20
REEP1	-1.085550053	1.11E-05
AC147651.1	-1.085931277	1.61E-05
LMO2	-1.086320655	8.06E-06
NHS	-1.086837645	1.28E-39
MTMR11	-1.08707519	4.58E-22
CDKL5	-1.08908228	1.15E-19
AC139099.1	-1.090852571	2.70E-05
NAV2	-1.091206155	2.76E-134
CLEC3A	-1.09136515	4.39E-36
FAM189A2	-1.09194245	7.33E-09
CRISPLD2	-1.092083808	4.32E-05
MELTF	-1.092127998	9.40E-09
DIPK1B	-1.092920543	3.36E-09
DBN1	-1.093356678	1.66E-36
NFKBIZ	-1.093810429	2.20E-41
ZBTB22	-1.094093099	4.17E-26
C14orf132	-1.094151716	3.39E-25
MXD4	-1.095389488	4.25E-54
CEACAM6	-1.095815405	6.04E-186
CLU	-1.095982806	2.90E-179
RIN2	-1.09767087	1.48E-16
SCNN1A	-1.098932851	4.46E-13
AREG	-1.100242436	1.40E-34
LIN7A	-1.100324894	6.81E-10
ZNF396	-1.100443738	0.000650226
USP51	-1.101387376	5.49E-05
ZNF784	-1.101637362	2.56E-06
RPL23AP87	-1.103332137	0.000241141

NRBP2	-1.105153656	4.89E-09
TMEM45B	-1.105758185	6.15E-34
CEACAM5	-1.106666335	4.04E-08
STON1	-1.107014127	1.06E-07
GLTPD2	-1.107800012	0.000276642
CACFD1	-1.10849856	9.31E-37
PYROXD2	-1.108636639	8.48E-12
OCEL1	-1.109986006	6.04E-10
TEX22	-1.110041385	0.000824129
NFATC2	-1.110087384	7.57E-17
MAST4	-1.1104709	3.79E-82
CMYA5	-1.110609926	1.53E-16
SLC25A29	-1.110648852	6.65E-51
ORAI3	-1.111017206	1.09E-26
ANKRD6	-1.112901536	0.000931786
TXNIP	-1.113563403	1.50E-167
ARSD	-1.114453917	2.45E-56
APH1B	-1.116705264	1.55E-07
IGFBP3	-1.117052115	2.30E-20
ADSS1	-1.117215825	2.09E-12
MMP13	-1.117689552	1.09E-05
ARHGAP33	-1.117813608	2.01E-13
POLD4	-1.117921774	8.87E-44
BPIFB1	-1.118017822	1.84E-06
A4GALT	-1.118852132	2.23E-29
DAPK2	-1.118904049	9.39E-25
BMERB1	-1.119176274	1.66E-25
CLSTN3	-1.119469854	5.55E-13
TP53I3	-1.120396425	4.54E-17
ZNF185	-1.121160031	4.62E-82
AMOTL2	-1.122182606	1.57E-105
EPHA4	-1.123321372	7.16E-30
HDAC11	-1.124756319	2.01E-23
RUNDC3A-AS1	-1.125485399	5.66E-14
DAB2	-1.127362071	8.66E-10
TFF1	-1.127981309	2.68E-40
PCDHAC2	-1.129554283	9.98E-07
LNCAROD	-1.131547425	1.57E-06
ATP2B4	-1.133497272	1.06E-05
RRAS	-1.133798334	1.54E-17
TMPRSS3	-1.134269654	0.000228669
FUT8-AS1	-1.136156472	7.19E-05
AC245100.8	-1.136716375	0.001653983
HELZ2	-1.137835462	6.93E-40
AC007686.3	-1.138367015	0.000214132
CLDN9	-1.138739244	2.41E-19
KNDC1	-1.139073174	0.000377972

SLC22A18AS	-1.140970184	0.000590715
GTF2IRD2	-1.141137357	2.32E-10
MB	-1.141632092	8.59E-18
ALS2CL	-1.14221102	3.41E-10
CBFA2T3	-1.142291588	1.19E-54
LINC02620	-1.142803474	0.000886944
LINC02321	-1.14325966	0.001046742
IFI6	-1.144591553	3.72E-12
AL354740.1	-1.145282309	7.72E-05
LHX1-DT	-1.145412829	0.001371494
ZMYND15	-1.146305308	0.000205958
TENT5B	-1.148010549	0.00016398
TGFBI	-1.148163338	3.67E-26
ARHGDIB	-1.152276075	0.001362276
KLF6	-1.152601846	2.08E-34
PLXNB3	-1.154272766	8.25E-15
LOXL1-AS1	-1.154857776	2.20E-14
SYNGR3	-1.154870374	1.76E-16
TUBB3	-1.156481256	9.00E-128
PAQR7	-1.157155538	2.56E-10
ADAMTS13	-1.158349712	3.20E-11
RAB4B	-1.158826759	2.25E-12
SYNPO	-1.159445211	1.73E-10
PPL	-1.159584266	4.56E-152
TINCR	-1.160439267	3.99E-48
ALDH1A3	-1.161505086	2.97E-41
SLC25A42	-1.162048029	8.73E-18
LIF	-1.162341752	3.45E-32
IFITM10	-1.162589906	8.69E-05
COL6A2	-1.163338645	1.31E-07
LOXL2	-1.164750803	4.52E-206
GSTM4	-1.166410426	7.97E-64
PRRT2	-1.168815169	2.44E-09
DYRK1B	-1.16892641	6.73E-30
NRCAM	-1.169153083	1.72E-84
OAS1	-1.169184877	0.000151445
TP53INP1	-1.17004497	1.24E-20
INHA	-1.170806474	2.95E-07
MAB21L4	-1.171804723	4.15E-15
C1QTNF6	-1.174902553	1.14E-207
SCN8A	-1.175293595	1.83E-06
CNIH2	-1.176451006	0.000244575
APOL6	-1.18045466	3.65E-12
ATP2A3	-1.181330716	3.41E-185
PLTP	-1.181401686	0.000134939
EFNA2	-1.182325332	0.001086694
CLCF1	-1.182708607	2.47E-14

	LMO7	-1.182952048	4.00E-94
	CORO1A	-1.183058925	1.80E-20
	PTPRM	-1.183418495	1.70E-09
	RAB26	-1.184020958	5.31E-13
	AC244197.3	-1.184628628	8.44E-09
	MDGA2	-1.18594841	6.04E-06
	EBF4	-1.187871085	0.001594397
	FAM66B	-1.188571475	0.002270602
	PAPSS2	-1.189129372	1.81E-164
	MAOA	-1.18985766	1.99E-08
	TMEM191B	-1.189950384	0.000186131
	KRT87P	-1.190649205	5.72E-08
	F3	-1.191382335	4.26E-11
	RAI2	-1.191561398	0.00033987
	SCX	-1.1915868	2.81E-10
	KRT15	-1.192428149	1.26E-16
	ERP27	-1.19366595	1.92E-13
	ZFHX2	-1.197645495	0.000221669
	ITGA2	-1.198047586	5.28E-99
	SYTL4	-1.202419475	1.23E-10
	COL5A2	-1.202875777	3.23E-13
	SCN1B	-1.203219198	1.56E-13
	TCP11L2	-1.203364012	8.62E-08
	LRRC6	-1.208594181	0.00055307
	ULK1	-1.2087994	5.67E-108
	TMSB4X	-1.209549267	5.91E-272
	VPS9D1	-1.210357077	1.02E-19
	PLA2G10	-1.210647232	1.16E-05
	GREB1	-1.211643543	0.001178956
	CAMK2N1	-1.212045336	1.65E-119
	SPNS2	-1.213480546	2.19E-50
	ARID5B	-1.215232939	9.66E-36
	CYP4F11	-1.216000128	5.31E-05
	MUC20	-1.216278204	5.96E-30
	TESK2	-1.216434691	0.000244845
	ESPN	-1.223213444	1.50E-16
	MAFF	-1.223390853	1.47E-15
	EFEMP1	-1.224492524	2.91E-22
	LMNTD2-AS1	-1.225819951	1.85E-05
	KCNN4	-1.226116177	2.29E-19
	LINC02732	-1.226226381	3.82E-45
	CAPN13	-1.226797221	0.000293675
	PSMG3-AS1	-1.227599554	6.84E-48
	SCARA3	-1.228536995	7.24E-32
	GNG7	-1.228766477	4.27E-09
	SELENBP1	-1.229216944	1.14E-13
	TMEM8B	-1.230497333	2.26E-06

USH1G	-1.231961006	2.87E-16
FN1	-1.232068069	1.32E-85
DIRC3	-1.232098441	1.40E-05
CMPK2	-1.232320971	0.000188215
AC008014.1	-1.233129676	0.000244523
CYP26B1	-1.233326177	4.14E-06
INPP4B	-1.235226641	3.19E-123
ZNF879	-1.235476399	0.000163986
PAPLN	-1.235701532	4.08E-06
DOK7	-1.235748888	1.14E-26
PGM5	-1.236230612	2.76E-08
LGALS1	-1.23670446	1.60E-123
SLC4A3	-1.237185415	1.40E-08
ABTB1	-1.238083588	1.35E-20
ACOX2	-1.238114182	0.00099289
COL6A1	-1.238946471	0.000295417
CSRP2	-1.239485339	1.80E-07
ZCCHC12	-1.241125743	0.000302882
ARHGEF37	-1.241886918	8.60E-05
SERPINA3	-1.242900027	6.05E-08
FGD5	-1.24296849	2.10E-05
ITGB4	-1.243242925	3.62E-173
STEAP4	-1.243879339	7.67E-17
PCOLCE	-1.247157825	3.23E-17
RND1	-1.248622478	1.33E-31
FSCN2	-1.251065035	2.91E-05
LTBP2	-1.25358502	1.34E-08
SOWAHB	-1.254855495	3.34E-13
RPS6KA2	-1.25488776	1.48E-13
HLA-DQB1	-1.268108152	1.89E-51
KIAA1210	-1.269377895	1.89E-09
BDKRB2	-1.270685233	1.99E-78
NES	-1.271682657	5.04E-09
SPINK1	-1.272219417	0.00024913
DDAH2	-1.272713792	1.42E-67
TNFAIP2	-1.273044085	2.17E-05
TGFB2	-1.273144687	1.10E-23
TMEM40	-1.273632271	6.53E-13
JAKMIP2	-1.277548236	2.36E-17
SCIN	-1.280479957	0.000174495
RUNX2	-1.281201113	0.000100825
ZSWIM4	-1.281490543	9.53E-14
PRICKLE2	-1.281959234	2.48E-05
ZNF682	-1.284734377	1.15E-05
AC144450.1	-1.284934041	4.89E-08
GPRC5A	-1.285935651	3.33E-254
EDN1	-1.286142769	2.46E-07

THSD4	-1.286217829	5.85E-49
LYPD3	-1.286621798	5.44E-52
MAGED4B	-1.290896982	2.27E-05
PBXIP1	-1.292156122	9.25E-66
TSPAN1	-1.292808834	1.83E-20
ENTPD2	-1.293063442	6.91E-10
COL27A1	-1.295397157	4.50E-10
RAP1GAP	-1.295404397	4.67E-50
WNT9A	-1.295768095	2.62E-06
CLIC3	-1.296480364	7.75E-94
TMEM139	-1.296560771	1.18E-07
MYPN	-1.296923547	1.16E-21
FOS	-1.29773589	7.41E-62
SYTL2	-1.298986287	1.15E-261
CAPN8	-1.301302545	5.67E-10
IFIT1	-1.301840294	0.000255094
AC022034.1	-1.303696009	2.14E-10
ADCY5	-1.304217446	8.85E-69
NEURL1B	-1.304972937	3.21E-37
ADAMTSL5	-1.305364504	8.39E-19
ADORA1	-1.305372026	2.43E-18
MAP2	-1.308592854	3.13E-11
PLA2G4F	-1.309505992	1.03E-08
TUBA1A	-1.310649009	7.43E-80
KLK6	-1.311127898	3.64E-06
FGD3	-1.312177135	4.79E-113
HMCN1	-1.312330509	1.37E-64
PRSS23	-1.313201631	3.20E-23
SLFN5	-1.314853334	2.37E-22
IRF9	-1.315186254	1.36E-24
INHBA	-1.317157558	2.12E-35
AC068580.3	-1.319559309	3.53E-05
MVP	-1.319981203	6.76E-61
DIO2	-1.323652313	5.84E-20
RET	-1.324536253	1.79E-101
IRF7	-1.325569736	3.28E-13
MST1R	-1.325986427	6.49E-11
FER1L4	-1.334590057	8.75E-11
NOXA1	-1.334796326	2.49E-17
VSIR	-1.334909908	5.35E-06
AL590822.3	-1.334998619	0.000320004
C19orf33	-1.33520091	1.20E-43
UGT1A6	-1.338319696	1.01E-07
CASKIN1	-1.339402731	1.77E-06
MARCKS	-1.339921114	4.39E-36
EGR3	-1.341480702	4.63E-18
COL9A2	-1.342789048	2.30E-13

	CDKN2B	-1.343686281	1.46E-72
	MUC1	-1.345758933	8.31E-40
	SYTL5	-1.350291555	1.86E-41
	CDK14	-1.357562031	2.23E-06
	TNS1	-1.35790573	1.91E-08
	DUSP6	-1.358035329	2.63E-05
	ABCA7	-1.358148943	5.28E-14
	MICAL1	-1.35996139	6.86E-31
	MAPK4	-1.361158091	8.52E-16
	RAB19	-1.363481906	9.29E-08
	LAMB3	-1.367023235	5.70E-09
	MAFB	-1.372611802	9.86E-07
	KLF7	-1.3736537	6.88E-08
	CHRD	-1.373995516	5.91E-27
	TRIM29	-1.377174764	1.22E-09
	FAM155A	-1.380297972	2.01E-13
	DUSP4	-1.380629583	6.85E-290
	CTSO	-1.382003281	4.23E-08
	SPOCK1	-1.388083418	4.38E-29
	SPEG	-1.388687134	5.84E-10
	TLCD2	-1.390387073	1.45E-15
	DDX60L	-1.391890213	1.15E-05
	MYEOV	-1.394012161	1.75E-82
	LOXL1	-1.397738638	1.29E-08
	C17orf82	-1.398489583	9.44E-10
	AC040970.1	-1.399256869	2.94E-05
	SUSD2	-1.400785488	6.05E-10
	CRAT	-1.400798552	1.29E-06
	MT2A	-1.402445398	9.11E-87
	NTN4	-1.403617276	1.46E-76
	CCNG2	-1.407877726	5.71E-64
	SAMD9	-1.409063656	3.00E-20
	MST1	-1.413859018	1.45E-06
	ALPK3	-1.415773178	1.95E-26
	ST6GALNAC2	-1.416327617	8.53E-45
	TNFSF15	-1.416393118	1.68E-07
	PXDC1	-1.417173771	3.12E-08
	MEIS3	-1.420736597	1.73E-22
	SLC16A2	-1.421672413	2.28E-07
	ABAT	-1.423057129	6.39E-142
	CHST3	-1.428218828	5.43E-05
	UBA7	-1.432293805	1.37E-09
	MEGF6	-1.433583174	3.49E-52
	ZNF175	-1.442778373	1.29E-20
	COL12A1	-1.442855125	6.68E-33
	MALL	-1.446178391	3.06E-117
	JPH2	-1.446641549	4.36E-34

	MATN3	-1.447166084	6.98E-18
	ABCC3	-1.450901433	1.58E-178
	DNAJB5	-1.453978134	3.01E-08
	MAF	-1.458302643	5.06E-05
	APCDD1	-1.460501011	8.91E-15
	GDF15	-1.464876897	1.58E-75
	FAT4	-1.465673799	1.10E-14
	ITGB6	-1.467889365	5.97E-140
	SH3PXD2A	-1.468364697	2.68E-09
	MRPL23-AS1	-1.480406366	5.76E-07
	ADGRF4	-1.487420897	2.91E-16
	MME	-1.48850875	3.48E-09
	DDX60	-1.490744133	4.55E-12
	RIPOR3	-1.490891291	8.48E-13
	SEMA3B	-1.49173959	3.69E-91
	POC1B-AS1	-1.492414893	5.58E-06
	TCIM	-1.492600006	1.85E-36
	PRR15	-1.492764744	2.19E-22
	LINC02600	-1.496983671	6.17E-07
	SEMA5B	-1.497024672	8.64E-22
	AC004264.1	-1.499308099	6.66E-07
	LAMC2	-1.50082616	8.70E-36
	CALML5	-1.513047724	3.95E-17
	GLRA3	-1.516461242	2.39E-09
	AL157935.2	-1.536431337	1.59E-06
	AC005821.1	-1.537652446	6.69E-17
	LHFPL6	-1.543287813	1.59E-23
	CCDC83	-1.545669225	4.51E-06
	PRR36	-1.549457348	2.20E-10
	BANK1	-1.551582741	1.69E-06
	PSG9	-1.559966042	2.11E-26
	CAPN9	-1.563987155	3.74E-10
	AC015802.6	-1.581077462	2.39E-08
	RASGRP1	-1.584197273	3.16E-28
	NT5E	-1.59828565	3.45E-119
	TMPRSS4	-1.599985514	3.01E-08
	LRRC15	-1.602236333	2.28E-19
	CYSRT1	-1.609924108	1.27E-38
	CCN2	-1.615919441	8.32E-13
	BHLHE41	-1.617215461	5.85E-11
	AL590004.3	-1.62313914	4.52E-33
	AC110619.1	-1.626839393	9.56E-73
	ABCA4	-1.630931089	8.63E-58
	MIR503HG	-1.654378633	6.94E-20
	AQP3	-1.656546033	8.59E-146
	AC006372.2	-1.658803087	1.50E-19
	MGP	-1.660559392	3.49E-09

	CEMIP	-1.665680398	4.32E-116
	SSPO	-1.669198224	3.91E-11
	EDIL3	-1.679434401	1.35E-23
	AFAP1L2	-1.683489524	1.69E-13
	AC010735.2	-1.686556716	2.13E-08
	AC006372.1	-1.687781372	1.35E-08
	PLXNA2	-1.699029804	7.03E-29
	MYO15B	-1.724971113	4.41E-07
	PHLDB2	-1.726783792	2.86E-12
	NEDD9	-1.731911662	8.86E-24
	ACHE	-1.733207809	2.32E-13
	SLC4A4	-1.781333333	7.34E-10
	SLCO2A1	-1.7841157	1.21E-22
	KRT16	-1.79773724	8.77E-08
	LINC01213	-1.807804024	2.09E-07
	RNF224	-1.810867937	2.60E-14
	SNAI2	-1.815639806	1.47E-13
	COL5A1	-1.833345824	2.37E-122
	TNFRSF11B	-1.839125962	7.19E-16
	VGLL1	-1.888013739	3.00E-12
	LINC00365	-1.892287133	6.44E-12
	LINC02747	-1.919900894	3.69E-27
	ALPP	-1.942288035	1.20E-14
	PSCA	-1.991706289	1.47E-25
	SERPINE1	-2.456583165	4.30E-17

Table S9: 2309 mRNAs with genotype-dependent differences in c-MYC-induced regulation

Gene symbol	padj	Gene symbol	padj	Gene symbol	padj
DEGS1	3.06E-43	JDP2	0.002193	DCLRE1A	0.018639
KRT81	1.64E-39	MBOAT2	0.002209	RPL38	0.018639
LIPA	3.64E-38	SLC39A11	0.002209	ZMYND8	0.018639
F2RL1	3.39E-32	DEPDC1	0.002211	GPD2	0.018648
CDKN1A	1.23E-31	EIF3E	0.002211	GDF11	0.018648
FDXR	2.37E-27	UBA52	0.002218	THNSL1	0.018761
CYFIP2	2.44E-25	ADAR	0.002234	TMEM11	0.018934
TAP1	4.64E-25	PRMT1	0.002264	EEF1AKNMT	0.018968
IRF2BP2	6.12E-24	ZWINT	0.002265	SLC22A5	0.01903
ITGB5	1.92E-22	NT5C2	0.002269	PRKCSH	0.01903
SESN1	8.24E-21	ZDHHC9	0.002281	SLC39A10	0.019236
COTL1	3.00E-20	TYSND1	0.002284	ANKRD13B	0.019236
ALDH3B2	9.73E-20	SLC25A39	0.002284	HM13	0.019236
HS3ST3B1	2.32E-19	FKBP1A	0.002284	FUT4	0.01924
CTNNAL1	7.48E-19	KHDC1	0.002296	FANCD2	0.019353
TM7SF3	1.62E-18	SPAG5	0.002297	NUDT21	0.019365
STC2	2.89E-17	PINK1	0.0023	DIP2C	0.019434
GRIN2C	1.02E-16	IFIT1	0.0023	GTF2IRD2	0.019438
PFKP	1.92E-16	KIF18A	0.00232	HOXC13	0.019438
CEBPB	4.97E-16	ZNF496	0.002348	MAL2	0.019452
SHB	2.73E-14	USP37	0.002354	NOL7	0.019481
ID3	7.97E-14	STOML2	0.002354	SLC66A1	0.019497
MET	8.20E-14	SLBP	0.002412	EGFR	0.019555
HPCAL1	9.05E-14	SLC6A6	0.002418	SOWAHC	0.019593
PIK3R3	2.35E-13	EIF4A2	0.002424	ABLIM1	0.019593
ZMAT3	2.35E-13	MPHOSPH6	0.002436	P2RY2	0.019642
AC093001,1	3.11E-13	SRSF4	0.002477	NGRN	0.019642
PHLDA3	5.64E-13	KNL1	0.002477	FAM225A	0.019693
ITGB1	8.27E-13	MOCOS	0.002506	B4GALT3	0.019758
ADORA2B	1.64E-12	RPLP1	0.002506	TARS1	0.019758
CD59	2.56E-12	NRIP1	0.002506	MTBP	0.019758
GRHL3	4.75E-12	UNC93B1	0.002506	EHBP1L1	0.019758
TPD52	6.46E-12	C2CD5	0.002506	LSM10	0.019776
POLH	8.71E-12	GNA11	0.002508	MRPS18B	0.019776
HPS3	8.74E-12	HMGCS1	0.002509	APTR	0.019776
PGRMC1	1.24E-11	LRPAP1	0.002518	TMC6	0.019776
FAM102B	2.61E-11	RPS3A	0.002518	EIF3K	0.019776
KYNU	3.88E-11	IL10RB-DT	0.002518	PLCG1	0.019776
EIF4EBP1	1.61E-10	DGCR8	0.002533	DAP3	0.019846
ABHD17C	1.83E-10	DPP7	0.002537	ASAP2	0.019846
SLC35C1	1.87E-10	DDX58	0.002548	PUF60	0.019846
ADAMTS19	2.35E-10	MFSD1	0.002566	NEDD4	0.019846
LTBP3	3.18E-10	ZBTB2	0.002566	TMED3	0.019846

GLMP	3.78E-10	APOBEC3B	0.002566	HMGB3	0.019846
TP53INP1	4.26E-10	VASP	0.002582	PTPN18	0.019868
ASS1	4.28E-10	GRB2	0.002619	RBL2	0.019868
DBN1	5.89E-10	PPP6R1	0.002631	FBXW9	0.019876
NUSAP1	6.47E-10	CCDC90B	0.002638	FIRRE	0.019903
ITPR1	8.38E-10	C5	0.002645	SLC9A2	0.01995
ADIPOR2	8.38E-10	RPL37	0.002651	TET2	0.01995
MOSPD1	1.05E-09	RPS27	0.002654	CBR1	0.01995
OAS2	1.36E-09	SULT2B1	0.002656	DCUN1D5	0.019975
SYT7	1.59E-09	EML1	0.002659	THOC1	0.020034
UACA	2.00E-09	UBE2C	0.002689	FNBP1L	0.020044
ETS2	2.13E-09	MIS18BP1	0.002713	TMEM165	0.020052
CHFR	5.07E-09	NDUFB8	0.002713	MRGBP	0.020052
FAM118A	5.20E-09	TMEM170B	0.002715	ARFGAP1	0.020071
LDHA	5.98E-09	CENPU	0.002717	NOP10	0.020109
TACC1	7.70E-09	TIMP2	0.002731	ZNF771	0.020109
PPM1D	7.70E-09	PARP9	0.002748	PIGP	0.02011
MALAT1	1.46E-08	USP1	0.002762	RPL22L1	0.020158
YPEL2	2.25E-08	FAM83D	0.002762	IL6R	0.020228
MRPL34	2.25E-08	CEP55	0.002779	CDKL5	0.020361
RHPN2	2.54E-08	CNPY2	0.002783	GOLGA7	0.020451
DNAJC1	2.56E-08	TCAF1	0.002804	CHID1	0.020451
ASPH	2.56E-08	WDR76	0.002805	TRIM25	0.020451
PHLDB1	2.59E-08	ZC3HAV1	0.002837	NPTXR	0.020451
HSPA5	2.89E-08	RAB11FIP1	0.002858	IMPDH1	0.020466
GTF3A	2.89E-08	INPP4B	0.002866	FTSJ1	0.020469
RPS27L	2.94E-08	CRY2	0.002866	TOM1	0.020494
OAS3	2.95E-08	RFX5	0.002897	FIBCD1	0.020622
TNFAIP3	3.63E-08	EXOSC5	0.002897	TSPAN3	0.02066
PSAP	3.99E-08	RPS18	0.002898	ZYX	0.020667
ACER2	4.08E-08	ZNF367	0.002898	RSL1D1	0.020689
NAGK	4.52E-08	SNHG7	0.002898	FIGN	0.020835
LIMK2	4.52E-08	MCM6	0.002915	MRPL12	0.020914
PYGB	4.82E-08	SEC16A	0.002919	PCM1	0.020975
RAP2B	6.00E-08	UBN1	0.002934	SYK	0.021063
TNFSF15	6.15E-08	ATP6V0B	0.002943	MTFR1L	0.021165
PKM	6.91E-08	EIF3I	0.002955	RAP1GDS1	0.021205
RAB27B	7.64E-08	TMTC2	0.002969	CBLB	0.021231
EGR1	9.50E-08	RELB	0.003011	APRT	0.021231
APBB2	1.01E-07	RPL34	0.003037	POLA1	0.021231
PGGHG	1.17E-07	GRSF1	0.003054	MTCO1P12	0.021235
CHAC1	1.19E-07	WDR77	0.003058	DHPS	0.021295
ADGRG6	1.19E-07	TPM2	0.003058	ATF3	0.021334
BTG2	1.20E-07	POLD3	0.003068	SOX4	0.021334
TIGAR	1.20E-07	ZNF443	0.003068	SEMA4D	0.021334
VPS18	1.28E-07	TMEM184A	0.003073	SLF2	0.021334
LPCAT4	1.60E-07	PPM1J	0.003086	FZD4	0.021397

SLC45A3	1.63E-07	PEPD	0.003087	PAK1IP1	0.021402
ISCU	2.27E-07	INCENP	0.003093	SLX4IP	0.021402
IRX2	3.24E-07	VAPB	0.003108	YDJC	0.021405
HELZ2	3.30E-07	BCL9	0.003112	NDUFB2	0.021426
JADE1	3.36E-07	TTC7A	0.003112	POGZ	0.02144
WDFY1	3.44E-07	FAS	0.003112	CA5BP1	0.02144
GDF15	3.46E-07	UBR7	0.003112	DIO2	0.021461
IKZF2	4.22E-07	MAP11	0.003123	PHPT1	0.021464
ANP32A	4.22E-07	MAPKAPK5-AS1	0.003164	ITGA2	0.021474
CYB5A	4.46E-07	PDXP	0.003164	INTS6L	0.02149
ERO1A	4.61E-07	NDC80	0.003176	HNRNPDL	0.021518
ITGA6	5.09E-07	NFKBIZ	0.003183	CBS	0.021574
KIF20A	5.81E-07	SNHG29	0.003189	CHMP2B	0.021608
STIL	6.01E-07	IFIH1	0.00321	MCUR1	0.021608
KCNG1	6.95E-07	KLHL5	0.003212	AUP1	0.021677
ZFP36L2	7.00E-07	MIS12	0.003223	FBXO5	0.021677
NCAM2	7.01E-07	IL27RA	0.00329	RAB15	0.021677
ENTR1	7.93E-07	TRNP1	0.003296	STK38	0.021695
DVL1	9.08E-07	CCDC88C	0.003315	PCGF6	0.021983
BAG1	9.08E-07	TIMM23	0.003321	ADCK2	0.02212
AEN	9.08E-07	WDR45	0.003332	IMPAD1	0.022169
BACE2	9.08E-07	PPIC	0.003336	OSBPL6	0.022249
PLCD3	9.75E-07	CNIH1	0.003363	SMIM26	0.022258
FHDC1	1.12E-06	POLR3K	0.003396	BRMS1	0.022502
STAT6	1.12E-06	AHSA1	0.003398	ZNF580	0.022533
RPS6KL1	1.12E-06	ERBB3	0.0034	EMC6	0.022536
PRKCA	1.29E-06	PLPPR2	0.0034	SULF2	0.02257
NBPF4	1.37E-06	SDF2L1	0.0034	SELENOS	0.022626
CENPF	1.38E-06	KLF9	0.003409	PTRH2	0.02264
BDH1	1.38E-06	CMTM8	0.003426	IQGAP1	0.022653
CCNA2	1.38E-06	FYN	0.003443	TMEM222	0.022716
EGLN3	1.43E-06	COPZ1	0.003451	TTYH3	0.022716
LACTB	1.43E-06	RAMAC	0.00348	SPDEF	0.022762
APOL6	1.43E-06	RPL17	0.00353	TTC4	0.022862
BRIP1	1.50E-06	ARFIP2	0.003533	HEATR6	0.022862
PPP1R2	1.62E-06	PSMB10	0.003533	PLK4	0.022944
RHOBTB2	1.62E-06	CDC6	0.003533	CMSS1	0.022991
SPTSSA	1.62E-06	NFATC2	0.003533	HSPA1B	0.02305
ATP6V0A4	1.75E-06	CEBPZOS	0.003541	CHAC2	0.023094
RPL15	1.77E-06	TNFRSF21	0.003556	PFDN6	0.023094
OAS1	1.89E-06	WARS2-AS1	0.003559	SYNJ2BP	0.023094
SPHK1	1.89E-06	ETF1	0.003568	PDPR	0.023094
CCNG1	1.92E-06	NEURL1B	0.003573	IP6K2	0.02311
BZW1	2.05E-06	MFSD3	0.003573	FEM1B	0.02311
FAM50A	2.11E-06	NAT9	0.003573	ANXA9	0.023154
TYRO3	2.18E-06	AURKA	0.003573	CEP97	0.023249

ARNT2	2.38E-06	CNOT7	0.003577	PDLIM7	0.023249
TPX2	2.95E-06	INKA2	0.003586	RNF125	0.023249
EEF1A2	2.95E-06	SPINT2	0.003612	PKMYT1	0.023429
DLG3	2.96E-06	TCF19	0.003661	OSTC	0.023441
USP2	3.21E-06	NRF1	0.003661	PSRC1	0.023527
MBNL2	3.26E-06	CHAF1B	0.003683	TPRN	0.023527
ZNF385A	3.32E-06	ARHGEF18	0.003709	SRSF6	0.023584
IQGAP3	3.72E-06	DNAJC11	0.00371	VGLL1	0.023584
CCDC78	3.72E-06	TSPAN14	0.00371	FTL	0.023597
ENO1	4.12E-06	PHLDA2	0.003721	SNHG10	0.023633
NEK6	4.22E-06	FNDC10	0.003731	NAA10	0.023633
ENTPD6	4.22E-06	NRCAM	0.003731	ATIC	0.023646
DOCK11	4.35E-06	BAX	0.003735	MARK3	0.023729
HECTD3	4.95E-06	RPS14	0.003845	MANF	0.023784
CUTA	5.26E-06	SNHG26	0.003845	PDCD10	0.023784
CPEB4	5.30E-06	UBXN8	0.003849	KCTD1	0.023795
FERMT1	5.30E-06	SHCBP1	0.003849	PLEKHS1	0.023807
FAM78A	5.94E-06	AGAP3	0.003854	NADSYN1	0.023813
FAHD1	6.27E-06	TMPO	0.00387	SOWAHB	0.023836
ARL6IP5	6.35E-06	CDC20	0.003873	LYRM4	0.023836
AC021087,5	6.41E-06	ATP5MD	0.003881	PPT2	0.023836
IGDCC3	6.52E-06	CLN3	0.003881	FAM241A	0.023876
SLC12A9	6.86E-06	NLGN2	0.003881	ARHGDIA	0.023876
ECE1	7.33E-06	LMO7	0.003971	DIAPH3	0.023911
BMP7	7.46E-06	PLXNB3	0.003977	NDUFAB1	0.023911
SLC3A2	7.69E-06	CYB561	0.003995	UCHL5	0.023944
NCOA2	7.81E-06	SDF4	0.004006	ISY1-RAB43	0.024011
ZNF217	8.33E-06	KCNN4	0.004006	TNFRSF11B	0.024011
ENTPD1	8.39E-06	COPS7B	0.004009	EFCAB11	0.024139
TSPYL2	8.39E-06	RPS3	0.004033	MTMR12	0.024241
HTATIP2	8.43E-06	EHD4	0.004033	MBTPS1	0.024241
YIPF2	8.43E-06	ARID5A	0.004037	TRAPPC6B	0.024304
TRIM16L	8.67E-06	RPL8	0.004143	ATP6V1F	0.024319
PRDX4	8.67E-06	CD99L2	0.004143	BSG	0.024419
AP002761,4	8.97E-06	KIF14	0.004185	FUT9	0.02447
ATP1A1	9.18E-06	TACC3	0.004188	SRD5A3	0.024526
PHACTR2	9.46E-06	BRCA2	0.004188	PDCD4	0.024526
ULK1	1.01E-05	LYPLA2	0.004203	MXRA7	0.024526
XPNPEP1	1.03E-05	PRCC	0.004228	KLHL2	0.024569
PODXL	1.06E-05	VLDLR	0.004228	RPS21	0.024569
PRNP	1.06E-05	CYC1	0.004258	TIA1	0.024603
GNPDA1	1.12E-05	PARVA	0.004276	RNF113A	0.024649
TMEM63B	1.12E-05	MPZL2	0.004282	SPC25	0.024716
ADGRE5	1.12E-05	VKORC1	0.004308	AP1S1	0.024797
PC	1.20E-05	TFAP2A	0.004316	SOD2_1	0.024812
MRPL36	1.26E-05	RIMS4	0.00433	AC021078,1	0.024884
RACK1	1.28E-05	EI24	0.004341	CLUHP3	0.024922

EPHA2	1.45E-05	KIF15	0.004395	H2BC21	0.024948
MEGF9	1.49E-05	SLC30A1	0.004463	TINCR	0.024948
CHEK1	1.49E-05	LGALS1	0.004463	FOXP4	0.024975
TUBG2	1.53E-05	ANKH	0.004483	POLR1D	0.024981
TAF13	1.58E-05	PHB	0.004493	S100A16	0.025143
NVL	1.61E-05	CYP2R1	0.004501	KDELR2	0.025143
MYH14	1.64E-05	RPL18	0.004518	NDUFB7	0.025143
MACC1	1.68E-05	STK38L	0.004524	DCP1B	0.02515
SLC7A5	1.84E-05	TLCD4	0.004551	RSRC2	0.025171
IRF9	1.88E-05	NCAPG2	0.004554	ITPKC	0.025171
DDB2	1.89E-05	SLC12A7	0.004576	RNASEH2C	0.025191
RPL6	1.93E-05	SLC17A5	0.00458	HSD17B7	0.025319
SKA3	1.96E-05	STRAP	0.004611	RUVBL2	0.025319
IGSF8	2.01E-05	CIP2A	0.004623	MPV17	0.025374
YPEL5	2.02E-05	TATDN1	0.004623	TACO1	0.025375
LDLR	2.02E-05	LMNB2	0.004623	RPS16	0.025375
CDC42EP1	2.02E-05	CDCA2	0.004651	C8orf76	0.025398
UNG	2.05E-05	AQP3	0.004652	MINDY2	0.02555
SKIL	2.20E-05	CENPA	0.004657	MAPK4	0.02555
ZMYND19	2.31E-05	PPP2CA	0.004657	ADAM17	0.025666
ZMIZ1	2.33E-05	C1orf198	0.004722	ATG101	0.025793
IKBIP	2.34E-05	ZP3	0.004722	ZNF410	0.025793
STON1	2.36E-05	PLEKHA2	0.004722	HDDC3	0.025824
SHISA9	2.43E-05	CBX1	0.004722	CLU	0.025855
TPI1	2.44E-05	GNG5	0.004729	ESCO2	0.025888
SYTL1	2.46E-05	MGST3	0.004729	NGDN	0.025888
MTFP1	2.46E-05	GEN1	0.004735	TMED10	0.025888
DANCR	2.60E-05	TMEM104	0.004745	ARHGAP18	0.02594
ID2	2.64E-05	ILKAP	0.004768	MCCC2	0.025951
KLF10	2.71E-05	TRIM38	0.004768	IDH3A	0.025968
SEC14L1	2.71E-05	GLRX5	0.004777	TMEM63C	0.026072
EPHA4	2.81E-05	MFSD12	0.004777	FAM168B	0.026199
STX3	2.85E-05	RTCB	0.004794	MBD3	0.026267
KLC2	3.01E-05	SP110	0.004823	CAPZA2	0.026373
DENND2C	3.01E-05	GAB1	0.00483	NIFK	0.026441
MCM2	3.01E-05	ASB6	0.00483	FKBP5	0.026542
MCMBP	3.14E-05	PLD1	0.004833	RNF34	0.026542
RNF126	3.22E-05	EBPL	0.004893	NOD1	0.026677
MLST8	3.26E-05	TIMMDC1	0.004894	ZNF639	0.02676
GALK1	3.43E-05	RPL30	0.004894	FAM72B	0.02678
KIAA2013	3.44E-05	EDIL3	0.005018	SCAMP3	0.02678
PLOD2	3.74E-05	BRD1	0.005018	POLR3H	0.02678
ILF3-DT	3.83E-05	COPS7A	0.005031	PDHA1	0.026827
PGK1	3.99E-05	PDE8A	0.005031	KLHL25	0.026862
PSEN2	4.11E-05	PTOV1	0.005031	SH2D5	0.027053
COL5A1	4.11E-05	WDR92	0.005072	SLC25A20	0.027053
DMPK	4.18E-05	PCBP2	0.005075	PSMG1	0.027053

COA4	4.41E-05	CENPI	0.005084	FANCI	0.027082
PLP2	4.41E-05	SGTA	0.005095	TGOLN2	0.027119
ELL2	4.46E-05	RPN1	0.005107	FAM53B	0.02715
KIAA1217	4.48E-05	AKAP9	0.00511	WDR46	0.027386
ANGPTL4	4.54E-05	LIN7A	0.00511	PCLO	0.027399
MIF	4.56E-05	MLYCD	0.005125	PPFIA3	0.02758
BUB3	4.59E-05	TMEM250	0.005138	POLR3C	0.027606
RAC3	4.60E-05	RER1	0.005182	EIF2S2	0.027661
FAM174C	4.76E-05	EEF1A1	0.005184	GCSH	0.027692
PPP1R26	4.79E-05	GK	0.005184	CROCCP2	0.027759
PGPEP1	4.82E-05	NEDD9	0.005184	HDAC2	0.027798
SEPTIN8	4.96E-05	B4GALT2	0.005207	PKIG	0.02791
NME4	4.96E-05	ZNHIT1	0.005213	EIF2D	0.027922
SERINC5	5.12E-05	LIG1	0.005221	AXIN1	0.027925
CDCA3	5.17E-05	COA6	0.005235	MAP3K9	0.027959
CIT	5.18E-05	LSM7	0.005245	NEIL3	0.028027
FAM83B	5.20E-05	KRT87P	0.00525	SIMC1	0.028054
E2F7	5.20E-05	AC245060,4	0.005258	AC080112,2	0.028064
SYTL2	5.24E-05	DDIAS	0.00526	SNRPD2	0.028156
CDCA5	5.82E-05	MBIP	0.00526	FKBP10	0.028209
SNHG3	5.83E-05	IKBKE	0.005279	DXO	0.028281
MDC1	6.03E-05	SLC25A4	0.005279	TGIF1	0.028281
NPTN	6.03E-05	NUDT4	0.005279	ETFB	0.028281
RBL1	6.03E-05	SSU72	0.005316	TARBP2	0.028479
MIR34AHG	6.11E-05	C1orf43	0.005344	CDR2	0.028481
FAM3C2	6.39E-05	COQ10B	0.005344	HASPIN	0.028515
KIF23	6.48E-05	CLSPN	0.005347	USP7	0.028643
CYP1B1	6.72E-05	RPL14	0.005347	TRIR	0.028643
SGCE	6.83E-05	NCOA7	0.005347	YARS2	0.028648
FOXM1	6.83E-05	TMEM64	0.005347	MRPL33	0.028661
SLC25A29	6.83E-05	ZNF462	0.005347	UBE2D2	0.028661
RPS5	6.83E-05	REPS1	0.005352	ELF3	0.028685
STX1A	7.14E-05	RNF167	0.005352	RBBP8NL	0.028721
CDKN2B	7.38E-05	FAM120AOS	0.005367	SRPRB	0.028786
RAB17	7.46E-05	BOK	0.005368	PKD2	0.028786
ESPL1	7.46E-05	S100A6	0.005383	IRX4	0.028911
PDIA3	7.46E-05	CTU2	0.00539	ZNF84	0.028911
RPL23A	7.55E-05	CHD1L	0.005486	C17orf82	0.028989
CLPTM1L	7.55E-05	PDIA6	0.005494	AC012321,1	0.029057
HERC6	7.62E-05	EPG5	0.0055	FAM122C	0.029131
TMEM189	7.62E-05	COBLL1	0.005516	CALCOCO1	0.029147
ATF4	7.62E-05	ATP10D	0.005557	ZMYM4	0.029213
MID1	7.63E-05	CD151	0.005581	NOMO1	0.02926
DKK1	7.63E-05	RPS19	0.005581	TLE5	0.02926
IFRD1	7.90E-05	HERPUD1	0.005592	VAV2	0.029347
TOP2A	7.96E-05	SMPD2	0.005598	MACROD1	0.029347
AMZ1	8.33E-05	ZNF641	0.005635	KIF20B	0.029355

RPS7	8.57E-05	OSBPL9	0.005646	LGALS3	0.029385
TRIB3	8.57E-05	TMCC1	0.005646	CRKL	0.029385
NCBP2AS2	8.61E-05	PCOLCE2	0.005646	ZNF860	0.029539
MASTL	8.69E-05	HYOU1	0.005646	HAGH	0.029586
VDAC2	8.69E-05	PHKA1	0.005681	BAIAP2-DT	0.029586
DDA1	8.69E-05	C19orf33	0.005699	SPOCK1	0.029615
KIF11	8.75E-05	PRKCE	0.005748	POLR3F	0.029659
SDC3	8.79E-05	EPS8L2	0.005748	RFC5	0.02966
MDM2	8.79E-05	EIF2A	0.005751	CHMP4B	0.029696
STAMBPL1	8.92E-05	THSD4	0.005751	KLF6	0.029739
PARP12	8.95E-05	RPL37A	0.005754	MICOS13	0.029796
BHLHE41	9.02E-05	PPP5C	0.005754	PRPS1	0.029796
RPL4	9.09E-05	TRIM14	0.005759	ECT2	0.029858
C1QBP	9.09E-05	FAM111A	0.005759	CRAT	0.029873
STAT1	9.19E-05	NFATC1	0.005781	LRRCC1	0.029886
HSF1	9.19E-05	BMPR2	0.005781	NEMP2	0.030017
MAP2K1	9.19E-05	OSBPL10	0.005781	ATP2A2	0.030044
ATAD2	9.22E-05	FUNDC2	0.005781	MPP7	0.030061
POF1B	9.22E-05	AMOTL1	0.005844	VAPA	0.030062
ABLIM3	9.42E-05	KLF13	0.005918	TSPO	0.030091
ID1	9.42E-05	CTNNA1	0.005954	AL135905,2	0.030151
ELOVL6	9.46E-05	WIPI1	0.006039	HERC4	0.030158
ANLN	9.72E-05	CNTRL	0.00604	SEMA3C	0.030171
KIF18B	0.000102	BIRC5	0.00604	RPL3	0.030212
PPFIA1	0.000103	NUP50	0.00604	TM2D3	0.030228
GADD45A	0.000105	PTPRJ	0.006083	GLRX3	0.030268
HAGHL	0.000105	KIF2C	0.006159	ZNF75D	0.030417
POLR2E	0.000106	REEP5	0.006159	AL358472,6	0.030428
BRCA1	0.000108	PSAT1	0.006159	CSNK1A1	0.030428
PBX1	0.000109	LHX1	0.006159	NEK2	0.030585
SLC6A9	0.000111	TBL1X	0.006159	GALNT2	0.030585
SAMD9	0.000111	SLC30A3	0.006167	C4orf46	0.030585
MYORG	0.000111	IER5	0.006184	WASHC2A	0.030585
UGDH	0.000112	SNHG8	0.00622	LRP4	0.030585
RDX	0.000112	KIAA0895L	0.00622	SLC25A3	0.030585
ALG5	0.000115	UBE2L3	0.00622	PRSS22	0.030585
DNPH1	0.000117	CASP8	0.006223	RNF213	0.030585
GTSE1	0.000117	CMYA5	0.006223	TBC1D31	0.03064
SPINDOC	0.000117	FOXN3	0.006241	ST14	0.03064
PARP14	0.000121	CENPE	0.006259	RSPH1	0.03064
ORC1	0.000121	SIRT1	0.006264	RTL10	0.030652
HTT	0.000122	EPHB4	0.006269	RPS20	0.030716
GLTP	0.000122	POLR2J3_2	0.006269	CKAP2L	0.030728
NCAPH	0.000122	TGFB1	0.006269	RFC4	0.030728
LBHD1	0.000122	RFWD3	0.006293	ZNF430	0.030753
RPN2	0.000125	RPL5	0.006326	RNF38	0.0308
RPL27	0.000127	ZNF704	0.006326	RB1	0.03085

THAP11	0.000127	PRKCH	0.0064	KMT2A	0.030876
HIP1	0.000128	CHPF2	0.00642	OSBPL11	0.030945
ASPM	0.000129	RAD51	0.006432	YY1	0.030945
FLVCR2	0.00013	RTN4	0.006449	FUCA2	0.031007
TMEM150C	0.000138	RPLP2	0.006451	TMEM65	0.031007
RPUSD1	0.000138	ECHDC2	0.006459	MACROH2A2	0.031007
EIF3G	0.000138	CBX3	0.0065	MARS1	0.031007
RPL18A	0.000139	TRIM45	0.006579	RTL6	0.031007
UGT1A6	0.000143	MRPS16	0.006579	SLC39A8	0.031025
MIR22HG	0.000144	TSSC4	0.006579	SRSF2	0.031097
FARSA	0.000144	TMEM120B	0.006579	COX5B	0.031143
TNFRSF10B	0.000146	ZIC2	0.006579	ARFGEF3	0.031323
TTL	0.000149	ISG20	0.006579	MAP3K4	0.031346
DCLRE1B	0.00015	TMEM97	0.006579	SYNCRIP	0.031437
RPS4X	0.00015	FBXL3	0.006594	FBH1	0.031447
EFEMP1	0.000151	BLOC1S3	0.006603	ALOXE3	0.031505
JAGN1	0.000151	ALMS1	0.006634	LRRC8C	0.031514
PAPSS2	0.000151	RRM1	0.006637	NDUFB9	0.031551
BBC3	0.000153	ZNF318	0.006648	AL024508,1	0.03156
MCM4	0.000155	FAM3C	0.006649	MID2	0.03156
GLA	0.000155	RBMS1	0.006657	LRRFIP1	0.031758
MICAL2	0.000157	TMEM43	0.006657	EML2	0.031759
LIF	0.000158	TMEM147	0.006657	SLC12A5	0.031759
CD81	0.00016	SMC1A	0.006657	MALL	0.031767
RGS16	0.000161	BLVRA	0.006676	COPS6	0.031825
MRPL10	0.000165	CALR	0.006676	RAB32	0.032012
CKAP2	0.000173	GINS1	0.006676	EIF4E	0.032018
UBE2E3	0.000177	NEU1	0.006684	PIGQ	0.03207
BCL7B	0.00018	SSR4	0.006684	WAC-AS1	0.032078
DDX60	0.000182	DNAAF2	0.00675	PFDN5	0.032217
TOMM40	0.00019	ASCC3	0.00679	IFRD2	0.032219
MCRIP2	0.000191	OCIAD1	0.006799	B3GNT10	0.032234
NOMO2	0.000192	MRPL38	0.006802	ARID5B	0.032275
MXD4	0.000194	KIF24	0.006803	AC010186,2	0.032291
PLK1	0.000194	UPP1	0.006807	PIM3	0.032359
RABGGTB	0.000198	PITHD1	0.006818	KDM7A	0.032366
DPY19L1	0.000198	ZXDB	0.006818	SH3BP2	0.032472
ELOF1	0.000198	YBX1	0.006882	UNC5B	0.032492
FBXO22	0.000201	CREBBP	0.006882	PTGES2	0.03251
TLE1	0.000206	TTC38	0.006882	PELO	0.032515
TPRG1L	0.00021	R3HDM4	0.006914	CTDSP2	0.032515
PCCB	0.00021	PAM16	0.006946	CKAP4	0.032565
PPP2R2A	0.00021	BLOC1S2	0.006947	IVD	0.03257
CAVIN1	0.00021	KMT5B	0.006947	CXADR	0.032652
LINGO1	0.000211	CIAO1	0.006974	EZR	0.032709
CCNF	0.000212	PLA2G4F	0.006976	ERAL1	0.032777
CDIPT	0.000216	ZNF761	0.006976	RRNAD1	0.032808

GPRC5A	0.00022	SPG21	0.007018	NFE2L1	0.032925
TPMT	0.000224	SMC4	0.007019	ZFHX3	0.03299
CDT1	0.000224	ATP1B3	0.00702	SLC35E3	0.033051
SLC25A30	0.000226	EXO1	0.007025	TMOD1	0.033081
EIF5	0.000226	KLF12	0.007053	LOXL1	0.033081
BCAR1	0.000229	MAN2C1	0.007059	ADCY7	0.033081
SRSF5	0.00023	GAPDH	0.00708	MPLKIP	0.033082
RITA1	0.000232	TRAPPC5	0.007093	STN1	0.03315
MAP4K3	0.000235	MAPK13	0.007097	RLF	0.033208
GSN	0.000237	USH1G	0.007127	RAB8B	0.033222
RPS8	0.000245	GNL3LP1	0.007134	NFKBIE	0.033247
MRPS34	0.000254	ASNS	0.007134	ALKBH2	0.033475
MKI67	0.000255	EMC2	0.007134	SNHG30	0.033503
C5orf30	0.000255	PREX1	0.007167	LUC7L2	0.033537
SH2B2	0.000255	PRKAR1B	0.0072	GEMIN7	0.033548
TSC22D1	0.000255	MCRIP1	0.00721	MICALL2	0.033563
TLCD2	0.000255	PAM	0.007216	GPR157	0.033687
DARS1	0.000257	ARID1B	0.007223	NHSL1	0.033687
WSB2	0.000257	MEX3B	0.007234	BAZ1B	0.033687
ALG2	0.000262	ZNF185	0.007238	PARP10	0.033687
AC068888,1	0.000269	JPT1	0.007346	CHCHD6	0.033691
PHGDH	0.000271	LMNB1	0.007357	PKN3	0.033756
NCAPD2	0.000272	TNIP2	0.007377	HOMER3	0.033756
TPBG	0.000275	GABBR1	0.00738	NSUN5	0.033836
RPL19	0.000275	PCDH1	0.007399	CD55	0.033865
MCM10	0.000278	ULBP1	0.00744	SFXN1	0.033895
PCGF5	0.000278	SNU13	0.007461	SOX13	0.033943
TGM1	0.000286	RPL35A	0.007497	NACA	0.033995
ESAM	0.000287	MCM7	0.007497	PARP16	0.034197
MAFK	0.000288	CAV1	0.007497	BPNT1	0.034412
DTNA	0.000289	TXNDC5	0.007552	TMEM161A	0.034412
ZNF561	0.000293	SLC19A2	0.007564	SMDT1	0.034412
EIF1	0.000296	SIN3A	0.007564	NPEPL1	0.034445
MRPS11	0.000297	YBX3	0.0076	BCL3	0.034449
ZNF629	0.000298	MRRF	0.007624	NIPA2	0.034522
CASP8AP2	0.0003	MPI	0.007629	EZH2	0.034527
PPP1R3B	0.000301	PIH1D1	0.007653	TLCD1	0.034551
BLM	0.000304	UBE2V1	0.007653	MRPS26	0.034551
EPPK1	0.000305	TLN2	0.007655	TIMP1	0.034551
NUP35	0.000309	FAM162A	0.007727	PROM2	0.034555
COQ5	0.000312	NR4A3	0.007738	ACACB	0.034555
RPL31	0.000316	MAP2K2	0.007745	COA3	0.034555
ZNF48	0.000317	DYNLRB1	0.007771	MRPL9	0.03469
RPL10	0.000323	RPS25	0.007947	PSMD14	0.03469
NCAPG	0.000327	ILF3	0.007947	TBX2	0.03469
PKP1	0.000333	NDUFS8	0.007981	PRKX	0.03469
RHOD	0.000334	ACBD6	0.007988	CDC25B	0.034718

NATD1	0.000336	MINPP1	0.007988	PIDD1	0.034742
PLOD3	0.000339	PISD	0.007991	C5orf22	0.034831
RPS13	0.000344	RNF144B	0.008047	NSUN3	0.034849
PNMA1	0.000344	NPAS2	0.008054	RNF216	0.034881
HJURP	0.000346	DNAJB11	0.008061	PNPLA8	0.034881
CALU	0.000352	OGDH	0.008073	TMEM109	0.034881
ZSWIM7	0.000352	CCSAP	0.008077	ANKLE2	0.034894
BCOR	0.000352	VEGFA	0.008077	WDR48	0.034898
C18orf54	0.000352	RFC3	0.008099	EMG1	0.034898
CIART	0.000353	PQBP1	0.008099	THOC5	0.034898
GAN	0.000354	SCFD2	0.008121	HUNK	0.035171
AHCY	0.000362	WASHC2C	0.008159	SLF1	0.035179
EIF3H	0.000374	HK2	0.008178	DEDD	0.035216
MMS22L	0.000378	NR1D2	0.008178	CNOT8	0.035216
TMED2	0.000378	STK25	0.008212	RAB4A	0.03523
PRMT6	0.000379	LINC01106	0.008256	SLC1A4	0.03523
PIK3R2	0.000379	PRR19	0.008256	RSL24D1	0.035284
WWC1	0.000382	ATP8B1	0.008267	ZNF630	0.035325
CIAO2B	0.000382	SMC3	0.008268	PRLR	0.035424
SNX17	0.000382	ABT1	0.008344	RPP25	0.035424
B3GAT3	0.000384	PARP8	0.008364	SLC5A6	0.035488
RBM18	0.000385	FBXO25	0.008381	ENTPD7	0.035488
AIF1L	0.000386	ATG4A	0.008433	NIN	0.035488
NCLN	0.000396	CNOT6	0.008448	PJA1	0.035488
IFI30	0.0004	NOL6	0.008505	JKAMP	0.035514
PDXK	0.0004	ACY1	0.00853	KLF16	0.035514
LANCL1	0.0004	FEN1	0.008565	TP53INP2	0.035514
ATAD3B	0.000406	CCNH	0.008749	FCF1	0.035638
OAF	0.000406	UGP2	0.008783	PUS3	0.035802
CABLES1	0.000409	SLC35B2	0.008783	DDT	0.035859
MGST1	0.000414	AURKB	0.008794	GALNT7	0.035863
DUSP5	0.000417	DEK	0.00882	PRKAB2	0.035878
MGAT1	0.000419	CNOT6L	0.008876	ISOC1	0.035886
ILVBL	0.000424	VANGL1	0.008889	CENPJ	0.035957
CCDC85B	0.000432	RPS15	0.008911	ALDOA	0.035957
MYO6	0.000439	CDK5RAP2	0.008929	RIBC2	0.036011
RRM2B	0.000439	TONSL	0.008936	PTPMT1	0.036047
BARD1	0.000441	AACS	0.008936	AC139099,1	0.036047
FURIN	0.000442	UBE2G2	0.008936	FGD5-AS1	0.036048
EYA2	0.000442	AC239868,1	0.008991	MTERF2	0.036048
CCP110	0.000449	ARHGAP11B_2	0.008991	CDK1	0.036051
MRPS30	0.000449	AAAS	0.008996	ZNF362	0.036106
SMAGP	0.000449	CDCA8	0.009019	METTL23	0.036106
EEF1G	0.000452	CLK3	0.009199	C7orf26	0.036152
RAD18	0.000457	RPL21	0.00924	LAMP1	0.036158
GABRP	0.000457	WWC3	0.009317	RECQL4	0.036203
TTK	0.000457	MYH10	0.009317	LAMC2	0.036224

SGPL1	0.000457	GPSM2	0.009321	LRRC41	0.036243
LONRF3	0.000457	ALG10B	0.009321	RUSC1	0.036326
TRIM24	0.00046	ARHGAP11A	0.009321	AMPD2	0.03635
AHNAK2	0.00046	TAF1C	0.009321	FXR2	0.03635
BUB1	0.000467	KCNK5	0.009383	YLPM1	0.036451
MEAK7	0.000467	PGM2L1	0.009383	POLR2F	0.036462
TMED4	0.000468	B4GALT7	0.009389	TRIM62	0.036498
RRAS2	0.000469	SELENOF	0.009447	UBE2Q1	0.036498
TICRR	0.000473	MTND2P28	0.009455	GAS5	0.036498
NTPCR	0.000474	ENTPD1-AS1	0.009524	CLPP	0.036498
GAS2L3	0.000492	B3GALT6	0.009569	PCED1A	0.03651
RPL27A	0.000511	FAM111B	0.009569	RABAC1	0.036521
SF3B2	0.000522	WDHD1	0.009582	PARM1	0.036541
RPS28	0.000526	RPS29	0.009635	AL365181,3	0.036577
KIF26A	0.000529	STX7	0.009645	RRM2	0.036577
QKI	0.000538	E2F1	0.009645	TET3	0.036577
RBM12B	0.000538	S100A4	0.009707	MAF1	0.036577
HNRNPF	0.000543	ARF1	0.009793	FJX1	0.036577
RNF19B	0.000544	SLC12A8	0.009795	ZNF106	0.036577
RAB3IL1	0.000561	AC008966,1	0.009806	CISD3	0.036577
GPRC5C	0.000561	LAMTOR5	0.009851	DUS3L	0.036577
AC243919,1	0.000562	COPS3	0.009851	CDH24	0.036592
KLHL24	0.000581	RPS23	0.009865	SERAC1	0.036681
PRIM2	0.000581	ATG4D	0.009883	LRP5	0.036769
RPL13	0.000586	PRC1	0.009886	ACTR1B	0.036779
SLC25A6	0.000586	RCN1	0.009959	NR1D1	0.036816
RIOK1	0.000588	MOAP1	0.010019	CKAP5	0.036824
TP53I11	0.000596	PMPCA	0.01004	UNC13D	0.036838
LAPTM4B	0.000597	NEPRO	0.010103	F12	0.036843
SLC38A1	0.000597	MAPK9	0.010103	FBXW5	0.036923
ANK3	0.000601	DHRS2	0.010103	FBXO45	0.036938
ITPR2	0.000601	GABPB1	0.010111	SLC25A40	0.03694
EEF1D	0.000602	FES	0.010116	UBE2H	0.03694
RTKN2	0.000602	C5orf38	0.010171	SLC9A6	0.03694
RAB12	0.000604	EMP2	0.010171	IMPDH2	0.036964
NHP2	0.000604	TK1	0.010178	VSIG10L	0.036964
SLC9A3R2	0.000609	RPL13A	0.010196	SLC2A11	0.036964
RPL39	0.000609	RPS27A	0.010239	GDI1	0.036964
KIFC1	0.000627	PDE4DIP	0.010241	SURF4	0.037166
UXS1	0.000633	OPN3	0.010253	TRIM21	0.037174
IMMP2L	0.000634	MTX2	0.010256	MAPK8IP1	0.037174
DESI1	0.000637	RRAGD	0.01027	DOCK7	0.037218
CGREF1	0.000638	RRP36	0.010271	EXOSC4	0.037354
PRR11	0.000638	SH2D3A	0.010271	AGTRAP	0.037379
PTRH1	0.000641	DIAPH1	0.010298	FADD	0.037395
NQO1	0.000648	ANAPC10	0.010375	CA11	0.037395
ATP5F1B	0.000657	SRGAP2C	0.01038	POLR1C	0.037439

PPIP5K1	0.000657	EOMES	0.01038	DDIT4	0.037439
KCTD13	0.000657	ANAPC16	0.010397	TMEM134	0.037439
DLC1	0.000661	SYDE2	0.010409	RPL28	0.037489
PBK	0.000662	QSOX1	0.010409	BBIP1	0.037581
ARHGAP12	0.000669	ADCY6	0.010459	COX7B	0.037581
BLCAP	0.000672	ZNF714	0.010476	RBBP5	0.037594
ZNF219	0.000673	RPL11	0.010518	TGFBR2	0.037594
CENPO	0.000676	DHRS13	0.01052	RNFT2	0.037594
RNPS1	0.000678	ZNF100	0.01052	PALB2	0.037594
FSTL3	0.000678	CAPN2	0.010527	TUBB3	0.037594
KIAA0930	0.000685	RAB10	0.010527	ARHGAP19	0.037793
AC004943,2	0.000685	ANXA4	0.010527	PPP6R2	0.037834
DHTKD1	0.000689	EMC3	0.010527	APOOL	0.03784
QTRT1	0.000689	PACSIN2	0.010527	MRPL4	0.037997
TARS2	0.00069	PIK3C2B	0.010551	RGL2	0.038084
GLB1L2	0.00069	MSH2	0.010584	CSNK1D	0.038084
RASGRP1	0.00069	UQCC3	0.010584	ADA	0.038084
RPL26	0.00069	NEMP1	0.010584	AASDHPPT	0.038259
PON2	0.000696	PPP2R5D	0.010589	PLD2	0.038259
CREB3L2	0.000696	SH3PXD2A	0.010604	EIF6	0.038284
PHF19	0.000711	LUC7L3	0.010657	TRMT61B	0.038384
MANBA	0.00072	NR3C1	0.010688	ATAD5	0.038397
EPCAM	0.000722	PLA2G12A	0.0107	MAX	0.038417
MBNL3	0.000722	DSTYK	0.010806	CRLF1	0.038433
RERG	0.000737	PVR	0.010806	SLC35F2	0.038449
ADSL	0.000747	ZNF213	0.010825	RTTN	0.03849
ANTKMT	0.000751	POLA2	0.010827	BRI3	0.038658
NTN1	0.000756	H2AW	0.010898	JAK1	0.03879
RACGAP1	0.000756	SF3A1	0.010995	WDTC1	0.038935
IFNGR2	0.000764	MRPL1	0.011003	TMEM70	0.038935
SGO1	0.000787	CARD10	0.011003	GSTK1	0.038952
RPLP0	0.000788	PEMT	0.01101	AL118506,1	0.038959
CHAF1A	0.000788	RPRD2	0.011017	MAP3K5	0.038979
ZFAND1	0.000794	PPM1F	0.011193	SMPD1	0.038979
DMAC1	0.000798	GPR160	0.01122	MNX1-AS1	0.03899
S100A11	0.0008	B4GALNT4	0.011265	ADK	0.039104
KAT7	0.000804	RPL10A	0.011266	CTBP2	0.039113
PKP3	0.000808	KDM6B	0.011279	C20orf27	0.039201
MTG1	0.00081	SLC2A6	0.011285	TAP2	0.039268
ATP6V1D	0.00081	ZNF395	0.011358	ESD	0.039268
CDK2	0.000824	RAD21	0.011388	NT5DC2	0.039366
SRGAP2	0.000824	MMS19	0.011434	RIPK1	0.039431
PPP1R14B	0.000824	TIAL1	0.011478	RTL8A	0.039431
REV3L	0.000837	RPL23	0.01149	C4orf3	0.039506
IPPK	0.000837	PEBP1	0.0115	TMEM209	0.039511
RPL32	0.000849	NME2	0.01153	TOMM20	0.039534
GALNT6	0.000852	ZPR1	0.011539	BAZ2B	0.039628

FREM2	0.000852	GSPT1	0.011543	HOOK3	0.039628
IGSF3	0.000863	FAT4	0.011547	DYNLL2	0.039629
PRDX3	0.000869	NPAT	0.011547	AK3	0.039661
H3C10	0.000869	RBM39	0.011547	TP53BP1	0.039671
RAD23A	0.000873	ITGB4	0.01155	SLC25A32	0.039695
CHCHD10	0.000873	POMGNT1	0.011591	RNF208	0.039764
LASP1	0.000876	NAXE	0.011605	ARHGEF2	0.039788
RPS11	0.000876	ZNF286A	0.011653	ZNF672	0.039936
CD320	0.000882	MTIF2	0.011663	WDR97	0.039936
N4BP2	0.000884	AP003108,2	0.011683	DMTN	0.039985
PPIB	0.000889	PTPRG-AS1	0.011689	NCSTN	0.040082
SMC2	0.000892	TMEM223	0.01172	TROAP	0.040082
PRIM1	0.000902	NDUFAF8	0.011752	ADGRF4	0.040208
ITGA3	0.00091	GRINA	0.011836	PHC1P1	0.040208
CDH3	0.000915	TRABD	0.011842	NIT2	0.040211
DTL	0.000933	LRRC61	0.011921	EIF3L	0.040211
RCC1	0.000937	OSER1	0.011921	NKAP	0.040211
CMIP	0.000941	PLCB3	0.01196	CCNK	0.040221
TJP1	0.000945	PTPN14	0.012021	RPL12	0.04027
WARS1	0.000955	LHX1-DT	0.012021	LRRC15	0.040313
LINC00294	0.000957	DGKZ	0.012065	BUD23	0.040394
F8A1	0.000957	ZNF335	0.012081	UBE2V2	0.040394
ISYNA1	0.000961	SIK2	0.012173	FBRSL1	0.040394
ATP6V1E1	0.000962	TANC2	0.012197	TERF2IP	0.040394
G2E3	0.000963	ASF1B	0.012197	ACSF3	0.040394
IGSF9	0.000968	LIN54	0.012276	YJU2	0.040394
SYNE2	0.000968	ORAI1	0.012286	NDUFA13	0.040394
HMMR	0.000977	DHODH	0.012286	CAPNS1	0.040394
SLC9A3-AS1	0.000977	SIDT1	0.012373	HDHD5	0.040394
OSGIN1	0.000989	SCAND2P	0.012373	GPRC5D-AS1	0.040432
SAP130	0.000994	INO80B	0.012402	TMC5	0.040466
RPS10	0.001001	FANCE	0.012402	BCLAF1	0.040533
RAD54L	0.001011	MOV10	0.012445	CTCF	0.040533
TRGC1	0.001024	RBMXL1	0.012629	PARP2	0.040533
PREB	0.001026	NPDC1	0.012755	TIMM17A	0.04061
UHRF1	0.001028	NFKB1	0.012822	QPRT	0.040651
DLGAP5	0.001028	DOP1B	0.012822	ANKRD10	0.040788
KCTD20	0.001031	CDKN1B	0.01303	SEC61G	0.040905
UROD	0.001036	TMED1	0.013053	PLEKHH1	0.041103
DUSP23	0.001046	ACTG1	0.013077	BRIX1	0.041116
MICA	0.001046	TSEN2	0.013175	SNHG6	0.041143
MEST	0.001046	DIMT1	0.013175	C15orf61	0.041154
HSP90B1	0.001046	DNMT3A	0.013271	UBE2J2	0.04121
KTN1	0.001046	TMEM139	0.013271	SON	0.04127
DNAJB6	0.00105	DNMT3B	0.013271	NPC2	0.041271
C1orf122	0.001068	NBPF1	0.013313	VPS45	0.041316
P4HA1	0.001068	SESTD1	0.013313	MRPS2	0.041316

TMX2	0.001068	JTB	0.013315	CEMIP2	0.04142
MICALL1	0.001073	LIN52	0.013341	CLTB	0.04145
RIMS3	0.001078	UBE2S	0.013357	LGMN	0.041522
BMP1	0.00108	H2BC18	0.013416	FAM92A	0.041617
SORBS3	0.00108	EIF5A	0.013543	HSPG2	0.041681
PLK3	0.001086	PARD6G	0.013558	INHBB	0.041681
BUB1B	0.001093	KDF1	0.013561	CNPPD1	0.041681
TRIM56	0.001098	IFITM3	0.013561	NOP16	0.041681
LRSAM1	0.001098	MYH9	0.013588	RDH11	0.041681
SP1	0.001113	PPL	0.013624	WDR61	0.041681
JMJD6	0.001113	SCX	0.013703	PLOD1	0.041683
PHLPP1	0.001113	SKA1	0.013727	ODC1	0.041809
ITPA	0.001113	MPST	0.01381	CENPT	0.041916
MRPS6	0.001113	GFM1	0.01382	AKNA	0.042044
RPL36A	0.00112	SMPDL3B	0.013853	WRNIP1	0.042047
NRP1	0.001123	C1D	0.013862	FLNA	0.042205
TBC1D9	0.001128	PTK6	0.013879	NEU3	0.04221
SF3B5	0.001128	SLC7A11	0.013955	SEC11A	0.04221
PRPF39	0.001128	TMED9	0.013955	RRP1	0.042257
LINC02732	0.001134	ACTR5	0.013966	AFAP1L2	0.042308
RPS17	0.001138	SRSF8	0.013988	AC004264,1	0.042368
LINC00365	0.001148	RAI1	0.013989	ZFP90	0.042397
CDK18	0.001156	PIM1	0.013995	ZNF480	0.042397
HS6ST1	0.001156	SEPHS2	0.013995	CTNND2	0.042413
MYDGF	0.001161	SERP1	0.014125	DHFR	0.042413
UBTF	0.001165	TGS1	0.014133	FANCG	0.042413
RPL7	0.001169	COIL	0.014168	AC010168,2	0.042577
DDX49	0.001175	KLF7	0.014187	CNP	0.042607
JPH2	0.001175	GHITM	0.014191	ELMO3	0.042634
MORC4	0.001175	NDUFAF4	0.014232	MRPS24	0.042708
GOLGB1	0.001176	TMEM86A	0.014269	BOP1	0.042708
SLC25A24	0.001179	FHOD1	0.014318	DIPK1A	0.042796
PLRG1	0.001198	LRCH4	0.014377	ZBTB8A	0.042825
CTXN1	0.001202	DUSP16	0.014398	CDC25C	0.042826
RPS6	0.001206	WDR90	0.014448	CAB39L	0.043021
METRNL	0.001206	NUDC	0.014454	ACSL1	0.043127
SMURF1	0.001207	FAM210B	0.014547	SGF29	0.043251
TSPAN13	0.001224	DNAJC9	0.014562	PEX16	0.043337
PITX1	0.001228	YARS1	0.014572	BTF3	0.043337
ZNF444	0.001241	TFPI	0.014572	MOB2	0.043337
ZDHHC24	0.00126	SRM	0.014617	FADS3	0.043337
ZIC5	0.00126	EML5	0.014728	NME7	0.043498
MCM5	0.001269	KIF4A	0.014794	EIF3M	0.043581
APLP2	0.001271	NOL3	0.014841	SRP14	0.043581
PAXIP1-AS1	0.001275	BRPF3	0.014909	LRRC37B	0.043602
AMOTL2	0.001277	PALM3	0.014917	TBC1D8B	0.043678
AL732372,1	0.001279	COL18A1	0.014922	DBF4	0.043854

DNMT1	0.001279	CDC45	0.014996	AC092718,3	0.043871
SLC12A6	0.001285	SEMA3F	0.015026	GPRIN1	0.043956
NOB1	0.001288	SLC7A2	0.015082	ZNHIT2	0.044056
RPS15A	0.001298	RPL35	0.015088	MRC2	0.044056
UROS	0.001299	IKZF4	0.015088	ARL6IP4	0.044098
HMOX1	0.001299	SYNGR2	0.01516	NECTIN1	0.044248
SEC62	0.0013	EIF3F	0.015178	VPS28	0.044405
FANCA	0.001301	PHLDB2	0.015228	CD99	0.044433
LBR	0.001302	RND1	0.015228	ATP2A3	0.044481
ERLIN1	0.00131	POGLUT3	0.015233	ZNF473	0.044481
ENTPD5	0.00131	SRGAP2B	0.01533	CFAP410	0.044481
NECAB3	0.001311	KHK	0.015342	CLPB	0.04454
ZBTB21	0.001351	FAM72A	0.015361	MAP3K10	0.04454
NNT	0.001354	SNX29	0.015361	PFDN4	0.04454
TSPYL1	0.001361	FOXRED2	0.015361	DIDO1	0.04454
RHPN1	0.001367	DEF8	0.015411	ARHGEF26	0.044573
TTC19	0.001368	AC012615,1	0.015419	PCDHGB5	0.044573
NSD2	0.001377	CD47	0.015452	H1-0	0.044599
SARS1	0.001396	TTI1	0.015452	RESF1	0.044791
LPCAT1	0.001396	TCEA1	0.015465	PPP1R10	0.044926
SFMBT2	0.001408	TMEM161B	0.015478	ACAP3	0.045093
TMEM205	0.001408	SMG6	0.015478	TRMT12	0.04522
PSTPIP2	0.001412	SCN8A	0.015585	FAM47E-STBD1	0.045237
CDR2L	0.001412	PRAF2	0.015657	GLI3	0.045237
TRUB2	0.001426	CCNB1	0.015668	LUC7L	0.045237
GPAM	0.001426	DUS1L	0.015668	AC009163,7	0.045237
FAM234B	0.001426	UQCR11	0.015679	EGLN1	0.045465
ZDHHC23	0.00146	PROCR	0.0157	HIGD2A	0.045522
TUBA1C	0.001466	ZFP62	0.015756	COX4I1	0.045522
MRPS25	0.001476	MLPH	0.015789	PAX9	0.045541
TMEM259	0.001486	SNX12	0.015796	CUEDC2	0.045585
GALE	0.001507	LACC1	0.015807	COMTD1	0.045591
ANKRD50	0.001511	C6orf120	0.01582	GMNN	0.045594
EPS8	0.001517	RASD1	0.01582	TUSC2	0.045779
EIF2B2	0.001524	GABPB1-IT1	0.01582	VAMP1	0.04578
FAM207A	0.001524	AVPI1	0.015842	SMCHD1	0.04578
BCL2L11	0.001531	SLC25A38	0.015872	PRRG4	0.045926
ACBD7	0.001531	QPCTL	0.015889	CCT4	0.046104
GTPBP10	0.001533	DNAJC22	0.015922	POP7	0.046125
GINS2	0.00154	PCMTD2	0.015922	IFITM1	0.046125
ZDHHC14	0.001543	TMUB1	0.015922	CDC34	0.046153
HEXB	0.001544	LINC01123	0.015923	ISOC2	0.04621
PTDSS2	0.001544	ERI2	0.015986	DNAJB1	0.046213
AP1S3	0.001546	CTTN	0.015993	PSPH	0.046246
TOP2B	0.001546	ATG16L1	0.016006	MSL2	0.046292
STIP1	0.001546	CNBP	0.016006	UBA7	0.046314
KTN1-AS1	0.001562	ITPK1	0.016006	NDUFV3	0.046396

RPS9	0.001564	TIMM44	0.016006	SP100	0.046644
SFT2D1	0.001581	NAP1L1	0.016008	FAM13B	0.046644
RPS24	0.001581	STUB1	0.016008	CDC42BPG	0.046644
HOMER2	0.001581	RASSF8	0.016124	RRAGB	0.046644
SMTN	0.001587	EMC7	0.016124	UGCG	0.046817
FBXO17	0.001595	EMC10	0.016214	MRE11	0.046817
URM1	0.001597	DCAF4	0.016216	TMEM99	0.046817
EMC8	0.001604	TRIP4	0.01622	DNAJC5	0.046817
TMPRSS13	0.001605	PNRC1	0.01626	TBX3	0.046822
HOOK2	0.001606	NGLY1	0.016275	ETFA	0.046852
UBE2D3	0.001613	RHOU	0.016309	LMX1B	0.046868
PRKAR2B	0.001617	MYO9B	0.016309	KNTC1	0.046868
LYPD6B	0.001637	ZBTB38	0.016315	TSR3	0.046868
ASTN2	0.001637	FHL2	0.016348	PSIP1	0.046917
APMAP	0.001662	PLK2	0.016348	ZNRD2	0.046921
RPL7A	0.001663	TEX264	0.016387	FAU	0.046964
THUMPD3-AS1	0.001665	SCRN2	0.016534	ZNF837	0.046982
PGAM1	0.001665	FAM221A	0.016563	TXNRD3	0.047025
TSNARE1	0.001683	IL15RA	0.016576	STK26	0.047184
RNF145	0.001686	PHB2	0.016577	LZTR1	0.047268
ALKBH5	0.001687	AP1AR	0.016624	CCDC126	0.047348
CPE	0.001688	TSPAN1	0.016637	TASP1	0.047357
TRIB2	0.001708	ZBED3	0.016646	COX15	0.047368
LNCAROD	0.001724	CLBA1	0.016836	TAF6	0.047402
CD63	0.001728	TMCO6	0.016969	KRT10	0.047402
RFC1	0.001735	RSU1	0.016969	RUNX2	0.047416
CYLD	0.001735	RBM12	0.016969	TMBIM4	0.047454
UBA2	0.001746	SLC1A5	0.016988	LRRC3	0.047476
RPL29	0.001748	DSN1	0.016998	NEAT1	0.047493
PAFAH1B1	0.001748	AK2	0.016999	FAM86C2P	0.047493
ALS2	0.00177	SCARA3	0.016999	FAM72D	0.047501
NFS1	0.00177	TRIB1	0.016999	FBXO9	0.047501
TEAD1	0.001829	CFAP298	0.017036	CRIM1	0.047526
TMEM159	0.001843	DTD2	0.017111	SLC39A4	0.047711
ABCG1	0.001843	CTC1	0.017111	AP001931,2	0.047738
ICMT	0.001861	TASOR	0.01715	CRTAP	0.04781
NDUFA8	0.001861	PTBP3	0.017205	CENPL	0.047827
CES2	0.001861	SRC	0.017205	NAT14	0.047917
SIRT7	0.001881	CYSRT1	0.017339	BSPRY	0.047956
TAF9B	0.001881	VPS33B	0.017368	C18orf32	0.047956
CDC25A	0.001932	MIS18A	0.017455	SIRT6	0.047988
SLC29A2	0.001937	CCNG2	0.017462	RABEPK	0.048021
ACOX1	0.001937	SMARCB1	0.017462	RPL7L1	0.048057
EPB41L1	0.001937	LSR	0.017471	CDC42BPA	0.048235
PAQR4	0.001944	NECAB1	0.017472	TMEM129	0.048235
MRPS12	0.001945	ZNF511	0.017537	EFNB2	0.048237
ARL4A	0.001955	TRPM6	0.017594	DENND6B	0.048319

MAPKAP1	0.001955	CDK8	0.017605	LMAN2	0.048475
KATNAL1	0.001955	SLC16A6	0.017815	TUBB6	0.048475
ZNF695	0.001969	ZNF420	0.01787	ATP6V0D1	0.048582
ATP2B1	0.00198	PSMF1	0.017924	ABCC10	0.048694
FAM174A	0.001985	KIAA0513	0.017947	CBX8	0.04871
VAT1	0.001994	BRAT1	0.017957	ATP5PO	0.048761
SEMA4A	0.00201	TOLLIP	0.017957	SNX2	0.048823
PYCR3	0.00201	CST3	0.018017	DEXI	0.048836
PCNX4	0.002011	EPB41L4A-AS1	0.01806	C8orf82	0.048874
FOXJ2	0.002016	SLC35A2	0.018191	NUMBL	0.048893
MKNK2	0.002017	HMGN4	0.018217	GOLGA8A	0.049279
ERCC6L	0.002017	C8orf33	0.018255	OGFRP1	0.049282
CEP78	0.002026	MIR9-3HG	0.018255	E2F2	0.04935
INO80E	0.002074	ZNF551	0.018255	COX7C	0.049355
DAB2IP	0.002092	DLG5	0.018328	KIF27	0.04942
EEF1B2	0.002094	CEP295	0.018353	GNG10	0.049424
DEPDC1B	0.002103	ZFYVE1	0.018491	ETV3	0.049669
CIAO3	0.002108	XXYLT1	0.018547	ADD1	0.049752
RELT	0.002138	NDUFS4	0.018547	HECA	0.049894
ZGRF1	0.002149	AADAT	0.018575	CDC37	0.049929
FOXO3	0.002157	EPB41L2	0.018575	COQ8A	0.049942
FIS1	0.002162	RPL24	0.018639	MYEOV	0.049942
				WDR73	0.049982

Table S10: E2F/DREAM targets associated with transcriptional clusters 1, 2 and 3

Cluster	Gene name	DREAM target	Cluster	Gene name	DREAM target	Cluster	Gene name	DREAM target
1	ARHGAP11A	yes	2	ANLN	yes	3	BAZ1B	yes
1	ARHGAP19	yes	2	APOBEC3B	yes	3	BCLAF1	yes
1	ASPM	yes	2	ATAD2	yes	3	BIRC5	yes
1	ATAD5	yes	2	BRCA1	yes	3	BRIX1	yes
1	AURKA	yes	2	BRIP1	yes	3	CCSAP	yes
1	AURKB	yes	2	C18orf54	yes	3	CDC25A	yes
1	BLM	yes	2	CASP8AP2	yes	3	CDR2	yes
1	BRCA2	yes	2	CDC25C	yes	3	DBF4	yes
1	BUB1	yes	2	CDK2	yes	3	FAM72D	yes
1	BUB1B	yes	2	CDK5RAP2	yes	3	FKBP5	yes
1	C4orf46	yes	2	CENPU	yes	3	GABPB1	yes
1	CCNA2	yes	2	CEP295	yes	3	GPD2	yes
1	CCNB1	yes	2	CIT	yes	3	IFRD1	yes
1	CCNF	yes	2	CKAP2	yes	3	ILF3	yes
1	CDC20	yes	2	CKAP2L	yes	3	JADE1	yes
1	CDC25B	yes	2	CLSPN	yes	3	LBR	yes
1	CDC45	yes	2	DCLRE1A	yes	3	MIS18A	yes
1	CDC6	yes	2	DGCR8	yes	3	PLK1	yes
1	CDCA2	yes	2	DHFR	yes	3	POLR3K	yes
1	CDCA5	yes	2	DHTKD1	yes	3	RAD18	yes
1	CDCA8	yes	2	DIAPH3	yes	3	RAD23A	yes
1	CDK1	yes	2	DSN1	yes	3	RCC1	yes
1	CDT1	yes	2	DTL	yes	3	RFC3	yes
1	CENPA	yes	2	E2F2	yes	3	RNPS1	yes
1	CENPE	yes	2	ECT2	yes	3	SKA3	yes
1	CENPF	yes	2	FAM111A	yes	3	SLBP	yes
1	CENPJ	yes	2	FAM111B	yes	3	SMCHD1	yes
1	CENPL	yes	2	FANCD2	yes	3	SRSF2	yes
1	CENPO	yes	2	FANCE	yes	3	SYNCRIP	yes
1	CEP55	yes	2	FANCG	yes	3	TCEA1	yes
1	CHAF1A	yes	2	GEN1	yes	3	THOC1	yes
1	CHEK1	yes	2	GPSM2	yes	3	TMEM109	yes
1	CIP2A	yes	2	GTSE1	yes	3	UNG	yes
1	CKAP5	yes	2	HJURP	yes	3	ZNF695	yes
1	CTCF	yes	2	HNRNPF	yes	3	CYP1B1	
1	DCLRE1B	yes	2	IQGAP3	yes	3	HSPA1B	
1	DDIAS	yes	2	KIF18A	yes	3	NFKB1	
1	DEPDC1	yes	2	KIF24	yes	3	ELOVL6	
1	DEPDC1B	yes	2	KIF2C	yes	3	MTMR12	
1	DLGAP5	yes	2	KIFC1	yes	3	RBM39	
1	DNAJC9	yes	2	KNL1	yes	3	DOCK7	
1	DNMT1	yes	2	KNTC1	yes	3	ANKLE2	
1	DNMT3B	yes	2	LRRCC1	yes	3	CEBPB	
1	E2F1	yes	2	MCM5	yes	3	TRMT61B	

1	ERCC6L	yes	2	MRE11	yes	3	TRIM25	
1	ESCO2	yes	2	NDC80	yes	3	FOXN3	
1	ESPL1	yes	2	NUSAP1	yes	3	AIF1L	
1	EXO1	yes	2	PARP2	yes	3	TAP2	
1	EZH2	yes	2	POLA2	yes	3	TMC5	
1	FAM83D	yes	2	POLD3	yes	3	TARS2	
1	FANCA	yes	2	PRIM2	yes	3	AP1AR	
1	FANCI	yes	2	PSRC1	yes	3	MAP3K5	
1	FBXO5	yes	2	RECQL4	yes	3	ZIC2	
1	FEN1	yes	2	RFC4	yes	3	OSBPL6	
1	G2E3	yes	2	RRM1	yes	3	NUDT4	
1	GAS2L3	yes	2	RRM2	yes	3	NR1D2	
1	GINS1	yes	2	SLF2	yes	3	ZIC5	
1	GINS2	yes	2	SP1	yes	3	SMAGP	
1	GMNN	yes	2	SPC25	yes	3	SLC19A2	
1	HASPIN	yes	2	STAT1	yes	3	TYRO3	
1	HMMR	yes	2	STIL	yes	3	TTL	
1	INCENP	yes	2	TCF19	yes	3	TRIM14	
1	KIF11	yes	2	TK1	yes	3	ERLIN1	
1	KIF14	yes	2	TONSL	yes	3	YY1	
1	KIF15	yes	2	TOP2A	yes	3	ZNF496	
1	KIF18B	yes	2	TRIM45	yes	3	AXIN1	
1	KIF20A	yes	2	UBR7	yes	3	USP7	
1	KIF20B	yes	2	USP37	yes	3	ZBTB2	
1	KIF23	yes	2	WDR76	yes	3	MCMBP	
1	KIF4A	yes	2	ZGRF1	yes	3	REPS1	
1	LIN54	yes	2	ZNF100	yes	3	YJU2	
1	LMNB1	yes	2	ZNF714	yes	3	MRPS34	
1	MASTL	yes	2	ZWINT	yes	3	GSPT1	
1	MCM10	yes	2	DEDD		3	MRPL9	
1	MCM2	yes	2	MACROD1		3	BRMS1	
1	MCM4	yes	2	TMOD1		3	THAP11	
1	MCM6	yes	2	ACAP3		3	POLR3C	
1	MCM7	yes	2	ASAP2		3	POLR3F	
1	MDC1	yes	2	QKI		3	NGDN	
1	MIS18BP1	yes	2	RELB		3	UBA2	
1	MKI67	yes	2	PHACTR2		3	AVPI1	
1	MMS22L	yes	2	TACC1		3	PRPS1	
1	MSH2	yes	2	FUT9		3	EIF5	
1	NCAPG	yes	2	GPRIN1		3	CDK8	
1	NCAPG2	yes	2	ZNF430		3	RSL1D1	
1	NCAPH	yes	2	TLN2		3	ZNF639	
1	NEIL3	yes	2	IKBKE		3	FBXO45	
1	NEK2	yes	2	CNP		3	PPP2CA	
1	NEMP1	yes	2	MINDY2		3	FAM53B	
1	NPAT	yes	2	AC004943.2		3	BCL2L11	
1	NUP50	yes	2	CENPI		3	GNL3LP1	

1	ORC1	yes
1	PBK	yes
1	PKMYT1	yes
1	PLK4	yes
1	POLA1	yes
1	PRC1	yes
1	PRIM1	yes
1	PSIP1	yes
1	RACGAP1	yes
1	RAD51	yes
1	RAD54L	yes
1	RBL1	yes
1	RFC5	yes
1	RFWD3	yes
1	RTTN	yes
1	SGO1	yes
1	SHCBP1	yes
1	SKA1	yes
1	SLF1	yes
1	SMC1A	yes
1	SMC2	yes
1	SMC3	yes
1	SMC4	yes
1	SPAG5	yes
1	TACC3	yes
1	TICRR	yes
1	TMEM209	yes
1	TMPO	yes
1	TPX2	yes
1	TROAP	yes
1	TTI1	yes
1	TTK	yes
1	UBE2C	yes
1	UHRF1	yes
1	USP1	yes
1	WDHD1	yes
1	ZNF367	yes
1	ZNF473	yes
1	BRD1	
1	PPFIA1	
1	EHD4	
1	SMARCB1	
1	FAM168B	
1	VAPB	
1	AC239868.1	
1	LUC7L2	
1	MAPK9	
2	LIG1	
2	PTPRG-AS1	
2	AMOTL2	
2	WWC3	
2	PCDH1	
2	RAB27B	
2	SLX4IP	
2	EFCAB11	
2	TAF9B	
2	H1-0	
2	SRGAP2C	
2	SRGAP2B	
2	SORBS3	
2	CDKN2B	
2	EFNB2	
2	NEDD9	
2	VGLL1	
2	HELZ2	
2	NEURL1B	
2	SOWAHC	
2	SCARA3	
2	TP53I11	
2	RASGRP1	
2	KRT81	
2	JDP2	
2	RHOU	
2	TRGC1	
2	PAQR4	
2	HERC6	
2	TRIM21	
2	ZNF362	
2	C5	
2	DAB2IP	
2	FBXL3	
2	HPS3	
2	FOXO3	
2	SIDT1	
2	FHDC1	
2	PDE4DIP	
2	TIA1	
2	NCOA2	
2	SYNE2	
2	ESAM	
2	ARHGEF2	
2	PKN3	
2	ETV3	
2	NEDD4	
3	FCF1	
3	FAM72A	
3	FAM72B	
3	PHGDH	
3	LINGO1	
3	SIRT1	
3	SF3A1	
3	SRSF4	

1	NRF1	2	HIP1	
1	ARHGEF26	2	PTPN14	
1	RBBP5	2	DNAJB1	
1	UBTF	2	ZNF410	
1	OSBPL11	2	CSNK1A1	
1	NR4A3	2	FUT4	
1	FEM1B	2	DHRS2	
1	TET3	2	ZBTB21	
1	ZNF106	2	TAF6	
1	ZNF704	2	RAB11FIP1	
1	B3GNT10	2	TJP1	
1	C1orf198	2	PCM1	
1	NCOA7	2	ZNF318	
1	ZNF860	2	PPP2R5D	
1	SF3B2	2	NHSL1	
1	HUNK	2	RLF	
1	CHAF1B	2	ZBTB8A	
1	ERI2	2	TRIM16L	
